# Circular RNA FCHO2 promotes airway remodeling in COPD via regulating nuclear translocation of PTBP1 to repress the splicing of GRN pre-mRNA

**DOI:** 10.1038/s41419-025-08107-9

**Published:** 2025-11-03

**Authors:** Yanfei Liu, Yucong Wang, Yuqing Fei, Hu Xu, Cheng Xue, Liudong Li, Lei Huang, Pengcheng Liu, Renming Li, Ge Shan, Liang Chen, Dahai Zhao

**Affiliations:** 1 Department of Respiratory and Critical Care Medicine, The Second Affiliated Hospital of Anhui Medical University, Hefei, China; 2 Department of Obstetrics and Gynecology, The First Affiliated Hospital of USTC, The RNA Institute, School of Basic Medical Sciences, Division of Life Sciences and Medicine, University of Science and Technology of China, Hefei, China; 3 Department of Cardiology, The First Affiliated Hospital of USTC, The RNA Institute, Division of Life Sciences and Medicine, University of Science and Technology of China, Hefei, China

**Keywords:** Non-coding RNAs, Respiratory tract diseases

## Abstract

Circular RNAs (circRNAs) have emerged as key regulators in human diseases, yet their mechanisms of action in chronic obstructive pulmonary disease (COPD) remain largely unknown. In this study, the conserved mammalian circRNA circFCHO2 was shown to play critical roles in COPD. The expression level of circFCHO2 was significantly increased in COPD cell models, mouse models, and human lung tissue samples. Moreover, we demonstrated that circFCHO2 promotes epithelial‒mesenchymal transition (EMT) in bronchial epithelial cells and extracellular matrix (ECM) remodeling. Mechanistically, circFCHO2 binds to and facilitates the nuclear translocation of PTBP1, thereby inhibiting the splicing of GRN pre-mRNA, which reduces PGRN protein expression levels and activates the NF-κB pathway. This activation of the NF-κB signaling pathway regulates the expression of EMT and ECM remodeling-related proteins, leading to the occurrence of airway remodeling. circFCHO2 knockdown reverses cigarette smoke-induced emphysema and airway remodeling of COPD in mice. Overall, our study advanced the understanding of the molecular mechanisms by which circRNAs contribute to airway remodeling in COPD patients.

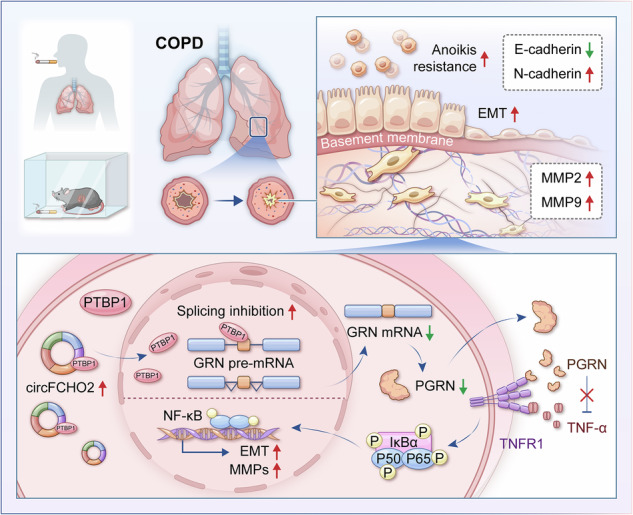

## Introduction

Chronic obstructive pulmonary disease (COPD) is a chronic progressive lung disease characterized by respiratory symptoms associated with chronic airflow limitation. It is estimated that 300 million people are affected globally, making it the third leading cause of death worldwide [1]. The Global Initiative for Chronic Obstructive Lung Disease (GOLD) defines COPD as “a disease usually caused by significant exposure to harmful particles or gases” with cigarette smoke (CS) remaining the primary environmental risk factor for COPD [2, 3].

As COPD progresses, patients experience airflow limitation and airway narrowing due to small airway remodeling, whereas alveolar destruction manifests as emphysema [4]. Epithelial‒mesenchymal transition (EMT) is one of the key mechanisms driving cigarette smoke-induced airway remodeling by affecting epithelial cell function and dynamically regulating the extracellular matrix (ECM), promoting abnormal airway structure [5, 6]. During ECM remodeling in COPD, ECM degradation dominates, leading to airway collapse and exacerbating emphysema [7, 8]. The use of inhaled corticosteroids (ICSs), the standard treatment for severe COPD, for more than 6 months has been shown to reduce the number of S100A4 and MMP9-positive cells (S100A4 and MMP9 are EMT-related proteins) in the fragmented basal membrane region of COPD patients [9]. Roflumilast (an oral PDE4 inhibitor) can restore cAMP levels, and inhibit the inflammation and EMT induced by cigarette smoke, thus alleviating airway remodeling and emphysema [10]. Exploring the molecular mechanisms involved in COPD airway remodeling is key to identifying therapeutic targets for COPD.

Circular RNA (circRNA) is a covalently closed, single-stranded RNA molecule produced by back-splicing [11–13]. Compared with linear RNAs, circRNAs are more stable because they are resistant to degradation by exonucleases, making them more promising as therapeutic targets [14]. Most circRNAs are located in the cytoplasm, where they exert their functions by adsorbing microRNAs (miRNAs), interacting with RNA-binding proteins (RBPs), or being translated into proteins. Several studies have explained the regulatory functions and mechanisms of circRNAs in COPD. For example, multiple studies have constructed COPD-related ceRNA regulatory networks and predicted potential circRNA‒mRNA‒miRNA interactions in COPD [15]. m6A-modified circSAV1 can be recognized by YTHDF1, promoting IREB2 translation and inducing ferroptosis in lung epithelial cells [16]. CircADAMTS6 can stabilize CAMK2A, promote M2 macrophage polarization and promote smoking-induced emphysema [17]. Circ0061052 competitively binds to miR-515-5p to regulate the expression of FoxC1/Snail and induces EMT in bronchial epithelial cells [18]. The molecular mechanisms by which circRNAs regulate COPD progression have yet to be further explored.

In this study, we aimed to clarify the molecular mechanisms by which circRNAs regulate airway remodeling during COPD progression. We found that the expression of circFCHO2 was significantly increased in COPD cell models, mouse models, and human lung tissue samples. Furthermore, we demonstrated that circFCHO2 promotes EMT in bronchial epithelial cells and regulates ECM remodeling. Mechanistically, circFCHO2 binds to and facilitates the nuclear translocation of PTBP1, thereby inhibiting GRN pre-mRNA splicing, reducing PGRN protein expression levels, activating the NF-κB signaling pathway, and regulating the expression of proteins related to EMT and ECM remodeling, leading to airway remodeling.

## Results

### circFCHO2, a conserved mammalian circRNA, is related to COPD

**Fig. 1:**
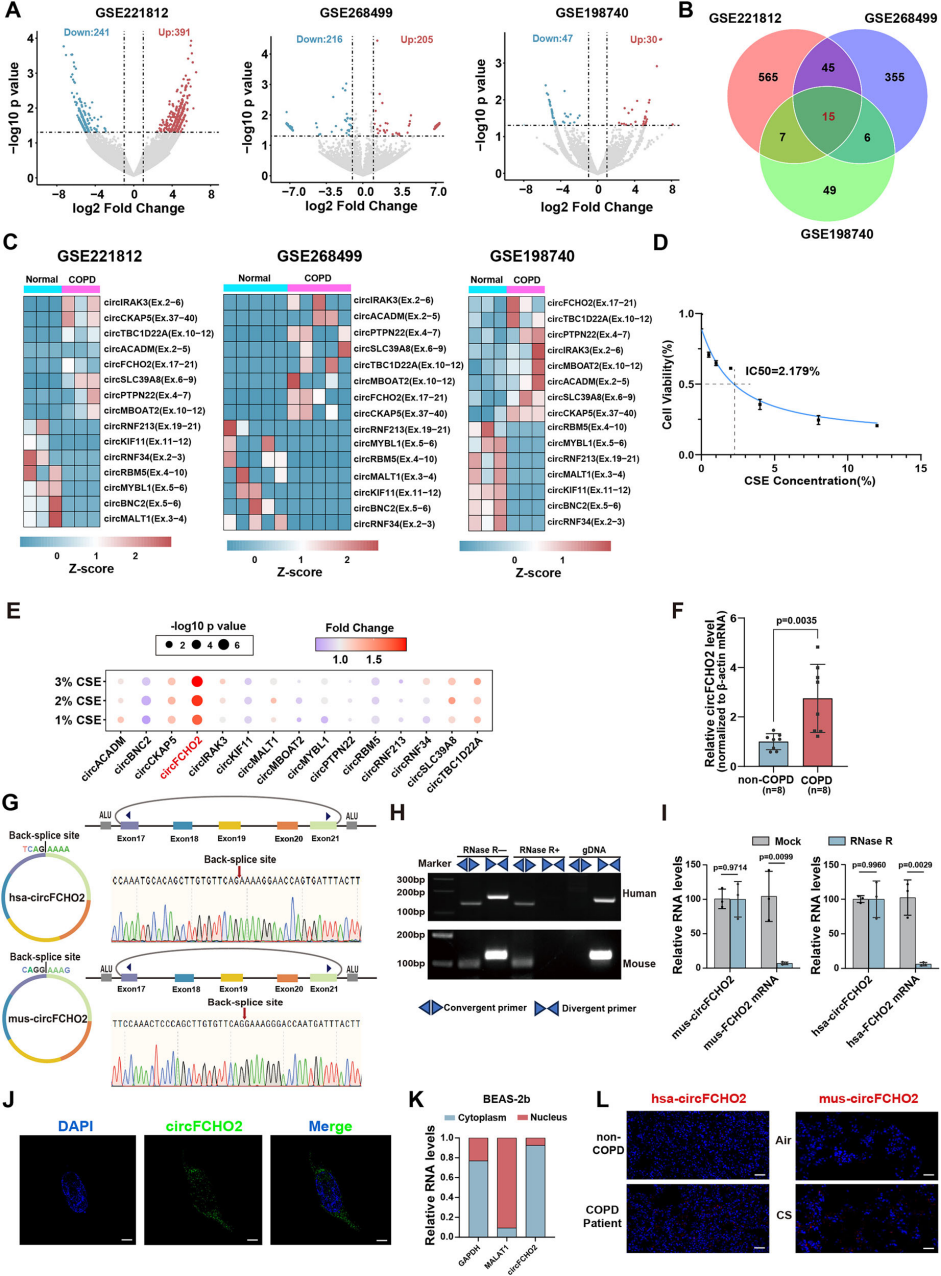
circFCHO2, a conserved mammalian circRNA, is related to COPD. **A** Volcano plots of the differentially- expressed circRNAs from three COPD-related RNA sequencing (RNA-seq) datasets (GSE221812, GSE268499, and GSE198740). Red points indicate upregulated circRNAs in COPD samples. The blue points indicate downregulated circRNAs in the COPD samples. **B** Venn diagram showing 15 overlapping differentially-expressed circRNAs from three RNA-seq datasets. **C** Heatmaps representing the expression levels of 15 differentially- expressed circRNAs in each COPD-related RNA-seq dataset analyzed via RT‒qPCR. The color bar shows the normalized level (by Z- score) of the circRNAs. **D** Line graph showing that a CSE concentration of 2.179% led to a 50% survival rate in BEAS-2B cells. **E** Heatmap showing the fold changes in the expression of the 15 identified circRNAs in BEAS-2B cells treated with 1–3% CSE. circFCHO2 showed the greatest upregulation (~2.5-fold change) among all the treatments. **F** Bar plot showing that circFCHO2 expression was upregulated in the lung tissue of COPD patients (*n* = 8) compared with that in the control group (*n* = 8). **G** Demonstration of the backsplicing sites of circFCHO2 by Sanger sequencing using divergent primers in humans and mice. The red arrows show the backsplicing sites of circFCHO2. **H** RT‒PCR analysis of circFCHO2; the back-splicing junction of circFCHO2 was successfully amplified using complementary DNA (cDNA) from humans and mice but not via genomic DNA (gDNA). **I** Bar plot showing that RNase R exonuclease assays verified the resistance of circFCHO2 cells to digestion by RT‒qPCR (*n* = 3). **J** Fluorescence in situ hybridization (FISH) of circFCHO2 in BEAS-2B cells. Representative images are shown (DAPI, blue; circFCHO2, green). Scale bar, 10 μm. **K** Bar plot showing the relative circFCHO2 expression levels in the cytoplasm and nucleus of BEAS-2B cells as determined by nucleocytoplasmic separation and RT‒qPCR. **L** Fluorescence in situ hybridization (FISH) of circFCHO2 in COPD patient samples and a mouse model. Representative images are shown (DAPI, blue; circFCHO2, red). Scale bar, 100 μm. For **D**, nonlinear regression (curve fit) was used. For **E**, **F** and **I**, *P* values were calculated via two-tailed unpaired Student’s *t* test and Welch’s *t*- test. The data are shown as the means ± SDs from independent experiments.

To investigate COPD-associated circRNAs, we analyzed differentially expressed circRNAs from three COPD-related RNA sequencing datasets (GSE221812, GSE268499, and GSE198740) and identified 15 shared differentially expressed circRNAs from these datasets. (Fig. 1A–C; S1A).

Next, we utilized cigarette smoke extract (CSE)-treated BEAS-2B cells to construct a COPD cell model for further screening of COPD-related circRNAs [19–21]. Using CCK-8 assays, we determined that a CSE concentration of 2.179% led to a 50% survival rate for bronchial epithelial cells (Fig. 1D). Among the 15 identified circRNAs, circFCHO2 showed the greatest upregulation (~2.5-fold change) with increasing CSE concentration (Fig. 1E). We also observed that circFCHO2 expression was upregulated in the lung tissue of COPD patients, suggesting that circFCHO2 may be clinically important for COPD pathogenesis (Fig. 1F; S1B).

circFCHO2 has a similar secondary structure and a high degree of conservation between humans and mice, with 87.4% sequence identity, and is derived from exons 17 to 21 of both the human and mouse FCHO2 genes The human genome encodes hsa-circFCHO2 (annotated as hsa_circ_0003571 in circBase) with a total length of 594 nucleotides, whereas the mouse genome encodes mus-circFCHO2 (currently unnamed in circBase) with a length of 591 nucleotides (Fig. S1C). The back-splicing junction (BSJ) of circFCHO2 was validated through PCR amplification and Sanger sequencing using human and mouse lung tissues, confirming its circular structure (Fig. 1G). Using divergent primers, we successfully amplified the BSJ of circFCHO2 in complementary DNA (cDNA) but not in genomic DNA (gDNA) (Fig. 1H). Additionally, RNase R exonuclease assays verified the resistance of circFCHO2 to digestion, further confirming its circular nature (Fig. 1I).

Since the function of noncoding RNAs is closely related to their subcellular localization [14, 22], we used fluorescence in situ hybridization (FISH) and nuclear‒cytoplasmic fractionation to assess the localization of circFCHO2 and found that it is predominantly localized in the cytoplasm in BEAS-2B cells (Fig. 1J, K).

We also established a COPD mouse model via cigarette smoke exposure and assessed imaging indicators (thoracic ratio > 1/1.5 and mediastinal shift) of the model by micro computed tomography (Fig. S1D). Tissue FISH revealed that circFCHO2 expression was significantly higher in the lung tissues of COPD patients and mice than in those of normal controls. (Fig. 1L, S1E).

Together, these results show that circFCHO2, as a mammalian conserved and highly expressed circRNA, might play physiological roles in COPD, as supported by data from cell lines, a COPD mouse model and clinical specimens.

### circFCHO2 promotes the EMT of bronchial epithelial cells and ECM remodeling

**Fig. 2:**
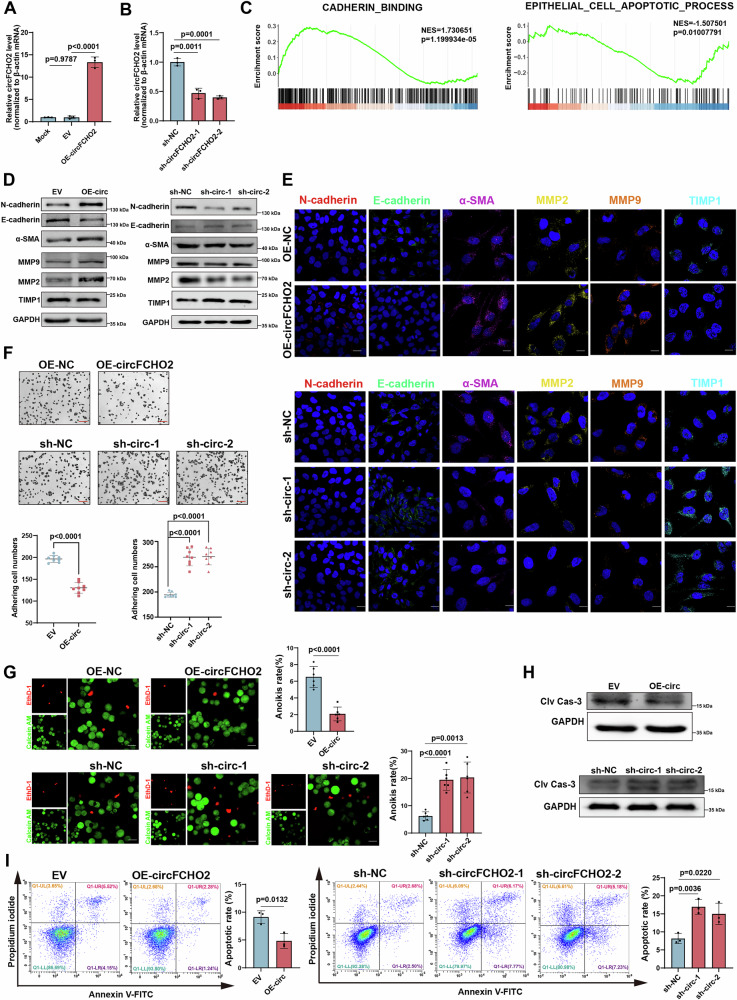
circFCHO2 promotes EMT and ECM remodeling in BEAS-2B cells. **A** Bar plot showing the overexpression (OE) efficiency of circFCHO2 in BEAS-2B cells (*n* = 3). **B** Bar plot indicating the knockdown (KD) efficiency of circFCHO2 in BEAS-2B cells by two independent shRNAs (*n* = 3). **C** Gene set enrichment analysis (GSEA) demonstrated that the DEGs significantly involved in the cell adhesion and apoptosis pathways. **D** The expression levels of EMT- and ECM remodeling -related proteins were examined by western blotting in circFCHO2 OE and KD BEAS-2B cells. **E** IF staining of EMT- and ECM remodeling -related proteins in OE and KD cells. Representative images are shown (DAPI, blue; N-cadherin, red; E-cadherin, green; α-SMA, purple; MMP2, yellow; MMP9, orange;TIMP1, sky blue). Scale bar, 20 μm. **F** Cell adhesion was assessed by crystal violet staining, and the number of adhered OE and KD cells was calculated (*n* = 8). Scale bar, 50 μm. **G** Anoikis was assessed via calcein AM and EthD-1 staining. The ratio of OE and KD cells with anoikis was calculated (*n* = 6). Scale bar, 20 μm. **H** The expression level of Clv-Cas3 in OE and KD cells was estimated by western blotting. **I** The number of apoptotic cells was detected by Annexin V/PI double staining and flow cytometry. The bar plot shows the ratio of apoptotic cells (*n* = 3). For **A**, **B**, **F**, **G** and **I**, *P* values from two-tailed unpaired Student’s *t* test, Welch’s *t*-test and one-way ANOVA are shown. The data are shown as the means ± SDs from independent experiments.

To investigate the function of circFCHO2 associated with the progression of COPD in a lung epithelial cell line, we established BEAS-2B cell lines with stable overexpression (OE) or knockdown (KD) of circFCHO2 using lentiviral vectors (Fig. 2A, B). RNA sequencing (RNA-seq) of these cell lines was performed, and gene set enrichment analysis (GSEA) revealed differentially expressed genes significantly involved in the cell adhesion and apoptosis pathways, which are highly correlated with epithelial‒mesenchymal transition (EMT) and extracellular matrix (ECM) remodeling (Fig. 2C).

EMT and ECM remodeling (primarily ECM degradation) are the critical mechanisms for airway remodeling in COPD [23–25]. Western blotting and immunohistochemistry (IHC) revealed high EMT levels in both the COPD cell model and patient tissues (characterized by the upregulation of N-cadherin/α-SMA and the downregulation of E-cadherin). Additionally, the downregulation of TIMP1 and the upregulation of MMP2/MMP9 confirmed the occurrence of ECM remodeling (Fig. S2A, B). circFCHO2 OE led to the upregulation of N-cadherin, α-SMA, MMP2, MMP9, and the downregulation of E-cadherin/MMP9 in BEAS-2B cells, which promoted EMT and ECM remodeling. Conversely, circFCHO2 KD inhibited EMT and weakened ECM remodeling (Fig. 2D, E; S2C). Epithelial cells undergoing EMT lose their ability to adhere to neighboring cells and the surrounding ECM [26, 27]. We found that circFCHO2 OE reduced cell adhesion to the ECM, whereas circFCHO2 KD increased adhesion (Fig. 2F; S2D).

EMT and ECM remodeling are often accompanied by resistance to anoikis [28, 29]. We assessed the level of anoikis and found that circFCHO2 OE reduced the level of anoikis, whereas circFCHO2 KD promoted anoikis (Fig. 2G). circFCHO2 OE downregulated the expression of Clv-Cas3 and reduced the number of apoptotic cells, whereas knockdown of circFCHO2 resulted in the opposite effects (Fig. 2H, I). Additionally, TUNEL staining confirmed that circFCHO2 OE significantly decreased DNA damage in cells, and circFCHO2 KD had the opposite effect (Fig. S2E).

Overall, circFCHO2 promotes EMT and ECM remodeling, and inhibits anoikis in bronchial epithelial cells, suggesting that circFCHO2 might accelerate the progression of COPD.

### circFCHO2 interacts with PTBP1 and regulates its nucleocytoplasmic distribution

We set out to explore the potential functional mechanism of circFCHO2. RNA immunoprecipitation (RIP) of Ago2, a mediator of circRNA‒miRNA interactions [30], revealed no enrichment of circFCHO2 in BEAS-2B cells, suggesting that circFCHO2 does not function as a miRNA sponge (Fig. S3C). We also predicted that circFCHO2 lacked an open reading frame (ORF) and internal ribosome entry site (IRES) by circRNA Db, indicating that it does not encode a protein (Fig. S3D). These findings suggest that circFCHO2 does not exert its function through miRNA sponging or by generating peptides, but rather binds to RNA-binding proteins (RBPs).

**Fig. 3:**
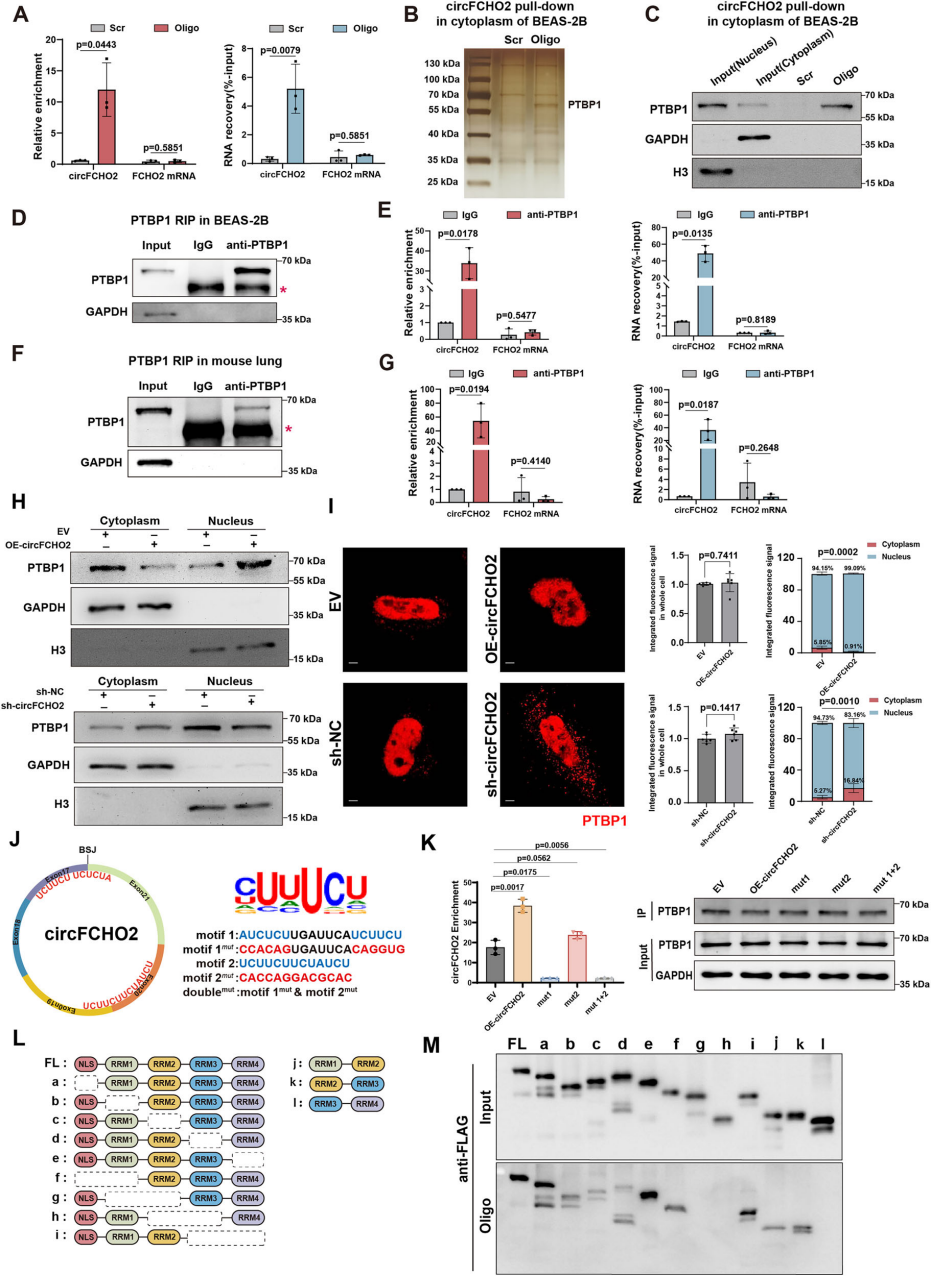
circFCHO2 interacts with PTBP1 and regulates its nucleocytoplasmic distribution. **A** Bar plots showing the pull-down efficiency of circFCHO2 in the cytoplasmic fraction of BEAS-2B cells by RT‒qPCR (*n* = 3). Scr, negative control of biotin-labeled oligo with scrambled sequences; Oligo, biotin-labeled oligo with antisense sequences to the circFCHO2 BSJ. **B** Silver staining revealed that the proteins copulled down with circFCHO2. **C** Western blot analysis demonstrated that PTBP1 copulldown with circFCHO2 in the cytoplasmic fraction of BEAS-2B cells. GAPDH was used as a negative control for cytoplasmic protein. Histone3 is a negative control of the nucleus. **D** RIP with an antibody against PTBP1 was performed in the cytoplasmic fraction of BEAS-2B cells. The RIP efficiency of the PTBP1 protein was validated through western blotting. The red asterisk denotes the heavy chain of the antibody. **E** Bar plots showing PTBP1-RIP enrichment of circFCHO2 in BEAS-2B cells by RT‒qPCR (*n* = 3). **F** RIP with an antibody against PTBP1 was performed in the lung tissue of a mouse. The RIP efficiency of the PTBP1 protein was validated through western blotting. The red asterisk denotes the heavy chain of the antibody. **G** Bar plots showing PTBP1-RIP enrichment of circFCHO2 in the mouse lung by RT‒qPCR (*n* = 3). **H** Western blots showing PTBP1 protein levels in the cytoplasm and nucleus of circFCHO2 OE and KD BEAS-2B cells by nucleocytoplasmic separation. **I** Immunofluorescence (IF) staining of PTBP1 (red) in OE and KD cells. Quantification of whole cell PTBP1 signals (left) and nuclear (Nuc)/cytoplasmic (Cyto) PTBP1 signals (right) are shown as bar plots (*n* = 6). Scale bar, 10 μm. **J** Conserved binding motifs of PTBP1 in circFCHO2. Conserved motif 1 and motif 2 are mutated separately to motif 1^mut^ and motif 2^mut^, or are mutated together as mut1 + 2. Mutation sites from 46-59 nt and 272-279 nt from the BSJ of human circFCHO2. **K** Western blot images indicating successful IP of PTBP1. Association of circFCHO2 examined by RT‒qPCR of PTBP1 RIP in BEAS-2B cells overexpressing the corresponding forms of circFCHO2 (*n* = 3). Enrichment, was normalized to that of IgG. EV, empty vector. **L** Schematic diagram of full-length PTBP1 and various truncated forms of PTBP1. **M** Full-length and truncated forms of PTBP1 were examined by western blotting with an anti-FLAG antibody. For A, **E** and **G**, *P* values from Welch’s *t*-test are shown. For **I**, **M**, *P* values were calculated via one-way ANOVA. The data are shown as the means ± SDs from independent experiments.

Next, to identify potential RBPs of circFCHO2 in human bronchial epithelial cells, we performed circFCHO2 RNA pulldown in the cytoplasm of BEAS-2B cells, because the majority of circFCHO2 is located in the cytoplasm. RT‒qPCR revealed that the oligo could effectively and specifically capture circFCHO2 (Fig. 3A). The circFCHO2 interacting proteins were then separated by SDS‒PAGE, followed by silver staining and mass spectrometry (MS) analysis, revealing the specific circFCHO2 binding band. PTBP1,a well-known RNA splicing factor [31–33], was identified as a circFCHO2-interacting protein in BEAS-2B cells (Fig. 3B; S3A, B). RNA pull-down of circFCHO2 and PTBP1 RIP assays using the cytoplasmic fraction of BEAS-2B cells further verified their interaction (Fig. 3C–E). This interaction was also validated in mouse lung tissue by PTBP1 RIP, indicating that the interaction between circFCHO2 and PTBP1 is conserved in humans and mice (Fig. 3F, G).

We further elucidated the regulatory function of circFCHO2 in PTBP1. The RNA and protein expression levels of PTBP1 were examined in circFCHO2 OE and KD BEAS-2B cells. There were no significant differences in PTBP1 expression levels between circFCHO2 OE/KD cells and control cells, indicating that circFCHO2 does not affect the generation or degradation of PTBP1 (Fig. S3E, F). Next, nuclear‒cytoplasmic fractionation was performed in BEAS-2B cells, and the distribution of PTBP1 in the nucleus and cytoplasm was analyzed by western blotting. Overexpression of circFCHO2 was found to promote PTBP1 import into the nucleus. Conversely, knockdown of circFCHO2 inhibited PTBP1 nuclear translocation (Fig. 3H). Immunofluorescence assays of PTBP1 also revealed that circFCHO2 OE promoted PTBP1 nuclear localization, whereas knockdown of circFCHO2 led to increased PTBP1 retention in the cytoplasm (Fig. 3I). This evidence indicated that circFCHO2 might regulate PTBP1 nucleocytoplasmic distribution.

We then investigated the mechanism by which circFCHO2 binds to PTBP1. Using RBPmap, a tool for predicting RBP binding motifs, we identified two conserved AU-rich motifs in the sequence of circFCHO2 (located at positions 46-59 nt and 272-279 nt from the human circFCHO2 BSJ, named motif 1 and motif 2), which are consistent with the binding preference of PTBP1 (Fig. 3J). We transfected artificially constructed plasmids expressing circFCHO2 with mutations in either motif 1 or motif 2 into BEAS-2B cells. We observed that mutation of motif 1 abolished the interaction between circFCHO2 and PTBP1, whereas mutation of motif 2 did not reduce the binding level between circFCHO2 and PTBP1, indicating that motif 1 is the dominant motif that mediates the interaction between circFCHO2 and PTBP1 (Fig. 3K). PTBP1 contains a nuclear localization signal (NLS) and four tandem RNA recognition motifs (RRMs) in its structure [34, 35]. To determine the interaction domain of PTBP1 with circFCHO2, we overexpressed the full-length and truncated forms of PTBP1 in BEAS-2B cells (Fig. 3L). circFCHO2 pulldown assays revealed that RRM1, RRM2, and RRM3 are all interacting domains of circFCHO2. Among them, RRM2 appears to play the most critical role in mediating the interaction between PTBP1 and circFCHO2 (Fig. 3M). Previous studies have shown that the nuclear‒cytoplasmic shuttling of PTBP1 requires at least the NLS and RRM1 and RRM2, with RRM2 being essential for this process [36]. These findings suggest that the nuclear‒cytoplasmic shuttling of PTBP1 is regulated by circFCHO2 to a certain extent.

These results indicated that circFCHO2 could bind to the splicing factor PTBP1 and promote its import into the nucleus. circFCHO2 AU-rich motifs could interact with the RRM1 and RRM2 domains of PTBP1, which regulate PTBP1 nuclear import.

### circFCHO2 inhibits PTBP1-mediated GRN mRNA precursor splicing

**Fig. 4:**
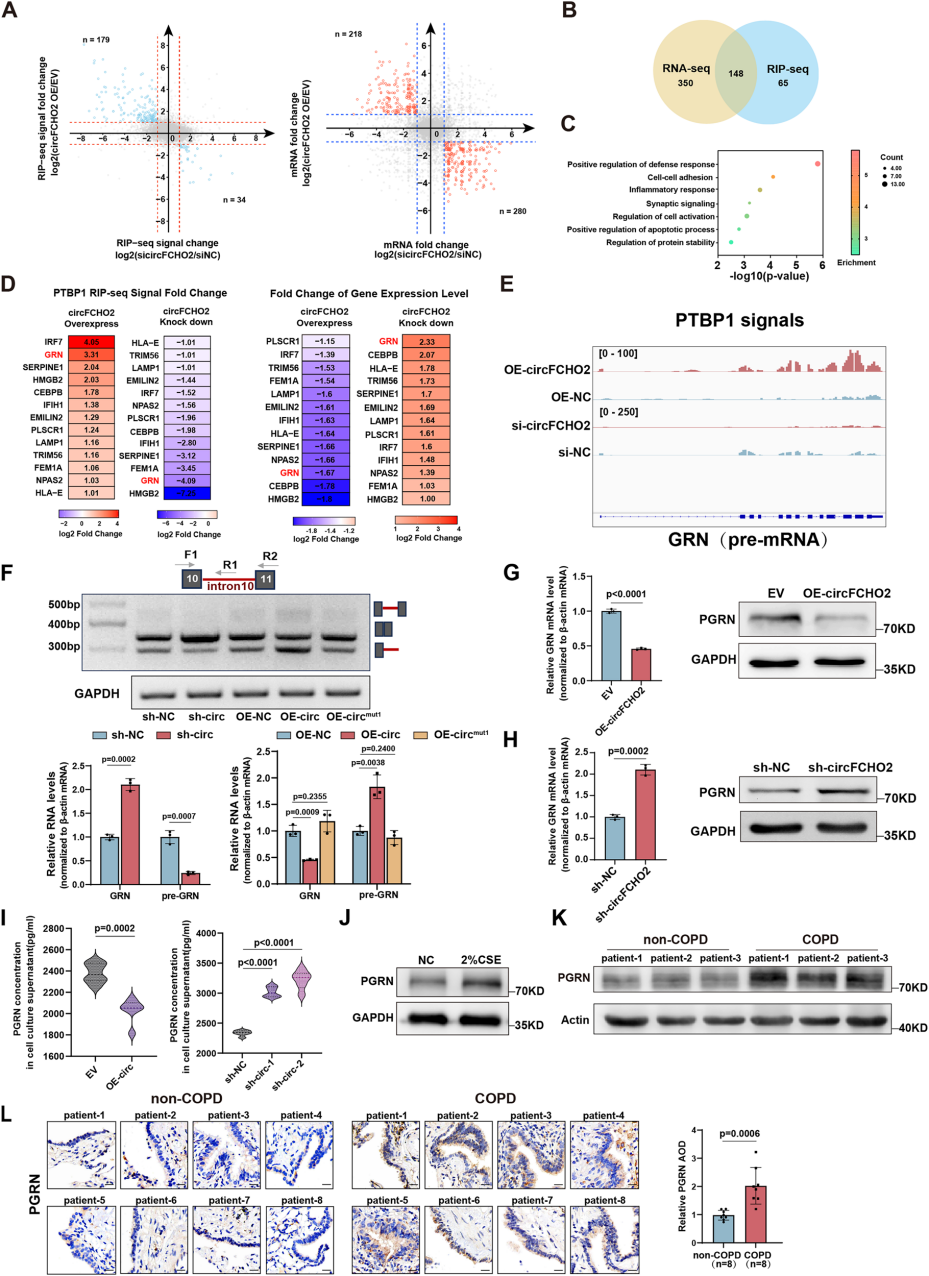
circFCHO2 inhibits PTBP1-mediated GRN mRNA precursor splicing. **A** RIP-seq indicated that 213 mRNAs presented opposite changes in PTBP1 binding levels under circFHCO2 KD and OE conditions. RNA-seq revealed that 498 mRNAs presented opposite changes in expression levels. **B** Venn diagram showing 148 mRNAs whose expression levels and PTBP1 binding levels were both regulated by circFCHO2, which could be considered downstream target genes of the interaction between circFCHO2 and PTBP1. **C** Gene Ontology (GO) analysis revealed that 148 downstream target genes were enriched in biological processes such as positive regulation of the defense response, cell adhesion, and the inflammatory response. The positive regulation of the defense response was the most significantly enriched (lowest *p* value). **D** Heatmap indicating the PTBP1 binding level and expression level of 13 downstream target genes enriched in this biological process, and GRN mRNAs were the genes most significantly affected by circFCHO2 OE and KD among these 13 genes. **E** IGV visualization of the PTBP1 RIP signal on GRN pre-mRNA in circFCHO2 OE and KD BEAS-2B cells. **F** The levels of GRN pre-mRNA and mature GRN mRNA were quantified via RT‒PCR. The RT‒qPCR results are presented as bar plots.(*n* = 3) (**G**) circFCHO2 OE reduced the expression of GRN mRNA and PGRN (*n* = 3). **H** circFCHO2 KD increased the levels of GRN mRNA and its translated protein, progranulin (PGRN). (*n* = 3) (**I**) Violin plot showed that circFCHO2 OE significantly decreased PGRN secretion, whereas circFCHO2 KD increased of PGRN secretion in BEAS-2B cells by enzyme linked immunosorbent assays (ELISA).(*n* = 6) (**J**) PGRN protein levels were increased in the BEAS-2B cell model treated with 2% CSE. (**K**, **L**) Western blot (*n* = 3) and immunohistochemistry (IHC)(*n* = 8) confirmed that PGRN protein levels were increased in COPD patients. Scale bar, 63 μm. For **F**, **G** and **H**, *P* values from two-tailed unpaired Student’s t test are shown. For I, and L, *P* values were calculated via Welch’s *t*-test. The data are shown as the means ± SDs from independent experiments.

To explore the downstream molecular mechanisms by which circFCHO2 and PTBP1 interact in human bronchial epithelial cells. The RNA obtained from PTBP1 RIP in circFCHO2 OE and circFCHO2 KD BEAS-2B cells was subjected to RNA sequencing (RIP-seq). RIP-seq analysis indicated that, compared with their respective controls, 213 mRNAs presented opposite changes in PTBP1 binding levels under circFHCO2 KD and OE conditions, which suggested that the interaction between these mRNAs and PTBP1 was regulated by circFCHO2. Moreover, a comparison of the RNA-seq results (indicated in the second part of the results) from circFCHO2 OE and KD cells revealed that 498 mRNAs presented opposite changes in expression levels, which indicated that the expression levels of these genes might be affected by circFCHO2 (Fig. 4A). We intersected these 213 mRNAs from RIP-seq and 498 mRNAs from RNA-seq, resulting in 148 mRNAs whose expression levels and PTBP1 binding levels are both regulated by circFCHO2, which could be considered downstream target genes of the interaction between circFCHO2 and PTBP1 (Fig. 4B). Gene Ontology (GO) analysis revealed that these 148 genes were enriched in biological processes such as positive regulation of the defense response, cell adhesion, and the inflammatory response (Fig. 4C).

Among these biological processes, positive regulation of the defense response was the most significantly enriched (lowest p value). We found that 13 downstream target genes were enriched in this biological process. Among these 13 genes, the PTBP1 binding level and expression level of GRN mRNA were most significantly affected by circFCHO2 OE and KD (Fig. 4D). Overexpression of circFCHO2 significantly increased the PTBP1 binding level on GRN pre-mRNA (with a higher binding level at the 3’ end than at the 5’ end), whereas circFCHO2 KD reduced the PTBP1 binding level (Fig. 4E). Therefore, we selected GRN as a downstream target gene for further investigation.

We previously demonstrated that circFCHO2 can promote the nuclear import of the splicing factor PTBP1, suggesting that circFCHO2 might regulate the splicing of GRN pre-mRNA through PTBP1, thereby regulating GRN expression. To test this hypothesis, we measured the ratio of pre-mRNA to mature mRNA from the GRN 10-11 exon (the region with the highest PTBP1 binding level) via RT‒PCR with specific primers. We observed that knockdown of circFCHO2 significantly increased mature GRN mRNA levels, whereas overexpression led to a significant increase in GRN pre-mRNA levels. Additionally, mutation of motif 1 did not significantly affect the precursor or mature GRN mRNA levels compared with those in the control. These data collectively demonstrate the critical role of circFCHO2-PTBP1 in modulating GRN RNA processing (Fig. 4F). Additionally, circFCHO2 KD increased the levels of GRN mRNA and its translated protein, progranulin (PGRN). In contrast, circFCHO2 OE reduced the expression of GRN mRNA and PGRN (Fig. 4G, H). PGRN is a multifunctional glycoprotein expressed in various tissues. As a secreted protein, it can bind to cell membrane receptors to perform its biological functions [37]. Therefore, we also quantified PGRN secretion levels in the cell culture medium using enzyme-linked immunosorbent assays (ELISAs). We found that circFCHO2 overexpression significantly decreased PGRN secretion, whereas insufficient circFCHO2 led to a marked increase in PGRN secretion in BEAS-2B cells (Fig. 4I).

In addition, RT‒qPCR, western blot, and IHC revealed that PGRN mRNA and protein levels were increased in both the COPD cell model (2% CSE treatment) and patient samples, suggesting a close association between PGRN and COPD (Fig. 4J–L; S4A).

These results demonstrate that circFCHO2 could interact with the splicing factor PTBP1 to promote its nuclear entry and binding to the GRN pre-mRNA, which inhibited the splicing of GRN pre-mRNA, leading to a downregulation of PGRN protein expression and secretion in bronchial epithelial cells.

### circFCHO2 promotes EMT and ECM remodeling by decreasing PGRN levels and activating the NF-κB signaling pathway

**Fig. 5:**
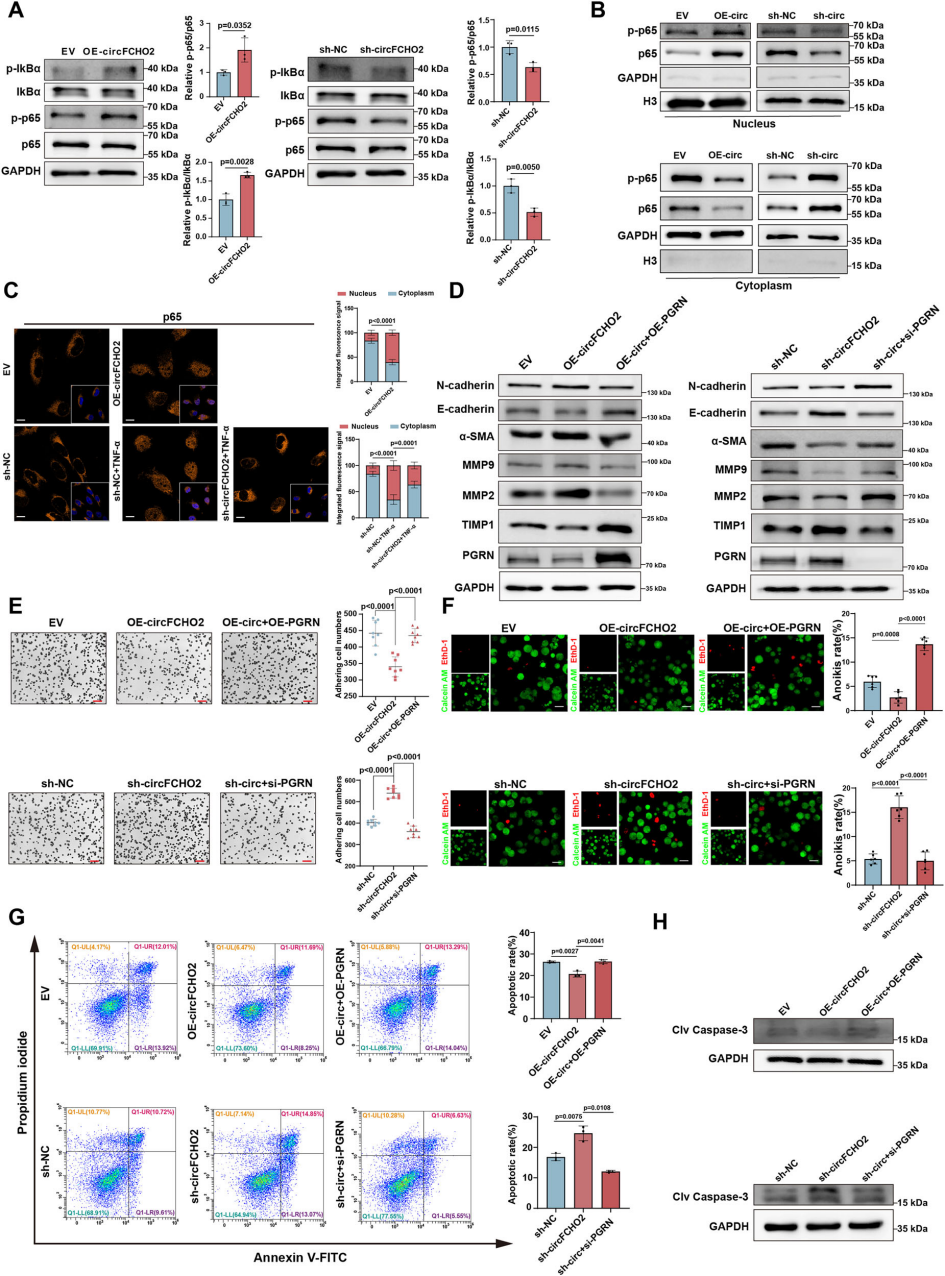
circFCHO2 promotes EMT and ECM remodeling by decreasing PGRN levels and activating the NF-κB signaling pathway. **A** The relative expression levels of p-IκBα/ IκBα and p-p65/p65 in circFCHO2 OE and KD BEAS-2B cells were examined by western blotting. Bar plots showing the relative levels of p-IκBα/ IκBα and p-p65/p65 (*n* = 3). **B** The levels of p65 and p-p65 in the nuclei of circFCHO2 OE and KD BEAS-2B cells. **C** Immunofluorescence (IF) staining of p65 (orange) in OE and KD cells. The final concentration of TNF-α used for cell treatment was 20 ng/mL. The quantification of nuclear (Nuc)/cytoplasmic (Cyto) p65 signals is shown as bar plots (*n* = 6). Scale bar, 20 μm. **D** The expression levels of EMT- and ECM remodeling -related proteins were examined by western blotting in circFCHO2 OE and KD cells with PGRN OE and KD. **E** Cell adhesion was assessed by crystal violet staining, and the number of adhering cells was calculated in circFCHO2 OE and KD cells with PGRN OE and KD (*n* = 8). Scale bar, 50 μm. **F** The level of anoikis was assessed by calcein AM and EthD-1 staining. The ratio of cells with anoikis was calculated in circFCHO2 OE and KD cells with PGRN OE and KD (*n* = 6). Scale bar, 20 μm.**G** The number of apoptotic OE and KD cells with PGRN OE and KD was detected by Annexin V/PI double staining and flow cytometry. The bar plots show the ratio of apoptotic cells (*n* = 3). **H** The expression level of Clv-Cas3 in OE and KD cells with PGRN OE and KD was estimated by western blotting. For A, *P* values from two-tailed unpaired Student’s *t*test. For **C**, **E**, **F** and **G**, *P* values were calculated via one-way ANOVA. The data are shown as the means ± SDs from independent experiments.

PGRN can inhibit the activation of the NF-κB signaling pathway by binding to the tumor necrosis factor receptor (TNFR) and blocking the TNFα/TNFR interaction [38, 39]. We found that the levels of key proteins in the NF-κB signaling pathway, such as phosphorylated IκBα (p-IκBα) and phosphorylated p65 (p-p65), were decreased in PGRN OE cells, whereas PGRN knockdown increased their phosphorylation levels (Fig. S4B–G). Furthermore, we demonstrated that PGRN OE inhibited the nuclear import of p65 and p-p65. In contrast, PGRN KD promoted the nuclear import of both p65 and p-p65 (Fig. S4H, I). These results demonstrate that PGRN suppresses NF-κB signaling activation in BEAS-2B cells. Accordingly, NF-κB pathway activity was examined in circFCHO2- overexpressing or circFCHO2-knockdown cells, revealing that circFCHO2 promotes NF-κB signaling activation and that knockdown of circFCHO2 inhibits TNF-α-induced nuclear translocation of p65/p-p65, whereas PGRN reversed the circFCHO2-induced activation of NF-κB signaling (Fig. 5A-C; S4J–N). The above results demonstrate that circFCHO2 regulates the activity of the canonical NF-κB pathway through PGRN.

The NF-κB signaling pathway is one of the key pathways involved in EMT and ECM remodeling, and the activation of the NF-κB signaling pathway can induce EMT and the expression of matrix metalloproteinases (MMPs) [40, 41]. These findings suggest that circFCHO2 may activate the NF-κB pathway by downregulating PGRN protein expression and secretion, thereby promoting EMT and ECM remodeling. To test this hypothesis, we overexpressed PGRN in circFCHO2 OE cells and assessed the level of EMT and ECM remodeling. We found that PGRN overexpression significantly reversed the EMT and ECM remodeling induced by circFCHO2 OE. In contrast, PGRN knockdown in circFCHO2 KD cells enhanced EMT and ECM remodeling (Fig. 5D). With respect to other phenotypes of EMT and ECM remodeling, PGRN overexpression in circFCHO2 OE cells also restored the cell-ECM adhesion capacity. Conversely, PGRN knockdown in circFCHO2 KD cells disrupted cell-ECM adhesion (Fig. 5E; S4O). PGRN overexpression in circFCHO2 OE cells decreased their resistance to anoikis, whereas PGRN knockdown increased their resistance to anoikis (Fig. 5F). Furthermore, PGRN overexpression in circFCHO2 OE cells promoted apoptosis, whereas PGRN knockdown reversed this effect (Fig. 5G, H).

Taken together, these results suggest that circFCHO2 can suppress the expression of PGRN, thereby activating the NF-κB signaling pathway and promoting EMT and ECM remodeling.

### circFCHO2 knockdown reverses cigarette smoke-induced emphysema and airway remodeling in COPD mice

**Fig. 6:**
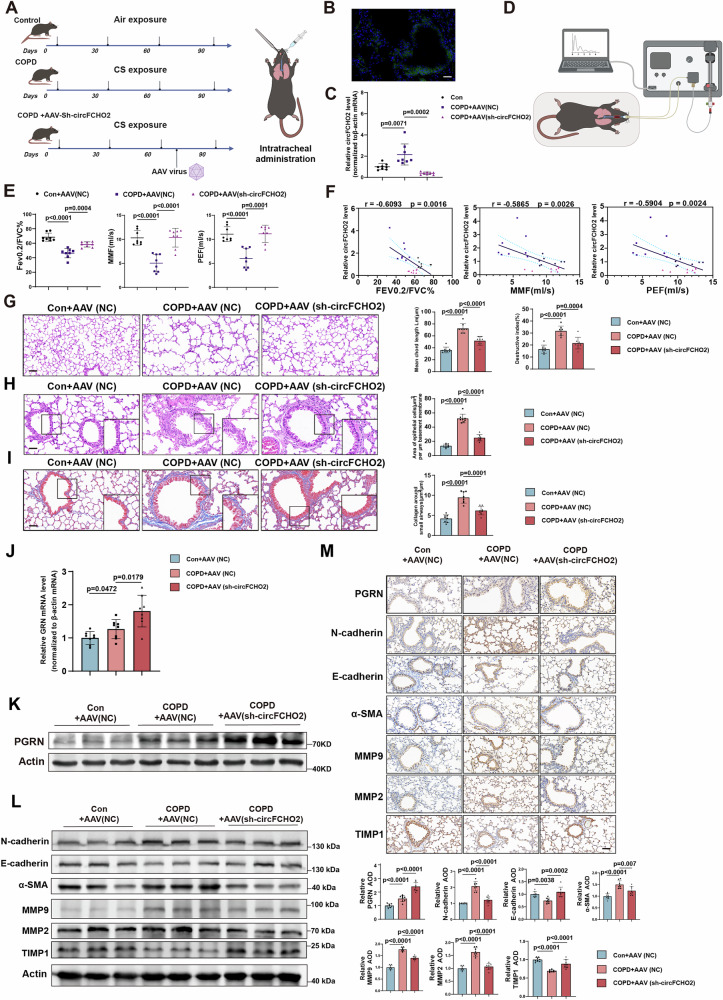
circFCHO2 knockdown reverses cigarette smoke-induced emphysema and airway remodeling in COPD mice. **A** Schematic chart of male C57BL/6 J mice exposed to air or to CS (300 mg/m^3^ TPM) with or without AAV-circFCHO2 shRNA treatment. **B** The GFP fluorescence signal shows the AAV infection efficiency in the mouse lung. Scale bar, 50 μm. **C** The levels of circFCHO2 in the lung tissues of the mice were determined via RT‒qPCR (*n* = 8). **D** Schematic chart of the lung function test in mice. **E** Lung function parameters were examined in COPD mice infected with AAV (*n* = 8). Fev0.2/FVC%, 200 ms/forced vital capacity percentage; MMF, mean flow rate from 25%-75% of the FVC; PEF, peak expiratory flow. **F** There was significant negative correlation between the relative circFCHO2 level and the lung function level (*n* = 8) (**G**) Mouse lung sections were stained with hematoxylin and eosin (H&E) (scale bar, 100 μm) and emphysema-like alveolar enlargement was quantified on the basis of the mean linear intercept and destructive index (*n* = 8). The bar plots show the mean chord length and destructive index in each group. **H** Mouse lung sections were stained with H&E (scale bar: 50 μm; inserts show expanded images of the indicated region). The quantification of epithelial thickness in the small airways was normalized to the perimeter of the basement membrane, as shown in the bar plot (*n* = 8). **I** Collagen deposition around small airways in lung sections was assessed using Masson’s trichrome histochemistry (blue color indicates collagen, scale bar: 50 μm; inserts show expanded images of the indicated region). The quantified collagen area around the small airways was normalized to the perimeter of the basement membrane (*n* = 8). **J** Bar plot showing the relative GRN mRNA levels in mouse lung tissues (*n* = 8). **K** Western blot analysis of PGRN protein levels in mouse lung tissues. **L** Western blots showing EMT- and ECM remodeling -related proteins in mouse lung tissues. **M** IHC staining in mouse lung tissues (*n* = 8). Scale bar 50 μm. Bar plot showing the relative protein expression levels in mouse lung tissues (*n* = 8). For **F**, Pearson’s correlations were used. For **C**, **E**, **G**, **H**, **I**, **J** and **L**, *P* values were calculated via one-way ANOVA. The data are shown as the means ± SDs from independent experiments.

circFCHO2 is a conserved circRNA in both humans and mice. To investigate the physiological function of circFCHO2, we administered AAV-circFCHO2-shRNA-GFP via bronchial injection to reduce the expression of circFCHO2 in the lungs of the established COPD mouse model (Fig. 6A, B). We confirmed the successful knockdown of circFCHO2 in the lungs of COPD model mice via RT‒qPCR following AAV injection (Fig. 6C).

Next, we assessed the changes in lung function and lung tissue histology in a COPD mouse model with circFCHO2 KD. We found that lung function parameters, including forced expiratory volume at 200 ms/forced vital capacity percentage (Fev0.2/FVC%), mean flow rate from 25%-75% of the FVC (MMF), and peak expiratory flow (PEF), were lower in the CS-treated group than in the control group. However,AAV-circFCHO2 treatment reversed these effects (Fig. 6D, E). We also observed a significant negative correlation between circFCHO2 expression levels and lung function, suggesting that low circFCHO2 levels partially reverse lung function in the COPD mouse model (Fig. 6F). Histopathological analysis of the lungs and airways via hematoxylin and eosin (H&E) staining revealed that circFCHO2 knockdown could partially restore the alveolar septal disruption and thickening of small airway epithelial areas induced by cigarette smoke in a COPD mouse model (Fig. 6G, H). We observed that collagen deposition around the small airways was also reduced following circFCHO2 KD by Masson staining and IHC staining (Fig. 6I; S5A, B). These results indicate that the downregulation of circFCHO2 can partially restore airway remodeling and emphysema in a COPD mouse model.

Consistent with the results obtained from the BEAS-2B cells, the GRN mRNA and protein expression levels were increased in the bronchial epithelial cells of the COPD model mice with circFCHO2 KD (Fig. 6J, K). Additionally, EMT and ECM remodeling were alleviated in the circFCHO2 KD COPD mouse model (Fig. 6L, M).

Collectively, these data confirmed that, circFCHO2 knockdown reversed cigarette smoke-induced COPD symptoms in mice, such as emphysema and airway remodeling by upregulating PGRN expression. These findings suggest that circFCHO2 might play an important pathological role in the progression of COPD.

## Discussion

Chronic obstructive pulmonary disease (COPD) is a chronic inflammatory lung disease resulting from the interplay between genetic and environmental factors. Its primary clinical manifestation is persistent airflow limitation that is largely irreversible. Airway remodeling and structural damage to lung tissue are key pathological features of COPD [1]. Currently, there are no clinically effective therapies capable of fully reversing the pathological changes associated with COPD. Therefore, gaining a deeper understanding of airway remodeling and structural damage in COPD is crucial for the development of novel pharmacological treatments. In this study, we identified circFCHO2 as a conserved circular RNA (circRNA) shared between humans and mice, with significantly higher expression levels in COPD tissues than in control tissues. Both in vivo and in vitro experiments demonstrated that circFCHO2 promotes epithelial‒mesenchymal transition (EMT) and extracellular matrix (ECM) remodeling, thereby exacerbating airway remodeling in COPD.

EMT is a complex biological process that plays a critical role in both normal physiological and pathological conditions. In addition to changes in cell adhesion, cells undergoing EMT exhibit reduced sensitivity to apoptotic signals, manifesting as increased antiapoptotic capacity [42, 43]. Tumor cells undergoing EMT often display increased resistance to anoikis, leading to the emergence of invasive and metastatic malignant tumors [44]. In normal tissues, however, anoikis prevents cells from surviving in inappropriate environments, thereby inhibiting potential abnormal proliferation and metastasis. Our study revealed that circFCHO2 not only promotes EMT in bronchial epithelial cells but also increases resistance to anoikis. However, under cigarette smoke extract (CSE) treatment, bronchial epithelial cells undergoing EMT exhibit increased apoptosis [45, 46]. Similarly, studies have shown that cigarette smoke may induce pyroptosis via the NLRP3 inflammasome and caspase-1 [47]. Reactive oxygen species (ROS) and inducible nitric oxide synthase (iNOS) in cigarette smoke can also trigger ferroptosis in lung epithelial cells by increasing intracellular iron accumulation and promoting oxidative stress [48]. These findings suggest that the molecular mechanisms underlying CSE-induced cell death are highly complex and require further investigation.

Progranulin (PGRN) is a multifunctional protein that has been implicated in various diseases, where its role may be dual, either protective or detrimental, depending on the context. Its functions in neurodegenerative diseases, particularly frontotemporal dementia (FTD) and Alzheimer’s disease (AD),have been extensively studied. As a neuroprotective factor, PGRN deficiency exacerbates neurodegenerative processes [49, 50]. In contrast, PGRN often promotes tumor growth, metastasis, and immune evasion in cancer. For example, tumor-derived PGRN in breast cancer recruits M2 macrophages, whose immunosuppressive cytokines inhibit T-cell function and metabolism, thereby facilitating tumor immune evasion [51]. Additionally, PGRN overexpression enhances the antiapoptotic capacity of myeloma cells and contributes to dexamethasone resistance [52]. In autoimmune diseases, PGRN plays dual physiological and pathological roles. On the one hand, as a unique ligand for TNFR (TNFR1 and TNFR2), it suppresses inflammatory cascades and inhibits Th1 and Th17 helper T-cell activity, alleviating conditions such as osteoarthritis, autoimmune uveitis (EAU), and experimental autoimmune encephalomyelitis (EAE) [39, 53]. On the other hand, in systemic lupus erythematosus (SLE), GRN (a degradation product of PGRN) may exacerbate immune responses and disease activity through its influence on TLR9 signaling. Given its diverse functions [54–56], PGRN has potential as a therapeutic target in various diseases. In our study, PGRN expression was upregulated in COPD cell models, patient samples, and mouse lung tissues. The overexpression of PGRN alleviated EMT and ECM remodeling, suggesting that PGRN may act as a protective factor to mitigate airway structural abnormalities during COPD progression. Notably, circFCHO2, which is listed in the circBase database, was detected in 12 samples, including cancer cells and various tissues [57–60]. Its regulatory effects on PGRN highlight its importance beyond COPD.

Our study revealed that circFCHO2 can activate the NF-κB signaling pathway by reducing the secretion of the PGRN protein. As a central pathway in cellular stress and immune responses, the NF-κB signaling pathway regulates target genes, including inflammatory cytokines, immune modulators, cell adhesion molecules, and oxidative stress-related genes [61, 62]. Studies have shown that NF-κB can directly bind to the promoter region of MMP-9 and promote its expression [63]. In COPD, activation of the NF-κB pathway enhances the secretion of MMP-9 by pulmonary epithelial cells, leading to the degradation of alveolar elastic fibers and basement membranes, and ultimately contributing to emphysema [64]. Additionally, NF-κB activation induces the expression of the EMT transcription factor ZEB1 and, in synergy with TGF-β, accelerates the progression of EMT [40, 65]. We cannot rule out the possibility that circFCHO2, through NF-κB activation, regulates the release of inflammatory cytokines by airway epithelial cells. In chronic airway inflammatory diseases, the immune microenvironment promotes the secretion of chemokines and cytokines by epithelial cells, further driving EMT. EMT, in turn, compromises the barrier function of the airway epithelium. Disruption of this epithelial barrier facilitates the penetration of inflammatory cytokines into deeper layers of the airway, exacerbating inflammation and fibrosis [20, 66, 67].

In summary, this study reveals that circFCHO2 is a pathogenic driver of COPD. In COPD patients and experimental COPD models, circFCHO2 binds to PTBP1 and promotes its nuclear translocation, reducing the splicing of GRN pre-mRNA into mature mRNA, which further decreases the translation of GRN mRNA into PGRN protein. The reduced expression and secretion of PGRN protein activate the NF-κB pathway, inducing EMT and ECM remodeling in bronchial epithelial cells, leading to airway remodeling and emphysema, and ultimately triggering COPD. This study highlights that circFCHO2 and PGRN are key regulators of EMT and ECM remodeling, and that circFCHO2-dependent EMT and ECM remodeling represent potential therapeutic targets for COPD.

## Methods and materials

### Clinical specimens

Lung tissue samples were obtained from 16 patients with operable, non-malignant pulmonary nodules recruited from the Second Affiliated Hospital of Anhui Medical University. Written informed consent was obtained from each patient for this study.

### Cell culture

The human normal lung epithelial BEAS-2B cells were purchased from Boster Biological Technology(Wuhan,China)and cultured in DMEM(Invitrogen, Gibco). Cells were maintained under standard culture conditions with 10%FBS (CLARK, FB25015) and 1% penicillin/streptomycin (Beyotime,C0222) at 37 °C and 5% CO_2_.

### Preparation of cigarette smoke extract(CSE) and CS exposure

Cigarette smoke extract (CSE) was prepared as previously described with minor modifications [68]. The research reference cigarettes 3R4F were used for experiments (Louisville, KY, USA). Briefly,4 mL of serum-free sterile DMEM medium was drawn into a 60-mL syringe. Subsequently,forty milliliters of cigarette smoke were drawn into the syringe and mixed with the medium by vigorous shaking for 30 s, nine cigarettes were used for each 4 mL of medium. The CSE solution, filtered through an aseptic 0.22 μm filter, was considered as 100%. CSE quality was accepted if ΔOD (A320-A540) was between 0.9 and 1.2 and the resulting solution must be used in 30 min. Wild-type C57BL/6 J mice were purchased from GemPharmatech (Beijing, China). In a whole-body exposure system (Beijing Huironghe Technology CO., Ltd, China), mice were exposed to CS (300 mg/m^3^ of total particulate matter (TPM)) for 120 min once a day, 5 days a week for 3 months.

### Micro-computed tomography (micro CT)

Micro-CT (PerkinElmer, CA, USA)was performed on mice three days before the conclusion of the study. The mice were sedated by inhalation of isoflurane. The radiography parameters were 45 kV, 250 μA, and 12 ms exposure per projection.

### Lung function test

After the modeling period, the mice were tracheostomized, and the trachea was cannulated. Then, the cannula was connected to a computer-controlled small animal ventilator with a sealed glass cover. After endotracheal intubation, the pulmonary function of the mice was measured using the AniRes2005 (BestLab High-Tech, Beijing,China).

### Knockdown of circFCHO2 in mouse lung tissue

AAV-circFCHO2 shRNA was purchased from OBiO Biotechnology Co. Ltd. (Shanghai, China). On day 70, 50 *μ*l of circFCHO2-Adeno-associated virus(AAV-circFCHO2-shRNA-GFP) was intratracheally administrated (total amount of titer 2 × 10^11^ vg per mouse, once) to the mouse. An equivalent amount of negative control (NC)-AAV diluted with PBS was applied as vehicle control.

### Single-molecule fluorescence in situ hybridization (smFISH)

The smFISH procedure was performed with minor modifications to a previously described method [69]. Cells or tissues were fixed with 4% paraformaldehyde (PFA) for 10 min at room temperature and then permeabilized overnight with ice-cold 70% ethanol at −20 °C. For hybridization, samples were prehybridized at 37 °C for 30 minutes in a hybridization buffer (consisting of 30% formamide, 5× SSC, 9 mM citric acid at pH 6.0, 0.1% Tween-20, 50 μg/mL heparin, 1× Denhardt’s solution, and 10% dextran sulfate) after being washed twice with 2× SSC. Next, the samples were hybridized with 2 pmol probes overnight at 37 °C in the hybridization buffer. For the amplification step, 18 pmol hairpins were heated to 95 °C for 90 s and then allowed to cool to room temperature in the dark for 30 min. Samples were incubated with these denatured probes overnight at room temperature, followed by three 5-minute washes in 2× SSC buffer. After a 10 min incubation in DAPI (5 mg/mL), the sections were washed three times for 5 min each in 2× SSC buffer. Images were acquired using a Zeiss LSM980 laser confocal microscope. ImageJ software was utilized for further data processing, and the ImageJ Plot Profile tool was employed to calculate signal intensity. All probe sequences are listed in Supplementary table 1 of the Supporting Information.

### RNase R treatment

Total RNA (1 μg) was incubated with 4 U/μg of RNase R (Yeasen, 14606) for 20 min at 37 °C. After treatment with RNase R, the RNA expression levels of circFCHO2 and FCHO2 mRNA were analyzed by RT-qPCR.

### Nuclear and cytoplasmic extraction

Cells were first washed twice with PBS and then incubated on ice for 20 min in a hypotonic buffer containing 10 mM Tris-HCl (pH 8.0), 140 mM NaCl, 1.5 mM MgCl_2_, 0.5% NP-40, 1 mM DTT, and 0.1 U/μL RNase inhibitor (Promega, N2615). After centrifugation at 1000 × *g* for 5 min at 4 °C, the supernatant was separated as the cytoplasmic fraction. The remaining pellet was resuspended in a nuclear resuspension buffer comprising 20 mM HEPES (pH 7.9), 400 mM NaCl, 1 mM EGTA, 1 mM EDTA, 1 mM DTT, and 0.1 U/μL RNase inhibitor. This mixture was incubated at 4 °C for 30 minutes. The nuclear fraction was then obtained by removing insoluble membrane debris through centrifugation at 12,000 × *g* for 15 min.

### Small interfering RNA, plasmids, and transfection

BEAS-2B cells were transfected with siRNAs (Supplementary table 1) synthesized by GenePharma (Shanghai), targeted to GRN 48 h using Lipofectamine 3000 (Invitrogen, L3000-015). All plasmids were constructed with restriction enzyme digestion and ligation or with recombinant methods (Vazyme, c113-02). For circFCHO2 overexpression, the circularized exons and the endogenous flanking sequences including one Alu pair were inserted into the pcDNA3 vector. The backbone vector of p3×FLAG was used for constructing FLAG-tagged PTBP1 (Gene synthesis ensured accurate expression of truncated variants. To enhance expression, a Kozak sequence (GCCACC) was incorporated upstream of all targets, followed by ligation into a 3×FLAG-tagged plasmid vector). The shRNA (Supplementary Table 1) against the BSJ of circFCHO2 was cloned into the vector pLKO.1 (Sigma) and the negative-control shRNA (shCtrl, MFCD07785395) was obtained from the MISSION shRNA Library (Sigma). All plasmids have been sequenced for confirmation.

These plasmids were co-transfected with packaging plasmids psPAX2 and pMD2G into 293 T cells. Infectious lentiviruses were harvested at 48 h after transfection, followed with concentration by ultracentrifugation. Stable BEAS-2b cell lines were obtained by the selection with 1 μg/ml puromycin for 2 weeks.

### Cell adhesion assay

The cell adhesion assay kit (Beibo Biotechnology, Shanghai, China) was used to evaluate cell-matrix adhesion. Culture plates were pre-coated by adding 50 μl of coating solution per well in a 96-well plate, sealed with film, and incubated at 2–8 °C overnight. After removing the coating solution, the plates were air-dried in a fume hood until completely dry, washed 1–3 times with washing buffer, and cells were prepared by trypsin digestion, PBS washing, and resuspension in medium. Cells (5 × 10^5/well) were seeded into 96-well plates (for CCK-8) or 48-well plates (for crystal violet staining), with five replicates per group. After 30 min incubation at 37 °C, the medium was aspirated, washed twice with DMEM, and 100 μl fresh medium was added. For CCK-8, 10 μl of Solution B was added per well, incubated for 30 min, and OD450 values were measured. The adhesion rate (%) was calculated as [(ODtest − ODblank) / (ODcontrol − ODblank)] × 100. For crystal violet staining, cells were fixed with 4% paraformaldehyde, stained with 0.1% crystal violet (500 μl/well) for 10 minutes, washed three times with PBS, air-dried, and imaged.

### Apoptosis analysis

Cells were seeded in 6-well plates at a density of 1 × 10^5^ cells per well. Cells were collected and washed twice with ice-cold PBS, resuspended in binding buffer, treated with Annexin V-FITC/PI (BD Biosciences, CA, USA) and incubated in the dark for 15 min. Flow cytometry analysis was performed within 1 h to measure apoptotic rate (BD FACVerse).

Anoikis detection kit was purchased from Biovision (Abcame,ab211153). Anoikis assays were performed as described previously [70]. 2 × 10^5^ cells were cultured in plates coated with poly-HEMA. Calcein AM/ethidium homodimer-1 (EthD-1) solution (500X, 1 μL) was added to each well of a 12-well anchorage resistant plate. The plates were then incubated for 30–60 min at 37 °C. The green calcein AM fluorescence (Ex, 485 nm; Em, 515 nm) and red EthD-1 fluorescence (Ex, 520 nm; Em, 590 nm) were detected by inverted fluorescence microscope. Green calcein AM fluorescence stains live cells, red EthD-1 fluorescence stains dead cells.

Apoptosis was also assessed by transferase-mediated dUTP nick end labeling (TUNEL) assay with an in situ cell death detection kit (Vazyme, A111-02). Briefly, cells were washed after treatment, fixed with 4% formaldehyde in PBS, and permeabilized with 0.1% Triton X-100. Then, the TUNEL assay was performed according to the manufacturer’s instructions. The number of TUNEL-positive cells and DAPI-positive nuclei were counted manually to calculate the percentage of apoptotic cells to total cells.

### PCR reactions

RNA was extracted with TRIzol reagent (Invitrogen) according to the manufacturer’s protocol. For RT-PCR, cDNA was synthesized from RNA with a GoScript Reverse Transcription System (Promega) according to the manufacturer’s protocol. For PCR with template of gDNA, gDNA was isolated with phenol/chloroform extraction. Real-time quantitative PCR (RT-qPCR) was performed with GoTaq SYBR Green qPCR Master Mix (Promega) on a PikoReal 96 real-time PCR system (Thermo Scientific) according to standard procedures. For semi-quantitative RT-PCR gels, 25–30 cycles of PCR were always performed. All PCR products were confirmed by Sanger sequencing. All used primer sequences are included in Supplementary table 1 of the Supporting Information.

### RNA Pull-Down and RNA Immunoprecipitation

BEAS-2b cells and murine lung tissues were cross-linked (a total of 0.4 J /cm^2^) in a UV cross-linker. Cells were harvested in ice-cold lysis buffer (20 mM HEPES, pH 7.4, 10 mM KCl, 2 mM MgCl2, 0.5%NP-40, 1 mM DTT, 1×Protease Inhibitor Cocktail, and 0.1 U/μL RNase inhibitor (Promega, N2615)) for 30 min on ice. The supernatant was collected after centrifugation at 1000 × *g* for 5 min at 4 °C, and subjected to sonication on ice for 5 min with a Sonics Vibra-Cell (3 s on, 6 s off, 30%). 100 pmol biotinylated AS oligos (for pull-down) or 2 μg antibody (for RIP) was added to the supernatant. After rotation 4 h at 4 °C, 50 μL M-280 Streptavidin Dynabeads (Invitrogen, 11206D, for RNA pulldown) or Protein G Dynabeads (Invitrogen, 10004D, for RIP), which were blocked with 500 ng/ μL yeast total RNA and 1 mg/ mL BSA for 1 h at room temperature were added. After rotation 4 h at 4 °C, washing once with lysis buffer, twice with high salt lysis buffer (20 mM HEPES, pH 7.4, 10 mM KCl, 500 Mm NaCl, 2 mM MgCl_2_, 0.5% NP-40, 1 mM DTT, 1× Protease Inhibitor Cocktail, and 0.1 U /μLRNase inhibitor). The purified RNAs were analyzed by RT-qPCR. The purified proteins were analyzed by MS after silver staining or western blot. The following antibodies were used: anti-PTBP1 (protentech, 12582-1-AP); anti-AGO2 (Sigma, SAB4200085).

### Silver staining

Silver staining was performed using the Protein Stains K kit (Sangon Biotech, C500021-0010). Briefly, after the polyacrylamide gel was washed twice with deionized water, 20 mL of fixative solution (composed of 40% ethanol and 10% acetic acid) was added. The gel was fixed on an oscillating shaker for 30 min. Subsequently, 20 mL of sensitizing solution was added, and the gel was shaken for 45 min. The gel was then washed three times with deionized water, and shaken for 5 min per wash. Next, the staining solution was added, and the gel was shaken for 30 min. After the staining solution was decanted, the gel was washed twice with deionized water, and shaken for 30 s each time. Finally, the developing solution was added, and the gel was shaken on a shaker until the protein bands became clearly visible.

### RNA-seq, RIP-seq and the corresponding bioinformatics analyses

The RNAs from RIP sample or whole-cell sample were utilized to construct whole transcriptome library by the TruSeq Ribo Profile Library Prep Kit (Illumina, United States), following manufacturer’s instructions. In brief, rRNA was removed by Illumina Ribo-Zero Gold kit and surplus RNA was purified for end repair and 50-adaptor ligation. Then, reverse transcription was performed with random primers containing 3’ adaptor sequences. Finally, the cDNAs were purified and amplified with PCR reaction. The products with 300–500 length were purified and quantified. These libraries were utilized to 150 nt paired-end sequencing with an Illumina Nova seq 6000 system (Novogene, China). Each library was generated a depth of ~8 G basepairs (for mRNA-seq sample) or ~6 G basepairs (for RIP sample), and then adapters were removed with cutadapt software to obtain cleandata.

To analyze RNA-seq, the high-throughput sequencing tools, Hisat2, and feature_counts, were used to map clean reads to Homo sapiens reference genome (hg19) and calculate the gene expression level. The differentially expressed gene (DEG) was determined by the “DEseq2” package in R software using the corresponding cutoff (q < 0.05, |log2(fold change)|> 1. These DEGs were pre-ranked and the GSEA analysis was implemented in GSEA software (version 4.1.0, https://www.gsea-msigdb.org).

Reads from RIP-seq cleaned data were aligned to the hg19 genome with Bowtie2 version 2.4.1 using default settings, with all duplicates, unmapped reads, reads with more than three mismatches, and non-uniquely mapped reads were removed by samtools version 1.1. The bamCoverage pipeline in deeptools (v 3.5.1) was used to generate PTBP1 binding reads coverage files (.bw). The multiBigwigSummary pipeline in deeptools was used to calculate the PTBP1 binding level on each genes. The PTBP1 binding reads coverage files was visualized by IGV (v 2.11.4) software.

To identify circRNAs in high throughput sequencing data. Firstly, the adapters and low quality reads were trimmed by cutadapt (v4.2, -m 10) to obtain clean reads. Then we aligned the reads to the human genome (hg19) by bwa mem(v0.7.17, default parameter). The gene names and circularized exons of the circRNAs were annotated using the CIRI2 algorithm (v2.0.6, -0) with alignment results. The backsplicing junction (BSJ) reads ≥2 were subjected to further analysis and it was normalized according to sequencing depth. The differentially expressed circRNAs was determined by the “DEseq2” package in R software using the corresponding cutoff (*p* < 0.05, |log2(fold change)|> 1.

### Protein mass spectrometry

The specific silver-stained band was cut, cleaned, and digested in gel with the digestion buffer (100 mM NH_4_HCO_3_, pH8.5) containing trypsin (Promega, V5111). The samples were analyzed using an LC-ESI-MS/MS system after extraction and purification. Protech’s ProtQuest software suite was used to search the mass spectrometric data against the UniProt protein database.

### Western blotting

whole-lung homogenates or cells were extracted, and proteins were separated by 10% SDS-PAGE. The proteins were transferred to polyvinylidene fluoride membranes with antibodies to the following proteins: anti-GAPDH (1:3000, Protentech, 60004-1-Ig); anti-Actin (1:3000, Protentech, 66009-1-Ig); anti-E-Cadherin (1:1000, CST, 24E10); anti-N-Cadherin (1:1000, CST, D4R1H); anti-MMP9 (1:1000, Servicebio, GB15132); anti-MMP2 (1:1000,Abclonal, A19080); anti-SMA(1:3000, protentech, 14395-1-AP); anti-TIMP1 (1:1000, protentech, 16644-1-AP). anti-CollagenI (1:1000, Abclonal, A16891); anti-PTBP1 (1:1000, protentech, 12582-1-AP); anti-AGO2 (1:1000, Sigma, SAB4200085); anti-Cleaved Caspase-3 (1:1000, CST,5A1E); anti-PGRN (1:1000, Abcame, ab208777); anti-PGRN (1:1000, Abcame, ab187070); anti-Histone-h3 (1:3000, protentech, 17168-1-AP); anti-IκBα (1:1000, CST, L35A5); anti-Phospho- IκBα (1:1000, CST,14D4); anti-NF-ΚB p65 (1:1000, CST,D14E12); anti-Phospho-NF-κB p65 (1:1000, CST, 93H1). The secondary antibody was raised against the same species as the primary antibody: anti-Rabbit IgG-HRP (1:5000, Santa Cruz, sc-2357); anti-Mouse IgG (1:5000, Abcame, ab205719); anti-Goat IgG-HRP (1:5000, Santa Cruz, sc-2354). For densitometric analyses, protein bands on the blots were measured by Image J software.

### Elisa

Enzyme-linked immunosorbent assay (ELISA) was performed to quantify the protein levels of PGRN in cell culture supernatant. Cells were seeded in 48-well microplates at a density of 1 × 10^4^ per well. After 48 h of culture, collect the cell culture medium and centrifuge at 1000 *g* for 15 min. The supernatant is collected as the sample and can be used directly in subsequent experiments without dilution. Standards, samples, reagents, and microplate preparations from Progranulin ELISA Kit (Elabscience, E-EL-H1578) were performed as described by the manufacturer. Optical density of each well was measured using a microplate reader at a wavelength of 450 nm (Anthos Labtec Instruments, Austria).

### Immunofluorescence staining

BEAS-2b cells were fixed with 4% formaldehyde after washing with PBS twice, and permeabilized with fresh PBS containing 0.5%Triton X-100 for 5 min at room temperature. After blocking with 1% BSA for 30 min at room temperature, samples were incubated with the indicated antibodies (1:200 diluted in 1% BSA) for 4 h at room temperature. With three 5 min washes in PBST buffer (PBS with 0.4% Tween-20), samples were incubated with Goat Anti-Rabbit Secondary Antibody Alexa Fluor 488 (Abcam, ab150077) (1:200 diluted in 1% BSA) for 2 h at room temperature, protected from light. After three 5 min washes in PBST buffer, nuclei were stained with DAPI, and the images were captured using laser confocal microscopy LSM980 (Zeiss). The following antibodies were used in IF: anti-E-Cadherin (CST, 24E10); anti-N-Cadherin (CST, D4R1H); anti-MMP9 (Servicebio, GB15132); anti-MMP2 (Abclonal, A19080); anti-SMA (protentech, 14395-1-AP); anti-TIMP1 (protentech, 26847-1-AP).

### H&E and Masson

To measure the airway remodeling and emphysema-like alveolar enlargement, Masson trichome and H&E staining were used. Specifically, mouse lungs were perfused, inflated, formalin-fixed, and cut into pieces of 3-4 μm thickness. Slides were deparaffinized serial passage twice in xylene, followed by a graded ethanol series. Slides were stained with Masson trichome (Servicebio, Wuhan, China) to identify collagen. Six small airways were randomly selected in each mouse lung, and collagen deposition (blue color) around the small airways was normalized to the perimeter of the airways. For morphological changes, lung slides were stained using hematoxylin and eosin (H&E, Servicebio, Wuhan, China). Four small airways were randomly selected in each mouse lung and epithelial cell size in the small airways was normalized to the perimeter of the basement membrane. Emphysema-like alveolar enlargements were assessed in the lung parenchyma (excluding airways and blood vessels) using mean linear intercepts (MLIs) and destructive indexes (DIs).

### Immunohistochemistry

For IHC staining, paraffin sections of murine tissues were deparaffinized with xylene and rehydrated with ethanol at decreasing concentrations. Then, samples were incubated with the indicated antibodies (1:200 diluted) overnight at 4 °C followed by incubating with the correspondent secondary antibodies (1:200 diluted) for 1 h at room temperature. Nuclei were stained with hematoxylin and images were captured using an inverted microscope (Olympus). The following antibodies were used in IHC: anti-CollagenI (Abclonal, A16891); anti-E-Cadherin (CST, 24E10); anti-N-Cadherin (CST, D4R1H); anti-MMP9 (Servicebio, GB15132); anti-MMP2 (Abclonal, A19080); anti-SMA (protentech, 14395-1-AP); anti-TIMP1 (protentech, 26847-1-AP); anti-PGRN (Abcame, ab208777); anti-PGRN (Protentech, 18410-1-AP); anti-Rabbit IgG-HRP (Santa Cruz, sc-2357); anti-Mouse IgG (Abcame, ab205719).

### Statistical analysis

Data were expressed as the means ± SD taken from at least three independent experiments. Statistical analyses were performed using GraphPad Prism 9 software, with *P* < 0.05 considered as significant. All the data were tested for normality and homogeneity of variance. For two groups of variables, data that did not follow a normal distribution, we calculated *p*-values with the Mann‒Whitney U test. For normally distributed data with homogeneity of variance, we calculated *p*-values with the unpaired t-test. For normally distributed data with heterogeneity of variance, we applied Welch’s t-test to calculate p-values. For data with more than three groups, non-normally distributed data were analyzed using the Kruskal-Wallis test. For normally distributed data with homogeneity of variance, we calculated p-values with one-way ANOVA followed by post-hoc tests. For normally distributed data with heterogeneity of variance, we applied Welch’s ANOVA to calculate p-values. The tests applied are indicated in the corresponding figure legends. Pearson’s correlation coefficient is used to calculate the correlation between two parameters. The sample size (N) was represented in the corresponding figure legends.

## Supplementary information

Supplementary Figures

Supplementary table1

Supplementary table2

Original western blots

## Data Availability

All data are available in the main article or in the materials. The sequencing reads from this study are deposited into the National Center for Biotechnology Information (NCBI) Gene Expression Omnibus with the accession number GSE286017 (https://www.ncbi.nlm.nih.gov/geo/query/acc.cgi?acc=GSE286017).
