# Supplementary material for: Circular RNA FCHO2 promotes airway remodeling in COPD via regulating nuclear translocation of PTBP1 to repress the splicing of GRN pre-mRNA
Source: Cell Death Dis. 2025 Nov 3;16(1):779. doi: 10.1038/s41419-025-08107-9 (PMC12583663; doi:10.1038/s41419-025-08107-9)
Supplement: Supplementary file 1 — Supplementary Figures [file 41419_2025_8107_MOESM1_ESM.docx]

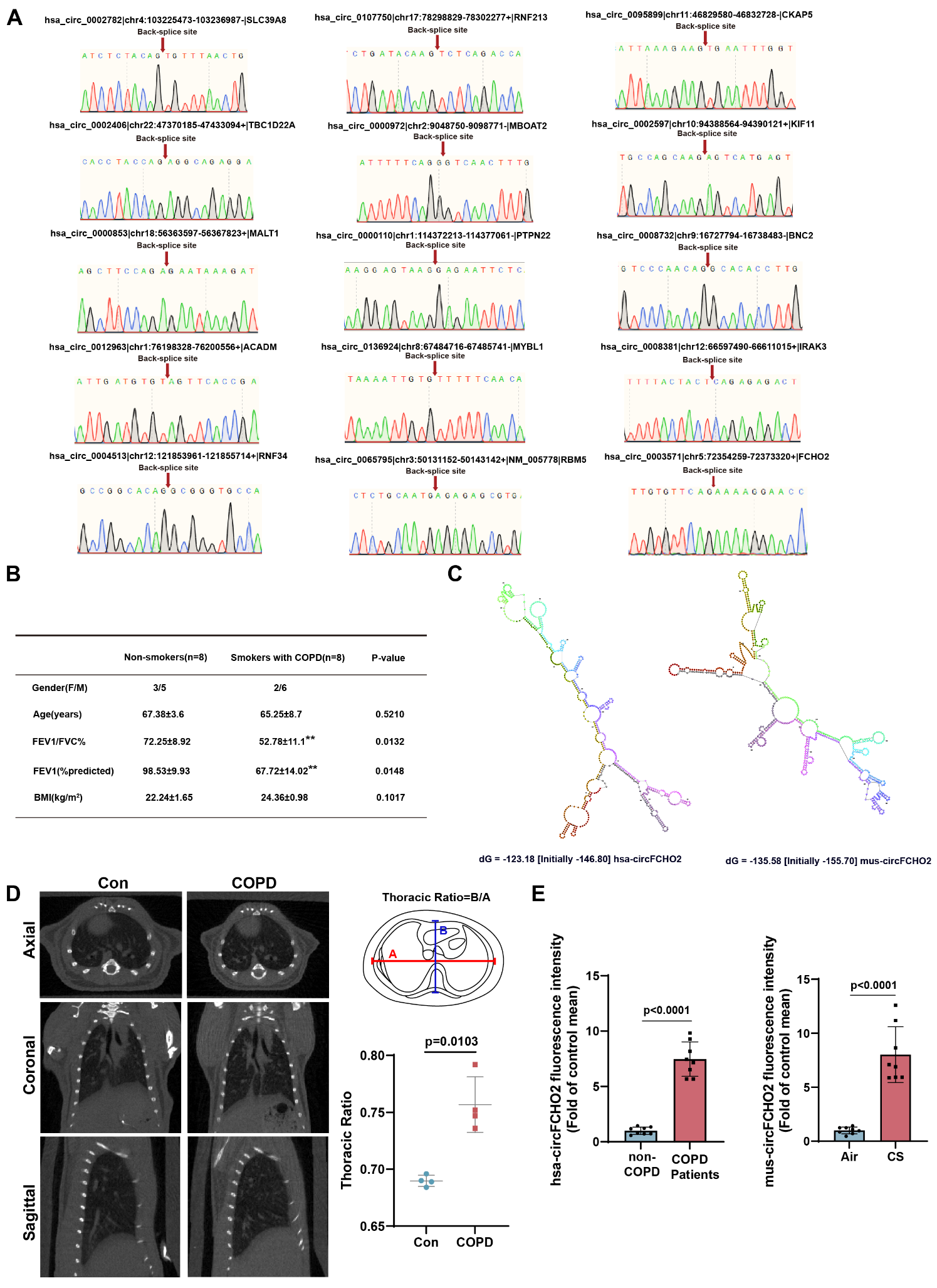


**Figure S1. Mammalian circFCHO2 is related to COPD, related to Figure 1.**

(A) Demonstration of the backsplicing sites of 15 circRNAs by Sanger sequencing using divergent primers. The red arrows show the backsplicing sites. (B) Clinical information for the lung tissue donors. (C) RNA secondary structure prediction of human and mouse circFCHO2 indicated by Mfold. (D) Representative axial (top row), coronal (middle row), and sagittal (bottom row) microcomputed X-ray microscopy (micro-CT) (n=4). (E) Quantification of FISH in human and mouse lung tissues revealed the fluorescence intensity in human and mouse circFCHO2 (n=8). For A ,descriptive data for the intervention and comparison groups were compared using the chi-square test for categorical variables and ANOVA for quantitative data. For C and D, *P* values were calculated via Welch's t-test. The data are shown as the means ± SDs from independent experiments.

**
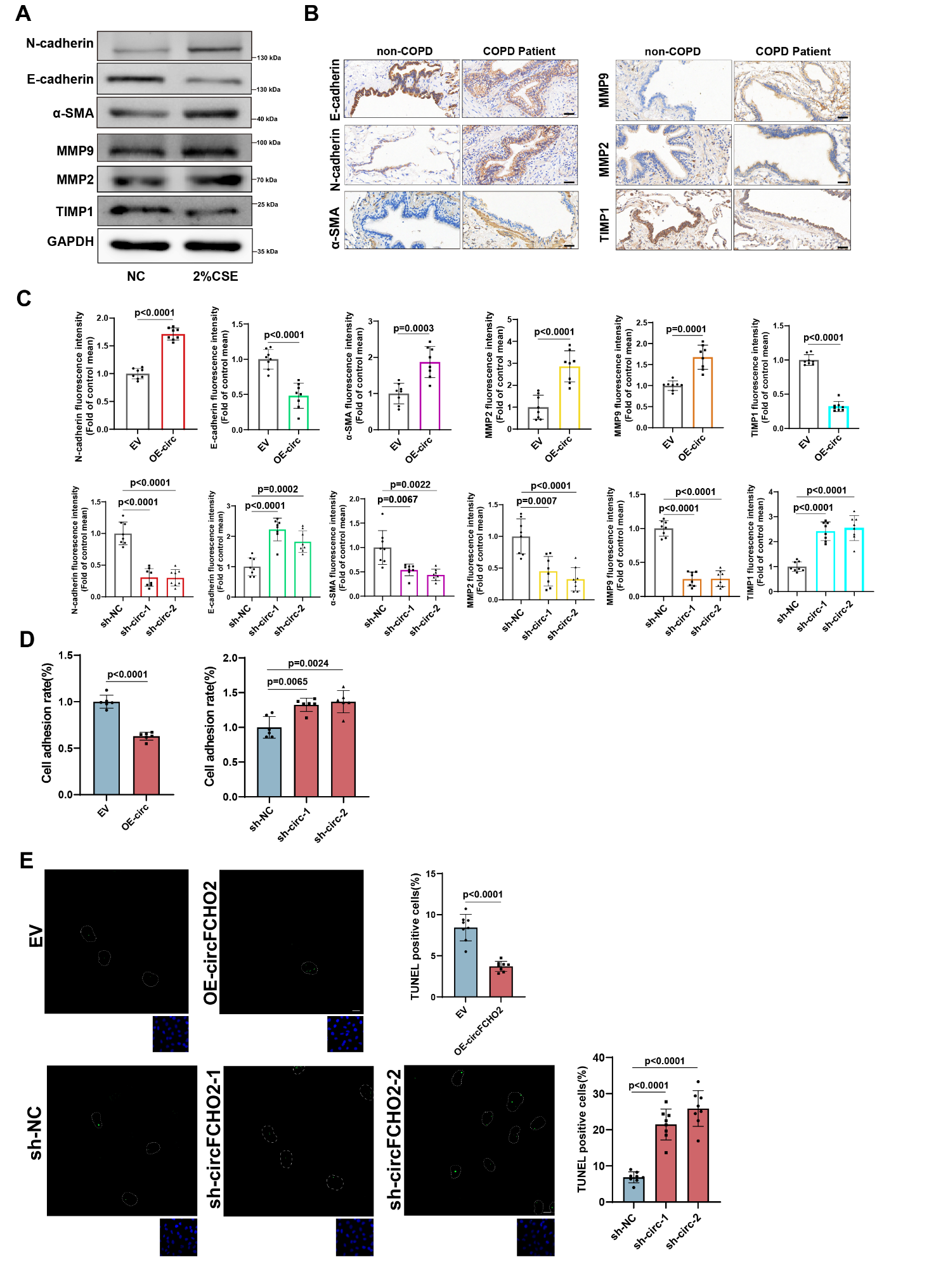
**

**Figure S2. The function of circFCHO2 in bronchial epithelial cells, related to Figure 2.**

(A) Western blots showing EMT- and ECM remodeling -related proteins in BEAS-2B cells treated with 2% CSE. (B) IHC results showing EMT- and ECM remodeling -related proteins in human lung tissues. Scale bar: 50 μm. (C) Quantification of IF in OE and KD cells revealed the fluorescence intensity of EMT- and ECM remodeling -related proteins (n=8). (D) Bar plots showing the cell adhesion rate determined via a microplate reader (n=6). (E) TUNEL assay results showing DNA damage in cells. The white dotted line represents TUNEL-positive cells. Bar plots were used to calculate the percentage of apoptotic cells to total cells (n=8). For C, D and E, *P* values were calculated from two-tailed unpaired Student’s t test, Welch's t-test and one-way ANOVA. The data are shown as the means ± SDs from independent experiments.


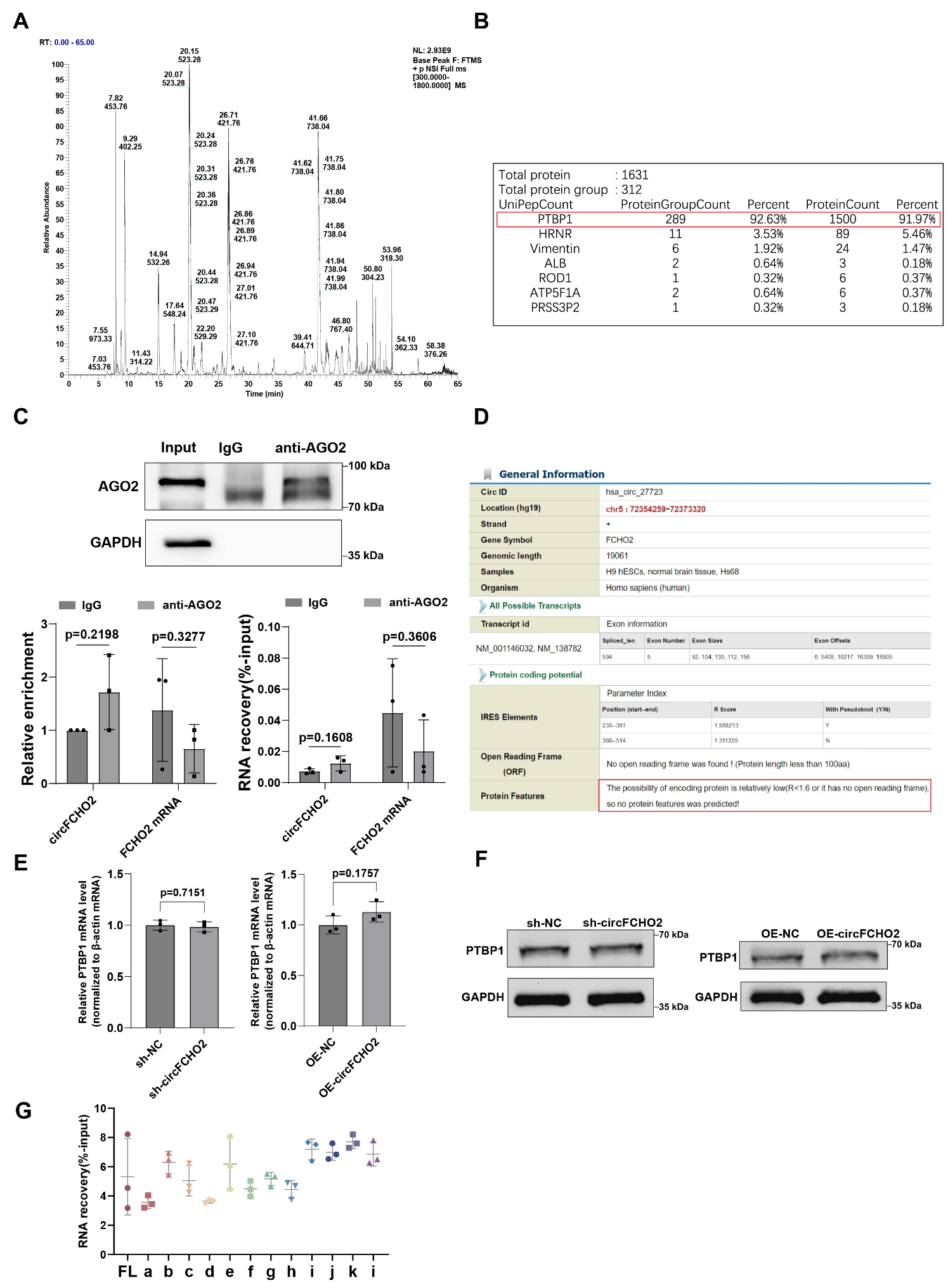


**Figure S3**. **Potential functional mechanism of circFCHO2, related to Figure 3.**

(A) LC‒MS/MS analysis results. (B) The table shows the proportion of top-ranked high-confidence proteins identified in the MS analysis. (C) RIP with an antibody against Ago2 was performed in BEAS-2B cells. The RIP efficiency of the Ago2 protein was validated through western blotting. (B) Bar plots showing no significant enrichment of circFCHO2 in AGO2 RIP (n=3). (D) circFCHO2 does not have an ORF or IRES predicted by circRNA Db. (E) Bar plots showing PTBP1 mRNA levels in circFCHO2 OE and KD cells (n=3). (F) Western blots showing PTBP1 protein levels in circFCHO2 OE and KD cells.(G) Pull-down efficiency of circFCHO2 was examined by RT-qPCR following the overexpression of full-length and truncated PTBP1 in BEAS-2B cells (n=3). C, *P* values from Welch's t-test. For E, *P* values were calculated from two-tailed unpaired Student’s t test. The data are shown as the means ± SDs from independent experiments. ns, not significant.

**
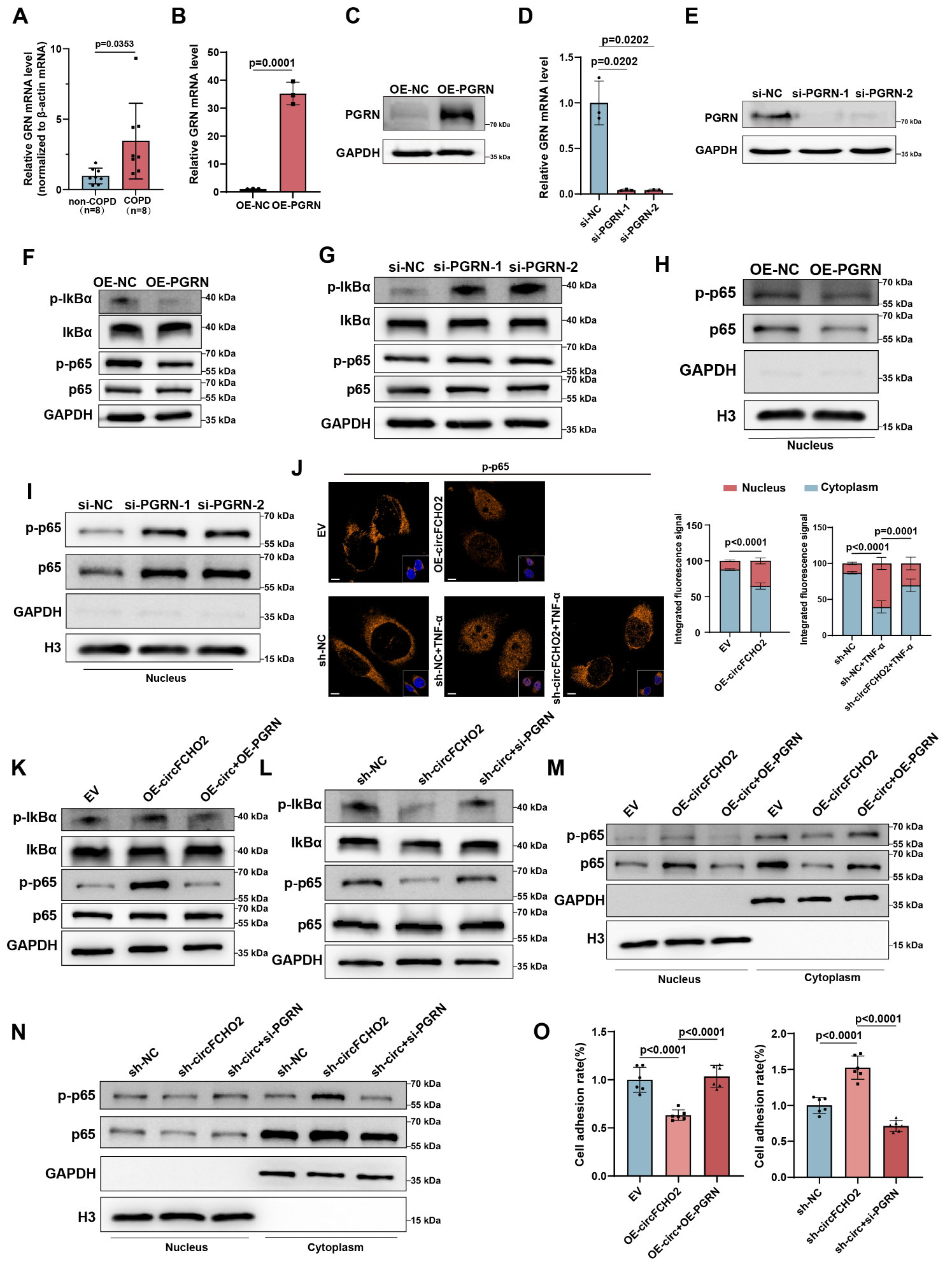
**

**Figure S4. The downstream function of the interaction between circFCHO2 and PTBP1, related to Figure 5.**

(A) GRN mRNA levels were increased in COPD patients (n=8). (B) Bar plot showing the GRN mRNA level in PGRN-overexpressed cells (n=3). (C) Western bolt analysis of PGRN protein levels in OE-PGRN cells. (D) Bar plot showing the GRN mRNA level in si-PGRN cells. (E) Western bolt analysis of PGRN protein levels in si-PGRN cells (n=3). (F) Western bolt showing the relative expression levels of p-IκBα/ IκBα and p-p65/p65 in OE-PGRN cells. (G) Western bolt showing the relative expression levels of p-IκBα/ IκBα and p-p65/p65 in si-PGRN cells. (H) The levels of p65 and p-p65 in the nuclei of PGRN OE cells. (I) The levels of p65 and p-p65 in the nuclei of PGRN KD cells. (J) Immunofluorescence (IF) staining of p-p65 (orange) in OE and KD cells. The final concentration of TNF-α used for cell treatment was 20 ng/mL. The quantification of nuclear (Nuc)/cytoplasmic (Cyto) p-p65 signals is shown as bar plots (n=6). Scale bar, 10 μm. (K) The relative expression levels of p-IκBα/ IκBα and p-p65/p65 were examined by western blot in circFCHO2 OE cells with PGRN OE. (L) The relative expression levels of p-IκBα/ IκBα and p-p65/p65 were examined by western blotting in circFCHO2 KD cells with PGRN KD. (M) The levels of p65 and p-p65 in the nucleus and cytoplasm of circFCHO2 OE cells with PGRN OE. (N) The levels of p65 and p-p65 in the nucleus and cytoplasm of circFCHO2 KD cells with PGRN KD. (O) Bar plots of the cell adhesion rate in circFCHO2 OE and KD cells with PGRN OE and KD were generated via a microplate reader(n=6). For B and O, *P* values were calculated from two-tailed unpaired Student’s t test. For A and D, *P* values were calculated via Welch's t-test. J, *P* values from one-way ANOVA. The data are shown as the means ± SDs from independent experiments.

**
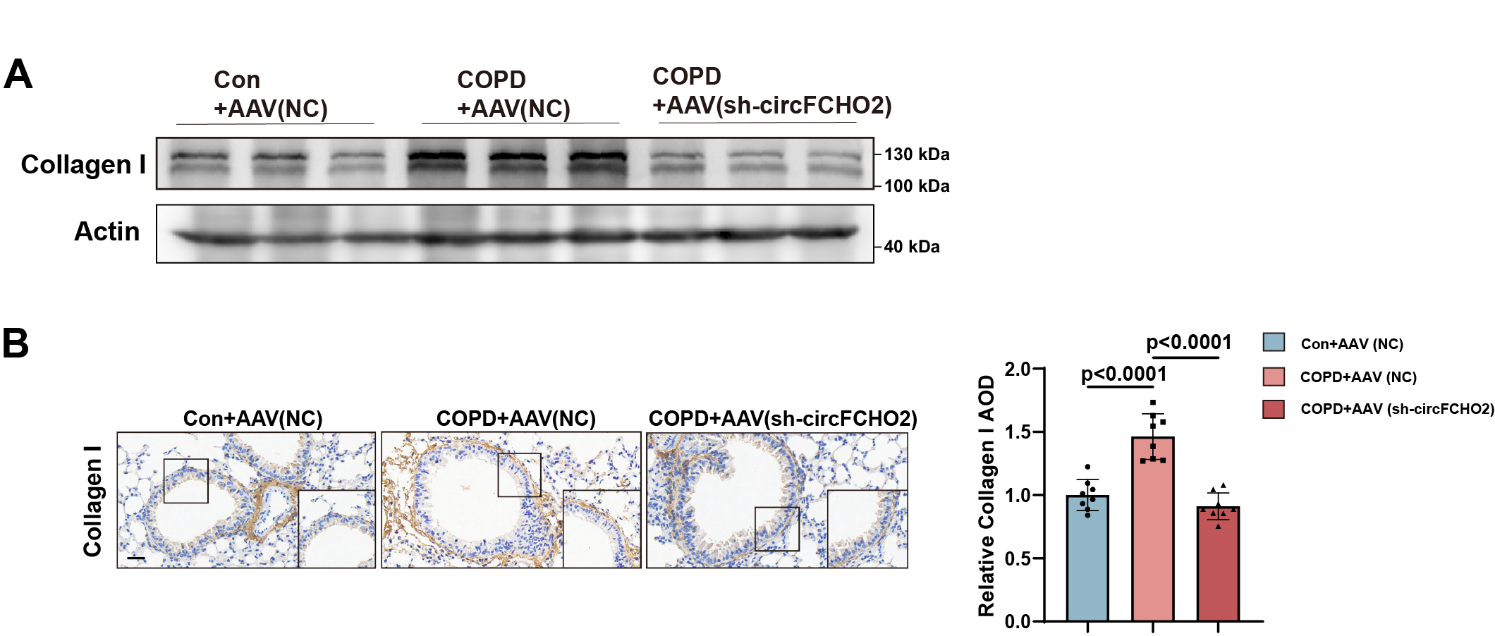
**

**Figure S5. The physiological function of circFCHO2 in mice, related to Figure 6.**

(A) Western blot analysis of Collagen I protein levels in mouse lung tissues. (B) IHC showed the collagen I protein level around small airways in mouse lung tissues (n=8). Scale bar: 50 μm.For B, *P* valuesm were calculated via two-tailed unpaired Student’s t test. The data are shown as the means ± SDs from independent experiments.
