# Supplementary material for: Circular RNA FCHO2 promotes airway remodeling in COPD via regulating nuclear translocation of PTBP1 to repress the splicing of GRN pre-mRNA
Source: Cell Death Dis. 2025 Nov 3;16(1):779. doi: 10.1038/s41419-025-08107-9 (PMC12583663; doi:10.1038/s41419-025-08107-9)
Supplement: Supplementary file 2 — Supplementary table1 [file 41419_2025_8107_MOESM2_ESM.pdf]

## Primer sequence

| | |
|---|---|
| hsa-circFCHO2-Divergent-F | CACCAGCAATCCAACTCCAG |
| hsa-circFCHO2-Divergent-R | AGGGGGTCCCAAGCAAGTA |
| hsa-circFCHO2-Convergent-F | TGGGAAATGTACCTAGCAAGC |
| hsa-circFCHO2-Convergent-R | TGGAAGACAGATGTGCATTGT |
| mus-circFCHO2-Divergent-F | AGCAGATCCTTCCAAACTCCC |
| mus-circFCHO2-Divergent-R | GACTCAAGCGACGATCCGA |
| mus-circFCHO2-Convergent-F | GTGATGTACCGTAGCCACTAGA |
| mus--circFCHO2-Convergent-R | GCTTGTCTGCTTCACGCATT |
| hsa-circFCHO2-F | CACCAGCAATCCAACTCCAG |
| hsa-circFCHO2-R | AGGGGGTCCCAAGCAAGTA |
| hsa-circPTPN22-F | ACAGACACTGAAGACTCCTGG |
| hsa-circPTPN22-R | TGTTCCACCCCATTCCAGTG |
| hsa-circIRAK3-F | TGGAGAAGGAGAGATTTTTGAGG |
| hsa-circIRAK3-R | ATGACGAACATCCAGCCAGC |
| hsa-circSLC39A8-F | GCTATGCAAATCCTGCTGTCAC |
| hsa-circSLC39A8-R | GGAGCTGGTTATTTGGGTAGCA |
| hsa-circTBC1D22A-F | TTCCGCTGGATGAACAACCT |
| hsa-circTBC1D22A-R | TCATGCACCAGTAGGTGTCG |
| hsa-circRNF213-F | CTGAAGATGTGTGGGAACGTG |
| hsa-circRNF213-R | GCAGACAAGACGATGCCAAAT |
| hsa-circMBOAT2-F | GGATACCTCACAGTGTGCCA |
| hsa-circMBOAT2-R | GTTCGAAACCAAATGGCTGCT |
| hsa-circBNC2-F | AACGACTACGGCTGAACCAG |
| hsa-circBNC2-R | ATATGCTGGCCAGTCTTGCT |
| hsa-circMALT1-F | ATTCAGCCAGTGGTCACAGC |
| hsa-circMALT1-R | GCCAAGACTGCCTTTGACTC |
| hsa-circMYBL1-F | TAAGAATGCGCTTGCTGCTC |
| hsa-circMYBL1-R | ATGAGAGCTTTCTGCCCACA |
| hsa-circACADM-F | ATTCTTTGGGGCAAATGCCTA |
| hsa-circACADM-R | ATATTCTGCAGCCACTGGGAT |
| hsa-circRNF34-F | ACAGCCCCCTCTGCTACTAT |
| hsa-circRNF34-R | AGCACGAAGCCCACATAGAC |
| hsa-circRBM5-F | AGCTGTCAATAACATCCGCCT |
| hsa-circRBM5-R | TCTTCGGATCGGTCACTGTT |
| hsa-circCKAP5-F | GGAAGCTGGCGATTATGCAG |
| hsa-circCKAP5-R | ATCTCGAACAGCCTTCTCTCG |
| hsa-circKIF11-F | TCAGCTTTGGAAAGTACTGAGGA |
| hsa-circKIF11-R | CTCCTCCTCAACAGCACCAAT |
| mus-circFCHO2-F | AGCAGATCCTTCCAAACTCCC |
| mus-circFCHO2-R | GACTCAAGCGACGATCCGA |
| hsa-MALT1-F | GCTCTGTGGTGTGGGATTGA |
| hsa-MALT1-R | AAGACAGCCACACGTTTTGC |
| hsa-PTBP1-F | TCTACTTGTGTCACTAACGGAC |
| hsa-PTBP1-R | TGAACTTCTTGCTGTCATTTCC |
| hsa-PGRN-F | GGCTGCTGTCCAATCCCAG |
| hsa-PGRN-R | TGACACTGCCCCTCAGCTAC |
| mus-PGRN-F | TGCTGCAAACTCAATTCTGGG |
| mus-PGRN-R | TGAGCCAGACATGTGAAGCC |
| hsa&mus-ACTB-F | GAGTACTTGCGCTCAGGAG |
| hsa&mus-ACTB-R | CCAACACAGTGCTGTCTGG |
| pre-GRN-F | TCCCACCCCAGAGACATCG |
| pre-GRN-R | GGGCTTAGCTCCCCTATCCAG |
| GRN-exon10-F | GTGTGACACGCAGAAGGGTA |
| GRN-intron10-R1 | GCTGGGTATCACCTATGGGC |
| GRN-exon11-R2 | CTGGGGTGGGATAAGGAAGC |

## shRNA sequence

| | |
|---|---|
| sh-circFCHO2-1 | CCGG-CTTGTGTTCAGAAAAGGAACC-CTCGAG-GGTTCCTTTTCTGAACACAAG-TTTTTT |
| sh-circFCHO2-2 | CCGG-GTGTTCAGAAAAGGAACCAGT-CTCGAG-ACTGGTTCCTTTTCTGAACAC-TTTTTT |

## siRNA sequence

| | |
|---|---|
| si-PGRN-1 | sense: GUGACCUGAUCCAGAGUAATT; antisense: UUACUCUGGAUCAGGUCACTT |
| si-PGRN-2 | sense: GUGCUGUGUUAUGGUCGAUTT; antisense: AUCGACCAUAACACAGCACTT |

| | |
|---|---|
| si-Negative Control | sense:UUCUCCGAACGUGUCACGUTT;<br>antisense: ACGUGACACGUUCGGAGAATT |

## pulldown probe sequence

| | |
|---|---|
| hsa-scramble-pulldown | TGAGGTCAAGACCACCTACAAGGCCAAGAAG |
| hsa-circFCHO2-pulldown | CTGGTTCCTTTTCTGAACACAAGCTGTGCA |

## Single-molecule fluorescence probe sequence

| | |
|---|---|
| sm-FISH-1 | GAGGAGGGCAGCAAACGGAACCTTTTCTGAAC<br>ACAAGCTGTGCAT |
| sm-FISH-2 | GGGTCCCAAGCAAGTAAATCACTGGTAGAAGA<br>GTCTTCCTTTACG |

| | **the sequence of the truncated mutations for PTBP1** | **Target sequence**(Kozak:GCCACC) |
|---|---|---|
| PTBP1-FL (NLS-RRM1-RRM2-RRM3-RRM4) | MDGIVPDIAVGTKRGSDELFSTCVTNGPFIMSSNSASAAN<br>GNDSKKFKGDSRSAGVPSRVIHIRKLPIDVTEGEVISLGLPF<br>GKVTNLLMLKGKNQAFIEMNTEEAANTMVNYYTSVTPVL<br>RGQPIYIQFSNHKELKTDSSPNQARAQAALQAVNSVQSG<br>NLALAASAAAVDAGMAMAGQSPVLRIIVENLFYPVTLDV<br>LHQIFSKFGTVLKIITFTKNNQFQALLQYADPVSAQHAKLS<br>LDGQNIYNACCTLRIDFSKLTSLNVKYNNDKSRDYTRPDL<br>PSGDSQPSLDQTMAAAFGAPGIISASPYAGAGFPPTFAIP<br>QAAGLSVPNVHGALAPLAIPSAAAAAAAAGRIAIPGLAGA<br>GNSVLLVSNLNPERVTPQSLFILFGVYGDVQRVKILFNKKE<br>NALVQMADGNQAQLAMSHLNGHKLHGKPIRITLSKHQN<br>VQLPREGQEDQGLTKDYGNSPLHRFKKPGSKNFQNIFPPS<br>ATLHLSNIPPSVSEEDLKVLFSSNGGVVKGFKFFQKDRKMA<br>LIQMGSVEEAVQALIDLHNHDLGENHHLRVSFSKSTI | GCCACCATGGACGGCATTGTCCCAGATATAGCCGTTGGTACAAAGCGGGGATCTGACGAGCTTTTCTCTACTTGTGTCACTAA<br>CGGACCGTTTATCATGAGCAGCAACTCGGCTTCTGCAGCAAACGGAAATGACAGCAAGAAGTTCAAAGGTGACAGCCGAAGT<br>GCAGGCGTCCCCTCTAGAGTGATCCACATCCGGAAGCTCCCCATCGACGTCACGGAGGGGGAAGTCATCTCCCTGGGGCTGC<br>CCTTTGGGAAGGTCACCAACCTCCTGATGCTGAAGGGGAAAAACCAGGCCTTCATCGAGATGAACACGGAGGAGGCTGCCAA<br>CACCATGGTGAACTACTACACCTCGGTGACCCCTGTGCTGCGCGGCCAGCCCATCTACATCCAGTTCTCCAACCACAAGGAG<br>CTGAAGACCGACAGCTCTCCCAACCAGGCGCGGGCCCAGGCGGCCCTGCAGGCGGTGAACTCGGTCCAGTCGGGGAACCTG<br>GCCTTGGCTGCCTCGGCGGCGGCCGTGGACGCAGGGATGGCGATGGCCGGGCAGAGCCCCGTGCTCAGGATCATCGTGGAG<br>AACCTCTTCTACCCTGTGACCCTGGATGTGCTGCACCAGATTTTCTCCAAGTTCGGCACAGTGTTGAAGATCATCACCTTCACC<br>AAGAACAACCAGTTCCAGGCCCTGCTGCAGTATGCGGACCCCGTGAGCGCCCAGCACGCCAAGCTGTCGCTGGACGGGCAG<br>AACATCTACAACGCCTGCTGCACGCTGCGCATCGACTTTTCCAAGCTCACCAGCCTCAACGTCAAGTACAACAATGACAAGAG<br>CCGTGACTACACACGCCCAGACCTGCCTTCCGGGGACAGCCAGCCCTCGCTGGACCAGACCATGGCCGCGGCCTTCGGTGC<br>ACCTGGTATAATCTCAGCCTCTCCGTATGCAGGAGCTGGTTTCCCTCCCACCTTTGCCATTCCTCAAGCTGCAGGCCTTTCCGT<br>TCCGAACGTCCACGGCGCCCTGGCCCCCCTGGCCATCCCCTCGGCGGCGGCGGCAGCTGCGGCGGCAGGTCGGATCGCCAT<br>CCCGGGCCTGGCGGGGGCAGGAAATTCTGTATTGCTGGTCAGCAACCTCAACCCAGAGAGAGTCACACCCCAAAGCCTCTTT<br>ATTCTTTTCGGCGTCTACGGTGACGTGCAGCGCGTGAAGATCCTGTTCAATAAGAAGGAGAACGCCCTAGTGCAGATGGCGGA<br>CGGCAACCAGGCCCAGCTGGCCATGAGCCACCTGAACGGGCACAAGCTGCACGGGAAGCCCATCCGCATCACGCTCTCGAA<br>GCACCAGAACGTGCAGCTGCCCCGCGAGGGCCAGGAGGACCAGGGCCTGACCAAGGACTACGGCAACTCACCCCTGCACC<br>GCTTCAAGAAGCCGGGCTCCAAGAACTTCCAGAACATATTCCCGCCCTCGGCCACGCTGCACCTCTCCAACATCCCGCCCTC<br>AGTCTCCGAGGAGGATCTCAAGGTCCTGTTTTCCAGCAATGGGGGCGTCGTCAAAGGATTCAAGTTCTTCCAGAAGGACCGCA<br>AGATGGCACTGATCCAGATGGGCTCCGTGGAGGAGGCGGTCCAGGCCCTCATTGACCTGCACAACCACGACCTCGGGGAGA<br>ACCACCACCTGCGGGTCTCCTTCTCCAAGTCCACCATC |
| PTBP1-a (RRM1-RRM2-RRM3-RRM4) | RVIHIRKLPIDVTEGEVISLGLPFGKVTNLLMLKGKNQAFIE<br>MNTEEAANTMVNYYTSVTPVLRGQPIYIQFSNHKELKTDS<br>SPNQARAQAALQAVNSVQSGNLALAASAAAVDAGMA<br>MAGQSPVLRIIVENLFYPVTLDVLHQIFSKFGTVLKIITFTKN<br>NQFQALLQYADPVSAQHAKLSLDGQNIYNACCTLRIDFS<br>KLTSLNVKYNNDKSRDYTRPDLPSGDSQPSLDQTMAAAF<br>GAPGIISASPYAGAGFPPTFAIPQAAGLSVPNVHGALAPLA<br>IPSAAAAAAAAGRIAIPGLAGAGNSVLLVSNLNPERVTPQ<br>SLFILFGVYGDVQRVKILFNKKENALVQMADGNQAQLAM<br>SHLNGHKLHGKPIRITLSKHQNVQLPREGQEDQGLTKDY<br>GNSPLHRFKKPGSKNFQNIFPPSATLHLSNIPPSVSEEDLKV<br>LFSSNGGVVKGFKFFQKDRKMALIQMGSVEEAVQALIDLH<br>NHDLGENHHLRVSFSKSTI | GCCACCATGAGAGTGATCCACATCCGGAAGCTCCCCATCGACGTCACGGAGGGGGAAGTCATCTCCCTGGGGCTGCC<br>CTTTGGGAAGGTCACCAACCTCCTGATGCTGAAGGGGAAAAACCAGGCCTTCATCGAGATGAACACGGAGGAGGCTG<br>CCAACACCATGGTGAACTACTACACCTCGGTGACCCCTGTGCTGCGCGGCCAGCCCATCTACATCCAGTTCTCCAACC<br>ACAAGGAGCTGAAGACCGACAGCTCTCCCAACCAGGCGCGGGCCCAGGCGGCCCTGCAGGCGGTGAACTCGGTCCA<br>GTCGGGGAACCTGGCCTTGGCTGCCTCGGCGGCGGCCGTGGACGCAGGGATGGCGATGGCCGGGCAGAGCCCCGTG<br>CTCAGGATCATCGTGGAGAACCTCTTCTACCCTGTGACCCTGGATGTGCTGCACCAGATTTTCTCCAAGTTCGGCACAG<br>TGTTGAAGATCATCACCTTCACCAAGAACAACCAGTTCCAGGCCCTGCTGCAGTATGCGGACCCCGTGAGCGCCCAG<br>CACGCCAAGCTGTCGCTGGACGGGCAGAACATCTACAACGCCTGCTGCACGCTGCGCATCGACTTTTCCAAGCTCAC<br>CAGCCTCAACGTCAAGTACAACAATGACAAGAGCCGTGACTACACACGCCCAGACCTGCCTTCCGGGGACAGCCAGC<br>CCTCGCTGGACCAGACCATGGCCGCGGCCTTCGGTGCACCTGGTATAATCTCAGCCTCTCCGTATGCAGGAGCTGGTT<br>TCCCTCCCACCTTTGCCATTCCTCAAGCTGCAGGCCTTTCCGTTCCGAACGTCCACGGCGCCCTGGCCCCCCTGGCCA<br>TCCCCTCGGCGGCGGCGGCAGCTGCGGCGGCAGGTCGGATCGCCATCCCGGGCCTGGCGGGGGCAGGAAATTCTGT<br>ATTGCTGGTCAGCAACCTCAACCCAGAGAGAGTCACACCCCAAAGCCTCTTTATTCTTTTCGGCGTCTACGGTGACGTG<br>CAGCGCGTGAAGATCCTGTTCAATAAGAAGGAGAACGCCCTAGTGCAGATGGCGGACGGCAACCAGGCCCAGCTGG<br>CCATGAGCCACCTGAACGGGCACAAGCTGCACGGGAAGCCCATCCGCATCACGCTCTCGAAGCACCAGAACGTGCA<br>GCTGCCCCGCGAGGGCCAGGAGGACCAGGGCCTGACCAAGGACTACGGCAACTCACCCCTGCACCGCTTCAAGAAG<br>CCGGGCTCCAAGAACTTCCAGAACATATTCCCGCCCTCGGCCACGCTGCACCTCTCCAACATCCCGCCCTCAGTCTCC<br>GAGGAGGATCTCAAGGTCCTGTTTTCCAGCAATGGGGGCGTCGTCAAAGGATTCAAGTTCTTCCAGAAGGACCGCAAG<br>ATGGCACTGATCCAGATGGGCTCCGTGGAGGAGGCGGTCCAGGCCCTCATTGACCTGCACAACCACGACCTCGGGGA<br>GAACCACCACCTGCGGGTCTCCTTCTCCAAGTCCACCATC |
| PTBP1-b (NLS-RRM2-RRM3-RRM4) | MDGIVPDIAVGTKRGSDELFSTCVTNGPFIMSSNSASAAN<br>GNDSKKFKGDSRSAGVPSQARAQAALQAVNSVQSGNLA<br>LAASAAAVDAGMAMAGQSPVLRIIVENLFYPVTLDVLHQI<br>FSKFGTVLKIITFTKNNQFQALLQYADPVSAQHAKLSLDG<br>QNIYNACCTLRIDFSKLTSLNVKYNNDKSRDYTRPDLPSG<br>DSQPSLDQTMAAAFGAPGIISASPYAGAGFPPTFAIPQAA<br>GLSVPNVHGALAPLAIPSAAAAAAAAGRIAIPGLAGAGNS<br>VLLVSNLNPERVTPQSLFILFGVYGDVQRVKILFNKKENAL<br>VQMADGNQAQLAMSHLNGHKLHGKPIRITLSKHQNVQL<br>PREGQEDQGLTKDYGNSPLHRFKKPGSKNFQNIFPPSATL<br>HLSNIPPSVSEEDLKVLFSSNGGVVKGFKFFQKDRKMALIQ<br>MGSVEEAVQALIDLHNHDLGENHHLRVSFSKSTI | GCCACCATGGACGGCATTGTCCCAGATATAGCCGTTGGTACAAAGCGGGGATCTGACGAGCTTTTCTCTACTTGTGTCA<br>CTAACGGACCGTTTATCATGAGCAGCAACTCGGCTTCTGCAGCAAACGGAAATGACAGCAAGAAGTTCAAAGGTGAC<br>AGCCGAAGTGCAGGCGTCCCCTCTCAGGCGCGGGCCCAGGCGGCCCTGCAGGCGGTGAACTCGGTCCAGTCGGGGA<br>ACCTGGCCTTGGCTGCCTCGGCGGCGGCCGTGGACGCAGGGATGGCGATGGCCGGGCAGAGCCCCGTGCTCAGGAT<br>CATCGTGGAGAACCTCTTCTACCCTGTGACCCTGGATGTGCTGCACCAGATTTTCTCCAAGTTCGGCACAGTGTTGAAG<br>ATCATCACCTTCACCAAGAACAACCAGTTCCAGGCCCTGCTGCAGTATGCGGACCCCGTGAGCGCCCAGCACGCCAA<br>GCTGTCGCTGGACGGGCAGAACATCTACAACGCCTGCTGCACGCTGCGCATCGACTTTTCCAAGCTCACCAGCCTCAA<br>CGTCAAGTACAACAATGACAAGAGCCGTGACTACACACGCCCAGACCTGCCTTCCGGGGACAGCCAGCCCTCGCTGG<br>ACCAGACCATGGCCGCGGCCTTCGGTGCACCTGGTATAATCTCAGCCTCTCCGTATGCAGGAGCTGGTTTCCCTCCCA<br>CCTTTGCCATTCCTCAAGCTGCAGGCCTTTCCGTTCCGAACGTCCACGGCGCCCTGGCCCCCCTGGCCATCCCCTCGG<br>CGGCGGCGGCAGCTGCGGCGGCAGGTCGGATCGCCATCCCGGGCCTGGCGGGGGCAGGAAATTCTGTATTGCTGGT<br>CAGCAACCTCAACCCAGAGAGAGTCACACCCCAAAGCCTCTTTATTCTTTTCGGCGTCTACGGTGACGTGCAGCGCGT<br>GAAGATCCTGTTCAATAAGAAGGAGAACGCCCTAGTGCAGATGGCGGACGGCAACCAGGCCCAGCTGGCCATGAGC<br>CACCTGAACGGGCACAAGCTGCACGGGAAGCCCATCCGCATCACGCTCTCGAAGCACCAGAACGTGCAGCTGCCCC<br>GCGAGGGCCAGGAGGACCAGGGCCTGACCAAGGACTACGGCAACTCACCCCTGCACCGCTTCAAGAAGCCGGGCTC<br>CAAGAACTTCCAGAACATATTCCCGCCCTCGGCCACGCTGCACCTCTCCAACATCCCGCCCTCAGTCTCCGAGGAGGA<br>TCTCAAGGTCCTGTTTTCCAGCAATGGGGGCGTCGTCAAAGGATTCAAGTTCTTCCAGAAGGACCGCAAGATGGCACT<br>GATCCAGATGGGCTCCGTGGAGGAGGCGGTCCAGGCCCTCATTGACCTGCACAACCACGACCTCGGGGAGAACCAC<br>CACCTGCGGGTCTCCTTCTCCAAGTCCACCATC |

| | | |
|---|---|---|
| PTBP1-c (NLS-RRM1-RRM3-RRM4) | MDGIVPDIAVGTKRGSDELFSTCVTNGPFIMSSNSASAAN<br>GNDSKKFKGDSRSAGVPSRVIHIRKLPIDVTEGEVISLGLPF<br>GKVTNLLMLKGKNQAFIEMNTEEAANTMVNYYTSVTPVL<br>RGQPIYIQFSNHKELKTDSSPNQARAQAALQAVNSVQSG<br>NLALAASAAAVDAGMAMAGQSPVTSLNVKYNNDKSRD<br>YTRPDLPSGDSQPSLDQTMAAAFGAPGIISASPYAGAGFP<br>PTFAIPQAAGLSVPNVHGALAPLAIPSAAAAAAAAGRIAIP<br>GLAGAGNSVLLVSNLNPERVTPQSLFILFGVYGDVQRVKIL<br>FNKKENALVQMADGNQAQLAMSHLNGHKLHGKPIRITLS<br>KHQNVQLPREGQEDQGLTKDYGNSPLHRFKKPGSKNFQ<br>NIFPPSATLHLSNIPPSVSEEDLKVLFSSNGGVVKGFKFFQK<br>DRKMALIQMGSVEEAVQALIDLHNHDLGENHHLRVSFSK<br>STI | GCCACCATGGACGGCATTGTCCCAGATATAGCCGTTGGTACAAAGCGGGGATCTGACGAGCTTTTCTCTACTTGTGTCA<br>CTAACGGACCGTTTATCATGAGCAGCAACTCGGCTTCTGCAGCAAACGGAAATGACAGCAAGAAGTTCAAAGGTGAC<br>AGCCGAAGTGCAGGCGTCCCCTCTAGAGTGATCCACATCCGGAAGCTCCCCATCGACGTCACGGAGGGGGAAGTCAT<br>CTCCCTGGGGCTGCCCTTTGGGAAGGTCACCAACCTCCTGATGCTGAAGGGGAAAAACCAGGCCTTCATCGAGATGA<br>ACACGGAGGAGGCTGCCAACACCATGGTGAACTACTACACCTCGGTGACCCCTGTGCTGCGCGGCCAGCCCATCTAC<br>ATCCAGTTCTCCAACCACAAGGAGCTGAAGACCGACAGCTCTCCCAACCAGGCGCGGGCCCAGGCGGCCCTGCAGG<br>CGGTGAACTCGGTCCAGTCGGGGAACCTGGCCTTGGCTGCCTCGGCGGCGGCCGTGGACGCAGGGATGGCGATGGC<br>CGGGCAGAGCCCCGTGACCAGCCTCAACGTCAAGTACAACAATGACAAGAGCCGTGACTACACACGCCCAGACCTG<br>CCTTCCGGGGACAGCCAGCCCTCGCTGGACCAGACCATGGCCGCGGCCTTCGGTGCACCTGGTATAATCTCAGCCTC<br>TCCGTATGCAGGAGCTGGTTTCCCTCCCACCTTTGCCATTCCTCAAGCTGCAGGCCTTTCCGTTCCGAACGTCCACGGC<br>GCCCTGGCCCCCCTGGCCATCCCCTCGGCGGCGGCGGCAGCTGCGGCGGCAGGTCGGATCGCCATCCCGGGCCTGG<br>CGGGGGCAGGAAATTCTGTATTGCTGGTCAGCAACCTCAACCCAGAGAGAGTCACACCCCAAAGCCTCTTTATTCTTTT<br>CGGCGTCTACGGTGACGTGCAGCGCGTGAAGATCCTGTTCAATAAGAAGGAGAACGCCCTAGTGCAGATGGCGGACG<br>GCAACCAGGCCCAGCTGGCCATGAGCCACCTGAACGGGCACAAGCTGCACGGGAAGCCCATCCGCATCACGCTCTC<br>GAAGCACCAGAACGTGCAGCTGCCCCGCGAGGGCCAGGAGGACCAGGGCCTGACCAAGGACTACGGCAACTCACC<br>CCTGCACCGCTTCAAGAAGCCGGGCTCCAAGAACTTCCAGAACATATTCCCGCCCTCGGCCACGCTGCACCTCTCCAA<br>CATCCCGCCCTCAGTCTCCGAGGAGGATCTCAAGGTCCTGTTTTCCAGCAATGGGGGCGTCGTCAAAGGATTCAAGTT<br>CTTCCAGAAGGACCGCAAGATGGCACTGATCCAGATGGGCTCCGTGGAGGAGGCGGTCCAGGCCCTCATTGACCTGC<br>ACAACCACGACCTCGGGGAGAACCACCACCTGCGGGTCTCCTTCTCCAAGTCCACCATC |
| PTBP1-d (NLS-RRM1-RRM2-RRM4) | MDGIVPDIAVGTKRGSDELFSTCVTNGPFIMSSNSASAAN<br>GNDSKKFKGDSRSAGVPSRVIHIRKLPIDVTEGEVISLGLPF<br>GKVTNLLMLKGKNQAFIEMNTEEAANTMVNYYTSVTPVL<br>RGQPIYIQFSNHKELKTDSSPNQARAQAALQAVNSVQSG<br>NLALAASAAAVDAGMAMAGQSPVLRIIVENLFYPVTLDV<br>LHQIFSKFGTVLKIITFTKNNQFQALLQYADPVSAQHAKLS<br>LDGQNIYNACCTLRIDFSKLTSLNVKYNNDKSRDYTRPDL<br>PSGDSQPSLDQTMAAAFGAPGIISASPYAGAGFPPTFAIP<br>QAAGLSVPNVHGALAPLAIPSAAAAAAAAGRIAIPGLAGA<br>GNQNVQLPREGQEDQGLTKDYGNSPLHRFKKPGSKNFQ<br>NIFPPSATLHLSNIPPSVSEEDLKVLFSSNGGVVKGFKFFQK<br>DRKMALIQMGSVEEAVQALIDLHNHDLGENHHLRVSFSK<br>STI | GCCACCATGGACGGCATTGTCCCAGATATAGCCGTTGGTACAAAGCGGGGATCTGACGAGCTTTTCTCTACTTGTGTCA<br>CTAACGGACCGTTTATCATGAGCAGCAACTCGGCTTCTGCAGCAAACGGAAATGACAGCAAGAAGTTCAAAGGTGAC<br>AGCCGAAGTGCAGGCGTCCCCTCTAGAGTGATCCACATCCGGAAGCTCCCCATCGACGTCACGGAGGGGGAAGTCAT<br>CTCCCTGGGGCTGCCCTTTGGGAAGGTCACCAACCTCCTGATGCTGAAGGGGAAAAACCAGGCCTTCATCGAGATGA<br>ACACGGAGGAGGCTGCCAACACCATGGTGAACTACTACACCTCGGTGACCCCTGTGCTGCGCGGCCAGCCCATCTAC<br>ATCCAGTTCTCCAACCACAAGGAGCTGAAGACCGACAGCTCTCCCAACCAGGCGCGGGCCCAGGCGGCCCTGCAGG<br>CGGTGAACTCGGTCCAGTCGGGGAACCTGGCCTTGGCTGCCTCGGCGGCGGCCGTGGACGCAGGGATGGCGATGGC<br>CGGGCAGAGCCCCGTGCTCAGGATCATCGTGGAGAACCTCTTCTACCCTGTGACCCTGGATGTGCTGCACCAGATTTT<br>CTCCAAGTTCGGCACAGTGTTGAAGATCATCACCTTCACCAAGAACAACCAGTTCCAGGCCCTGCTGCAGTATGCGGA<br>CCCCGTGAGCGCCCAGCACGCCAAGCTGTCGCTGGACGGGCAGAACATCTACAACGCCTGCTGCACGCTGCGCATC<br>GACTTTTCCAAGCTCACCAGCCTCAACGTCAAGTACAACAATGACAAGAGCCGTGACTACACACGCCCAGACCTGCCT<br>TCCGGGGACAGCCAGCCCTCGCTGGACCAGACCATGGCCGCGGCCTTCGGTGCACCTGGTATAATCTCAGCCTCTCC<br>GTATGCAGGAGCTGGTTTCCCTCCCACCTTTGCCATTCCTCAAGCTGCAGGCCTTTCCGTTCCGAACGTCCACGGCGCC<br>CTGGCCCCCCTGGCCATCCCCTCGGCGGCGGCGGCAGCTGCGGCGGCAGGTCGGATCGCCATCCCGGGCCTGGCGG<br>GGGCAGGAAATCAGAACGTGCAGCTGCCCCGCGAGGGCCAGGAGGACCAGGGCCTGACCAAGGACTACGGCAACT<br>CACCCCTGCACCGCTTCAAGAAGCCGGGCTCCAAGAACTTCCAGAACATATTCCCGCCCTCGGCCACGCTGCACCTCT<br>CCAACATCCCGCCCTCAGTCTCCGAGGAGGATCTCAAGGTCCTGTTTTCCAGCAATGGGGGCGTCGTCAAAGGATTCA<br>AGTTCTTCCAGAAGGACCGCAAGATGGCACTGATCCAGATGGGCTCCGTGGAGGAGGCGGTCCAGGCCCTCATTGAC<br>CTGCACAACCACGACCTCGGGGAGAACCACCACCTGCGGGTCTCCTTCTCCAAGTCCACCATC |
| PTBP1-e (NLS-RRM1-RRM2-RRM3) | MDGIVPDIAVGTKRGSDELFSTCVTNGPFIMSSNSASAAN<br>GNDSKKFKGDSRSAGVPSRVIHIRKLPIDVTEGEVISLGLPF<br>GKVTNLLMLKGKNQAFIEMNTEEAANTMVNYYTSVTPVL<br>RGQPIYIQFSNHKELKTDSSPNQARAQAALQAVNSVQSG<br>NLALAASAAAVDAGMAMAGQSPVLRIIVENLFYPVTLDV<br>LHQIFSKFGTVLKIITFTKNNQFQALLQYADPVSAQHAKLS<br>LDGQNIYNACCTLRIDFSKLTSLNVKYNNDKSRDYTRPDL<br>PSGDSQPSLDQTMAAAFGAPGIISASPYAGAGFPPTFAIP<br>QAAGLSVPNVHGALAPLAIPSAAAAAAAAGRIAIPGLAGA<br>GNSVLLVSNLNPERVTPQSLFILFGVYGDVQRVKILFNKKE<br>NALVQMADGNQAQLAMSHLNGHKLHGKPIRITLSKHQN<br>VQLPREGQEDQGLTKDYGNSPLHRFKKPGSKNFQNIFPPS | GCCACCATGGACGGCATTGTCCCAGATATAGCCGTTGGTACAAAGCGGGGATCTGACGAGCTTTTCTCTACTTGTGTCA<br>CTAACGGACCGTTTATCATGAGCAGCAACTCGGCTTCTGCAGCAAACGGAAATGACAGCAAGAAGTTCAAAGGTGAC<br>AGCCGAAGTGCAGGCGTCCCCTCTAGAGTGATCCACATCCGGAAGCTCCCCATCGACGTCACGGAGGGGGAAGTCAT<br>CTCCCTGGGGCTGCCCTTTGGGAAGGTCACCAACCTCCTGATGCTGAAGGGGAAAAACCAGGCCTTCATCGAGATGA<br>ACACGGAGGAGGCTGCCAACACCATGGTGAACTACTACACCTCGGTGACCCCTGTGCTGCGCGGCCAGCCCATCTAC<br>ATCCAGTTCTCCAACCACAAGGAGCTGAAGACCGACAGCTCTCCCAACCAGGCGCGGGCCCAGGCGGCCCTGCAGG<br>CGGTGAACTCGGTCCAGTCGGGGAACCTGGCCTTGGCTGCCTCGGCGGCGGCCGTGGACGCAGGGATGGCGATGGC<br>CGGGCAGAGCCCCGTGCTCAGGATCATCGTGGAGAACCTCTTCTACCCTGTGACCCTGGATGTGCTGCACCAGATTTT<br>CTCCAAGTTCGGCACAGTGTTGAAGATCATCACCTTCACCAAGAACAACCAGTTCCAGGCCCTGCTGCAGTATGCGGA<br>CCCCGTGAGCGCCCAGCACGCCAAGCTGTCGCTGGACGGGCAGAACATCTACAACGCCTGCTGCACGCTGCGCATC<br>GACTTTTCCAAGCTCACCAGCCTCAACGTCAAGTACAACAATGACAAGAGCCGTGACTACACACGCCCAGACCTGCCT<br>TCCGGGGACAGCCAGCCCTCGCTGGACCAGACCATGGCCGCGGCCTTCGGTGCACCTGGTATAATCTCAGCCTCTCC<br>GTATGCAGGAGCTGGTTTCCCTCCCACCTTTGCCATTCCTCAAGCTGCAGGCCTTTCCGTTCCGAACGTCCACGGCGCC<br>CTGGCCCCCCTGGCCATCCCCTCGGCGGCGGCGGCAGCTGCGGCGGCAGGTCGGATCGCCATCCCGGGCCTGGCGG<br>GGGCAGGAAATTCTGTATTGCTGGTCAGCAACCTCAACCCAGAGAGAGTCACACCCCAAAGCCTCTTTATTCTTTTCGG<br>CGTCTACGGTGACGTGCAGCGCGTGAAGATCCTGTTCAATAAGAAGGAGAACGCCCTAGTGCAGATGGCGGACGGCA<br>ACCAGGCCCAGCTGGCCATGAGCCACCTGAACGGGCACAAGCTGCACGGGAAGCCCATCCGCATCACGCTCTCGAA<br>GCACCAGAACGTGCAGCTGCCCCGCGAGGGCCAGGAGGACCAGGGCCTGACCAAGGACTACGGCAACTCACCCCTG<br>CACCGCTTCAAGAAGCCGGGCTCCAAGAACTTCCAGAACATATTCCCGCCCTCG |
| PTBP1-f (RRM2-RRM3-RRM4) | QARAQAALQAVNSVQSGNLALAASAAAVDAGMAMAG<br>QSPVLRIIVENLFYPVTLDVLHQIFSKFGTVLKIITFTKNNQF<br>QALLQYADPVSAQHAKLSLDGQNIYNACCTLRIDFSKLTS<br>LNVKYNNDKSRDYTRPDLPSGDSQPSLDQTMAAAFGAP<br>GIISASPYAGAGFPPTFAIPQAAGLSVPNVHGALAPLAIPS<br>AAAAAAAAGRIAIPGLAGAGNSVLLVSNLNPERVTPQSLFI<br>LFGVYGDVQRVKILFNKKENALVQMADGNQAQLAMSHL<br>NGHKLHGKPIRITLSKHQNVQLPREGQEDQGLTKDYGNS<br>PLHRFKKPGSKNFQNIFPPSATLHLSNIPPSVSEEDLKVLFSS<br>NGGVVKGFKFFQKDRKMALIQMGSVEEAVQALIDLHNH<br>DLGENHHLRVSFSKSTI | GCCACCATGCAGGCGCGGGCCCAGGCGGCCCTGCAGGCGGTGAACTCGGTCCAGTCGGGGAACCTGGCCTTGGCTG<br>CCTCGGCGGCGGCCGTGGACGCAGGGATGGCGATGGCCGGGCAGAGCCCCGTGCTCAGGATCATCGTGGAGAACCT<br>CTTCTACCCTGTGACCCTGGATGTGCTGCACCAGATTTTCTCCAAGTTCGGCACAGTGTTGAAGATCATCACCTTCACC<br>AAGAACAACCAGTTCCAGGCCCTGCTGCAGTATGCGGACCCCGTGAGCGCCCAGCACGCCAAGCTGTCGCTGGACG<br>GGCAGAACATCTACAACGCCTGCTGCACGCTGCGCATCGACTTTTCCAAGCTCACCAGCCTCAACGTCAAGTACAACA<br>ATGACAAGAGCCGTGACTACACACGCCCAGACCTGCCTTCCGGGGACAGCCAGCCCTCGCTGGACCAGACCATGGC<br>CGCGGCCTTCGGTGCACCTGGTATAATCTCAGCCTCTCCGTATGCAGGAGCTGGTTTCCCTCCCACCTTTGCCATTCCT<br>CAAGCTGCAGGCCTTTCCGTTCCGAACGTCCACGGCGCCCTGGCCCCCCTGGCCATCCCCTCGGCGGCGGCGGCAGC<br>TGCGGCGGCAGGTCGGATCGCCATCCCGGGCCTGGCGGGGGCAGGAAATTCTGTATTGCTGGTCAGCAACCTCAACC<br>CAGAGAGAGTCACACCCCAAAGCCTCTTTATTCTTTTCGGCGTCTACGGTGACGTGCAGCGCGTGAAGATCCTGTTCAA<br>TAAGAAGGAGAACGCCCTAGTGCAGATGGCGGACGGCAACCAGGCCCAGCTGGCCATGAGCCACCTGAACGGGCAC<br>AAGCTGCACGGGAAGCCCATCCGCATCACGCTCTCGAAGCACCAGAACGTGCAGCTGCCCCGCGAGGGCCAGGAGG<br>ACCAGGGCCTGACCAAGGACTACGGCAACTCACCCCTGCACCGCTTCAAGAAGCCGGGCTCCAAGAACTTCCAGAAC<br>ATATTCCCGCCCTCGGCCACGCTGCACCTCTCCAACATCCCGCCCTCAGTCTCCGAGGAGGATCTCAAGGTCCTGTTTT<br>CCAGCAATGGGGGCGTCGTCAAAGGATTCAAGTTCTTCCAGAAGGACCGCAAGATGGCACTGATCCAGATGGGCTCC<br>GTGGAGGAGGCGGTCCAGGCCCTCATTGACCTGCACAACCACGACCTCGGGGAGAACCACCACCTGCGGGTCTCCTT<br>CTCCAAGTCCACCATC |
| PTBP1-g (NLS-RRM3-RRM4) | MDGIVPDIAVGTKRGSDELFSTCVTNGPFIMSSNSASAAN<br>GNDSKKFKGDSRSAGVPSTSLNVKYNNDKSRDYTRPDLPS<br>GDSQPSLDQTMAAAFGAPGIISASPYAGAGFPPTFAIPQA<br>AGLSVPNVHGALAPLAIPSAAAAAAAAGRIAIPGLAGAGN<br>SVLLVSNLNPERVTPQSLFILFGVYGDVQRVKILFNKKENA<br>LVQMADGNQAQLAMSHLNGHKLHGKPIRITLSKHQNVQ<br>LPREGQEDQGLTKDYGNSPLHRFKKPGSKNFQNIFPPSAT<br>LHLSNIPPSVSEEDLKVLFSSNGGVVKGFKFFQKDRKMALI<br>QMGSVEEAVQALIDLHNHDLGENHHLRVSFSKSTI | GCCACCATGGACGGCATTGTCCCAGATATAGCCGTTGGTACAAAGCGGGGATCTGACGAGCTTTTCTCTACTTGTGTCA<br>CTAACGGACCGTTTATCATGAGCAGCAACTCGGCTTCTGCAGCAAACGGAAATGACAGCAAGAAGTTCAAAGGTGAC<br>AGCCGAAGTGCAGGCGTCCCCTCTACCAGCCTCAACGTCAAGTACAACAATGACAAGAGCCGTGACTACACACGCCC<br>AGACCTGCCTTCCGGGGACAGCCAGCCCTCGCTGGACCAGACCATGGCCGCGGCCTTCGGTGCACCTGGTATAATCT<br>CAGCCTCTCCGTATGCAGGAGCTGGTTTCCCTCCCACCTTTGCCATTCCTCAAGCTGCAGGCCTTTCCGTTCCGAACGT<br>CCACGGCGCCCTGGCCCCCCTGGCCATCCCCTCGGCGGCGGCGGCAGCTGCGGCGGCAGGTCGGATCGCCATCCCG<br>GGCCTGGCGGGGGCAGGAAATTCTGTATTGCTGGTCAGCAACCTCAACCCAGAGAGAGTCACACCCCAAAGCCTCTT<br>TATTCTTTTCGGCGTCTACGGTGACGTGCAGCGCGTGAAGATCCTGTTCAATAAGAAGGAGAACGCCCTAGTGCAGAT<br>GGCGGACGGCAACCAGGCCCAGCTGGCCATGAGCCACCTGAACGGGCACAAGCTGCACGGGAAGCCCATCCGCATC<br>ACGCTCTCGAAGCACCAGAACGTGCAGCTGCCCCGCGAGGGCCAGGAGGACCAGGGCCTGACCAAGGACTACGGCA<br>ACTCACCCCTGCACCGCTTCAAGAAGCCGGGCTCCAAGAACTTCCAGAACATATTCCCGCCCTCGGCCACGCTGCAC<br>CTCTCCAACATCCCGCCCTCAGTCTCCGAGGAGGATCTCAAGGTCCTGTTTTCCAGCAATGGGGGCGTCGTCAAAGGA<br>TTCAAGTTCTTCCAGAAGGACCGCAAGATGGCACTGATCCAGATGGGCTCCGTGGAGGAGGCGGTCCAGGCCCTCAT<br>TGACCTGCACAACCACGACCTCGGGGAGAACCACCACCTGCGGGTCTCCTTCTCCAAGTCCACCATC |

| | | |
|---|---|---|
| PTBP1-h (NLS-RRM1-RRM4) | MDGIVPDIAVGTKRGSDELFSTCVTNGPFIMSSNSASAAN<br>GNDSKKFKGDSRSAGVPSRVIHIRKLPIDVTEGEVISLGLPF<br>GKVTNLLMLKGKNQAFIEMNTEEAANTMVNYYTSVTPVL<br>RGQPIYIQFSNHKELKTDSSPNQARAQAALQAVNSVQSG<br>NLALAASAAAVDAGMAMAGQSPVQNVQLPREGQEDQ<br>GLTKDYGNSPLHRFKKPGSKNFQNIFPPSATLHLSNIPPSV<br>SEEDLKVLFSSNGGVVKGFKFFQKDRKMALIQMGSVEEAV<br>QALIDLHNHDLGENHHLRVSFSKSTI | GCCACCATGGACGGCATTGTCCCAGATATAGCCGTTGGTACAAAGCGGGGATCTGACGAGCTTTTCTCTACTTGTGTCA<br>CTAACGGACCGTTTATCATGAGCAGCAACTCGGCTTCTGCAGCAAACGGAAATGACAGCAAGAAGTTCAAAGGTGAC<br>AGCCGAAGTGCAGGCGTCCCCTCTAGAGTGATCCACATCCGGAAGCTCCCCATCGACGTCACGGAGGGGGAAGTCAT<br>CTCCCTGGGGCTGCCCTTTGGGAAGGTCACCAACCTCCTGATGCTGAAGGGGAAAAACCAGGCCTTCATCGAGATGA<br>ACACGGAGGAGGCTGCCAACACCATGGTGAACTACTACACCTCGGTGACCCCTGTGCTGCGCGGCCAGCCCATCTAC<br>ATCCAGTTCTCCAACCACAAGGAGCTGAAGACCGACAGCTCTCCCAACCAGGCGCGGGCCCAGGCGGCCCTGCAGG<br>CGGTGAACTCGGTCCAGTCGGGGAACCTGGCCTTGGCTGCCTCGGCGGCGGCCGTGGACGCAGGGATGGCGATGGC<br>CGGGCAGAGCCCCGTGCAGAACGTGCAGCTGCCCCGCGAGGGCCAGGAGGACCAGGGCCTGACCAAGGACTACGG<br>CAACTCACCCCTGCACCGCTTCAAGAAGCCGGGCTCCAAGAACTTCCAGAACATATTCCCGCCCTCGGCCACGCTGC<br>ACCTCTCCAACATCCCGCCCTCAGTCTCCGAGGAGGATCTCAAGGTCCTGTTTTCCAGCAATGGGGGCGTCGTCAAAG<br>GATTCAAGTTCTTCCAGAAGGACCGCAAGATGGCACTGATCCAGATGGGCTCCGTGGAGGAGGCGGTCCAGGCCCTC<br>ATTGACCTGCACAACCACGACCTCGGGGAGAACCACCACCTGCGGGTCTCCTTCTCCAAGTCCACCATC |
| PTBP1-i (NLS-RRM1-RRM2) | MDGIVPDIAVGTKRGSDELFSTCVTNGPFIMSSNSASAAN<br>GNDSKKFKGDSRSAGVPSRVIHIRKLPIDVTEGEVISLGLPF<br>GKVTNLLMLKGKNQAFIEMNTEEAANTMVNYYTSVTPVL<br>RGQPIYIQFSNHKELKTDSSPNQARAQAALQAVNSVQSG<br>NLALAASAAAVDAGMAMAGQSPVLRIIVENLFYPVTLDV<br>LHQIFSKFGTVLKIITFTKNNQFQALLQYADPVSAQHAKLS<br>LDGQNIYNACCTLRIDFSKLTSLNVKYNNDKSRDYTRPDL<br>PSGDSQPSLDQTMAAAFGAPGIISASPYAGAGFPPTFAIP<br>QAAGLSVPNVHGALAPLAIPSAAAAAAAAGRIAIPGLAGA<br>GN | GCCACCATGGACGGCATTGTCCCAGATATAGCCGTTGGTACAAAGCGGGGATCTGACGAGCTTTTCTCTACTTGTGTCA<br>CTAACGGACCGTTTATCATGAGCAGCAACTCGGCTTCTGCAGCAAACGGAAATGACAGCAAGAAGTTCAAAGGTGAC<br>AGCCGAAGTGCAGGCGTCCCCTCTAGAGTGATCCACATCCGGAAGCTCCCCATCGACGTCACGGAGGGGGAAGTCAT<br>CTCCCTGGGGCTGCCCTTTGGGAAGGTCACCAACCTCCTGATGCTGAAGGGGAAAAACCAGGCCTTCATCGAGATGA<br>ACACGGAGGAGGCTGCCAACACCATGGTGAACTACTACACCTCGGTGACCCCTGTGCTGCGCGGCCAGCCCATCTAC<br>ATCCAGTTCTCCAACCACAAGGAGCTGAAGACCGACAGCTCTCCCAACCAGGCGCGGGCCCAGGCGGCCCTGCAGG<br>CGGTGAACTCGGTCCAGTCGGGGAACCTGGCCTTGGCTGCCTCGGCGGCGGCCGTGGACGCAGGGATGGCGATGGC<br>CGGGCAGAGCCCCGTGCTCAGGATCATCGTGGAGAACCTCTTCTACCCTGTGACCCTGGATGTGCTGCACCAGATTTT<br>CTCCAAGTTCGGCACAGTGTTGAAGATCATCACCTTCACCAAGAACAACCAGTTCCAGGCCCTGCTGCAGTATGCGGA<br>CCCCGTGAGCGCCCAGCACGCCAAGCTGTCGCTGGACGGGCAGAACATCTACAACGCCTGCTGCACGCTGCGCATC<br>GACTTTTCCAAGCTCACCAGCCTCAACGTCAAGTACAACAATGACAAGAGCCGTGACTACACACGCCCAGACCTGCCT<br>TCCGGGGACAGCCAGCCCTCGCTGGACCAGACCATGGCCGCGGCCTTCGGTGCACCTGGTATAATCTCAGCCTCTCC<br>GTATGCAGGAGCTGGTTTCCCTCCCACCTTTGCCATTCCTCAAGCTGCAGGCCTTTCCGTTCCGAACGTCCACGGCGCC<br>CTGGCCCCCCTGGCCATCCCCTCGGCGGCGGCGGCAGCTGCGGCGGCAGGTCGGATCGCCATCCCGGGCCTGGCGG<br>GGGCAGGAAAT |
| PTBP1-j (RRM1-RRM2) | RVIHIRKLPIDVTEGEVISLGLPFGKVTNLLMLKGKNQAFIE<br>MNTEEAANTMVNYYTSVTPVLRGQPIYIQFSNHKELKTDS<br>SPNQARAQAALQAVNSVQSGNLALAASAAAVDAGMA<br>MAGQSPVLRIIVENLFYPVTLDVLHQIFSKFGTVLKIITFTKN<br>NQFQALLQYADPVSAQHAKLSLDGQNIYNACCTLRIDFS<br>KLTSLNVKYNNDKSRDYTRPDLPSGDSQPSLDQTMAAAF<br>GAPGIISASPYAGAGFPPTFAIPQAAGLSVPNVHGALAPLA<br>IPSAAAAAAAAGRIAIPGLAGAGN | GCCACCATGAGAGTGATCCACATCCGGAAGCTCCCCATCGACGTCACGGAGGGGGAAGTCATCTCCCTGGGGCTGCC<br>CTTTGGGAAGGTCACCAACCTCCTGATGCTGAAGGGGAAAAACCAGGCCTTCATCGAGATGAACACGGAGGAGGCTG<br>CCAACACCATGGTGAACTACTACACCTCGGTGACCCCTGTGCTGCGCGGCCAGCCCATCTACATCCAGTTCTCCAACC<br>ACAAGGAGCTGAAGACCGACAGCTCTCCCAACCAGGCGCGGGCCCAGGCGGCCCTGCAGGCGGTGAACTCGGTCCA<br>GTCGGGGAACCTGGCCTTGGCTGCCTCGGCGGCGGCCGTGGACGCAGGGATGGCGATGGCCGGGCAGAGCCCCGTG<br>CTCAGGATCATCGTGGAGAACCTCTTCTACCCTGTGACCCTGGATGTGCTGCACCAGATTTTCTCCAAGTTCGGCACAG<br>TGTTGAAGATCATCACCTTCACCAAGAACAACCAGTTCCAGGCCCTGCTGCAGTATGCGGACCCCGTGAGCGCCCAG<br>CACGCCAAGCTGTCGCTGGACGGGCAGAACATCTACAACGCCTGCTGCACGCTGCGCATCGACTTTTCCAAGCTCAC<br>CAGCCTCAACGTCAAGTACAACAATGACAAGAGCCGTGACTACACACGCCCAGACCTGCCTTCCGGGGACAGCCAGC<br>CCTCGCTGGACCAGACCATGGCCGCGGCCTTCGGTGCACCTGGTATAATCTCAGCCTCTCCGTATGCAGGAGCTGGTT<br>TCCCTCCCACCTTTGCCATTCCTCAAGCTGCAGGCCTTTCCGTTCCGAACGTCCACGGCGCCCTGGCCCCCCTGGCCA<br>TCCCCTCGGCGGCGGCGGCAGCTGCGGCGGCAGGTCGGATCGCCATCCCGGGCCTGGCGGGGGCAGGAAAT |
| PTBP1-k (RRM2-RRM3) | QARAQAALQAVNSVQSGNLALAASAAAVDAGMAMAG<br>QSPVLRIIVENLFYPVTLDVLHQIFSKFGTVLKIITFTKNNQF<br>QALLQYADPVSAQHAKLSLDGQNIYNACCTLRIDFSKLTS<br>LNVKYNNDKSRDYTRPDLPSGDSQPSLDQTMAAAFGAP<br>GIISASPYAGAGFPPTFAIPQAAGLSVPNVHGALAPLAIPS<br>AAAAAAAAGRIAIPGLAGAGNSVLLVSNLNPERVTPQSLFI<br>LFGVYGDVQRVKILFNKKENALVQMADGNQAQLAMSHL<br>NGHKLHGKPIRITLSKHQNVQLPREGQEDQGLTKDYGNS<br>PLHRFKKPGSKNFQNIFPPS | GCCACCATGCAGGCGCGGGCCCAGGCGGCCCTGCAGGCGGTGAACTCGGTCCAGTCGGGGAACCTGGCCTTGGCTG<br>CCTCGGCGGCGGCCGTGGACGCAGGGATGGCGATGGCCGGGCAGAGCCCCGTGCTCAGGATCATCGTGGAGAACCT<br>CTTCTACCCTGTGACCCTGGATGTGCTGCACCAGATTTTCTCCAAGTTCGGCACAGTGTTGAAGATCATCACCTTCACC<br>AAGAACAACCAGTTCCAGGCCCTGCTGCAGTATGCGGACCCCGTGAGCGCCCAGCACGCCAAGCTGTCGCTGGACG<br>GGCAGAACATCTACAACGCCTGCTGCACGCTGCGCATCGACTTTTCCAAGCTCACCAGCCTCAACGTCAAGTACAACA<br>ATGACAAGAGCCGTGACTACACACGCCCAGACCTGCCTTCCGGGGACAGCCAGCCCTCGCTGGACCAGACCATGGC<br>CGCGGCCTTCGGTGCACCTGGTATAATCTCAGCCTCTCCGTATGCAGGAGCTGGTTTCCCTCCCACCTTTGCCATTCCT<br>CAAGCTGCAGGCCTTTCCGTTCCGAACGTCCACGGCGCCCTGGCCCCCCTGGCCATCCCCTCGGCGGCGGCGGCAGC<br>TGCGGCGGCAGGTCGGATCGCCATCCCGGGCCTGGCGGGGGCAGGAAATTCTGTATTGCTGGTCAGCAACCTCAACC<br>CAGAGAGAGTCACACCCCAAAGCCTCTTTATTCTTTTCGGCGTCTACGGTGACGTGCAGCGCGTGAAGATCCTGTTCAA<br>TAAGAAGGAGAACGCCCTAGTGCAGATGGCGGACGGCAACCAGGCCCAGCTGGCCATGAGCCACCTGAACGGGCAC<br>AAGCTGCACGGGAAGCCCATCCGCATCACGCTCTCGAAGCACCAGAACGTGCAGCTGCCCCGCGAGGGCCAGGAGG<br>ACCAGGGCCTGACCAAGGACTACGGCAACTCACCCCTGCACCGCTTCAAGAAGCCGGGCTCCAAGAACTTCCAGAAC<br>ATATTCCCGCCCTCG |
| PTBP1-l (RRM3-RRM4) | TSLNVKYNNDKSRDYTRPDLPSGDSQPSLDQTMAAAFG<br>APGIISASPYAGAGFPPTFAIPQAAGLSVPNVHGALAPLAI<br>PSAAAAAAAAGRIAIPGLAGAGNSVLLVSNLNPERVTPQS<br>LFILFGVYGDVQRVKILFNKKENALVQMADGNQAQLAMS<br>HLNGHKLHGKPIRITLSKHQNVQLPREGQEDQGLTKDYG<br>NSPLHRFKKPGSKNFQNIFPPSATLHLSNIPPSVSEEDLKVL<br>FSSNGGVVKGFKFFQKDRKMALIQMGSVEEAVQALIDLH<br>NHDLGENHHLRVSFSKSTI | GCCACCATGACCAGCCTCAACGTCAAGTACAACAATGACAAGAGCCGTGACTACACACGCCCAGACCTGCCTTCCGG<br>GGACAGCCAGCCCTCGCTGGACCAGACCATGGCCGCGGCCTTCGGTGCACCTGGTATAATCTCAGCCTCTCCGTATG<br>CAGGAGCTGGTTTCCCTCCCACCTTTGCCATTCCTCAAGCTGCAGGCCTTTCCGTTCCGAACGTCCACGGCGCCCTGG<br>CCCCCCTGGCCATCCCCTCGGCGGCGGCGGCAGCTGCGGCGGCAGGTCGGATCGCCATCCCGGGCCTGGCGGGGGC<br>AGGAAATTCTGTATTGCTGGTCAGCAACCTCAACCCAGAGAGAGTCACACCCCAAAGCCTCTTTATTCTTTTCGGCGTC<br>TACGGTGACGTGCAGCGCGTGAAGATCCTGTTCAATAAGAAGGAGAACGCCCTAGTGCAGATGGCGGACGGCAACCA<br>GGCCCAGCTGGCCATGAGCCACCTGAACGGGCACAAGCTGCACGGGAAGCCCATCCGCATCACGCTCTCGAAGCAC<br>CAGAACGTGCAGCTGCCCCGCGAGGGCCAGGAGGACCAGGGCCTGACCAAGGACTACGGCAACTCACCCCTGCACC<br>GCTTCAAGAAGCCGGGCTCCAAGAACTTCCAGAACATATTCCCGCCCTCGGCCACGCTGCACCTCTCCAACATCCCGC<br>CCTCAGTCTCCGAGGAGGATCTCAAGGTCCTGTTTTCCAGCAATGGGGGCGTCGTCAAAGGATTCAAGTTCTTCCAGA<br>AGGACCGCAAGATGGCACTGATCCAGATGGGCTCCGTGGAGGAGGCGGTCCAGGCCCTCATTGACCTGCACAACCAC<br>GACCTCGGGGAGAACCACCACCTGCGGGTCTCCTTCTCCAAGTCCACCATC |
