# Supplementary material for: Circular RNA FCHO2 promotes airway remodeling in COPD via regulating nuclear translocation of PTBP1 to repress the splicing of GRN pre-mRNA
Source: Cell Death Dis. 2025 Nov 3;16(1):779. doi: 10.1038/s41419-025-08107-9 (PMC12583663; doi:10.1038/s41419-025-08107-9)
Supplement: Supplementary file 3 — Supplementary table2 [file 41419_2025_8107_MOESM3_ESM.pdf]

GSE198740

name	chrom	start	end	strand	Normal_1	Normal_2	Normal_3	COPD_1	COPD_2	COPD_3	log2FoldCl	pval	padj	Expression
circFCHO2(Ex.17-21)	chr5	72354259	72373320	+	8	10	0	40	23	6	2.496277	0.043762	0.948861	Up
circPTPN22(Ex.4-7)	chr1	1.14E+08	1.14E+08	-	9	23	0	16	65	80	2.802184	0.016542	0.948861	Up
circIRAK3(Ex.2-6)	chr12	66597490	66611015	+	7	5	4	11	8	32	2.219391	0.041237	0.948861	Up
circSLC39A8(Ex.6-9)	chr4	1.03E+08	1.03E+08	-	4	0	0	7	15	10	3.584428	0.018196	0.948861	Up
circTBC1D22A(Ex.10-12)	chr22	47370185	47433094	+	9	0	0	32	7	18	3.215093	0.022306	0.948861	Up
circMBOAT2(Ex.10-12)	chr2	9048750	9098771	-	0	3	4	7	7	16	2.744996	0.043685	0.948861	Up
circACADM(Ex.2-5)	chr1	76198328	76200556	+	0	0	3	4	9	18	4.10629	0.010477	0.948861	Up
circCKAP5(Ex.37-40)	chr11	46829580	46832728	-	0	0	3	7	6	6	3.457791	0.045173	0.948861	Up
circRNF213(Ex.19-21)	chr17	78298829	78302277	+	8	3	8	0	0	0	-4.44225	0.042114	0.948861	Down
circBNC2(Ex.5-6)	chr9	16727794	16738483	-	6	5	8	0	0	0	-4.45075	0.039899	0.948861	Down
circMALT1(Ex.3-4)	chr18	56363597	56367823	+	10	3	11	0	0	0	-4.76419	0.021483	0.948861	Down
circMYBL1(Ex.5-6)	chr8	67484716	67485741	-	4	9	10	0	0	0	-4.73219	0.022294	0.948861	Down
circRNF34(Ex.2-3)	chr12	1.22E+08	1.22E+08	+	6	4	8	0	0	0	-4.36183	0.048116	0.948861	Down
circRBM5(Ex.4-10)	chr3	50131152	50143142	+	5	10	2	0	0	0	-4.41038	0.048835	0.948861	Down
circKIF11(Ex.11-12)	chr10	94388564	94390121	+	7	8	9	0	0	0	-4.80829	0.0178	0.948861	Down
circOXCT1(Ex.8-11)	chr5	41801124	41807540	-	12	4	2	0	0	0	-4.46752	0.044894	0.948861	Down
circNDC1(Ex.11-13)	chr1	54262362	54266521	-	6	4	15	0	0	0	-4.7782	0.020573	0.948861	Down
circELP4(Ex.6-9)	chr11	31648657	31671769	+	7	4	7	0	0	0	-4.37991	0.046501	0.948861	Down
circCCDC150(Ex.3-13)	chr2	1.98E+08	1.98E+08	+	45	18	24	2	3	0	-3.46488	0.005683	0.948861	Down
circCLK3(Ex.5-7)	chr15	74914835	74918309	+	4	9	6	0	0	0	-4.50107	0.03721	0.948861	Down
circFIRRM(Ex.3-14)	chr1	1.7E+08	1.7E+08	+	7	6	5	0	0	0	-4.42406	0.0426	0.948861	Down
circCCDC88B(Ex.2-3)	chr11	64108079	64108490	+	34	38	40	8	5	4	-2.0778	0.030382	0.948861	Down
circZNF202(Ex.4-7)	chr11	1.24E+08	1.24E+08	-	13	6	3	0	0	0	-4.75324	0.023479	0.948861	Down
circBTAF1(Ex.23-26)	chr10	93754292	93768032	+	7	12	2	0	0	0	-4.72117	0.026526	0.948861	Down
circGTF2H2(Ex.2-8)	chr5	70351179	70358623	-	9	5	3	0	0	0	-4.37016	0.049896	0.948861	Down
circTTF1(Ex.3-4)	chr9	1.35E+08	1.35E+08	-	15	20	16	0	3	0	-3.42726	0.023174	0.948861	Down
circTMEM91(Ex.2-3)	chr19	41884186	41888826	+	8	12	5	0	0	0	-4.93385	0.014125	0.948861	Down
circZNF410(Ex.6-7)	chr14	74370663	74371786	+	9	6	9	0	0	0	-4.80165	0.018204	0.948861	Down
circFBXO9(Ex.3-8)	chr6	52938279	52957345	+	25	7	5	2	0	0	-3.61726	0.038486	0.948861	Down
circCEP350(Ex.6-6)	chr1	1.8E+08	1.8E+08	+	5	5	14	0	0	0	-4.72915	0.022656	0.948861	Down
circSLC14A1(Ex.5-9)	chr18	43314239	43328390	+	80	89	0	0	0	0	-7.76197	0.047026	0.948861	Down
circRNF123(Ex.16-19)	chr3	49738924	49739838	+	7	4	7	0	0	0	-4.37991	0.046501	0.948861	Down
circANKLE2(Ex.2-5)	chr12	1.33E+08	1.33E+08	-	10	7	6	0	0	0	-4.78092	0.019587	0.948861	Down
circCENPK(Ex.3-5)	chr5	64847391	64850773	-	8	6	3	0	0	0	-4.37461	0.049022	0.948861	Down
circTRAPPC11(Ex.17-18)	chr4	1.85E+08	1.85E+08	+	14	8	4	0	0	0	-4.99166	0.012876	0.948861	Down
circTAF1(Ex.9-14)	chrX	70601593	70604899	+	12	5	2	0	0	0	-4.55046	0.037809	0.948861	Down
circPTPRC(Ex.12-14)	chr1	1.99E+08	1.99E+08	+	10	9	8	0	0	0	-5.0034	0.010996	0.948861	Down
circANLN(Ex.12-18)	chr7	36460206	36465349	+	20	9	3	0	0	0	-5.30824	0.00653	0.948861	Down
circSEMA4B(Ex.2-7)	chr15	90760671	90764997	+	5	11	2	0	0	0	-4.49665	0.04185	0.948861	Down
circWNK1(Ex.11-12)	chr12	988739	990165	+	15	8	5	0	0	0	-5.08928	0.00979	0.948861	Down
circPTCD3(Ex.6-9)	chr2	86344178	86350885	+	10	13	7	0	0	0	-5.18311	0.007082	0.948861	Down
circRBM5(Ex.5-12)	chr3	50137415	50145002	+	15	4	2	0	0	0	-4.69267	0.029733	0.948861	Down
circECT2(Ex.7-14)	chr3	1.72E+08	1.72E+08	+	41	19	15	5	0	2	-2.84106	0.02419	0.948861	Down
circWNK1(Ex.5-8)	chr12	966327	971436	+	11	4	3	0	0	0	-4.45029	0.043969	0.948861	Down

circNGLY1(Ex.3-4)	chr3	25792589	25805802	-	11	12	12	2	0	0	-3.4843	0.040809	0.948861	Down
circSEPHS1(Ex.2-5)	chr10	13375817	13387028	-	8	3	8	0	0	0	-4.44225	0.042114	0.948861	Down
circFRYL(Ex.8-11)	chr4	48607758	48611840	-	23	10	40	0	5	0	-3.0963	0.028507	0.948861	Down
circUSP24(Ex.60-63)	chr1	55541130	55545332	-	10	16	52	0	6	0	-2.88775	0.042309	0.948861	Down
circANLN(Ex.8-12)	chr7	36455367	36460353	+	9	9	9	0	0	0	-4.9916	0.01122	0.948861	Down
circESCO1(Ex.8-9)	chr18	19119881	19140951	-	216	101	77	34	18	18	-1.89947	0.026237	0.948861	Down
circEYA3(Ex.3-10)	chr1	28337458	28374937	-	17	13	5	0	0	0	-5.42918	0.003966	0.948861	Down
circMTREX(Ex.8-11)	chr5	54639159	54642972	+	11	9	7	0	0	0	-5.01511	0.010865	0.948861	Down
circNUP98(Ex.20-22)	chr11	3726430	3733958	-	22	20	34	5	0	4	-2.43909	0.040094	0.948861	Down
circVPS33A(Ex.9-11)	chr12	1.23E+08	1.23E+08	-	22	23	9	0	0	4	-3.2799	0.02722	0.948861	Down
circTP53BP1(Ex.5-11)	chr15	43762056	43773220	-	8	7	5	0	0	0	-4.58613	0.030535	0.948861	Down
circSMAD2(Ex.2-3)	chr18	45396846	45423180	-	0	0	8	6	8	22	2.954589	0.046664	0.948861	Up
circFAM228B(Ex.6-8)	chr2	24369618	24384483	+	0	0	2	3	2	14	3.944102	0.038748	0.948861	Up
circSLC11A1(Ex.4-8)	chr2	2.19E+08	2.19E+08	+	0	0	0	2	2	6	4.763439	0.043342	0.948861	Up
circITPR1(Ex.50-52)	chr3	4824303	4829823	+	0	0	0	5	6	0	5.016636	0.040626	0.948861	Up
circHERC1(Ex.12-13)	chr15	64026923	64039282	-	0	0	0	6	6	2	5.335416	0.011648	0.948861	Up
circUSP25(Ex.10-18)	chr21	17191017	17214859	+	0	0	0	7	0	4	4.931343	0.047587	0.948861	Up
circLIN9(Ex.2-9)	chr1	2.26E+08	2.26E+08	-	0	0	0	21	9	4	6.613387	2.22E-04	0.948861	Up
circDENND4A(Ex.3-12)	chr15	66015186	66048810	-	0	0	0	3	2	6	4.909354	0.031373	0.948861	Up
circAATF(Ex.5-8)	chr17	35343916	35348156	+	0	0	0	2	7	6	5.394306	0.00993	0.948861	Up
circDDX59(Ex.2-2)	chr1	2.01E+08	2.01E+08	-	0	0	0	3	4	2	4.68539	0.049088	0.948861	Up
circSP100(Ex.25-28)	chr2	2.31E+08	2.31E+08	+	0	0	0	51	0	30	7.809196	0.045805	0.948861	Up
circARHGAP26(Ex.7-11)	chr5	1.42E+08	1.42E+08	+	0	0	0	8	0	4	5.062026	0.037489	0.948861	Up
circNUP98(Ex.29-30)	chr11	3704430	3707424	-	0	0	0	4	3	4	4.94409	0.027808	0.948861	Up
circNBEAL1(Ex.41-43)	chr2	2.04E+08	2.04E+08	+	0	0	0	3	5	4	5.08113	0.020412	0.948861	Up
circPRUNE1(Ex.4-6)	chr1	1.51E+08	1.51E+08	+	0	0	0	11	6	16	6.503893	2.31E-04	0.948861	Up
circATG2B(Ex.26-29)	chr14	96775790	96778496	-	0	0	0	2	5	4	4.952295	0.028311	0.948861	Up
circCEP350(Ex.7-9)	chr1	1.8E+08	1.8E+08	+	0	0	0	4	3	20	6.165697	0.001207	0.948861	Up
circBCAT1(Ex.8-10)	chr12	24982757	24989530	-	0	0	0	8	4	0	5.132915	0.032969	0.948861	Up
circRBSN(Ex.3-4)	chr3	15137480	15138217	-	0	0	0	7	2	2	4.970028	0.029149	0.948861	Up
circTRAK2(Ex.3-14)	chr2	2.02E+08	2.02E+08	-	0	0	0	3	3	4	4.80179	0.037939	0.948861	Up
circWASHC4(Ex.20-23)	chr12	1.06E+08	1.06E+08	+	0	0	0	5	0	6	4.899931	0.049791	0.948861	Up
circAMBRA1(Ex.9-14)	chr11	46455024	46529920	-	0	0	0	0	7	4	4.960826	0.045207	0.948861	Up

GSE221812

name	chrom	start	end	strand	Normal_1	Normal_2	Normal_3	COPD_1	COPD_2	COPD_3	log2FoldCl	pval	padj	Expression
circFCHO2(Ex.17-21)	chr5	72354259	72373320	+	0	3	0	25	13	5	4.584222	0.002802	0.97594	Up
circPTPN22(Ex.4-7)	chr1	1.14E+08	1.14E+08	-	0	2	0	0	10	27	4.704552	0.006167	0.97594	Up
circIRAK3(Ex.2-6)	chr12	66597490	66611015	+	0	0	0	37	15	35	3.753093	0.006185	0.97594	Up
circSLC39A8(Ex.6-9)	chr4	1.03E+08	1.03E+08	-	0	0	0	10	21	22	3.733849	0.007613	0.97594	Up
circTBC1D22A(Ex.10-12)	chr22	47370185	47433094	+	0	0	0	21	9	19	4.079738	0.007473	0.97594	Up
circMBOAT2(Ex.10-12)	chr2	9048750	9098771	-	0	0	0	7	14	29	4.502695	0.003133	0.97594	Up
circACADM(Ex.2-5)	chr1	76198328	76200556	+	0	0	0	0	0	6	5.341655	0.007643	0.97594	Up
circCKAP5(Ex.37-40)	chr11	46829580	46832728	-	0	0	0	16	7	12	5.040807	0.002117	0.97594	Up
circRNF213(Ex.19-21)	chr17	78298829	78302277	+	7	16	0	0	0	0	-6.21051	0.00162	0.97594	Down
circBNC2(Ex.5-6)	chr9	16727794	16738483	-	7	6	17	0	0	0	-5.62177	0.005155	0.97594	Down
circMALT1(Ex.3-4)	chr18	56363597	56367823	+	11	6	28	0	0	0	-5.98174	0.006619	0.97594	Down
circMYBL1(Ex.5-6)	chr8	67484716	67485741	-	21	32	34	0	0	0	-7.30072	1.45E-04	0.97594	Down
circRNF34(Ex.2-3)	chr12	1.22E+08	1.22E+08	+	25	16	0	0	0	0	-6.22185	0.004691	0.97594	Down
circRBM5(Ex.4-10)	chr3	50131152	50143142	+	31	0	22	0	0	0	-6.43924	0.003482	0.97594	Down
circKIF11(Ex.11-12)	chr10	94388564	94390121	+	9	6	0	0	0	0	-6.03163	6.64E-04	0.97594	Down
circHLTF(Ex.7-8)	chr3	1.49E+08	1.49E+08	-	14	0	0	2	0	0	-4.84617	0.03587	0.97594	Down
circDEPDC4(Ex.2-3)	chr12	1.01E+08	1.01E+08	-	18	6	9	3	0	0	-3.03717	0.039038	0.97594	Down
circCHD9(Ex.18-19)	chr16	53288350	53289691	+	13	0	0	0	0	0	-5.16059	0.041154	0.97594	Down
circARAP2(Ex.17-20)	chr4	36134848	36150129	-	18	0	0	0	0	0	-5.56648	0.023931	0.97594	Down
circATP6V0A1(Ex.9-12)	chr17	40635056	40646491	+	15	0	0	0	0	0	-5.18576	0.039434	0.97594	Down
circVPS8(Ex.37-45)	chr3	1.85E+08	1.85E+08	+	14	0	15	0	0	0	-5.45312	0.027682	0.97594	Down
circLMBRD1(Ex.8-14)	chr6	70407455	70428973	-	4	0	0	0	0	0	-4.96387	0.032194	0.97594	Down
circSENP6(Ex.2-5)	chr6	76331248	76344527	+	24	5	0	0	0	0	-5.66983	0.021999	0.97594	Down
circOAT(Ex.2-7)	chr10	1.26E+08	1.26E+08	-	16	0	9	0	0	0	-5.32363	0.033101	0.97594	Down
circRBM26(Ex.4-6)	chr13	79942865	79946065	-	7	7	0	0	0	0	-4.72303	0.046803	0.97594	Down
circMAP4(Ex.2-3)	chr3	48019355	48040369	-	16	0	0	0	0	0	-5.30286	0.034673	0.97594	Down
circAGFG1(Ex.11-12)	chr2	2.28E+08	2.28E+08	+	15	0	0	0	0	0	-5.26764	0.035336	0.97594	Down
circATRIP(Ex.2-4)	chr3	48491443	48495818	+	16	0	0	0	0	0	-5.63741	0.011659	0.97594	Down
circSUPT20H(Ex.2-24)	chr13	37586329	37625720	-	22	0	0	0	0	0	-5.65566	0.022231	0.97594	Down
circIFRD1(Ex.7-9)	chr7	1.12E+08	1.12E+08	+	9	0	13	0	0	0	-5.68697	0.010355	0.97594	Down
circCDC73(Ex.5-10)	chr1	1.93E+08	1.93E+08	+	16	0	0	0	0	0	-5.41334	0.029224	0.97594	Down
circGOLM2(Ex.3-8)	chr15	44620883	44673174	+	14	13	0	0	0	0	-6.21633	0.004482	0.97594	Down
circTBL1XR1(Ex.2-15)	chr3	1.77E+08	1.77E+08	-	17	0	0	0	0	0	-5.09741	0.045755	0.97594	Down
circABCC1(Ex.27-29)	chr16	16225646	16230501	+	12	2	25	0	0	0	-5.90774	0.003641	0.97594	Down
circWASHC4(Ex.28-30)	chr12	1.06E+08	1.06E+08	+	10	2	0	0	0	0	-5.11546	0.02648	0.97594	Down
circANK1(Ex.18-29)	chr8	41551416	41563759	-	32	5	0	0	0	0	-6.10534	0.00645	0.97594	Down
circEIF2AK1(Ex.8-9)	chr7	6080523	6082624	-	12	0	0	93	7	7	3.169127	0.047124	0.97594	Up
circMTA2(Ex.14-14)	chr11	62362736	62362964	-	21	0	0	0	0	0	-5.5283	0.025797	0.97594	Down
circTP53BP2(Ex.5-6)	chr1	2.24E+08	2.24E+08	-	38	0	0	3	0	0	-4.9641	0.032237	0.97594	Down
circMTMR8(Ex.3-4)	chrX	63574657	63576218	-	12	3	0	0	0	0	-5.19296	0.023892	0.97594	Down
circUBE2G1(Ex.3-5)	chr17	4186093	4200109	-	7	5	0	0	0	0	-4.81224	0.03936	0.97594	Down
circCDK5RAP2(Ex.12-13)	chr9	1.23E+08	1.23E+08	-	12	0	0	0	0	0	-5.20016	0.022872	0.97594	Down
circTULP4(Ex.6-8)	chr6	1.59E+08	1.59E+08	+	22	4	13	0	0	3	-3.77196	0.03309	0.97594	Down
circSLC38A10(Ex.3-10)	chr17	79244718	79258695	-	15	0	0	0	0	0	-5.3505	0.031658	0.97594	Down
circTACC1(Ex.5-11)	chr8	38684686	38700913	+	4	0	12	0	0	0	-4.79657	0.040426	0.97594	Down

circESCO2(Ex.5-9)	chr8	27641517	27650328	+	18	0	0	0	0	0	-5.73133	0.010001	0.97594	Down
circNEK4(Ex.9-15)	chr3	52771602	52780920	-	11	5	10	0	0	0	-5.42663	0.00798	0.97594	Down
circZMAT4(Ex.5-6)	chr8	40438684	40532450	-	16	10	0	0	0	0	-5.6912	0.005352	0.97594	Down
circSLC30A9(Ex.2-17)	chr4	42003633	42080342	+	18	8	16	0	0	0	-6.4035	2.54E-04	0.97594	Down
circSLC4A1(Ex.13-13)	chr17	42334718	42334912	-	12	2	0	0	0	0	-4.89877	0.03809	0.97594	Down
circCUL2(Ex.5-12)	chr10	35321363	35343467	-	7	0	17	0	0	0	-5.06936	0.045413	0.97594	Down
circZNF277(Ex.2-4)	chr7	1.12E+08	1.12E+08	+	9	0	17	0	0	0	-5.21718	0.037561	0.97594	Down
circNEMF(Ex.17-22)	chr14	50269171	50281575	-	12	0	0	0	0	0	-5.14384	0.041095	0.97594	Down
circSTRN3(Ex.8-10)	chr14	31382730	31398517	-	24	0	0	0	0	0	-4.69647	0.045661	0.97594	Down
circIPO11(Ex.25-28)	chr5	61846194	61887499	+	5	0	0	0	0	0	-5.43017	0.02917	0.97594	Down
circCEP43(Ex.10-12)	chr6	1.67E+08	1.67E+08	+	2	0	0	0	0	26	4.218968	0.029152	0.97594	Up
circKIF14(Ex.11-12)	chr1	2.01E+08	2.01E+08	-	10	0	0	0	0	0	-5.00283	0.049835	0.97594	Down
circSTRN3(Ex.2-9)	chr14	31388172	31425448	-	24	3	30	0	0	0	-6.52096	0.001161	0.97594	Down
circTIAM1(Ex.7-8)	chr21	32595722	32598266	-	23	4	0	2	0	0	-4.71046	0.028805	0.97594	Down
circINTS8(Ex.15-17)	chr8	95869005	95877918	+	11	3	0	0	0	0	-5.01706	0.030145	0.97594	Down
circTRRAP(Ex.13-14)	chr7	98503800	98506585	+	11	5	12	0	0	0	-5.39326	0.016624	0.97594	Down
circJMJD1C(Ex.12-14)	chr10	64954046	64958487	-	25	4	0	0	0	0	-5.67951	0.022004	0.97594	Down
circMYO1F(Ex.13-14)	chr19	8609181	8610620	-	4	9	29	0	0	2	-3.73613	0.034054	0.97594	Down
circUBA2(Ex.9-12)	chr19	34941170	34945461	+	22	0	15	0	0	0	-5.86674	0.016155	0.97594	Down
circABCB7(Ex.10-15)	chrX	74280058	74290357	-	3	2	0	19	7	17	3.217558	0.048167	0.97594	Up
circLYPLA1(Ex.3-4)	chr8	54975888	54978373	-	5	0	0	0	0	0	-5.08758	0.027019	0.97594	Down
circCREBRF(Ex.2-4)	chr5	1.73E+08	1.73E+08	+	18	12	0	0	0	0	-6.49807	2.55E-04	0.97594	Down
circUBTF(Ex.3-5)	chr17	42293022	42294072	-	11	7	0	0	0	0	-4.78251	0.02318	0.97594	Down
circTTLL3(Ex.2-4)	chr3	9852305	9855029	+	14	4	10	0	0	0	-5.62311	0.005466	0.97594	Down
circEXOC6(Ex.5-8)	chr10	94659356	94679807	+	16	0	0	0	0	0	-5.16828	0.041573	0.97594	Down
circCPLANE1(Ex.37-47)	chr5	37125346	37164429	-	9	0	0	0	0	0	-5.27221	0.019971	0.97594	Down
circTNPO1(Ex.12-15)	chr5	72182897	72187723	+	4	2	0	0	2	0	-4.39196	0.043548	0.97594	Down
circAP3B1(Ex.23-24)	chr5	77330185	77335098	-	7	13	0	0	0	0	-5.35982	0.017263	0.97594	Down
circGXYLT1(Ex.3-7)	chr12	42491244	42512973	-	15	0	0	0	0	0	-5.22433	0.037429	0.97594	Down
circTFE3(Ex.2-9)	chrX	48888912	48898095	-	6	9	24	0	0	0	-6.18993	5.76E-04	0.97594	Down
circHEXB(Ex.6-9)	chr5	74001044	74012498	+	14	0	0	0	0	0	-5.24206	0.036393	0.97594	Down
circLRCH1(Ex.14-19)	chr13	47285926	47308133	+	5	0	0	31	19	24	2.692542	0.039443	0.97594	Up
circGFPT1(Ex.10-15)	chr2	69565030	69577315	-	11	6	0	0	0	0	-3.79944	0.049811	0.97594	Down
circVPS13A(Ex.17-24)	chr9	79843038	79888280	+	15	0	0	0	0	0	-5.04081	0.047913	0.97594	Down
circRNF19B(Ex.5-8)	chr1	33403998	33411229	-	13	0	0	0	0	0	-4.92712	0.036569	0.97594	Down
circDNM1L(Ex.5-9)	chr12	32863863	32875567	+	4	2	0	0	0	0	-5.03767	0.031049	0.97594	Down
circTNS3(Ex.21-24)	chr7	47336680	47344601	-	98	46	118	0	0	0	-4.3371	0.006682	0.97594	Down
circFLACC1(Ex.2-9)	chr2	2.02E+08	2.02E+08	-	13	7	0	0	0	0	-5.0807	0.04526	0.97594	Down
circOPA1(Ex.2-4)	chr3	1.93E+08	1.93E+08	+	32	0	0	0	0	0	-4.11593	0.039129	0.97594	Down
circPDS5A(Ex.8-16)	chr4	39900009	39918808	-	8	3	0	0	0	0	-5.3816	0.00894	0.97594	Down
circLTBP1(Ex.4-5)	chr2	33335649	33360027	+	6	2	0	0	0	0	-5.70881	0.011746	0.97594	Down
circPICALM(Ex.10-12)	chr11	85707869	85712201	-	21	0	8	0	0	0	-5.58225	0.023955	0.97594	Down
circARFIP1(Ex.4-8)	chr4	1.54E+08	1.54E+08	+	10	0	0	0	0	0	-5.47152	0.027564	0.97594	Down
circHLTF(Ex.3-4)	chr3	1.49E+08	1.49E+08	-	9	0	0	0	0	0	-5.25709	0.035607	0.97594	Down
circCEP44(Ex.5-10)	chr4	1.75E+08	1.75E+08	+	12	6	13	0	0	0	-5.54073	0.013124	0.97594	Down
circHSF5(Ex.2-5)	chr17	56536129	56557628	-	5	0	12	0	0	0	-4.77003	0.043731	0.97594	Down

circAKAP13(Ex.16-19)	chr15	86227972	86253876	+	10	0	18	0	0	0	-6.12728	0.001748	0.97594	Down
circNUP153(Ex.6-8)	chr6	17669206	17669777	-	2	0	0	16	3	15	3.966244	0.042924	0.97594	Up
circPKD2(Ex.8-10)	chr4	88977238	88983156	+	9	0	0	0	0	0	-5.00001	0.04997	0.97594	Down
circOXCT1(Ex.8-11)	chr5	41801124	41807540	-	10	3	0	0	0	0	-4.81986	0.040214	0.97594	Down
circSTIM2(Ex.9-10)	chr4	27010050	27010624	+	2	0	0	4	14	9	3.880459	0.018655	0.97594	Up
circCLN6(Ex.2-5)	chr15	68503601	68510988	-	11	0	10	0	0	0	-5.00898	0.048843	0.97594	Down
circTMCO4(Ex.3-9)	chr1	20072949	20113610	-	2	6	0	0	0	0	-5.98294	0.007895	0.97594	Down
circGMFG(Ex.5-6)	chr19	39819640	39820266	-	9	5	0	0	0	0	-5.03887	0.028322	0.97594	Down
circZNF185(Ex.19-23)	chrX	1.52E+08	1.52E+08	+	10	0	0	0	0	0	-5.32387	0.032646	0.97594	Down
circFANCA(Ex.16-18)	chr16	89846277	89849510	-	14	0	15	0	0	0	-5.93818	0.006922	0.97594	Down
circZDHHC20(Ex.4-5)	chr13	21976936	21987911	-	11	3	17	0	0	0	-5.49945	0.014604	0.97594	Down
circNUP107(Ex.6-10)	chr12	69090599	69103873	+	17	0	0	0	0	0	-5.192	0.039861	0.97594	Down
circARMH3(Ex.5-13)	chr10	1.04E+08	1.04E+08	-	19	0	10	0	0	0	-5.54646	0.024842	0.97594	Down
circHNRNPAB(Ex.3-5)	chr5	1.78E+08	1.78E+08	+	13	9	0	0	0	0	-5.2017	0.038721	0.97594	Down
circSYNE1(Ex.4-10)	chr6	1.53E+08	1.53E+08	-	8	2	0	0	0	0	-4.48339	0.037437	0.97594	Down
circMAP4K3(Ex.9-12)	chr2	39552659	39553418	-	6	5	0	0	0	0	-4.09213	0.039328	0.97594	Down
circUIMC1(Ex.2-5)	chr5	1.76E+08	1.76E+08	-	12	12	0	0	0	0	-5.30095	0.034008	0.97594	Down
circUXS1(Ex.5-10)	chr2	1.07E+08	1.07E+08	-	21	0	19	0	0	0	-6.32128	0.001398	0.97594	Down
circAP4E1(Ex.16-20)	chr15	51276219	51293380	+	6	3	0	0	0	0	-5.43406	0.016862	0.97594	Down
circTENT2(Ex.4-12)	chr5	78919075	78964851	+	6	17	9	7	0	0	-3.39163	0.017747	0.97594	Down
circMAP4K3(Ex.23-26)	chr2	39499424	39507497	-	10	0	14	0	0	0	-5.1423	0.041246	0.97594	Down
circHMGCR(Ex.3-4)	chr5	74639678	74640157	+	10	33	0	2	0	0	-4.05206	0.040842	0.97594	Down
circXPO1(Ex.17-23)	chr2	61709515	61717911	-	8	7	0	0	0	0	-6.23999	4.34E-04	0.97594	Down
circTECTA(Ex.8-10)	chr11	1.21E+08	1.21E+08	+	10	0	13	0	0	0	-5.09107	0.043996	0.97594	Down
circRIOK3(Ex.3-7)	chr18	21043931	21047490	+	11	0	14	0	0	0	-5.21455	0.037635	0.97594	Down
circNIPBL(Ex.2-4)	chr5	36953720	36958333	+	6	15	25	8	0	0	-2.96513	0.041259	0.97594	Down
circGOLIM4(Ex.9-10)	chr3	1.68E+08	1.68E+08	-	10	3	14	0	0	0	-5.54414	0.006291	0.97594	Down
circCARS2(Ex.7-9)	chr13	1.11E+08	1.11E+08	-	24	0	0	0	2	0	-4.72708	0.043141	0.97594	Down
circMS4A3(Ex.2-3)	chr11	59828619	59830078	+	7	4	0	0	0	0	-4.85503	0.037171	0.97594	Down
circIK(Ex.6-9)	chr5	1.4E+08	1.4E+08	+	5	10	8	0	0	0	-5.0709	0.026941	0.97594	Down
circAKAP10(Ex.6-8)	chr17	19843026	19845223	-	9	6	0	2	0	0	-4.57436	0.009658	0.97594	Down
circMYO9B(Ex.8-11)	chr19	17270205	17278874	+	18	0	0	0	0	0	-5.6411	0.011792	0.97594	Down
circPI4KA(Ex.6-7)	chr22	21172771	21174188	-	19	0	8	0	0	0	-5.46826	0.027684	0.97594	Down
circMICAL3(Ex.21-21)	chr22	18314620	18314873	-	9	0	15	0	0	0	-5.11838	0.042534	0.97594	Down
circBMS1(Ex.14-15)	chr10	43312047	43312942	+	0	8	0	0	0	0	-4.85655	0.036119	0.97594	Down
circVPS35L(Ex.25-27)	chr16	19661704	19680621	+	0	11	33	18	68	94	2.462545	0.049089	0.97594	Up
circPIK3R2(Ex.12-14)	chr19	18276970	18279356	+	0	2	15	0	0	0	-4.73637	0.046666	0.97594	Down
circFANCL(Ex.8-13)	chr2	58387243	58393009	-	0	6	25	0	0	0	-5.30661	0.033875	0.97594	Down
circDYSF(Ex.33-42)	chr2	71825694	71871193	+	0	3	0	0	0	0	-5.14974	0.012667	0.97594	Down
circFAM13B(Ex.6-7)	chr5	1.37E+08	1.37E+08	-	0	5	0	0	27	13	3.739538	0.049473	0.97594	Up
circFAM114A1(Ex.9-12)	chr4	38924391	38933995	+	0	3	0	17	3	11	3.402591	0.037959	0.97594	Up
circDIP2A(Ex.6-8)	chr21	47924274	47931527	+	0	6	9	0	0	0	-4.96876	0.030388	0.97594	Down
circCALM2(Ex.2-4)	chr2	47389425	47397903	-	0	3	21	6	4	0	-3.72184	0.021463	0.97594	Down
circTXNDC16(Ex.2-6)	chr14	53003437	53011089	-	0	15	0	0	0	0	-5.0073	0.008453	0.97594	Down
circHOOK3(Ex.4-8)	chr8	42785265	42814457	+	0	18	0	0	0	0	-5.51298	0.013855	0.97594	Down
circNRDC(Ex.14-21)	chr1	52266228	52279761	-	0	7	14	0	0	0	-5.00831	0.029501	0.97594	Down

circGCLC(Ex.6-12)	chr6	53370190	53379135	-	0	3	0	0	0	0	-5.37996	0.01828	0.97594	Down
circADGRE2(Ex.18-20)	chr19	14854232	14857135	-	0	2	5	0	0	0	-5.06714	0.029309	0.97594	Down
circDENND1B(Ex.3-7)	chr1	1.98E+08	1.98E+08	-	0	2	0	2	0	43	4.712326	0.017311	0.97594	Up
circUSP15(Ex.2-7)	chr12	62687960	62743088	+	0	18	0	0	0	0	-4.82383	0.020826	0.97594	Down
circSENP6(Ex.8-16)	chr6	76368978	76388643	+	0	10	0	0	0	0	-5.57118	0.006123	0.97594	Down
circTEX9(Ex.5-10)	chr15	56680670	56704634	+	0	5	0	56	16	0	3.788709	0.048709	0.97594	Up
circAGTPBP1(Ex.10-17)	chr9	88233898	88272558	-	0	4	0	0	0	0	-5.04247	0.01567	0.97594	Down
circPEX1(Ex.4-5)	chr7	92146590	92147569	-	0	7	16	0	0	0	-5.16169	0.023294	0.97594	Down
circMLLT10(Ex.14-16)	chr10	22002701	22016857	+	0	3	25	0	0	0	-5.13607	0.043174	0.97594	Down
circCSNK1A1(Ex.3-6)	chr5	1.49E+08	1.49E+08	-	0	4	0	0	0	0	-5.1003	0.044377	0.97594	Down
circTBC1D22A(Ex.11-12)	chr22	47432967	47507499	+	0	4	0	0	0	0	-5.09147	0.013815	0.97594	Down
circNPEPPS(Ex.13-17)	chr17	45673719	45682918	+	0	2	0	3	0	0	4.250682	0.028549	0.97594	Up
circRHD(Ex.3-6)	chr1	25617132	25629950	+	0	10	18	0	0	0	-5.41481	0.015763	0.97594	Down
circLYST(Ex.9-13)	chr1	2.36E+08	2.36E+08	-	0	5	0	0	0	2	-4.61777	0.015948	0.97594	Down
circERBB2(Ex.3-7)	chr17	37864574	37866734	+	0	10	0	0	0	0	-5.04106	0.046355	0.97594	Down
circDERL1(Ex.3-7)	chr8	1.24E+08	1.24E+08	-	0	2	0	38	0	2	3.451045	0.034234	0.97594	Up
circPDS5B(Ex.25-30)	chr13	33327470	33334858	+	0	5	11	2	0	0	-4.53971	0.019581	0.97594	Down
circKLHL20(Ex.8-10)	chr1	1.74E+08	1.74E+08	+	0	7	0	0	0	0	-5.14218	0.04122	0.97594	Down
circHEATR5B(Ex.19-24)	chr2	37255066	37268435	-	0	4	17	0	0	0	-5.22042	0.012027	0.97594	Down
circLARS1(Ex.7-10)	chr5	1.46E+08	1.46E+08	-	0	3	0	16	0	12	3.677279	0.033272	0.97594	Up
circXPO1(Ex.4-10)	chr2	61724014	61749818	-	0	3	4	0	0	0	-4.84378	0.038603	0.97594	Down
circCOMMD7(Ex.4-8)	chr20	31291825	31294562	-	0	7	0	14	12	18	3.095302	0.025372	0.97594	Up
circABCC4(Ex.26-29)	chr13	95695936	95715113	-	0	5	0	0	0	0	-5.06093	0.027672	0.97594	Down
circNDUFA10(Ex.5-9)	chr2	2.41E+08	2.41E+08	-	0	5	7	0	0	4	-2.915	0.047571	0.97594	Down
circSLC35B3(Ex.6-7)	chr6	8417635	8419910	-	0	7	19	0	0	0	-5.07409	0.044997	0.97594	Down
circDNAAF11(Ex.2-11)	chr8	1.34E+08	1.34E+08	-	0	2	12	0	0	0	-4.74706	0.044026	0.97594	Down
circUBR5(Ex.48-50)	chr8	1.03E+08	1.03E+08	-	0	18	0	0	0	0	-5.25095	0.035792	0.97594	Down
circSNTB1(Ex.3-4)	chr8	1.22E+08	1.22E+08	-	0	12	0	0	0	0	-4.87185	0.037067	0.97594	Down
circZFP91(Ex.2-4)	chr11	58352329	58377807	+	0	9	13	3	0	0	-4.35825	0.022367	0.97594	Down
circSLC43A2(Ex.2-5)	chr17	1516489	1531214	-	0	8	8	0	0	0	-5.00445	0.029548	0.97594	Down
circPDE5A(Ex.10-20)	chr4	1.2E+08	1.2E+08	-	0	7	12	0	0	0	-5.15195	0.023716	0.97594	Down
circCCDC30(Ex.3-6)	chr1	42948334	42964587	+	0	2	0	3	17	3	3.837596	0.021728	0.97594	Up
circUBR3(Ex.2-11)	chr2	1.71E+08	1.71E+08	+	0	4	0	9	49	19	3.010282	0.022105	0.97594	Up
circFRYL(Ex.36-47)	chr4	48541988	48555400	-	0	6	0	0	0	0	-5.12836	0.042165	0.97594	Down
circGPSM2(Ex.2-11)	chr1	1.09E+08	1.09E+08	+	0	9	2	32	0	48	2.941747	0.032014	0.97594	Up
circPABPC1(Ex.8-14)	chr8	1.02E+08	1.02E+08	-	0	7	0	0	0	0	-4.9369	0.033305	0.97594	Down
circFLVCR1(Ex.3-8)	chr1	2.13E+08	2.13E+08	+	0	4	0	7	12	21	2.779793	0.04575	0.97594	Up
circNIT2(Ex.2-6)	chr3	1E+08	1E+08	+	0	11	0	0	0	0	-4.84962	0.0369	0.97594	Down
circZNF106(Ex.8-9)	chr15	42736400	42737179	-	0	3	0	14	3	6	2.896222	0.04915	0.97594	Up
circVCL(Ex.3-12)	chr10	75830428	75855613	+	0	3	0	14	22	0	3.828669	0.03006	0.97594	Up
circDLGAP5(Ex.9-15)	chr14	55621335	55642736	-	0	5	0	35	0	45	3.732611	0.017273	0.97594	Up
circADGRE3(Ex.14-15)	chr19	14736304	14741019	-	0	8	0	0	0	0	-5.03351	0.047742	0.97594	Down
circCA2(Ex.3-6)	chr8	86385922	86389504	+	0	3	9	0	0	0	-4.75012	0.042808	0.97594	Down
circZFAT(Ex.3-3)	chr8	1.36E+08	1.36E+08	-	0	3	0	45	6	0	4.042888	0.036496	0.97594	Up
circMYO9A(Ex.12-14)	chr15	72252242	72270633	-	0	4	0	0	0	0	-4.73326	0.044109	0.97594	Down
circABCD3(Ex.3-7)	chr1	94930331	94941293	+	0	9	25	0	0	0	-6.3908	9.78E-04	0.97594	Down

circDAAM1(Ex.16-19)	chr14	59806792	59820682	+	0	2	0	2	10	7	4.047673	0.041417	0.97594	Up
circUBXN2B(Ex.3-6)	chr8	59343078	59352329	+	0	5	0	0	0	0	-4.80702	0.038918	0.97594	Down
circSLC9A9(Ex.9-12)	chr3	1.43E+08	1.43E+08	-	0	2	0	70	0	0	4.880305	0.02098	0.97594	Up
circZCCHC17(Ex.2-5)	chr1	31782891	31811895	+	0	6	0	0	0	0	-5.19777	0.022124	0.97594	Down
circEFL1(Ex.16-18)	chr15	82443806	82456325	-	0	16	0	0	0	0	-5.13482	0.041411	0.97594	Down
circDENND5B(Ex.2-7)	chr12	31595709	31648853	-	0	7	0	0	0	0	-5.04652	0.046413	0.97594	Down
circMTREX(Ex.8-11)	chr5	54639159	54642972	+	0	5	0	0	0	0	-5.24572	0.020695	0.97594	Down
circMARF1(Ex.11-12)	chr16	15715590	15716980	-	0	4	9	0	0	0	-4.8934	0.034447	0.97594	Down
circTMEM38B(Ex.2-5)	chr9	1.08E+08	1.09E+08	+	0	7	14	0	0	0	-5.00831	0.029501	0.97594	Down
circACAT1(Ex.6-10)	chr11	1.08E+08	1.08E+08	+	0	12	0	0	0	0	-5.09312	0.007033	0.97594	Down
circATP9B(Ex.12-15)	chr18	77013381	77067234	+	0	19	40	0	6	0	-3.86675	0.042521	0.97594	Down
circLRCH3(Ex.2-5)	chr3	1.98E+08	1.98E+08	+	0	4	0	0	0	0	-4.86402	0.039175	0.97594	Down
circEXOSC9(Ex.4-10)	chr4	1.23E+08	1.23E+08	+	0	6	0	32	28	0	4.065086	0.015031	0.97594	Up
circTBC1D8(Ex.16-19)	chr2	1.02E+08	1.02E+08	-	0	9	0	0	0	0	-5.28727	0.01926	0.97594	Down
circCILK1(Ex.2-5)	chr6	52895863	52906206	-	0	13	0	92	23	6	3.21014	0.030759	0.97594	Up
circUSP33(Ex.20-22)	chr1	78167054	78178966	-	0	11	11	0	0	0	-5.64919	0.01041	0.97594	Down
circATG4B(Ex.9-11)	chr2	2.43E+08	2.43E+08	+	0	6	0	0	0	0	-5.33321	0.018287	0.97594	Down
circHERC1(Ex.48-51)	chr15	63946318	63950917	-	0	3	28	0	0	0	-5.27962	0.03619	0.97594	Down
circTCTN2(Ex.9-14)	chr12	1.24E+08	1.24E+08	+	0	4	0	0	0	0	-5.11763	0.0253	0.97594	Down
circLRRK2(Ex.3-8)	chr12	40626076	40643747	+	0	8	0	2	25	15	3.112025	0.04942	0.97594	Up
circATF7IP2(Ex.10-13)	chr16	10565967	10574821	+	0	8	19	0	0	0	-5.2282	0.022442	0.97594	Down
circHOOK3(Ex.8-10)	chr8	42814374	42821756	+	0	16	23	0	0	0	-7.05887	2.60E-04	0.97594	Down
circCCNB1(Ex.5-7)	chr5	68470078	68471364	+	0	15	0	0	0	0	-5.07306	0.044725	0.97594	Down
circHOOK3(Ex.12-15)	chr8	42828432	42841938	+	0	6	0	45	14	7	2.763168	0.030813	0.97594	Up
circKIDINS220(Ex.7-12)	chr2	8933940	8946499	-	0	18	0	0	0	0	-4.70223	0.014584	0.97594	Down
circPPP2R1A(Ex.12-13)	chr19	52724232	52725494	+	0	5	0	0	0	0	-4.65474	0.04906	0.97594	Down
circRAD50(Ex.6-9)	chr5	1.32E+08	1.32E+08	+	0	3	8	0	0	0	-5.07131	0.027102	0.97594	Down
circSERINC1(Ex.3-6)	chr6	1.23E+08	1.23E+08	-	0	6	29	0	0	0	-5.92212	0.007	0.97594	Down
circIBTK(Ex.24-26)	chr6	82891596	82901596	-	0	5	0	14	0	23	3.482742	0.041407	0.97594	Up
circPCCA(Ex.15-19)	chr13	1.01E+08	1.01E+08	+	0	2	0	28	10	0	3.400163	0.032123	0.97594	Up
circCASD1(Ex.14-17)	chr7	94178845	94183887	+	0	4	24	4	0	0	-3.8629	0.04939	0.97594	Down
circPAN3(Ex.15-17)	chr13	28851374	28855516	+	0	12	0	0	0	0	-4.75277	0.045316	0.97594	Down
circFAM20B(Ex.2-3)	chr1	1.79E+08	1.79E+08	+	0	3	0	0	0	14	4.389145	0.022206	0.97594	Up
circTAB3(Ex.2-8)	chrX	30864668	30889918	-	0	0	47	0	0	5	-3.88586	0.046922	0.97594	Down
circEXOC6(Ex.13-14)	chr10	94700484	94708136	+	0	0	21	0	0	0	-5.30823	0.018646	0.97594	Down
circUBR5(Ex.36-49)	chr8	1.03E+08	1.03E+08	-	0	0	11	0	0	0	-5.39387	0.017713	0.97594	Down
circFAM185A(Ex.3-7)	chr7	1.02E+08	1.02E+08	+	0	0	12	52	9	27	2.849726	0.042718	0.97594	Up
circSLC44A1(Ex.4-12)	chr9	1.08E+08	1.08E+08	+	0	0	28	0	0	0	-5.51595	0.025287	0.97594	Down
circBLTP3B(Ex.3-8)	chr12	1E+08	1E+08	-	0	0	10	0	0	0	-5.22547	0.020966	0.97594	Down
circRUFY2(Ex.2-3)	chr10	70161377	70164601	-	0	0	17	0	0	0	-5.50421	0.025824	0.97594	Down
circITGA4(Ex.12-17)	chr2	1.82E+08	1.82E+08	+	0	0	12	0	0	0	-5.57075	0.011962	0.97594	Down
circCLK1(Ex.8-11)	chr2	2.02E+08	2.02E+08	-	0	0	24	0	0	0	-5.57431	0.023058	0.97594	Down
circXRN1(Ex.37-40)	chr3	1.42E+08	1.42E+08	-	0	0	22	0	0	0	-5.08229	0.044647	0.97594	Down
circUSP3(Ex.8-11)	chr15	63855094	63866602	+	0	0	8	0	0	0	-4.7568	0.041731	0.97594	Down
circRANBP2(Ex.21-23)	chr2	1.09E+08	1.09E+08	+	0	0	9	0	0	0	-4.99896	0.029541	0.97594	Down
circPHAX(Ex.2-2)	chr5	1.26E+08	1.26E+08	+	0	0	6	57	16	34	3.081605	0.026683	0.97594	Up

circVPS13B(Ex.23-24)	chr8	1E+08	1E+08	+	0	0	14	0	0	0	-5.14563	0.040249	0.97594	Down
circIFT74(Ex.11-13)	chr9	27016905	27029102	+	0	0	16	0	0	0	-5.07282	0.045057	0.97594	Down
circPRKDC(Ex.51-52)	chr8	48765234	48766775	-	0	0	7	0	0	0	-4.9761	0.030608	0.97594	Down
circPPP2R3C(Ex.3-11)	chr14	35560276	35579835	-	0	0	58	0	0	0	-3.88204	0.046179	0.97594	Down
circSPAG1(Ex.2-5)	chr8	1.01E+08	1.01E+08	+	0	0	19	0	0	0	-5.12609	0.041561	0.97594	Down
circREPS1(Ex.8-19)	chr6	1.39E+08	1.39E+08	-	0	0	20	0	0	0	-5.42511	0.015086	0.97594	Down
circHECTD4(Ex.15-20)	chr12	1.13E+08	1.13E+08	-	0	0	18	0	0	0	-5.06639	0.044767	0.97594	Down
circFAM114A2(Ex.2-8)	chr5	1.53E+08	1.53E+08	-	0	0	20	0	0	0	-5.13214	0.025951	0.97594	Down
circCCNE1(Ex.5-6)	chr19	30308044	30308448	+	0	0	8	0	0	0	-5.45323	0.006625	0.97594	Down
circRPS6KA2(Ex.17-19)	chr6	1.67E+08	1.67E+08	-	0	0	7	0	0	0	-5.3071	0.017788	0.97594	Down
circDCAF17(Ex.4-9)	chr2	1.72E+08	1.72E+08	+	0	0	16	0	0	0	-5.46456	0.027206	0.97594	Down
circDZIP3(Ex.22-26)	chr3	1.08E+08	1.08E+08	+	0	0	27	0	0	0	-5.75962	0.018495	0.97594	Down
circDENND3(Ex.11-13)	chr8	1.42E+08	1.42E+08	+	0	0	16	0	0	0	-5.62686	0.022027	0.97594	Down
circRAB7A(Ex.2-5)	chr3	1.29E+08	1.29E+08	+	0	0	15	0	0	0	-5.31768	0.01866	0.97594	Down
circMAN2A1(Ex.16-17)	chr5	1.09E+08	1.09E+08	+	0	0	2	24	12	0	4.430468	0.010335	0.97594	Up
circWDR26(Ex.11-13)	chr1	2.25E+08	2.25E+08	-	0	0	21	0	0	0	-5.18231	0.039645	0.97594	Down
circUBR4(Ex.35-42)	chr1	19482765	19489105	-	0	0	17	0	0	0	-5.24572	0.035403	0.97594	Down
circSTRN3(Ex.6-9)	chr14	31388172	31405830	-	0	0	20	0	0	0	-5.6754	0.020096	0.97594	Down
circWWOX(Ex.7-8)	chr16	78458767	78466649	+	0	0	5	0	0	0	-5.01971	0.028844	0.97594	Down
circMAP4K5(Ex.6-14)	chr14	50923234	50949134	-	0	0	12	0	0	0	-5.27078	0.034374	0.97594	Down
circKIF13B(Ex.22-23)	chr8	28989825	28991727	-	0	0	15	0	0	0	-4.66609	0.049919	0.97594	Down
circNLRP12(Ex.3-3)	chr19	54312841	54314542	-	0	0	28	0	0	0	-5.61919	0.021887	0.97594	Down
circAPC(Ex.2-8)	chr5	1.12E+08	1.12E+08	+	0	0	29	0	0	0	-5.33152	0.033391	0.97594	Down
circMED17(Ex.2-8)	chr11	93521167	93530885	+	0	0	32	3	0	0	-4.62996	0.027605	0.97594	Down
circCCDC47(Ex.2-9)	chr17	61831793	61843554	-	0	0	23	2	0	0	-4.1049	0.019064	0.97594	Down
circANKRD13C(Ex.6-12)	chr1	70736539	70771973	-	0	0	16	0	0	0	-5.10657	0.042347	0.97594	Down
circZC3H4(Ex.10-11)	chr19	47584770	47585552	-	0	0	0	0	0	0	-5.07301	0.029898	0.97594	Down
circARHGAP8(Ex.4-6)	chr22	45204187	45218349	+	0	0	0	37	17	0	3.531208	0.027648	0.97594	Up
circCKAP5(Ex.6-10)	chr11	46819633	46831424	-	0	0	0	11	4	14	2.834347	0.041453	0.97594	Up
circKNTC1(Ex.9-13)	chr12	1.23E+08	1.23E+08	+	0	0	0	0	0	0	-5.01487	0.029068	0.97594	Down
circNPHP4(Ex.2-11)	chr1	5987709	6046387	-	0	0	0	0	0	0	-5.11312	0.025029	0.97594	Down
circMARCHF6(Ex.20-25)	chr5	10414545	10430140	+	0	0	0	0	0	0	-5.23265	0.036939	0.97594	Down
circCHFR(Ex.7-8)	chr12	1.33E+08	1.33E+08	-	0	0	0	0	0	0	-5.00726	0.048713	0.97594	Down
circZC3H7B(Ex.12-17)	chr22	41738533	41747650	+	0	0	0	26	16	0	3.471175	0.041869	0.97594	Up
circAGTPBP1(Ex.4-7)	chr9	88287465	88296250	-	0	0	0	0	0	0	-5.01843	0.048584	0.97594	Down
circANKRD42(Ex.3-8)	chr11	82917100	82947551	+	0	0	0	0	0	0	-4.84976	0.036467	0.97594	Down
circWRN(Ex.9-18)	chr8	30938383	30958471	+	0	0	0	0	0	0	-5.41772	0.028948	0.97594	Down
circAPC(Ex.12-14)	chr5	1.12E+08	1.12E+08	+	0	0	0	0	0	0	-5.45731	0.028177	0.97594	Down
circFNBP1L(Ex.13-15)	chr1	94012399	94014983	+	0	0	0	0	0	0	-5.15253	0.039841	0.97594	Down
circMFSD8(Ex.3-9)	chr4	1.29E+08	1.29E+08	-	0	0	0	15	7	13	3.162847	0.042389	0.97594	Up
circACSL6(Ex.18-20)	chr5	1.31E+08	1.31E+08	-	0	0	0	0	0	0	-5.15605	0.040338	0.97594	Down
circTMEM169(Ex.2-2)	chr2	2.17E+08	2.17E+08	+	0	0	0	3	31	21	3.387541	0.029601	0.97594	Up
circMAP4K3(Ex.7-15)	chr2	39535084	39560715	-	0	0	0	0	0	0	-4.87673	0.035751	0.97594	Down
circMON2(Ex.27-32)	chr12	62959017	62974200	+	0	0	0	0	0	0	-5.54297	0.025195	0.97594	Down
circCPSF2(Ex.2-9)	chr14	92592466	92609638	+	0	0	0	0	0	0	-5.38005	0.017603	0.97594	Down
circZCCHC17(Ex.4-6)	chr1	31810022	31819587	+	0	0	0	0	0	0	-5.35413	0.031293	0.97594	Down

circRNF111(Ex.3-6)	chr15	59344504	59359282	+	0	0	0	0	0	0	-5.09077	0.044009	0.97594	Down
circHMMR(Ex.13-16)	chr5	1.63E+08	1.63E+08	+	0	0	0	0	0	0	-4.99665	0.049809	0.97594	Down
circTRAPPC9(Ex.13-16)	chr8	1.41E+08	1.41E+08	-	0	0	0	0	8	40	4.528272	0.020991	0.97594	Up
circMAP7D3(Ex.4-5)	chrX	1.35E+08	1.35E+08	-	0	0	0	0	0	0	-5.3548	0.016818	0.97594	Down
circHK3(Ex.14-18)	chr5	1.76E+08	1.76E+08	-	0	0	0	0	0	0	-5.04511	0.048029	0.97594	Down
circDOCK2(Ex.28-32)	chr5	1.69E+08	1.69E+08	+	0	0	0	0	5	29	4.210755	0.031134	0.97594	Up
circUPF2(Ex.5-7)	chr10	12041905	12046726	-	0	0	0	0	0	0	-5.19092	0.038304	0.97594	Down
circAKAP9(Ex.30-31)	chr7	91707010	91709466	+	0	0	0	7	0	15	3.906943	0.04379	0.97594	Up
circDDX6(Ex.6-9)	chr11	1.19E+08	1.19E+08	-	0	0	0	0	0	0	-5.02563	0.049191	0.97594	Down
circCEP250(Ex.18-20)	chr20	34067056	34067970	+	0	0	0	0	0	0	-5.48376	0.026589	0.97594	Down
circNCBP1(Ex.7-18)	chr9	1E+08	1E+08	+	0	0	0	0	0	0	-5.05045	0.046984	0.97594	Down
circCAB39(Ex.2-5)	chr2	2.32E+08	2.32E+08	+	0	0	0	0	28	26	4.173675	0.030854	0.97594	Up
circSASS6(Ex.4-5)	chr1	1.01E+08	1.01E+08	-	0	0	0	41	2	19	4.196569	0.018201	0.97594	Up
circCNTLN(Ex.6-7)	chr9	17273731	17298350	+	0	0	0	66	8	0	4.705904	0.017296	0.97594	Up
circHERC1(Ex.48-50)	chr15	63947941	63950917	-	0	0	0	0	0	0	-5.58795	0.023122	0.97594	Down
circFCHO2(Ex.9-16)	chr5	72330484	72351934	+	0	0	0	35	8	0	4.488965	0.009483	0.97594	Up
circFNIP1(Ex.8-10)	chr5	1.31E+08	1.31E+08	-	0	0	0	14	5	3	3.464343	0.031377	0.97594	Up
circBMP2K(Ex.13-14)	chr4	79793768	79800045	+	0	0	0	14	5	20	4.477093	0.009805	0.97594	Up
circIKBKB(Ex.2-4)	chr8	42129601	42147791	+	0	0	0	0	0	0	-5.26305	0.036188	0.97594	Down
circAIFM1(Ex.7-13)	chrX	1.29E+08	1.29E+08	-	0	0	0	0	12	0	4.112472	0.03225	0.97594	Up
circOPTN(Ex.12-13)	chr10	13169745	13174197	+	0	0	0	20	11	13	4.501698	0.0089	0.97594	Up
circPPP4R3B(Ex.4-4)	chr2	55825552	55826175	-	0	0	0	0	0	0	-5.17075	0.039855	0.97594	Down
circUBE2C(Ex.3-5)	chr20	44443023	44444552	+	0	0	0	0	0	0	-5.12484	0.042906	0.97594	Down
circDAP3(Ex.4-9)	chr1	1.56E+08	1.56E+08	+	0	0	0	0	0	0	-5.12242	0.042178	0.97594	Down
circSS18(Ex.6-10)	chr18	23612363	23619420	-	0	0	0	0	0	0	-5.10132	0.044901	0.97594	Down
circRALB(Ex.3-4)	chr2	1.21E+08	1.21E+08	+	0	0	0	59	10	16	3.624098	0.017744	0.97594	Up
circGAPVD1(Ex.5-9)	chr9	1.28E+08	1.28E+08	+	0	0	0	49	0	0	4.37061	0.025028	0.97594	Up
circWAPL(Ex.10-17)	chr10	88203032	88221044	-	0	0	0	4	9	11	3.627091	0.044682	0.97594	Up
circGDI2(Ex.2-9)	chr10	5808457	5842668	-	0	0	0	0	5	8	4.183075	0.016623	0.97594	Up
circOGT(Ex.20-21)	chrX	70787350	70787966	+	0	0	0	9	22	4	3.265415	0.038768	0.97594	Up
circCHD9(Ex.32-36)	chr16	53340149	53348956	+	0	0	0	3	14	0	4.28274	0.014516	0.97594	Up
circITCH(Ex.17-19)	chr20	33065577	33068537	+	0	0	0	0	3	27	4.433951	0.02199	0.97594	Up
circTRAPPC10(Ex.2-4)	chr21	45451972	45472357	+	0	0	0	14	2	37	4.346512	0.018149	0.97594	Up
circCCDC7(Ex.2-6)	chr10	32740520	32751977	+	0	0	0	8	0	13	4.113062	0.032471	0.97594	Up
circAFG1L(Ex.2-6)	chr6	1.09E+08	1.09E+08	+	0	0	0	0	10	13	4.068126	0.017995	0.97594	Up
circNFX1(Ex.6-11)	chr9	33311104	33332500	+	0	0	0	4	12	14	3.792871	0.014232	0.97594	Up
circJAK2(Ex.9-18)	chr9	5064883	5080683	+	0	0	0	5	27	19	3.890065	0.045571	0.97594	Up
circERLEC1(Ex.7-11)	chr2	54028534	54040210	+	0	0	0	0	0	9	4.047756	0.039983	0.97594	Up
circNCAPD2(Ex.3-5)	chr12	6618883	6619976	+	0	0	0	10	22	29	4.080377	0.008087	0.97594	Up
circPPP6R3(Ex.6-19)	chr11	68318589	68363686	+	0	0	0	9	14	5	4.437538	0.011587	0.97594	Up
circPPIL4(Ex.8-10)	chr6	1.5E+08	1.5E+08	-	0	0	0	8	6	11	3.855864	0.030456	0.97594	Up
circORC3(Ex.6-7)	chr6	88317391	88318947	+	0	0	0	8	5	4	3.49899	0.030305	0.97594	Up
circTAF1(Ex.24-25)	chrX	70618422	70621589	+	0	0	0	24	5	17	4.129209	0.017697	0.97594	Up
circFXR2(Ex.2-6)	chr17	7506227	7509467	-	0	0	0	10	0	15	4.419813	0.020625	0.97594	Up
circSCLT1(Ex.12-17)	chr4	1.3E+08	1.3E+08	-	0	0	0	0	6	5	3.574764	0.043424	0.97594	Up
circSNTB1(Ex.2-2)	chr8	1.22E+08	1.22E+08	-	0	0	0	23	23	0	4.123843	0.013823	0.97594	Up

circNARS2(Ex.2-6)	chr11	78239888	78282489	-	0	0	0	12	14	0	4.41004	0.010703	0.97594	Up
circATP9B(Ex.9-11)	chr18	76953183	76974038	+	0	0	0	8	6	16	3.883066	0.029868	0.97594	Up
circEPB41(Ex.10-17)	chr1	29362338	29424447	+	0	0	0	27	11	9	4.134491	0.008577	0.97594	Up
circHMG20A(Ex.5-9)	chr15	77763252	77771663	+	0	0	0	70	17	5	3.910814	0.012519	0.97594	Up
circAGAP2(Ex.4-5)	chr12	58127809	58128187	-	0	0	0	17	0	0	3.853385	0.041884	0.97594	Up
circFKBP15(Ex.4-8)	chr9	1.16E+08	1.16E+08	-	0	0	0	3	8	14	3.963526	0.012831	0.97594	Up
circTECR(Ex.6-8)	chr19	14674794	14675670	+	0	0	0	13	4	20	3.423742	0.025476	0.97594	Up
circZGRF1(Ex.7-10)	chr4	1.14E+08	1.14E+08	-	0	0	0	50	2	3	3.708444	0.039408	0.97594	Up
circATAD2B(Ex.3-13)	chr2	24080285	24111269	-	0	0	0	15	6	7	4.186267	0.016013	0.97594	Up
circPPP6R3(Ex.10-13)	chr11	68334482	68341692	+	0	0	0	0	15	18	3.984072	0.023542	0.97594	Up
circMYH10(Ex.27-27)	chr17	8409636	8409807	-	0	0	0	6	18	0	4.352705	0.023722	0.97594	Up
circVIRMA(Ex.17-19)	chr8	95507959	95511734	-	0	0	0	46	10	10	3.467064	0.022374	0.97594	Up
circARHGAP26(Ex.9-11)	chr5	1.42E+08	1.42E+08	+	0	0	0	15	6	18	4.132977	0.015907	0.97594	Up
circWDR47(Ex.5-8)	chr1	1.1E+08	1.1E+08	-	0	0	0	13	18	0	4.042313	0.037161	0.97594	Up
circTBC1D14(Ex.6-8)	chr4	7000812	7006651	+	0	0	0	0	10	20	3.768801	0.049421	0.97594	Up
circSPIDR(Ex.16-19)	chr8	48626051	48642027	+	0	0	0	23	0	0	5.173775	0.009574	0.97594	Up
circPICALM(Ex.7-16)	chr11	85692788	85722179	-	0	0	0	7	0	6	4.462382	0.032357	0.97594	Up
circLIMA1(Ex.7-9)	chr12	50586235	50594667	-	0	0	0	0	6	5	4.311731	0.041844	0.97594	Up
circADAL(Ex.3-5)	chr15	43625509	43628024	+	0	0	0	0	5	11	4.721537	0.010594	0.97594	Up
circHK1(Ex.8-14)	chr10	71136690	71149052	+	0	0	0	7	0	4	4.627981	0.025971	0.97594	Up
circPCM1(Ex.19-23)	chr8	17823508	17830196	+	0	0	0	24	14	0	4.897367	0.031084	0.97594	Up
circTNFAIP3(Ex.2-3)	chr6	1.38E+08	1.38E+08	+	0	0	0	0	14	7	4.945499	0.006842	0.97594	Up
circSETD1A(Ex.13-13)	chr16	30982699	30983040	+	0	0	0	10	12	0	4.422876	0.036453	0.97594	Up
circPI4K2A(Ex.3-5)	chr10	99416042	99422725	+	0	0	0	23	0	27	5.345136	0.016052	0.97594	Up
circSCAP(Ex.2-3)	chr3	47476498	47484581	-	0	0	0	0	0	32	5.632013	0.010557	0.97594	Up
circST7(Ex.4-9)	chr7	1.17E+08	1.17E+08	+	0	0	0	28	0	48	6.517648	8.46E-04	0.97594	Up
circFLVCR1(Ex.3-5)	chr1	2.13E+08	2.13E+08	+	0	0	0	4	17	0	4.65761	0.025528	0.97594	Up
circGGNBP2(Ex.3-4)	chr17	34910661	34913176	+	0	0	0	11	0	5	4.86585	0.01684	0.97594	Up
circPPP6R3(Ex.9-13)	chr11	68331771	68341692	+	0	0	0	24	5	4	4.592	0.029509	0.97594	Up
circTFRC(Ex.5-13)	chr3	1.96E+08	1.96E+08	-	0	0	0	39	5	15	5.290695	0.010355	0.97594	Up
circTANGO6(Ex.4-6)	chr16	68900982	68912183	+	0	0	0	0	0	12	4.531203	0.04877	0.97594	Up
circDIP2B(Ex.14-17)	chr12	51086732	51090958	+	0	0	0	13	0	11	4.485696	0.03182	0.97594	Up
circCAPRIN2(Ex.11-16)	chr12	30867893	30877385	-	0	0	0	16	3	49	6.075412	2.33E-04	0.97594	Up
circRNPEP(Ex.4-5)	chr1	2.02E+08	2.02E+08	+	0	0	0	5	21	0	4.693443	0.041875	0.97594	Up
circTMEM62(Ex.5-10)	chr15	43438691	43452992	+	0	0	0	17	4	0	4.644142	0.024738	0.97594	Up
circOBI1(Ex.2-3)	chr13	79216257	79219132	-	0	0	0	12	0	6	4.184925	0.03081	0.97594	Up
circPIK3CB(Ex.13-16)	chr3	1.38E+08	1.38E+08	-	0	0	0	12	0	7	4.589846	0.025901	0.97594	Up
circRALGAPB(Ex.2-5)	chr20	37117046	37128276	+	0	0	0	5	0	0	4.614253	0.024895	0.97594	Up
circSLC44A1(Ex.9-11)	chr9	1.08E+08	1.08E+08	+	0	0	0	0	6	8	4.596666	0.026616	0.97594	Up
circSETDB1(Ex.2-2)	chr1	1.51E+08	1.51E+08	+	0	0	0	9	2	22	5.014398	0.007092	0.97594	Up
circSGMS1(Ex.3-5)	chr10	52220433	52279681	-	0	0	0	24	7	0	4.816099	0.01917	0.97594	Up
circRABEP1(Ex.6-7)	chr17	5250085	5253924	+	0	0	0	22	0	0	4.595702	0.046783	0.97594	Up
circTMEM181(Ex.2-10)	chr6	1.59E+08	1.59E+08	+	0	0	0	56	19	0	5.807421	0.004067	0.97594	Up
circST7L(Ex.5-7)	chr1	1.13E+08	1.13E+08	-	0	0	0	8	0	0	4.964167	0.027587	0.97594	Up
circSYNE2(Ex.65-67)	chr14	64574209	64586327	+	0	0	0	3	18	0	5.16569	0.012261	0.97594	Up
circSUSD1(Ex.11-14)	chr9	1.15E+08	1.15E+08	-	0	0	0	5	8	0	4.755262	0.02116	0.97594	Up

circSETX(Ex.3-4)	chr9	1.35E+08	1.35E+08	-	0	0	0	20	0	0	4.574924	0.045585	0.97594	Up
circSLCO4C1(Ex.2-8)	chr5	1.02E+08	1.02E+08	-	0	0	0	11	0	0	4.992339	0.015157	0.97594	Up
circHIPK2(Ex.6-9)	chr7	1.39E+08	1.39E+08	-	0	0	0	23	0	5	5.030666	0.005623	0.97594	Up
circSLC36A1(Ex.2-9)	chr5	1.51E+08	1.51E+08	+	0	0	0	22	3	0	4.746774	0.038952	0.97594	Up
circKIF24(Ex.3-6)	chr9	34286615	34306439	-	0	0	0	0	0	0	5.066477	0.02316	0.97594	Up
circHNRNPUL2(Ex.2-10)	chr11	62487495	62491898	-	0	0	0	8	0	6	5.285926	0.008524	0.97594	Up
circTANGO6(Ex.6-7)	chr16	68912021	68914533	+	0	0	0	4	9	4	4.257213	0.047062	0.97594	Up
circMCTP2(Ex.11-14)	chr15	94910834	94928754	+	0	0	0	18	0	0	5.12943	0.010536	0.97594	Up
circFAM228B(Ex.4-6)	chr2	24360779	24369705	+	0	0	0	5	4	8	4.136336	0.033297	0.97594	Up
circPLXDC2(Ex.4-7)	chr10	20357099	20453496	+	0	0	0	7	13	0	4.260026	0.046915	0.97594	Up
circSTN1(Ex.3-5)	chr10	1.06E+08	1.06E+08	-	0	0	0	5	0	14	4.731693	0.011018	0.97594	Up
circMTM1(Ex.6-10)	chrX	1.5E+08	1.5E+08	+	0	0	0	13	0	2	4.27613	0.046681	0.97594	Up
circSTAT6(Ex.12-14)	chr12	57493779	57496704	-	0	0	0	27	21	0	5.362752	0.015885	0.97594	Up
circRHAG(Ex.6-9)	chr6	49574561	49580247	-	0	0	0	0	3	0	4.756394	0.02363	0.97594	Up
circFERMT1(Ex.7-9)	chr20	6075586	6078278	-	0	0	0	8	7	0	4.662471	0.023591	0.97594	Up
circSEL1L(Ex.7-10)	chr14	81964236	81966006	-	0	0	0	32	10	0	5.032654	0.025882	0.97594	Up
circGLE1(Ex.2-2)	chr9	1.31E+08	1.31E+08	+	0	0	0	3	11	0	4.74219	0.022357	0.97594	Up
circFRMD4A(Ex.13-24)	chr10	13693903	13749113	-	0	0	0	0	6	11	4.364207	0.022995	0.97594	Up
circUBR2(Ex.25-28)	chr6	42620229	42626058	+	0	0	0	18	19	0	4.955012	0.0287	0.97594	Up
circEPS15(Ex.9-21)	chr1	51860053	51913807	-	0	0	0	14	0	0	4.76255	0.035363	0.97594	Up
circHEATR5B(Ex.18-21)	chr2	37264998	37276986	-	0	0	0	11	14	0	4.622491	0.044081	0.97594	Up
circSEPTIN6(Ex.3-6)	chrX	1.19E+08	1.19E+08	-	0	0	0	8	2	0	4.53199	0.031554	0.97594	Up
circZER1(Ex.9-12)	chr9	1.32E+08	1.32E+08	-	0	0	0	42	0	2	5.611732	0.00695	0.97594	Up
circPTBP3(Ex.6-10)	chr9	1.15E+08	1.15E+08	-	0	0	0	0	0	26	5.071096	0.041455	0.97594	Up
circAATF(Ex.3-4)	chr17	35310186	35311207	+	0	0	0	16	0	2	4.550055	0.029783	0.97594	Up
circCTNNAL1(Ex.7-11)	chr9	1.12E+08	1.12E+08	-	0	0	0	6	4	0	4.2952	0.042707	0.97594	Up
circCPT1A(Ex.3-5)	chr11	68571468	68580044	-	0	0	0	8	0	0	4.293939	0.041728	0.97594	Up
circPTPN4(Ex.16-18)	chr2	1.21E+08	1.21E+08	+	0	0	0	0	6	10	5.257178	0.004172	0.97594	Up
circOPTN(Ex.6-7)	chr10	13158267	13161040	+	0	0	0	0	15	0	4.754322	0.036934	0.97594	Up
circCCAR2(Ex.2-3)	chr8	22463249	22463689	+	0	0	0	17	4	18	5.415107	0.002821	0.97594	Up
circKNL1(Ex.2-4)	chr15	40895113	40898650	+	0	0	0	0	0	28	4.884677	0.031352	0.97594	Up
circCCDC138(Ex.3-9)	chr2	1.09E+08	1.09E+08	+	0	0	0	25	2	9	5.808599	0.001407	0.97594	Up
circMED13(Ex.16-20)	chr17	60042367	60060475	-	0	0	0	9	4	0	4.693842	0.012389	0.97594	Up
circARMC8(Ex.3-6)	chr3	1.38E+08	1.38E+08	+	0	0	0	22	3	0	4.453439	0.035722	0.97594	Up
circNAP1L4(Ex.3-6)	chr11	2992678	2999572	-	0	0	0	4	0	21	4.657604	0.04313	0.97594	Up
circCLASP2(Ex.9-16)	chr3	33648083	33673863	-	0	0	0	37	2	0	4.842007	0.036353	0.97594	Up
circCWC22(Ex.7-14)	chr2	1.81E+08	1.81E+08	-	0	0	0	15	5	7	5.403159	0.002631	0.97594	Up
circDHX57(Ex.11-14)	chr2	39055482	39074215	-	0	0	0	33	0	0	5.603189	0.004334	0.97594	Up
circCLIP1(Ex.4-5)	chr12	1.23E+08	1.23E+08	-	0	0	0	6	11	0	4.491012	0.032178	0.97594	Up
circAEBP2(Ex.2-3)	chr12	19615444	19626289	+	0	0	0	26	16	0	5.283665	0.017755	0.97594	Up
circCDC27(Ex.2-4)	chr17	45247283	45259003	-	0	0	0	11	0	22	5.514592	0.002242	0.97594	Up
circFAF1(Ex.5-6)	chr1	51204535	51210447	-	0	0	0	19	0	6	4.741619	0.023545	0.97594	Up
circNUP42(Ex.2-2)	chr7	23224689	23224917	+	0	0	0	4	16	0	4.592182	0.01686	0.97594	Up
circSREK1IP1(Ex.2-4)	chr5	64023934	64050189	-	0	0	0	12	0	2	3.941168	0.048493	0.97594	Up
circPPP4R3B(Ex.5-16)	chr2	55785911	55816092	-	0	0	0	17	0	0	4.851049	0.016367	0.97594	Up
circPRUNE1(Ex.4-6)	chr1	1.51E+08	1.51E+08	+	0	0	0	5	12	6	4.249881	0.048261	0.97594	Up

circFRYL(Ex.11-14)	chr4	48597914	48607850	-	0	0	0	29	0	0	5.949341	0.002242	0.97594	Up
circSMCHD1(Ex.8-10)	chr18	2694525	2698039	+	0	0	0	20	0	0	4.622164	0.043283	0.97594	Up
circUSP32(Ex.3-4)	chr17	58372044	58379065	-	0	0	0	0	17	0	5.278087	0.008181	0.97594	Up
circGUF1(Ex.2-6)	chr4	44682458	44685335	+	0	0	0	0	9	11	4.600678	0.026613	0.97594	Up
circHECTD1(Ex.7-26)	chr14	31596991	31641328	-	0	0	0	8	0	6	4.599134	0.025984	0.97594	Up
circTXNDC16(Ex.13-16)	chr14	52936755	52949683	-	0	0	0	15	7	20	5.48796	0.002098	0.97594	Up
circWDHD1(Ex.23-25)	chr14	55411050	55423922	-	0	0	0	3	0	9	4.195515	0.049901	0.97594	Up
circTFRC(Ex.6-8)	chr3	1.96E+08	1.96E+08	-	0	0	0	29	7	0	6.166966	4.31E-04	0.97594	Up
circMICAL2(Ex.3-3)	chr11	12183626	12183966	+	0	0	0	0	8	0	4.670343	0.041136	0.97594	Up
circSHARPIN(Ex.2-8)	chr8	1.45E+08	1.45E+08	-	0	0	0	8	0	0	4.371672	0.036214	0.97594	Up
circVMP1(Ex.5-7)	chr17	57816198	57851246	+	0	0	0	6	11	0	4.254054	0.048221	0.97594	Up
circIQGAP1(Ex.12-15)	chr15	90996007	90999547	+	0	0	0	0	26	2	5.081593	0.014206	0.97594	Up
circSWT1(Ex.2-9)	chr1	1.85E+08	1.85E+08	+	0	0	0	7	24	0	5.02529	0.007563	0.97594	Up
circSFI1(Ex.2-4)	chr22	31904241	31927115	+	0	0	0	0	2	12	4.750536	0.022486	0.97594	Up
circSON(Ex.7-9)	chr21	34939463	34945761	+	0	0	0	6	5	6	3.988784	0.045067	0.97594	Up
circUSP37(Ex.6-8)	chr2	2.19E+08	2.19E+08	-	0	0	0	0	0	8	4.703817	0.026081	0.97594	Up
circMYBL2(Ex.2-5)	chr20	42302446	42315712	+	0	0	0	2	10	4	4.419281	0.038851	0.97594	Up
circTNIK(Ex.3-12)	chr3	1.71E+08	1.71E+08	-	0	0	0	0	2	25	4.717043	0.042203	0.97594	Up
circTSC1(Ex.3-6)	chr9	1.36E+08	1.36E+08	-	0	0	0	0	3	18	5.252588	0.009758	0.97594	Up
circATF2(Ex.5-12)	chr2	1.76E+08	1.76E+08	-	0	0	0	0	34	14	5.707233	0.001798	0.97594	Up
circPBRM1(Ex.11-12)	chr3	52668618	52676061	-	0	0	0	5	0	16	4.709983	0.013068	0.97594	Up
circUSP20(Ex.7-9)	chr9	1.33E+08	1.33E+08	+	0	0	0	8	6	0	4.792617	0.008769	0.97594	Up
circCUX1(Ex.5-6)	chr7	1.02E+08	1.02E+08	+	0	0	0	0	5	8	4.360749	0.038428	0.97594	Up
circINTS6(Ex.7-9)	chr13	51957465	51961676	-	0	0	0	12	18	19	5.644509	0.001433	0.97594	Up
circPIKFYVE(Ex.23-26)	chr2	2.09E+08	2.09E+08	+	0	0	0	5	0	15	4.294951	0.04321	0.97594	Up
circZNF638(Ex.3-9)	chr2	71582849	71607410	+	0	0	0	7	9	12	4.684735	0.012254	0.97594	Up
circANKRD27(Ex.17-18)	chr19	33113328	33116872	-	0	0	0	10	4	0	4.545574	0.029133	0.97594	Up
circCLEC16A(Ex.4-7)	chr16	11063018	11066918	+	0	0	0	11	5	0	4.421306	0.034514	0.97594	Up
circVPS8(Ex.14-18)	chr3	1.85E+08	1.85E+08	+	0	0	0	0	16	0	4.858593	0.032001	0.97594	Up
circTMEM41B(Ex.2-5)	chr11	9309249	9321248	-	0	0	0	4	11	0	4.656917	0.024594	0.97594	Up
circNUP107(Ex.12-27)	chr12	69109407	69135760	+	0	0	0	27	2	4	4.909443	0.009439	0.97594	Up
circSTAT6(Ex.12-19)	chr12	57492288	57496704	-	0	0	0	0	6	17	4.891306	0.008409	0.97594	Up
circAKAP9(Ex.4-6)	chr7	91621472	91624090	+	0	0	0	0	0	34	5.107344	0.023369	0.97594	Up
circSLC9C1(Ex.2-5)	chr3	1.12E+08	1.12E+08	-	0	0	0	6	9	0	4.574322	0.028155	0.97594	Up
circMTHFD1(Ex.10-18)	chr14	64892448	64906984	+	0	0	0	7	7	24	5.948572	1.36E-04	0.97594	Up
circHMGCS1(Ex.2-4)	chr5	43298111	43307926	-	0	0	0	0	6	17	4.934339	0.009056	0.97594	Up
circR3HDM2(Ex.3-4)	chr12	57696959	57704246	-	0	0	0	0	15	7	4.90719	0.016376	0.97594	Up
circRIOX2(Ex.3-8)	chr3	97666205	97680552	-	0	0	0	5	11	0	4.97979	0.0148	0.97594	Up
circUSP14(Ex.8-12)	chr18	197616	203190	+	0	0	0	9	0	17	4.798367	0.018762	0.97594	Up
circCHD9(Ex.2-2)	chr16	53189838	53191453	+	0	0	0	4	7	0	4.412659	0.037307	0.97594	Up
circNARF(Ex.4-7)	chr17	80426640	80439087	+	0	0	0	0	8	7	4.538707	0.015663	0.97594	Up
circMAP3K8(Ex.3-3)	chr10	30727845	30728203	+	0	0	0	28	0	0	4.738493	0.03749	0.97594	Up
circTMBIM6(Ex.5-8)	chr12	50152010	50153104	+	0	0	0	2	13	3	4.592374	0.016465	0.97594	Up
circFANCI(Ex.24-27)	chr15	89838146	89844673	+	0	0	0	8	3	0	4.324812	0.041579	0.97594	Up
circEXOC4(Ex.5-6)	chr7	1.33E+08	1.33E+08	+	0	0	0	0	7	9	4.922317	0.007385	0.97594	Up
circFGD4(Ex.11-15)	chr12	32772632	32786623	+	0	0	0	0	7	12	4.367073	0.040773	0.97594	Up

circBARD1(Ex.2-4)	chr2	2.16E+08	2.16E+08	-	0	0	0	0	20	0	5.085664	0.023417	0.97594	Up
circXPO4(Ex.14-15)	chr13	21374295	21375129	-	0	0	0	0	8	0	5.102068	0.011374	0.97594	Up
circUTRN(Ex.70-72)	chr6	1.45E+08	1.45E+08	+	0	0	0	7	0	0	4.706581	0.038841	0.97594	Up
circATAD5(Ex.8-9)	chr17	29183973	29185341	+	0	0	0	0	12	17	4.858403	0.032442	0.97594	Up
circFECH(Ex.8-10)	chr18	55218547	55222184	-	0	0	0	4	2	0	4.208074	0.03183	0.97594	Up
circTTK(Ex.11-21)	chr6	80732041	80750420	+	0	0	0	13	18	0	4.864669	0.032064	0.97594	Up
circSMARCA4(Ex.15-17)	chr19	11121057	11129699	+	0	0	0	19	7	6	4.919314	0.003406	0.97594	Up
circLARP1(Ex.9-10)	chr5	1.54E+08	1.54E+08	+	0	0	0	0	9	0	4.650651	0.041793	0.97594	Up
circNR2C2(Ex.3-11)	chr3	15055096	15076316	+	0	0	0	23	0	10	4.825019	0.033154	0.97594	Up
circMAPK9(Ex.3-8)	chr5	1.8E+08	1.8E+08	-	0	0	0	7	15	4	4.581899	0.029128	0.97594	Up
circSYNE1(Ex.9-10)	chr6	1.53E+08	1.53E+08	-	0	0	0	8	0	7	4.97422	0.007133	0.97594	Up
circTRAPPC13(Ex.4-9)	chr5	64933523	64954328	+	0	0	0	0	0	21	4.795453	0.035714	0.97594	Up
circRTN4IP1(Ex.5-8)	chr6	1.07E+08	1.07E+08	-	0	0	0	0	0	28	5.22461	0.019085	0.97594	Up
circFCHSD2(Ex.4-5)	chr11	72712035	72726930	-	0	0	0	3	0	0	4.961665	0.016126	0.97594	Up
circSERGEF(Ex.2-9)	chr11	17981017	18029623	-	0	0	0	37	0	0	5.033324	0.013956	0.97594	Up
circANLN(Ex.12-18)	chr7	36460206	36465349	+	0	0	0	9	0	0	5.225858	0.019011	0.97594	Up
circLMBR1(Ex.2-5)	chr7	1.57E+08	1.57E+08	-	0	0	0	12	0	0	4.75468	0.035812	0.97594	Up
circCLEC16A(Ex.21-21)	chr16	11217599	11217803	+	0	0	0	7	7	6	4.502414	0.031665	0.97594	Up
circGLB1(Ex.3-9)	chr3	33093250	33110462	-	0	0	0	0	12	0	5.29954	0.03258	0.97594	Up
circHIPK1(Ex.2-2)	chr1	1.14E+08	1.14E+08	+	0	0	0	18	0	7	4.862383	0.017318	0.97594	Up
circPRUNE1(Ex.7-7)	chr1	1.51E+08	1.51E+08	+	0	0	0	7	11	0	4.481515	0.031829	0.97594	Up
circKANSL2(Ex.3-5)	chr12	49065582	49073616	-	0	0	0	4	4	0	4.034171	0.039949	0.97594	Up
circSF3B3(Ex.16-24)	chr16	70594372	70604052	+	0	0	0	11	11	0	5.372913	0.016328	0.97594	Up
circNECAP1(Ex.4-6)	chr12	8244365	8245651	+	0	0	0	5	2	9	4.593531	0.015305	0.97594	Up
circATM(Ex.23-26)	chr11	1.08E+08	1.08E+08	+	0	0	0	8	0	0	4.791536	0.034506	0.97594	Up
circUBAP2(Ex.9-10)	chr9	33960824	33963789	-	0	0	0	12	7	0	4.464377	0.032481	0.97594	Up
circYPEL1(Ex.2-2)	chr22	22064917	22065197	-	0	0	0	0	10	0	4.685501	0.040662	0.97594	Up
circSTIM1(Ex.7-10)	chr11	4095732	4104728	+	0	0	0	0	6	5	4.183902	0.03184	0.97594	Up
circANKRD27(Ex.5-13)	chr19	33122308	33135385	-	0	0	0	6	0	3	4.265155	0.027871	0.97594	Up
circBRCA1(Ex.14-18)	chr17	41215350	41226538	-	0	0	0	5	7	0	4.201024	0.049271	0.97594	Up
circVPS13C(Ex.2-15)	chr15	62299507	62336454	-	0	0	0	0	2	0	4.37356	0.03963	0.97594	Up
circATXN7(Ex.3-5)	chr3	63898264	63938159	+	0	0	0	8	4	0	4.276143	0.043753	0.97594	Up
circHERC1(Ex.13-26)	chr15	63990927	64027048	-	0	0	0	27	0	0	4.525405	0.033762	0.97594	Up
circUSP36(Ex.16-16)	chr17	76799467	76800060	-	0	0	0	8	6	0	4.585218	0.030037	0.97594	Up
circBRCA1(Ex.4-9)	chr17	41247863	41258550	-	0	0	0	9	16	6	5.212902	0.001358	0.97594	Up
circNFATC3(Ex.4-6)	chr16	68191772	68208417	+	0	0	0	0	10	19	4.572213	0.048849	0.97594	Up
circUSP24(Ex.24-29)	chr1	55600001	55607404	-	0	0	0	14	0	20	5.42015	0.002333	0.97594	Up
circNUSAP1(Ex.5-8)	chr15	41648237	41663882	+	0	0	0	11	13	13	5.154184	0.010717	0.97594	Up
circDTL(Ex.3-8)	chr1	2.12E+08	2.12E+08	+	0	0	0	0	6	11	4.302607	0.042663	0.97594	Up
circSLC37A3(Ex.4-7)	chr7	1.4E+08	1.4E+08	-	0	0	0	16	2	5	4.511558	0.018837	0.97594	Up
circFANCI(Ex.13-15)	chr15	89819942	89824531	+	0	0	0	17	0	0	4.995743	0.013413	0.97594	Up
circPRKAA1(Ex.7-8)	chr5	40764616	40765340	-	0	0	0	6	6	0	4.8811	0.017077	0.97594	Up
circZNF207(Ex.5-6)	chr17	30687911	30688534	+	0	0	0	0	5	17	5.614543	0.004942	0.97594	Up
circPPP2CA(Ex.2-6)	chr5	1.34E+08	1.34E+08	-	0	0	0	0	9	14	4.532789	0.049638	0.97594	Up
circIL10RA(Ex.4-6)	chr11	1.18E+08	1.18E+08	+	0	0	0	8	0	2	4.334689	0.041169	0.97594	Up
circPTBP3(Ex.2-4)	chr9	1.15E+08	1.15E+08	-	0	0	0	0	10	2	4.360955	0.024001	0.97594	Up

circRICTOR(Ex.8-12)	chr5	38968045	38982138	-	0	0	0	4	9	0	4.57431	0.015444	0.97594	Up
circADAM10(Ex.6-8)	chr15	58932976	58938403	-	0	0	0	13	0	0	4.926201	0.029187	0.97594	Up
circGCLC(Ex.7-10)	chr6	53371711	53374048	-	0	0	0	24	12	11	5.994832	9.88E-05	0.97594	Up
circHSPA4(Ex.13-16)	chr5	1.32E+08	1.32E+08	+	0	0	0	15	30	10	5.486137	0.006656	0.97594	Up
circSORT1(Ex.4-7)	chr1	1.1E+08	1.1E+08	-	0	0	0	36	4	0	5.120546	0.012375	0.97594	Up
circMCTP1(Ex.6-10)	chr5	94244956	94267696	-	0	0	0	16	0	0	4.573857	0.046007	0.97594	Up
circSLC25A17(Ex.3-5)	chr22	41175013	41190584	-	0	0	0	22	0	10	4.614685	0.044605	0.97594	Up
circTGM2(Ex.7-10)	chr20	36766515	36770601	-	0	0	0	0	9	0	4.722784	0.038348	0.97594	Up
circMTR(Ex.20-23)	chr1	2.37E+08	2.37E+08	+	0	0	0	0	0	9	4.671308	0.041046	0.97594	Up
circSNW1(Ex.6-10)	chr14	78197331	78203418	-	0	0	0	14	18	4	4.69754	0.042035	0.97594	Up
circCD36(Ex.6-14)	chr7	80292306	80303463	+	0	0	0	15	19	0	4.920118	0.029897	0.97594	Up
circANKRD12(Ex.3-7)	chr18	9195549	9216898	+	0	0	0	6	6	15	5.009217	0.007167	0.97594	Up
circARHGEF12(Ex.12-17)	chr11	1.2E+08	1.2E+08	+	0	0	0	4	0	9	4.349702	0.03902	0.97594	Up
circECT2(Ex.7-14)	chr3	1.72E+08	1.72E+08	+	0	0	0	9	0	13	4.612233	0.044082	0.97594	Up
circSLC11A2(Ex.2-11)	chr12	51388327	51404549	-	0	0	0	4	7	15	4.612879	0.014814	0.97594	Up
circDENND5A(Ex.9-15)	chr11	9171628	9192324	-	0	0	0	0	0	9	4.29107	0.041237	0.97594	Up
circZDHHC20(Ex.6-11)	chr13	21955573	21975824	-	0	0	0	9	12	8	4.846067	0.008874	0.97594	Up
circRCOR1(Ex.9-10)	chr14	1.03E+08	1.03E+08	+	0	0	0	11	3	9	4.749534	0.011448	0.97594	Up
circDDHD2(Ex.7-10)	chr8	38099768	38105353	+	0	0	0	23	0	0	4.543048	0.048344	0.97594	Up
circSP100(Ex.25-28)	chr2	2.31E+08	2.31E+08	+	0	0	0	4	0	14	4.559333	0.030498	0.97594	Up
circPRPSAP2(Ex.5-6)	chr17	18775896	18781183	+	0	0	0	30	3	0	4.686663	0.042803	0.97594	Up
circDPY19L1(Ex.8-10)	chr7	35006506	35013217	-	0	0	0	3	0	13	4.884837	0.009922	0.97594	Up
circAGFG2(Ex.2-6)	chr7	1E+08	1E+08	+	0	0	0	8	0	7	4.432493	0.019968	0.97594	Up
circPAPOLA(Ex.19-20)	chr14	97022512	97027048	+	0	0	0	4	4	5	4.158017	0.032629	0.97594	Up
circSQSTM1(Ex.2-5)	chr5	1.79E+08	1.79E+08	+	0	0	0	9	0	10	4.180504	0.049993	0.97594	Up
circME1(Ex.2-5)	chr6	84055892	84117620	-	0	0	0	27	13	6	5.17212	0.011007	0.97594	Up
circKRIT1(Ex.2-3)	chr7	91873316	91874485	-	0	0	0	8	0	21	5.399585	0.002479	0.97594	Up
circMDN1(Ex.89-89)	chr6	90368329	90368636	-	0	0	0	3	8	0	4.330474	0.041281	0.97594	Up
circDHX8(Ex.17-22)	chr17	41590730	41599594	+	0	0	0	8	11	17	5.083839	0.012089	0.97594	Up
circLCMT1(Ex.4-5)	chr16	25151492	25162936	+	0	0	0	14	6	10	5.06363	0.005374	0.97594	Up
circGRAMD1B(Ex.12-14)	chr11	1.23E+08	1.23E+08	+	0	0	0	8	4	0	4.31098	0.025283	0.97594	Up
circBAZ1B(Ex.2-3)	chr7	72922657	72925182	-	0	0	0	5	8	23	4.677399	0.043277	0.97594	Up
circNCOA5(Ex.2-7)	chr20	44691999	44708092	-	0	0	0	9	0	8	4.580315	0.026292	0.97594	Up
circHCK(Ex.3-5)	chr20	30661122	30662524	+	0	0	0	9	4	28	5.123021	0.005748	0.97594	Up
circCFLAR(Ex.9-9)	chr2	2.02E+08	2.02E+08	+	0	0	0	5	8	0	4.308979	0.042191	0.97594	Up
circKIF11(Ex.20-21)	chr10	94409592	94410274	+	0	0	0	23	0	0	4.827682	0.034922	0.97594	Up
circEPS15(Ex.6-11)	chr1	51910561	51930998	-	0	0	0	30	4	0	4.647713	0.044828	0.97594	Up
circRAB11FIP4(Ex.6-9)	chr17	29848933	29851014	+	0	0	0	4	0	24	5.167697	0.010775	0.97594	Up
circARHGEF37(Ex.4-10)	chr5	1.49E+08	1.49E+08	+	0	0	0	7	0	17	4.716068	0.024286	0.97594	Up
circSLC25A24(Ex.5-7)	chr1	1.09E+08	1.09E+08	-	0	0	0	18	0	11	4.749544	0.020609	0.97594	Up
circSCYL2(Ex.2-5)	chr12	1.01E+08	1.01E+08	+	0	0	0	8	0	0	4.252282	0.044327	0.97594	Up
circSMARCC1(Ex.2-8)	chr3	47755905	47814426	-	0	0	0	9	4	29	5.10437	0.006442	0.97594	Up
circSDCCAG8(Ex.6-9)	chr1	2.43E+08	2.43E+08	+	0	0	0	9	16	14	5.019231	0.014023	0.97594	Up
circLRIG1(Ex.2-9)	chr3	66455622	66512933	-	0	0	0	14	5	13	4.80778	0.019361	0.97594	Up
circHNRNPU(Ex.8-10)	chr1	2.45E+08	2.45E+08	-	0	0	0	5	35	0	5.227555	0.021169	0.97594	Up
circMPP7(Ex.5-8)	chr10	28412960	28436492	-	0	0	0	31	0	6	4.587514	0.049655	0.97594	Up

circNUP98(Ex.29-30)	chr11	3704430	3707424	-	0	0	0	15	0	0	4.935572	0.027841	0.97594	Up
circSPPL2A(Ex.9-12)	chr15	51017418	51024881	-	0	0	0	12	0	34	4.944388	0.048958	0.97594	Up
circUSP9X(Ex.2-6)	chrX	40982724	40996275	+	0	0	0	19	12	0	4.751299	0.021727	0.97594	Up
circLRP6(Ex.20-22)	chr12	12277499	12279855	-	0	0	0	17	5	6	4.675495	0.013921	0.97594	Up
circMIS18BP1(Ex.6-9)	chr14	45696866	45705147	-	0	0	0	22	0	12	4.611967	0.04535	0.97594	Up
circATP7A(Ex.3-4)	chrX	77243738	77245454	+	0	0	0	12	2	0	4.684114	0.043386	0.97594	Up
circSEPTIN2(Ex.8-9)	chr2	2.42E+08	2.42E+08	+	0	0	0	6	0	24	4.717535	0.039785	0.97594	Up
circACADM(Ex.2-4)	chr1	76194086	76198607	+	0	0	0	9	0	10	4.439508	0.018301	0.97594	Up
circGTF2H1(Ex.5-11)	chr11	18361111	18373993	+	0	0	0	3	0	12	4.51911	0.03088	0.97594	Up
circMICAL2(Ex.8-17)	chr11	12237766	12261132	+	0	0	0	7	2	0	5.106809	0.005861	0.97594	Up
circAAAS(Ex.2-7)	chr12	53708082	53714476	-	0	0	0	20	4	5	5.441445	0.001067	0.97594	Up
circMANBA(Ex.2-7)	chr4	1.04E+08	1.04E+08	-	0	0	0	8	8	16	4.867563	0.017599	0.97594	Up
circCPT1A(Ex.7-8)	chr11	68562272	68564401	-	0	0	0	11	5	0	5.01315	0.015207	0.97594	Up
circTNPO1(Ex.4-7)	chr5	72151601	72168547	+	0	0	0	9	0	0	4.746128	0.036714	0.97594	Up
circGSAP(Ex.3-9)	chr7	77006603	77033932	-	0	0	0	3	6	5	4.279638	0.026787	0.97594	Up
circUSF2(Ex.2-7)	chr19	35760349	35762044	+	0	0	0	4	6	6	5.03509	0.002198	0.97594	Up
circARMH3(Ex.9-16)	chr10	1.04E+08	1.04E+08	-	0	0	0	8	9	0	4.383072	0.037218	0.97594	Up
circMRE11(Ex.3-6)	chr11	94211901	94224131	-	0	0	0	26	0	0	4.858925	0.031606	0.97594	Up
circMFSD14B(Ex.6-9)	chr9	97207246	97216352	+	0	0	0	11	0	3	4.320746	0.041032	0.97594	Up
circCCSER1(Ex.2-3)	chr4	91229395	91234198	+	0	0	0	9	0	5	4.395781	0.019513	0.97594	Up
circITGA4(Ex.15-16)	chr2	1.82E+08	1.82E+08	+	0	0	0	20	6	18	5.316933	0.008034	0.97594	Up
circATP2C1(Ex.12-15)	chr3	1.31E+08	1.31E+08	+	0	0	0	9	0	6	4.210686	0.047643	0.97594	Up
circBUB1B(Ex.17-19)	chr15	40501836	40504849	+	0	0	0	3	12	0	4.237826	0.048	0.97594	Up
circTADA2A(Ex.11-14)	chr17	35825535	35831241	+	0	0	0	9	15	9	5.129146	0.011288	0.97594	Up
circHIF1A(Ex.3-7)	chr14	62188227	62199242	+	0	0	0	12	0	0	5.122885	0.021683	0.97594	Up
circOGA(Ex.7-8)	chr10	1.04E+08	1.04E+08	-	0	0	0	2	0	14	4.65263	0.025851	0.97594	Up
circIQGAP2(Ex.22-27)	chr5	75960851	75970534	+	0	0	0	5	10	0	4.436802	0.034606	0.97594	Up
circAPPL1(Ex.11-13)	chr3	57283388	57287766	+	0	0	0	16	5	0	4.958289	0.015687	0.97594	Up
circTICRR(Ex.14-18)	chr15	90149999	90163073	+	0	0	0	2	10	13	4.407394	0.039386	0.97594	Up
circDDX60L(Ex.32-37)	chr4	1.69E+08	1.69E+08	-	0	0	0	10	6	10	4.514717	0.030632	0.97594	Up
circFAM13B(Ex.2-8)	chr5	1.37E+08	1.37E+08	-	0	0	0	5	15	0	4.624231	0.04507	0.97594	Up
circLBR(Ex.9-11)	chr1	2.26E+08	2.26E+08	-	0	0	0	14	4	4	4.464114	0.020012	0.97594	Up
circLPIN2(Ex.2-3)	chr18	2954502	2960847	-	0	0	0	10	10	4	4.43221	0.035661	0.97594	Up
circPCM1(Ex.8-9)	chr8	17808124	17810695	+	0	0	0	20	16	3	5.679995	3.34E-04	0.97594	Up
circSLC22A16(Ex.4-5)	chr6	1.11E+08	1.11E+08	-	0	0	0	7	20	0	4.602407	0.047052	0.97594	Up
circKNL1(Ex.8-24)	chr15	40907539	40949671	+	0	0	0	10	6	0	4.675769	0.023396	0.97594	Up
circLRRC43(Ex.2-3)	chr12	1.23E+08	1.23E+08	+	0	0	0	7	0	10	5.370344	0.002879	0.97594	Up
circSRPRA(Ex.4-5)	chr11	1.26E+08	1.26E+08	-	0	0	0	21	5	10	5.180037	0.004289	0.97594	Up
circTFRC(Ex.4-12)	chr3	1.96E+08	1.96E+08	-	0	0	0	24	0	13	4.769062	0.036532	0.97594	Up
circWWP2(Ex.6-7)	chr16	69875927	69905834	+	0	0	0	24	7	0	4.621719	0.044934	0.97594	Up
circR3HDM2(Ex.13-13)	chr12	57677607	57677839	-	0	0	0	8	12	7	4.41423	0.037313	0.97594	Up
circPIGN(Ex.2-11)	chr18	59810539	59830889	-	0	0	0	14	6	6	4.42148	0.035858	0.97594	Up
circCLTC(Ex.2-4)	chr17	57721637	57725762	+	0	0	0	4	11	0	4.703877	0.01134	0.97594	Up
circSEC63(Ex.9-11)	chr6	1.08E+08	1.08E+08	-	0	0	0	0	9	0	4.928963	0.015316	0.97594	Up
circHECTD4(Ex.44-53)	chr12	1.13E+08	1.13E+08	-	0	0	0	0	13	0	4.830502	0.032663	0.97594	Up
circCLEC16A(Ex.20-21)	chr16	11214472	11217803	+	0	0	0	0	7	0	4.294148	0.042074	0.97594	Up

circDIAPH3(Ex.10-15)	chr13	60548486	60566717	-	0	0	0	0	26	0	5.035217	0.026044	0.97594	Up
circMLC1(Ex.4-7)	chr22	50515270	50518826	-	0	0	0	0	23	15	5.137337	0.022599	0.97594	Up
circDHX35(Ex.9-11)	chr20	37630373	37632550	+	0	0	0	0	23	7	4.782831	0.037689	0.97594	Up
circUSP36(Ex.6-13)	chr17	76808942	76823429	-	0	0	0	0	18	14	4.736696	0.040119	0.97594	Up
circSLC22A15(Ex.3-4)	chr1	1.17E+08	1.17E+08	+	0	0	0	0	9	19	4.605194	0.046014	0.97594	Up
circSTAM(Ex.10-12)	chr10	17746430	17747740	+	0	0	0	0	6	13	4.358028	0.041492	0.97594	Up
circSPECC1(Ex.4-4)	chr17	20107646	20109225	+	0	0	0	0	4	0	4.294097	0.043214	0.97594	Up
circRFC1(Ex.7-9)	chr4	39322003	39325037	-	0	0	0	0	7	0	4.83309	0.017745	0.97594	Up
circPCNX2(Ex.18-21)	chr1	2.33E+08	2.33E+08	-	0	0	0	0	6	14	4.554236	0.048799	0.97594	Up
circKMT2A(Ex.18-20)	chr11	1.18E+08	1.18E+08	+	0	0	0	0	3	12	4.293323	0.043592	0.97594	Up
circWDR41(Ex.4-8)	chr5	76745585	76759051	-	0	0	0	0	4	13	4.248542	0.047609	0.97594	Up
circPI4KA(Ex.8-9)	chr22	21165255	21167794	-	0	0	0	0	12	0	5.109001	0.022527	0.97594	Up
circSETD9(Ex.3-5)	chr5	56208838	56210793	+	0	0	0	0	6	0	4.70342	0.040742	0.97594	Up
circMIB1(Ex.4-6)	chr18	19353585	19359646	+	0	0	0	0	6	8	4.809102	0.018833	0.97594	Up
circFCHSD2(Ex.5-8)	chr11	72695133	72712179	-	0	0	0	0	7	0	4.210162	0.047528	0.97594	Up
circRPS6KC1(Ex.3-6)	chr1	2.13E+08	2.13E+08	+	0	0	0	0	16	10	4.901735	0.01664	0.97594	Up
circARHGEF12(Ex.20-25)	chr11	1.2E+08	1.2E+08	+	0	0	0	0	20	9	4.800246	0.03563	0.97594	Up
circPPM1A(Ex.2-2)	chr14	60749402	60750255	+	0	0	0	0	8	0	4.33702	0.039442	0.97594	Up
circUSP15(Ex.5-8)	chr12	62715245	62749256	+	0	0	0	0	8	22	4.732871	0.039208	0.97594	Up
circFLNB(Ex.5-7)	chr3	58080563	58083704	+	0	0	0	0	16	0	4.678202	0.043098	0.97594	Up
circMAP2K4(Ex.3-7)	chr17	11984673	12016677	+	0	0	0	0	14	18	5.048689	0.013204	0.97594	Up
circPPIP5K2(Ex.22-26)	chr5	1.03E+08	1.03E+08	+	0	0	0	0	6	0	4.744798	0.037621	0.97594	Up
circATR(Ex.19-27)	chr3	1.42E+08	1.42E+08	-	0	0	0	0	0	30	4.953468	0.047441	0.97594	Up
circNARS1(Ex.4-6)	chr18	55278869	55282962	-	0	0	0	0	0	15	4.575199	0.026906	0.97594	Up
circSWT1(Ex.8-15)	chr1	1.85E+08	1.85E+08	+	0	0	0	0	0	28	4.911632	0.04986	0.97594	Up
circIST1(Ex.2-8)	chr16	71949529	71957283	+	0	0	0	0	0	20	4.530075	0.049671	0.97594	Up
circPOC1B(Ex.5-10)	chr12	89853415	89866052	-	0	0	0	0	0	42	5.600308	0.011235	0.97594	Up
circKLHDC10(Ex.5-8)	chr7	1.3E+08	1.3E+08	+	0	0	0	0	0	12	4.626984	0.042842	0.97594	Up
circINTS7(Ex.2-9)	chr1	2.12E+08	2.12E+08	-	0	0	0	0	0	14	4.553217	0.028454	0.97594	Up
circDGKE(Ex.3-5)	chr17	54921380	54925426	+	0	0	0	0	0	12	5.024171	0.02608	0.97594	Up
circCERT1(Ex.9-14)	chr5	74680467	74698883	-	0	0	0	0	0	4	4.262113	0.045969	0.97594	Up
circTPP2(Ex.14-18)	chr13	1.03E+08	1.03E+08	+	0	0	0	0	0	33	4.812694	0.037305	0.97594	Up
circGABRR2(Ex.7-8)	chr6	89974131	89975484	-	0	0	0	0	0	0	4.600644	0.043875	0.97594	Up
circSETD5(Ex.12-17)	chr3	9486732	9495552	+	0	0	0	0	0	0	5.511543	0.024139	0.97594	Up
circUSP54(Ex.5-10)	chr10	75299202	75305430	-	0	0	0	0	0	0	6.23525	0.01004	0.97594	Up

GSE268499

name	chrom	start	end	strand	Normal_1	Normal_2	Normal_3	Normal_4	Normal_5	COPD_1	COPD_2	COPD_3	COPD_4	COPD_5	log2FoldCl	pval	padj	Expression
circFCHO2(Ex.17-21)	chr5	72354259	72373320	+	0	0	0	0	0	35.80251	39.87877	28.79023	0	0	6.82137	0.022648	1	Up
circPTPN22(Ex.4-7)	chr1	1.14E+08	1.14E+08	-	0	0	0	0	0	35.80251	39.87877	0	0	33.05457	6.869428	0.021838	1	Up
circIRAK3(Ex.2-6)	chr12	66597490	66611015	+	0	0	0	0	0	35.80251	0	57.58047	0	0	6.663796	0.027851	1	Up
circSLC39A8(Ex.6-9)	chr4	1.03E+08	1.03E+08	-	0	0	0	0	0	0	39.87877	0	0	66.10915	6.829618	0.024171	1	Up
circTBC1D22A(Ex.10-12)	chr22	47370185	47433094	+	0	0	0	0	0	0	39.87877	0	54.15945	0	6.686168	0.027328	1	Up
circMBOAT2(Ex.10-12)	chr2	9048750	9098771	-	0	0	0	0	0	71.60503	0	0	27.07972	0	6.739484	0.026115	1	Up
circACADM(Ex.2-5)	chr1	76198328	76200556	+	0	0	0	0	0	0	0	57.58047	54.15945	0	6.939403	0.021974	1	Up
circCKAP5(Ex.37-40)	chr11	46829580	46832728	-	0	0	0	0	0	35.80251	39.87877	0	27.07972	0	6.801438	0.02301	1	Up
circRNF213(Ex.19-21)	chr17	78298829	78302277	+	71.3708	29.16727	0	0	0	0	0	0	0	0	-6.76103	0.02564	1	Down
circBNC2(Ex.5-6)	chr9	16727794	16738483	-	0	0	57.30823	31.45149	0	0	0	0	0	0	-6.59798	0.029443	1	Down
circMALT1(Ex.3-4)	chr18	56363597	56367823	+	0	58.33455	0	0	31.22366	0	0	0	0	0	-6.60309	0.029317	1	Down
circMYBL1(Ex.5-6)	chr8	67484716	67485741	-	47.58053	0	0	62.90297	0	0	0	0	0	0	-6.91556	0.022437	1	Down
circRNF34(Ex.2-3)	chr12	1.22E+08	1.22E+08	+	23.79027	0	28.65412	0	31.22366	0	0	0	0	0	-6.52148	0.027578	1	Down
circRBM5(Ex.4-10)	chr3	50131152	50143142	+	47.58053	0	0	31.45149	31.22366	0	0	0	0	0	-6.90238	0.021364	1	Down
circKIF11(Ex.11-12)	chr10	94388564	94390121	+	0	58.33455	57.30823	0	0	0	0	0	0	0	-6.98148	0.02118	1	Down
circRNPEP(Ex.4-5)	chr1	2.02E+08	2.02E+08	+	23.79027	0	0	62.90297	0	0	0	0	0	0	-6.56636	0.030234	1	Down
circNCAPD3(Ex.4-8)	chr11	1.34E+08	1.34E+08	-	0	0	0	0	0	0	0	28.79023	54.15945	0	6.512125	0.031632	1	Up
circUGGT2(Ex.30-31)	chr13	96515883	96519677	-	380.6442	350.0073	601.7364	157.2574	281.0129	179.0126	79.75754	201.5316	135.3986	264.4366	-1.05431	0.030292	0.354147	Down
circACSL6(Ex.12-17)	chr5	1.31E+08	1.31E+08	-	237.9027	175.0036	544.4282	251.6119	0	35.80251	39.87877	0	27.07972	66.10915	-2.85593	0.038708	0.442256	Down
circATG4C(Ex.4-10)	chr1	63282246	63307218	+	0	0	0	0	0	71.60503	0	0	0	33.05457	6.810385	0.024575	1	Up
circSLC14A1(Ex.5-9)	chr18	43314239	43328390	+	0	0	0	0	0	0	79.75754	28.79023	0	0	6.87997	0.02314	1	Up
circANKRD26(Ex.17-23)	chr10	27326115	27337908	-	689.9177	320.84	515.7741	691.9327	124.8946	358.0251	119.6363	316.6926	135.3986	33.05457	-1.29104	0.048968	0.549084	Down
circHECTD4(Ex.8-13)	chr12	1.13E+08	1.13E+08	-	71.3708	29.16727	0	0	0	0	0	0	0	0	-6.76103	0.02564	1	Down
circCHD9(Ex.18-19)	chr16	53288350	53289691	+	0	0	0	0	0	0	79.75754	28.79023	0	0	6.87997	0.02314	1	Up
circZNF175(Ex.3-4)	chr19	52084644	52085135	+	0	0	0	0	0	0	0	57.58047	27.07972	0	6.536456	0.030997	1	Up
circFARSB(Ex.2-15)	chr2	2.23E+08	2.24E+08	-	23.79027	0	85.96235	0	0	0	0	0	0	0	-6.91911	0.022368	1	Down
circABCG2(Ex.10-12)	chr4	89020476	89028418	-	23.79027	0	0	62.90297	0	0	0	0	0	0	-6.56636	0.030234	1	Down
circHOOK3(Ex.4-8)	chr8	42785265	42814457	+	0	0	0	0	0	0	0	0	54.15945	33.05457	6.571453	0.030103	1	Up
circSLCO4C1(Ex.2-8)	chr5	1.02E+08	1.02E+08	-	0	0	0	0	0	0	0	0	54.15945	33.05457	6.571453	0.030103	1	Up
circAGFG2(Ex.2-6)	chr7	1E+08	1E+08	+	0	0	57.30823	0	31.22366	0	0	0	0	0	-6.59789	0.029445	1	Down
circBAZ1B(Ex.2-3)	chr7	72922657	72925182	-	0	0	0	0	0	35.80251	39.87877	28.79023	0	0	6.82137	0.022648	1	Up
circDOCK7(Ex.13-14)	chr1	63084377	63085664	-	0	0	0	0	0	0	0	57.58047	27.07972	0	6.536456	0.030997	1	Up
circCCDC186(Ex.5-6)	chr10	1.16E+08	1.16E+08	-	47.58053	29.16727	28.65412	0	0	0	0	0	0	0	-6.85356	0.022181	1	Down
circTNFAIP3(Ex.2-3)	chr6	1.38E+08	1.38E+08	+	71.3708	29.16727	0	31.45149	62.44731	214.8151	159.5151	115.1609	162.4783	264.4366	2.242364	0.020953	0.249808	Up
circTACC3(Ex.5-8)	chr4	1732614	1737561	+	47.58053	29.16727	0	0	0	0	0	0	0	0	-6.38524	0.035129	1	Down
circCYTH1(Ex.8-11)	chr17	76688499	76695050	-	0	0	0	0	0	0	39.87877	0	27.07972	33.05457	6.759681	0.023694	1	Up
circHELQ(Ex.6-12)	chr4	84350677	84364767	-	0	0	0	0	0	0	39.87877	57.58047	0	0	6.729009	0.02635	1	Up
circAKAP13(Ex.12-14)	chr15	86201768	86213061	+	118.9513	145.8364	171.9247	31.45149	187.3419	644.4452	358.9089	115.1609	379.1161	132.2183	1.305068	0.031997	0.373599	Up
circWASHC4(Ex.20-23)	chr12	1.06E+08	1.06E+08	+	0	0	0	0	0	35.80251	0	0	54.15945	0	6.618938	0.028926	1	Up
circKAT2B(Ex.3-5)	chr3	20136755	20142960	+	0	0	0	0	0	35.80251	0	0	54.15945	0	6.618938	0.028926	1	Up
circTRIM37(Ex.13-14)	chr17	57128575	57134415	-	0	0	0	0	0	0	79.75754	0	27.07972	0	6.86084	0.023527	1	Up
circUSP24(Ex.56-67)	chr1	55537487	55551711	-	0	29.16727	0	62.90297	0	0	0	0	0	0	-6.65098	0.028157	1	Down
circXPA(Ex.2-5)	chr9	1E+08	1E+08	-	23.79027	58.33455	0	0	0	0	0	0	0	0	-6.4808	0.032468	1	Down
circPICALM(Ex.9-17)	chr11	85692172	85714494	-	0	0	0	0	0	0	0	0	81.23917	33.05457	6.965952	0.021469	1	Up
circCLOCK(Ex.16-18)	chr4	56314946	56316399	-	71.3708	0	0	0	31.22366	0	0	0	0	0	-6.78969	0.025018	1	Down
circRIF1(Ex.15-20)	chr2	1.52E+08	1.52E+08	+	0	0	0	0	0	35.80251	0	28.79023	27.07972	0	6.641543	0.02564	1	Up
circABCB7(Ex.10-15)	chrX	74280058	74290357	-	23.79027	29.16727	0	0	31.22366	0	0	0	0	0	-6.51554	0.027659	1	Down
circITGA4(Ex.12-17)	chr2	1.82E+08	1.82E+08	+	0	0	0	0	0	0	0	28.79023	54.15945	33.05457	6.983745	0.020214	1	Up
circNUP155(Ex.28-32)	chr5	37298970	37303516	-	0	0	0	62.90297	31.22366	0	0	0	0	0	-6.68189	0.027429	1	Down
circVPS45(Ex.11-14)	chr1	1.5E+08	1.5E+08	+	47.58053	0	0	0	31.22366	0	0	0	0	0	-6.42232	0.034075	1	Down
circFOXP3(Ex.2-6)	chrX	49113208	49114984	-	0	0	0	0	0	0	79.75754	28.79023	0	0	6.87997	0.02314	1	Up
circDZANK1(Ex.3-7)	chr20	18429628	18440950	-	0	0	0	0	0	35.80251	0	0	0	66.10915	6.768892	0.025466	1	Up
circECPAS(Ex.7-10)	chr9	1.14E+08	1.14E+08	-	0	0	0	0	0	71.60503	0	0	0	33.05457	6.810385	0.024575	1	Up
circBPTF(Ex.8-9)	chr17	65899905	65900956	+	23.79027	0	0	62.90297	0	0	0	0	0	0	-6.56636	0.030234	1	Down
circATP6V0A1(Ex.17-20)	chr17	40653215	40665996	+	23.79027	29.16727	0	0	31.22366	0	0	0	0	0	-6.51554	0.027659	1	Down

circORAI2(Ex.2-3)	chr7	1.02E+08	1.02E+08	+	475.8053	291.6727	343.8494	157.2574	343.4602	107.4075	159.5151	57.58047	216.6378	33.05457	-1.48533	0.010183	0.171436	Down
circGDAP2(Ex.3-6)	chr1	1.18E+08	1.18E+08	-	0	0	0	0	0	35.80251	0	0	0	66.10915	6.768892	0.025466	1	Up
circMMACHC(Ex.2-3)	chr1	45973028	45974036	+	0	0	0	0	0	0	0	28.79023	0	66.10915	6.672999	0.027635	1	Up
circFOXM1(Ex.4-8)	chr12	2973486	2977920	-	0	0	0	0	0	35.80251	0	28.79023	0	33.05457	6.717306	0.024321	1	Up
circMTR(Ex.13-16)	chr1	2.37E+08	2.37E+08	+	0	0	0	0	0	0	0	0	81.23917	33.05457	6.965952	0.021469	1	Up
circKCNK5(Ex.2-4)	chr6	39161945	39163763	-	0	0	0	0	0	0	39.87877	0	54.15945	0	6.686168	0.027328	1	Up
circEAF2(Ex.3-4)	chr3	1.22E+08	1.22E+08	+	0	0	0	0	0	71.60503	0	28.79023	0	0	6.760284	0.025655	1	Up
circCCDC25(Ex.5-7)	chr8	27605594	27610104	-	47.58053	0	0	0	31.22366	0	0	0	0	0	-6.42232	0.034075	1	Down
circUSP9X(Ex.28-30)	chrX	41055845	41058003	+	71.3708	58.33455	28.65412	157.2574	124.8946	0	0	0	27.07972	0	-3.99889	0.048069	0.541019	Down
circLIN54(Ex.5-12)	chr4	83852096	83867627	-	23.79027	29.16727	28.65412	0	0	0	0	0	0	0	-6.48689	0.02815	1	Down
circGIT2(Ex.2-6)	chr12	1.1E+08	1.1E+08	-	0	0	0	0	0	35.80251	39.87877	28.79023	0	0	6.82137	0.022648	1	Up
circASAP1(Ex.7-9)	chr8	1.31E+08	1.31E+08	-	0	0	0	0	0	0	39.87877	0	54.15945	0	6.686168	0.027328	1	Up
circCATSPERB(Ex.6-9)	chr14	92159470	92174580	-	0	29.16727	0	0	62.44731	143.2101	279.1514	115.1609	54.15945	165.2729	3.042617	0.042046	0.478589	Up
circTECTA(Ex.8-10)	chr11	1.21E+08	1.21E+08	+	0	0	57.30823	31.45149	0	0	0	0	0	0	-6.59798	0.029443	1	Down
circMETTL14(Ex.6-9)	chr4	1.2E+08	1.2E+08	+	47.58053	29.16727	28.65412	0	0	0	0	0	0	0	-6.85356	0.022181	1	Down
circIWS1(Ex.8-10)	chr2	1.28E+08	1.28E+08	-	285.4832	87.50182	200.5788	345.9663	281.0129	107.4075	39.87877	143.9512	81.23917	132.2183	-1.26053	0.036639	0.421278	Down
circGOLGA1(Ex.13-14)	chr9	1.28E+08	1.28E+08	-	23.79027	58.33455	28.65412	0	0	0	0	0	0	0	-6.92326	0.021244	1	Down
circBRCA1(Ex.16-21)	chr17	41201138	41219712	-	547.1761	466.6764	85.96235	251.6119	437.1312	286.4201	119.6363	57.58047	81.23917	132.2183	-1.41178	0.014345	0.173928	Down
circPARD3B(Ex.19-20)	chr2	2.06E+08	2.06E+08	+	71.3708	29.16727	0	0	0	0	0	0	0	0	-6.76103	0.02564	1	Down
circFRY(Ex.51-59)	chr13	32828361	32863880	+	0	0	0	0	0	0	39.87877	0	54.15945	0	6.686168	0.027328	1	Up
circNAP1L4(Ex.3-6)	chr11	2992678	2999572	-	0	0	0	0	0	71.60503	0	0	0	33.05457	6.810385	0.024575	1	Up
circCREBBP(Ex.17-21)	chr16	3799628	3808973	-	23.79027	0	28.65412	0	62.44731	0	0	0	0	0	-6.9741	0.020488	1	Down
circMAP4K5(Ex.2-13)	chr14	50929383	50998876	-	0	0	0	0	0	0	0	28.79023	54.15945	0	6.512125	0.031632	1	Up
circPDHX(Ex.6-7)	chr11	34988187	34991833	+	0	0	0	0	0	0	39.87877	0	54.15945	0	6.686168	0.027328	1	Up
circCLEC16A(Ex.20-21)	chr16	11214472	11217803	+	47.58053	0	28.65412	0	0	0	0	0	0	0	-6.39174	0.034942	1	Down
circNLRC5(Ex.16-38)	chr16	57073693	57104530	+	23.79027	0	85.96235	0	0	0	0	0	0	0	-6.91911	0.022368	1	Down
circUSP24(Ex.33-36)	chr1	55589120	55591431	-	47.58053	0	57.30823	0	0	0	0	0	0	0	-6.84445	0.023865	1	Down
circSLC25A32(Ex.2-3)	chr8	1.04E+08	1.04E+08	-	0	0	28.65412	0	0	71.60503	119.6363	143.9512	135.3986	66.10915	4.191989	0.024499	0.289416	Up
circPPP2R3C(Ex.5-10)	chr14	35564254	35577442	-	214.1124	641.68	515.7741	220.1604	218.5656	107.4075	119.6363	143.9512	324.9567	66.10915	-1.24806	0.022528	0.267176	Down
circMTPAP(Ex.2-3)	chr10	30629155	30630569	-	0	0	0	0	0	35.80251	0	28.79023	0	33.05457	6.717306	0.024321	1	Up
circWDFY3(Ex.52-58)	chr4	85617123	85630500	-	23.79027	0	28.65412	31.45149	0	0	0	0	0	0	-6.52158	0.027577	1	Down
circNIPBL(Ex.2-5)	chr5	36953720	36961685	+	71.3708	29.16727	0	0	0	0	0	0	0	0	-6.76103	0.02564	1	Down
circHEATR5A(Ex.6-9)	chr14	31855626	31863432	-	0	58.33455	57.30823	0	0	0	0	0	0	0	-6.98148	0.02118	1	Down
circXRN1(Ex.37-40)	chr3	1.42E+08	1.42E+08	-	47.58053	0	0	0	62.44731	0	0	0	0	0	-6.90231	0.022697	1	Down
circCAB39(Ex.3-6)	chr2	2.32E+08	2.32E+08	+	0	29.16727	28.65412	0	31.22366	0	0	0	0	0	-6.60869	0.026107	1	Down
circNR1H2(Ex.3-9)	chr19	50880844	50885431	+	333.0637	408.3418	916.9317	471.7723	218.5656	71.60503	119.6363	345.4828	135.3986	99.16372	-1.61523	0.001759	0.099707	Down
circMYO1F(Ex.14-16)	chr19	8604831	8609348	-	0	29.16727	0	0	62.44731	0	0	0	0	0	-6.63503	0.028538	1	Down
circGPATCH2L(Ex.6-9)	chr14	76642966	76662315	+	23.79027	29.16727	28.65412	0	0	0	0	0	0	0	-6.48689	0.02815	1	Down
circRBBP8(Ex.15-17)	chr18	20581549	20596887	+	0	0	0	0	0	35.80251	0	0	54.15945	0	6.618938	0.028926	1	Up
circTTC13(Ex.2-12)	chr1	2.31E+08	2.31E+08	-	0	0	0	0	0	0	79.75754	0	27.07972	0	6.86084	0.023527	1	Up
circDDX6(Ex.2-4)	chr11	1.19E+08	1.19E+08	-	47.58053	0	0	0	31.22366	0	0	0	0	0	-6.42232	0.034075	1	Down
circBABAM1(Ex.2-8)	chr19	17379603	17387718	+	23.79027	0	57.30823	0	0	0	0	0	0	0	-6.47513	0.032621	1	Down
circWDR26(Ex.7-12)	chr1	2.25E+08	2.25E+08	-	0	0	0	0	0	35.80251	0	0	0	66.10915	6.768892	0.025466	1	Up
circABHD3(Ex.6-8)	chr18	19236822	19239304	-	71.3708	0	28.65412	0	0	0	0	0	0	0	-6.76605	0.02553	1	Down
circCNOT1(Ex.15-16)	chr16	58608513	58609033	-	0	29.16727	0	62.90297	0	0	0	0	0	0	-6.65098	0.028157	1	Down
circERCC6L2(Ex.7-12)	chr9	98683457	98703831	+	0	0	0	0	0	0	0	28.79023	27.07972	33.05457	6.59481	0.02641	1	Up
circTTLL4(Ex.13-16)	chr2	2.2E+08	2.2E+08	+	0	0	0	0	0	0	39.87877	0	0	66.10915	6.829618	0.024171	1	Up
circATAD2B(Ex.2-7)	chr2	24103509	24118840	-	0	58.33455	28.65412	0	0	0	0	0	0	0	-6.57615	0.029987	1	Down
circFAH(Ex.6-13)	chr15	80460394	80473501	+	0	0	0	0	0	0	0	0	54.15945	33.05457	6.571453	0.030103	1	Up
circATP11B(Ex.8-10)	chr3	1.83E+08	1.83E+08	+	0	0	0	0	0	71.60503	0	0	27.07972	0	6.739484	0.026115	1	Up
circACTR3(Ex.7-8)	chr2	1.15E+08	1.15E+08	+	0	0	57.30823	31.45149	0	0	0	0	0	0	-6.59798	0.029443	1	Down
circDIPK2A(Ex.2-2)	chr3	1.44E+08	1.44E+08	+	0	0	0	0	0	71.60503	0	0	0	33.05457	6.810385	0.024575	1	Up
circDHX37(Ex.19-21)	chr12	1.25E+08	1.25E+08	-	0	0	28.65412	0	62.44731	0	0	0	0	0	-6.64051	0.028407	1	Down
circHNRNPA2B1(Ex.5-10)	chr7	26232115	26236278	-	0	58.33455	57.30823	0	0	0	0	0	0	0	-6.98148	0.02118	1	Down
circSF3B3(Ex.16-18)	chr16	70594372	70597953	+	23.79027	29.16727	0	31.45149	0	0	0	0	0	0	-6.51563	0.027658	1	Down
circNETO2(Ex.2-6)	chr16	47156568	47165936	-	0	0	0	0	0	71.60503	0	28.79023	0	0	6.760284	0.025655	1	Up

circFBXO21(Ex.4-7)	chr12	1.18E+08	1.18E+08	-	0	0	0	0	0	0	0	57.58047	54.15945	0	6.939403	0.021974	1 Up
circCFLAR(Ex.9-9)	chr2	2.02E+08	2.02E+08	+	261.6929	58.33455	85.96235	62.90297	93.67097	0	0	28.79023	0	0	-4.27776	0.020601	0.246258 Down
circOBI1(Ex.2-3)	chr13	79216257	79219132	-	47.58053	58.33455	0	0	0	0	0	0	0	0	-6.84883	0.023775	1 Down
circWRN(Ex.28-31)	chr8	31001066	31007971	+	0	0	0	0	0	0	0	0	54.15945	33.05457	6.571453	0.030103	1 Up
circTCF25(Ex.6-9)	chr16	89958601	89962491	+	0	0	0	0	0	0	79.75754	0	27.07972	0	6.86084	0.023527	1 Up
circSLC35F5(Ex.8-15)	chr2	1.14E+08	1.14E+08	-	0	29.16727	0	0	62.44731	0	0	0	0	0	-6.63503	0.028538	1 Down
circFAM117A(Ex.6-7)	chr17	47793527	47795076	-	0	0	0	0	0	0	0	0	54.15945	33.05457	6.571453	0.030103	1 Up
circMPP1(Ex.5-9)	chrX	1.54E+08	1.54E+08	-	285.4832	58.33455	143.2706	283.0634	187.3419	71.60503	39.87877	57.58047	135.3986	33.05457	-1.49647	0.042514	0.483307 Down
circMTBP(Ex.6-11)	chr8	1.21E+08	1.21E+08	+	0	0	0	0	0	0	0	57.58047	54.15945	0	6.939403	0.021974	1 Up
circELMOD3(Ex.4-11)	chr2	85584090	85604597	+	47.58053	0	28.65412	31.45149	0	0	0	0	0	0	-6.88053	0.021736	1 Down
circFANCI(Ex.24-27)	chr15	89838146	89844673	+	0	58.33455	0	31.45149	0	0	0	0	0	0	-6.60318	0.029315	1 Down
circATP10A(Ex.16-17)	chr15	25928433	25932975	-	0	0	0	31.45149	62.44731	286.4201	199.3938	86.3707	27.07972	330.5457	3.303034	0.044567	0.505367 Up
circVPS13B(Ex.2-3)	chr8	1E+08	1E+08	+	71.3708	0	0	0	31.22366	0	0	0	0	0	-6.78969	0.025018	1 Down
circGRIPAP1(Ex.2-11)	chrX	48846023	48855916	-	0	0	0	0	0	0	0	0	81.23917	33.05457	6.965952	0.021469	1 Up
circIQGAP2(Ex.33-34)	chr5	75993812	75997038	+	0	0	0	0	0	0	39.87877	0	0	66.10915	6.829618	0.024171	1 Up
circUSP34(Ex.78-79)	chr2	61416045	61417549	-	0	0	0	0	0	0	39.87877	28.79023	0	33.05457	6.780195	0.02332	1 Up
circKMT2A(Ex.17-23)	chr11	1.18E+08	1.18E+08	+	0	0	0	0	0	0	0	57.58047	0	33.05457	6.617791	0.028954	1 Up
circGLT1D1(Ex.5-10)	chr12	1.29E+08	1.29E+08	+	47.58053	0	57.30823	0	0	393.8276	119.6363	115.1609	162.4783	165.2729	3.172871	0.045637	0.51677 Up
circEP300(Ex.4-8)	chr22	41523491	41533794	+	0	58.33455	28.65412	0	0	0	0	0	0	0	-6.57615	0.029987	1 Down
circPIGN(Ex.3-4)	chr18	59828366	59829562	-	0	0	0	0	0	0	0	0	27.07972	66.10915	6.650889	0.028157	1 Up
circFBXL4(Ex.3-7)	chr6	99347144	99375698	-	23.79027	58.33455	0	0	0	0	0	0	0	0	-6.4808	0.032468	1 Down
circHERC5(Ex.5-12)	chr4	89384683	89397181	+	0	0	0	0	0	0	0	0	27.07972	66.10915	6.650889	0.028157	1 Up
circCEP350(Ex.7-9)	chr1	1.8E+08	1.8E+08	+	47.58053	0	0	0	62.44731	0	0	0	0	0	-6.90231	0.022697	1 Down
circCAST(Ex.26-29)	chr5	96100925	96103703	+	47.58053	0	0	0	31.22366	0	0	0	0	0	-6.42232	0.034075	1 Down
circKDM5A(Ex.11-27)	chr12	401925	443588	-	0	0	0	0	0	0	0	28.79023	0	66.10915	6.672999	0.027635	1 Up
circANKRD42(Ex.3-8)	chr11	82917100	82947551	+	190.3221	379.1746	372.5035	377.4178	530.8021	143.2101	39.87877	230.3219	270.7972	132.2183	-1.18301	0.022998	0.272399 Down
circTHADA(Ex.2-7)	chr2	43813540	43819555	-	0	0	0	0	0	0	79.75754	28.79023	0	0	6.87997	0.02314	1 Up
circTOP1(Ex.9-16)	chr20	39721112	39744079	+	0	0	28.65412	31.45149	31.22366	0	0	0	0	0	-6.6406	0.025575	1 Down
circHACE1(Ex.18-19)	chr6	1.05E+08	1.05E+08	-	142.7416	233.3382	28.65412	62.90297	62.44731	0	0	28.79023	0	0	-4.19177	0.027867	0.327493 Down
circKIAA0319(Ex.5-9)	chr6	24578338	24583930	-	118.9513	350.0073	315.1953	220.1604	124.8946	429.6302	199.3938	547.0145	785.312	396.6549	1.058611	0.02134	0.254081 Up
circMALT1(Ex.5-6)	chr18	56376610	56377304	+	0	58.33455	28.65412	0	0	0	0	0	0	0	-6.57615	0.029987	1 Down
circGALNT2(Ex.8-10)	chr1	2.3E+08	2.3E+08	+	0	0	0	0	0	71.60503	39.87877	0	0	0	6.908163	0.02258	1 Up
circLRIG1(Ex.4-7)	chr3	66460553	66467690	-	0	0	0	31.45149	62.44731	0	0	0	0	0	-6.66637	0.027792	1 Down
circFRMD3(Ex.2-6)	chr9	85950431	86004623	-	0	58.33455	57.30823	0	0	0	0	0	0	0	-6.98148	0.02118	1 Down
circRAD21(Ex.8-9)	chr8	1.18E+08	1.18E+08	-	23.79027	87.50182	0	0	0	0	0	0	0	0	-6.93173	0.022123	1 Down
circTLE4(Ex.14-17)	chr9	82324538	82336803	+	47.58053	29.16727	0	0	0	0	0	0	0	0	-6.38524	0.035129	1 Down
circABR(Ex.6-14)	chr17	959275	982630	-	23.79027	0	28.65412	31.45149	0	0	0	0	0	0	-6.52158	0.027577	1 Down
circHERC1(Ex.9-13)	chr15	64026923	64041990	-	23.79027	0	0	62.90297	0	0	0	0	0	0	-6.56636	0.030234	1 Down
circPRIM1(Ex.9-11)	chr12	57132218	57135360	-	23.79027	58.33455	0	0	0	0	0	0	0	0	-6.4808	0.032468	1 Down
circWIPI1(Ex.7-10)	chr17	66424970	66430767	-	47.58053	0	57.30823	0	0	0	0	0	0	0	-6.84445	0.023865	1 Down
circUGGT1(Ex.6-14)	chr2	1.29E+08	1.29E+08	+	23.79027	0	28.65412	0	62.44731	0	0	0	0	0	-6.9741	0.020488	1 Down
circLMF1(Ex.4-6)	chr16	929570	961079	-	23.79027	0	28.65412	0	31.22366	0	0	0	0	0	-6.52148	0.027578	1 Down
circITPR2(Ex.53-54)	chr12	26551809	26553191	-	404.4345	87.50182	401.1576	471.7723	156.1183	179.0126	39.87877	57.58047	189.5581	66.10915	-1.5158	0.017639	0.211693 Down
circUSP9X(Ex.33-34)	chrX	41069762	41073962	+	23.79027	262.5055	85.96235	691.9327	343.4602	107.4075	0	28.79023	54.15945	66.10915	-2.47074	0.02541	0.299397 Down
circHERC5(Ex.12-16)	chr4	89396992	89410487	+	618.5469	291.6727	85.96235	251.6119	156.1183	214.8151	79.75754	28.79023	81.23917	132.2183	-1.39516	0.034912	0.403983 Down
circRAB3GAP2(Ex.15-19)	chr1	2.2E+08	2.2E+08	-	0	0	0	0	0	35.80251	0	57.58047	0	0	6.663796	0.027851	1 Up
circGIGYF2(Ex.16-18)	chr2	2.34E+08	2.34E+08	+	0	0	0	0	0	0	79.75754	28.79023	0	0	6.87997	0.02314	1 Up
circHERC6(Ex.17-20)	chr4	89352314	89358933	+	0	0	0	0	0	0	39.87877	0	54.15945	0	6.686168	0.027328	1 Up
circDOP1B(Ex.27-32)	chr21	37642311	37653909	+	47.58053	0	28.65412	0	0	0	0	0	0	0	-6.39174	0.034942	1 Down
circTRMT2B(Ex.10-13)	chrX	1E+08	1E+08	-	0	0	0	0	0	0	0	0	54.15945	33.05457	6.571453	0.030103	1 Up
circANK1(Ex.29-31)	chr8	41550166	41551620	-	23.79027	0	0	62.90297	0	0	0	0	0	0	-6.56636	0.030234	1 Down
circWDR43(Ex.6-11)	chr2	29140759	29152576	+	47.58053	0	28.65412	0	0	0	0	0	0	0	-6.39174	0.034942	1 Down
circDYNC1H1(Ex.63-69)	chr14	1.03E+08	1.03E+08	+	0	0	0	0	0	0	39.87877	0	27.07972	33.05457	6.759681	0.023694	1 Up
circZNF280D(Ex.11-13)	chr15	56968868	56971019	-	0	0	0	0	0	0	79.75754	0	0	33.05457	6.926145	0.022229	1 Up
circCNOT10(Ex.6-7)	chr3	32757717	32758729	+	0	0	0	0	0	0	0	0	27.07972	66.10915	6.650889	0.028157	1 Up
circEXOC5(Ex.2-3)	chr14	57713429	57714430	-	0	0	0	0	0	0	0	57.58047	0	33.05457	6.617791	0.028954	1 Up

circDNM3(Ex.15-16)	chr1	1.72E+08	1.72E+08	+	0	0	0	0	0	35.80251	0	57.58047	0	0	6.663796	0.027851	1	Up
circHNRNPL(Ex.2-6)	chr19	39334482	39338074	-	333.0637	262.5055	257.887	314.5149	249.7892	393.8276	797.5754	1036.448	568.6742	727.2006	1.306386	4.33E-05	0.003561	Up
circRUFY2(Ex.2-3)	chr10	70161377	70164601	-	47.58053	0	0	62.90297	0	0	0	0	0	0	-6.91556	0.022437	1	Down
circR3HDM1(Ex.13-16)	chr2	1.36E+08	1.36E+08	+	0	58.33455	0	31.45149	0	0	0	0	0	0	-6.60318	0.029315	1	Down
circNUP153(Ex.4-7)	chr6	17669524	17675599	-	0	0	0	0	0	0	0	0	27.07972	66.10915	6.650889	0.028157	1	Up
circPRKCB(Ex.8-9)	chr16	24124294	24135302	+	0	29.16727	57.30823	0	0	0	0	0	0	0	-6.5651	0.030266	1	Down
circDNMT1(Ex.10-17)	chr19	10270334	10279040	-	0	0	0	0	0	71.60503	0	28.79023	0	0	6.760284	0.025655	1	Up
circALDH18A1(Ex.12-13)	chr10	97376234	97381008	-	0	0	0	0	0	0	0	28.79023	54.15945	33.05457	6.983745	0.020214	1	Up
circCDC14A(Ex.13-15)	chr1	1.01E+08	1.01E+08	+	118.9513	0	57.30823	31.45149	93.67097	250.6176	239.2726	287.9023	216.6378	132.2183	1.901424	0.034181	0.396036	Up
circNCF2(Ex.7-14)	chr1	1.84E+08	1.84E+08	-	23.79027	58.33455	0	0	0	0	0	0	0	0	-6.4808	0.032468	1	Down
circAOAH(Ex.5-12)	chr7	36633945	36677516	-	23.79027	29.16727	28.65412	0	0	0	0	0	0	0	-6.48689	0.02815	1	Down
circSLC6A8(Ex.2-7)	chrX	1.53E+08	1.53E+08	+	0	58.33455	28.65412	0	0	0	0	0	0	0	-6.57615	0.029987	1	Down
circMIGA1(Ex.3-7)	chr1	78267016	78280916	+	0	0	0	0	0	0	0	57.58047	54.15945	0	6.939403	0.021974	1	Up
circKLHDC1(Ex.2-8)	chr14	50175877	50196266	+	0	58.33455	0	0	31.22366	0	0	0	0	0	-6.60309	0.029317	1	Down
circSPIDR(Ex.12-14)	chr8	48612965	48614577	+	47.58053	0	0	0	62.44731	0	0	0	0	0	-6.90231	0.022697	1	Down
circEFCAB14(Ex.7-7)	chr1	47154025	47154216	-	0	0	28.65412	62.90297	0	0	0	0	0	0	-6.65639	0.028028	1	Down
circEPSTI1(Ex.8-10)	chr13	43469145	43491760	-	0	0	0	0	0	0	39.87877	57.58047	0	0	6.729009	0.02635	1	Up
circTPMT(Ex.3-8)	chr6	18132364	18148146	-	0	0	0	0	0	35.80251	0	0	0	66.10915	6.768892	0.025466	1	Up
circGAPVD1(Ex.16-24)	chr9	1.28E+08	1.28E+08	+	0	0	0	0	0	35.80251	79.75754	0	0	0	6.963409	0.021517	1	Up
circANKRA2(Ex.4-7)	chr5	72850147	72853465	-	0	0	0	62.90297	31.22366	0	0	0	0	0	-6.68189	0.027429	1	Down
circSOAT1(Ex.5-9)	chr1	1.79E+08	1.79E+08	+	47.58053	0	57.30823	0	0	0	0	0	0	0	-6.84445	0.023865	1	Down
circWDHD1(Ex.23-25)	chr14	55411050	55423922	-	0	0	0	0	0	0	0	57.58047	0	33.05457	6.617791	0.028954	1	Up
circCLSPN(Ex.15-20)	chr1	36204737	36212593	-	71.3708	29.16727	0	0	0	0	0	0	0	0	-6.76103	0.02564	1	Down
circPIGN(Ex.22-30)	chr18	59739906	59768416	-	23.79027	0	85.96235	0	0	0	0	0	0	0	-6.91911	0.022368	1	Down
circZMYM2(Ex.19-23)	chr13	20638591	20657172	+	23.79027	0	28.65412	0	31.22366	0	0	0	0	0	-6.52148	0.027578	1	Down
circPCM1(Ex.19-20)	chr8	17823508	17823982	+	0	0	0	0	0	0	0	57.58047	27.07972	0	6.536456	0.030997	1	Up
circHIF1A(Ex.3-7)	chr14	62188227	62199242	+	0	29.16727	0	31.45149	31.22366	0	0	0	0	0	-6.63513	0.02565	1	Down
circCCDC150(Ex.3-13)	chr2	1.98E+08	1.98E+08	+	0	0	0	0	0	0	0	28.79023	0	66.10915	6.672999	0.027635	1	Up
circPAPPA(Ex.18-21)	chr9	1.19E+08	1.19E+08	+	47.58053	291.6727	28.65412	125.8059	31.22366	35.80251	0	0	0	0	-3.89253	0.045744	0.51677	Down
circSCAP(Ex.2-3)	chr3	47476498	47484581	-	23.79027	29.16727	0	0	62.44731	0	0	0	0	0	-6.96975	0.020546	1	Down
circAGO2(Ex.12-14)	chr8	1.42E+08	1.42E+08	-	0	58.33455	28.65412	0	0	0	0	0	0	0	-6.57615	0.029987	1	Down
circMSRB3(Ex.3-5)	chr12	65720606	65762806	+	0	0	0	0	0	0	79.75754	28.79023	0	0	6.87997	0.02314	1	Up
circBCAT1(Ex.8-10)	chr12	24982757	24989530	-	0	0	0	0	0	0	0	57.58047	27.07972	0	6.536456	0.030997	1	Up
circFARS2(Ex.2-3)	chr6	5368783	5404934	+	47.58053	0	0	0	62.44731	0	0	0	0	0	-6.90231	0.022697	1	Down
circARFGAP2(Ex.6-10)	chr11	47192977	47195391	-	0	0	0	0	0	35.80251	0	0	54.15945	0	6.618938	0.028926	1	Up
circOXCT1(Ex.9-16)	chr5	41739492	41805783	-	0	0	0	0	0	35.80251	0	0	27.07972	33.05457	6.69587	0.024712	1	Up
circTRAK2(Ex.2-7)	chr2	2.02E+08	2.02E+08	-	0	0	0	0	0	35.80251	39.87877	0	27.07972	0	6.801438	0.02301	1	Up
circSDCBP(Ex.2-7)	chr8	59477578	59492353	+	47.58053	0	0	31.45149	31.22366	0	0	0	0	0	-6.90238	0.021364	1	Down
circKRBOX5(Ex.2-4)	chr16	31733947	31765221	+	0	0	0	0	0	0	39.87877	0	0	66.10915	6.829618	0.024171	1	Up
circSETDB1(Ex.2-2)	chr1	1.51E+08	1.51E+08	+	0	0	0	0	0	0	0	57.58047	27.07972	0	6.536456	0.030997	1	Up
circALDH16A1(Ex.2-4)	chr19	49961743	49963105	+	23.79027	0	85.96235	0	0	0	0	0	0	0	-6.91911	0.022368	1	Down
circSOS1(Ex.2-8)	chr2	39262353	39294894	-	0	0	0	0	0	0	79.75754	0	27.07972	0	6.86084	0.023527	1	Up
circSTXBP5(Ex.20-23)	chr6	1.48E+08	1.48E+08	+	23.79027	0	57.30823	0	0	0	0	0	0	0	-6.47513	0.032621	1	Down
circSTX5(Ex.3-5)	chr11	62594627	62595103	-	0	0	0	0	0	35.80251	0	28.79023	0	33.05457	6.717306	0.024321	1	Up
circDHX36(Ex.14-16)	chr3	1.54E+08	1.54E+08	-	0	0	0	0	0	35.80251	0	0	0	66.10915	6.768892	0.025466	1	Up
circARHGAP19(Ex.7-11)	chr10	98988904	99006094	-	0	0	0	0	0	71.60503	39.87877	0	0	0	6.908163	0.02258	1	Up
circARMH3(Ex.12-20)	chr10	1.04E+08	1.04E+08	-	475.8053	58.33455	114.6165	314.5149	374.6839	35.80251	79.75754	86.3707	162.4783	66.10915	-1.63285	0.016077	0.19372	Down
circGON4L(Ex.8-12)	chr1	1.56E+08	1.56E+08	-	0	0	0	0	0	35.80251	0	0	54.15945	0	6.618938	0.028926	1	Up
circIL1R1(Ex.7-10)	chr2	1.03E+08	1.03E+08	+	0	0	0	0	0	71.60503	39.87877	0	0	0	6.908163	0.02258	1	Up
circDIP2B(Ex.5-7)	chr12	51064969	51069231	+	23.79027	29.16727	0	94.35446	62.44731	286.4201	79.75754	259.1121	297.8769	33.05457	2.194188	0.034986	0.404325	Up
circMAPKAPK5(Ex.3-7)	chr12	1.12E+08	1.12E+08	+	0	0	0	0	0	0	0	86.3707	27.07972	0	6.944877	0.021869	1	Up
circKLHDC10(Ex.5-8)	chr7	1.3E+08	1.3E+08	+	0	29.16727	57.30823	0	0	0	0	0	0	0	-6.5651	0.030266	1	Down
circKIFAP3(Ex.3-10)	chr1	1.7E+08	1.7E+08	-	0	0	0	0	0	0	0	57.58047	27.07972	0	6.536456	0.030997	1	Up
circINO80C(Ex.2-4)	chr18	33058246	33060527	-	0	0	0	0	0	35.80251	79.75754	0	0	0	6.963409	0.021517	1	Up
circAAK1(Ex.15-16)	chr2	69732701	69734710	-	0	0	0	0	0	0	39.87877	57.58047	0	0	6.729009	0.02635	1	Up
circCREB1(Ex.3-7)	chr2	2.08E+08	2.08E+08	+	71.3708	0	0	0	31.22366	0	0	0	0	0	-6.78969	0.025018	1	Down

circACOT9(Ex.4-8)	chrX	23739998	23751334	-	0	58.33455	57.30823	0	0	0	0	0	0	0	-6.98148	0.02118	1	Down
circPAPOLG(Ex.6-12)	chr2	60997593	61009905	+	0	0	0	31.45149	62.44731	0	0	0	0	0	-6.66637	0.027792	1	Down
circKIF11(Ex.5-12)	chr10	94368777	94390121	+	0	58.33455	0	31.45149	0	0	0	0	0	0	-6.60318	0.029315	1	Down
circMCTP1(Ex.7-9)	chr5	94248511	94259726	-	71.3708	0	28.65412	0	0	0	0	0	0	0	-6.76605	0.02553	1	Down
circAPPL1(Ex.11-13)	chr3	57283388	57287766	+	0	0	0	0	0	0	0	57.58047	54.15945	0	6.939403	0.021974	1	Up
circTK2(Ex.4-9)	chr16	66547634	66570920	-	0	0	0	0	0	0	0	57.58047	54.15945	0	6.939403	0.021974	1	Up
circABCC4(Ex.5-11)	chr13	95838955	95863035	-	0	0	0	0	0	0	0	28.79023	0	66.10915	6.672999	0.027635	1	Up
circSPTA1(Ex.9-16)	chr1	1.59E+08	1.59E+08	-	0	0	0	0	0	35.80251	0	0	0	66.10915	6.768892	0.025466	1	Up
circCLEC16A(Ex.4-7)	chr16	11063018	11066918	+	71.3708	0	28.65412	0	0	0	0	0	0	0	-6.76605	0.02553	1	Down
circQRICH2(Ex.2-7)	chr17	74283273	74301022	-	0	0	0	0	0	35.80251	79.75754	0	0	0	6.963409	0.021517	1	Up
circTOX4(Ex.4-5)	chr14	21956749	21957562	+	0	58.33455	0	31.45149	0	0	0	0	0	0	-6.60318	0.029315	1	Down
circAGO3(Ex.6-12)	chr1	36469942	36492899	+	0	0	0	0	0	0	39.87877	0	0	66.10915	6.829618	0.024171	1	Up
circIQGAP2(Ex.33-35)	chr5	75993812	75998415	+	0	0	0	0	31.22366	143.2101	159.5151	143.9512	135.3986	66.10915	4.380766	0.011331	0.171436	Up
circKANK1(Ex.7-9)	chr9	734748	740934	+	0	0	0	0	0	0	0	28.79023	54.15945	0	6.512125	0.031632	1	Up
circCTPS2(Ex.5-6)	chrX	16711264	16711610	-	23.79027	0	57.30823	31.45149	0	0	0	0	0	0	-6.94046	0.020956	1	Down
circMAP4K5(Ex.19-28)	chr14	50901112	50910754	-	0	0	0	0	0	0	79.75754	0	27.07972	0	6.86084	0.023527	1	Up
circHIRA(Ex.2-8)	chr22	19381865	19398301	-	0	29.16727	0	0	0	71.60503	39.87877	86.3707	162.4783	198.3274	4.261542	0.02142	0.254699	Up
circSASS6(Ex.4-5)	chr1	1.01E+08	1.01E+08	-	0	0	0	0	0	0	39.87877	57.58047	0	0	6.729009	0.02635	1	Up
circLPIN1(Ex.2-3)	chr2	11905659	11907984	+	47.58053	58.33455	0	0	0	0	0	0	0	0	-6.84883	0.023775	1	Down
circNPHP3(Ex.4-6)	chr3	1.32E+08	1.32E+08	-	0	0	0	0	0	71.60503	0	28.79023	0	0	6.760284	0.025655	1	Up
circSPECC1(Ex.4-7)	chr17	20107646	20135718	+	0	0	57.30823	0	31.22366	0	0	0	0	0	-6.59789	0.029445	1	Down
circANXA6(Ex.4-8)	chr5	1.51E+08	1.51E+08	-	0	0	0	0	0	0	39.87877	57.58047	0	0	6.729009	0.02635	1	Up
circDBT(Ex.2-10)	chr1	1.01E+08	1.01E+08	-	0	0	0	0	0	71.60503	0	0	0	33.05457	6.810385	0.024575	1	Up
circNCSTN(Ex.3-5)	chr1	1.6E+08	1.6E+08	+	0	0	0	0	0	0	0	0	81.23917	33.05457	6.965952	0.021469	1	Up
circVPS13A(Ex.10-17)	chr9	79829245	79843180	+	0	0	0	0	0	0	39.87877	0	27.07972	33.05457	6.759681	0.023694	1	Up
circMAPK14(Ex.4-10)	chr6	36040650	36070426	+	0	0	28.65412	31.45149	31.22366	0	0	0	0	0	-6.6406	0.025575	1	Down
circLDHB(Ex.3-7)	chr12	21790005	21799950	-	47.58053	0	28.65412	0	0	0	0	0	0	0	-6.39174	0.034942	1	Down
circCTBP2(Ex.3-5)	chr10	1.27E+08	1.27E+08	-	118.9513	87.50182	28.65412	94.35446	156.1183	322.2226	358.9089	115.1609	162.4783	264.4366	1.321681	0.032111	0.374451	Up
circFBXW7(Ex.4-7)	chr4	1.53E+08	1.53E+08	-	0	0	0	0	0	0	0	57.58047	0	33.05457	6.617791	0.028954	1	Up
circCLTC(Ex.3-6)	chr17	57724759	57733388	+	737.4982	204.1709	888.2776	440.3208	936.7097	572.8402	79.75754	345.4828	108.3189	231.382	-1.27667	0.033948	0.393839	Down
circU2SURP(Ex.17-24)	chr3	1.43E+08	1.43E+08	+	23.79027	0	57.30823	0	31.22366	0	0	0	0	0	-6.94039	0.020957	1	Down
circANK1(Ex.14-16)	chr8	41571674	41573367	-	0	0	0	0	0	0	39.87877	0	27.07972	33.05457	6.759681	0.023694	1	Up
circATP8A1(Ex.2-5)	chr4	42618050	42629126	-	0	0	0	0	0	0	0	28.79023	81.23917	0	6.92105	0.022328	1	Up
circMIGA1(Ex.3-5)	chr1	78267016	78272786	+	0	0	0	0	0	0	0	57.58047	0	33.05457	6.617791	0.028954	1	Up
circTRAPPC8(Ex.24-28)	chr18	29412047	29432467	-	0	0	0	0	0	0	0	0	27.07972	66.10915	6.650889	0.028157	1	Up
circSHARPIN(Ex.2-6)	chr8	1.45E+08	1.45E+08	-	0	0	0	0	0	0	79.75754	0	0	33.05457	6.926145	0.022229	1	Up
circCA1(Ex.2-7)	chr8	86241918	86253888	-	0	0	0	0	0	0	0	86.3707	27.07972	0	6.944877	0.021869	1	Up
circAKAP9(Ex.12-14)	chr7	91646331	91652323	+	0	0	0	31.45149	0	71.60503	79.75754	86.3707	297.8769	66.10915	4.285905	0.017455	0.20976	Up
circUSP33(Ex.18-20)	chr1	78178885	78181553	-	23.79027	0	57.30823	0	0	0	0	0	0	0	-6.47513	0.032621	1	Down
circXPO7(Ex.6-24)	chr8	21832181	21857185	+	23.79027	29.16727	57.30823	0	0	0	0	0	0	0	-6.91459	0.021381	1	Down
circHECTD1(Ex.27-34)	chr14	31582310	31592253	-	642.3372	350.0073	458.4659	251.6119	343.4602	322.2226	159.5151	172.7414	54.15945	264.4366	-1.08583	0.024442	0.289117	Down
circUBR5(Ex.48-50)	chr8	1.03E+08	1.03E+08	-	0	58.33455	57.30823	0	0	0	0	0	0	0	-6.98148	0.02118	1	Down
circDZIP3(Ex.20-22)	chr3	1.08E+08	1.08E+08	+	47.58053	0	0	31.45149	31.22366	0	0	0	0	0	-6.90238	0.021364	1	Down
circLRRC43(Ex.2-3)	chr12	1.23E+08	1.23E+08	+	23.79027	87.50182	0	0	0	0	0	0	0	0	-6.93173	0.022123	1	Down
circCLEC16A(Ex.21-21)	chr16	11217599	11217803	+	0	0	0	0	0	0	39.87877	28.79023	27.07972	0	6.707749	0.02458	1	Up
circTCP11L2(Ex.2-6)	chr12	1.07E+08	1.07E+08	+	23.79027	0	57.30823	0	0	0	0	0	0	0	-6.47513	0.032621	1	Down
circPAN3(Ex.2-5)	chr13	28748409	28771483	+	0	29.16727	57.30823	0	0	0	0	0	0	0	-6.5651	0.030266	1	Down
circCDK11B(Ex.12-14)	chr1	1572770	1573952	-	0	0	0	0	0	0	0	0	81.23917	33.05457	6.965952	0.021469	1	Up
circWDR31(Ex.3-4)	chr9	1.16E+08	1.16E+08	-	0	0	0	0	0	35.80251	0	0	54.15945	0	6.618938	0.028926	1	Up
circBRWD1(Ex.11-13)	chr21	40646300	40649277	-	0	0	0	0	0	0	39.87877	0	54.15945	0	6.686168	0.027328	1	Up
circCSNK1A1(Ex.6-9)	chr5	1.49E+08	1.49E+08	-	0	0	0	0	0	0	0	57.58047	54.15945	0	6.939403	0.021974	1	Up
circARMH3(Ex.16-22)	chr10	1.04E+08	1.04E+08	-	0	0	0	0	0	0	0	57.58047	0	33.05457	6.617791	0.028954	1	Up
circEPB41(Ex.12-16)	chr1	29379616	29391670	+	0	29.16727	0	31.45149	31.22366	0	0	0	0	0	-6.63513	0.02565	1	Down
circLONP2(Ex.8-12)	chr16	48311249	48368269	+	23.79027	29.16727	57.30823	0	0	0	0	0	0	0	-6.91459	0.021381	1	Down
circTNRC6B(Ex.10-12)	chr22	40675999	40681774	+	0	29.16727	0	0	62.44731	0	0	0	0	0	-6.63503	0.028538	1	Down
circR3HCC1L(Ex.2-3)	chr10	99915850	99922732	+	23.79027	0	57.30823	0	0	0	0	0	0	0	-6.47513	0.032621	1	Down

circRBX1(Ex.2-3)	chr22	41349559	41360121	+	0	0	0	0	0	0	39.87877	0	0	66.10915	6.829618	0.024171	1	Up
circSAAL1(Ex.7-10)	chr11	18105082	18111057	-	71.3708	0	0	31.45149	0	0	0	0	0	0	-6.78977	0.025016	1	Down
circNEDD4L(Ex.16-19)	chr18	56010138	56024484	+	0	0	57.30823	0	31.22366	0	0	0	0	0	-6.59789	0.029445	1	Down
circKIF3A(Ex.3-5)	chr5	1.32E+08	1.32E+08	-	23.79027	0	85.96235	0	0	0	0	0	0	0	-6.91911	0.022368	1	Down
circFCHSD2(Ex.11-12)	chr11	72598515	72600990	-	23.79027	0	28.65412	31.45149	0	0	0	0	0	0	-6.52158	0.027577	1	Down
circTAX1BP1(Ex.10-11)	chr7	27832685	27834065	+	0	0	0	0	0	35.80251	39.87877	0	0	33.05457	6.869428	0.021838	1	Up
circFBXL20(Ex.13-14)	chr17	37420428	37421706	-	95.16106	29.16727	0	31.45149	62.44731	143.2101	199.3938	143.9512	108.3189	297.4912	2.032946	0.041046	0.468376	Up
circEPRS1(Ex.10-12)	chr1	2.2E+08	2.2E+08	-	190.3221	233.3382	343.8494	220.1604	593.2494	71.60503	79.75754	28.79023	162.4783	99.16372	-1.84094	0.001523	0.088368	Down
circARHGEF12(Ex.12-17)	chr11	1.2E+08	1.2E+08	+	47.58053	0	28.65412	31.45149	0	0	0	0	0	0	-6.88053	0.021736	1	Down
circATF2(Ex.5-12)	chr2	1.76E+08	1.76E+08	-	0	0	28.65412	62.90297	0	0	0	0	0	0	-6.65639	0.028028	1	Down
circSEC23IP(Ex.5-10)	chr10	1.22E+08	1.22E+08	+	23.79027	0	0	0	62.44731	0	0	0	0	0	-6.54945	0.030665	1	Down
circUSP34(Ex.22-27)	chr2	61538675	61546462	-	0	29.16727	0	62.90297	0	0	0	0	0	0	-6.65098	0.028157	1	Down
circSMARCC1(Ex.9-18)	chr3	47716965	47752298	-	0	0	0	0	0	0	39.87877	0	54.15945	0	6.686168	0.027328	1	Up
circIFRD1(Ex.5-9)	chr7	1.12E+08	1.12E+08	+	0	0	0	0	0	35.80251	0	0	27.07972	33.05457	6.69587	0.024712	1	Up
circCHD9(Ex.17-19)	chr16	53283783	53289691	+	0	29.16727	57.30823	0	0	0	0	0	0	0	-6.5651	0.030266	1	Down
circUBR3(Ex.2-11)	chr2	1.71E+08	1.71E+08	+	47.58053	0	0	0	31.22366	0	0	0	0	0	-6.42232	0.034075	1	Down
circPARP2(Ex.5-8)	chr14	20818685	20822406	+	142.7416	29.16727	114.6165	94.35446	281.0129	465.4327	319.0301	403.0633	135.3986	958.5826	1.769917	0.004517	0.156769	Up
circVPS4A(Ex.2-3)	chr16	69349911	69350275	+	23.79027	29.16727	0	0	31.22366	0	0	0	0	0	-6.51554	0.027659	1	Down
circUSP33(Ex.17-18)	chr1	78181427	78183732	-	23.79027	0	57.30823	0	0	0	0	0	0	0	-6.47513	0.032621	1	Down
circPRRC2C(Ex.8-12)	chr1	1.71E+08	1.72E+08	+	0	29.16727	85.96235	0	0	0	0	0	0	0	-6.98579	0.0211	1	Down
circUBR2(Ex.17-23)	chr6	42609320	42618124	+	47.58053	0	57.30823	0	0	0	0	0	0	0	-6.84445	0.023865	1	Down
circDCAF11(Ex.4-6)	chr14	24586475	24587364	+	0	0	0	0	0	0	0	0	54.15945	33.05457	6.571453	0.030103	1	Up
circCDC23(Ex.10-13)	chr5	1.38E+08	1.38E+08	-	0	0	0	0	0	35.80251	0	0	54.15945	0	6.618938	0.028926	1	Up
circGDI2(Ex.2-9)	chr10	5808457	5842668	-	47.58053	0	0	31.45149	0	0	0	0	0	0	-6.42242	0.034072	1	Down
circRASA1(Ex.2-5)	chr5	86627165	86633908	+	23.79027	87.50182	0	0	62.44731	250.6176	558.3028	86.3707	54.15945	198.3274	2.709388	0.046387	0.523382	Up
circNASP(Ex.10-12)	chr1	46080685	46082065	+	0	0	0	0	0	0	0	57.58047	54.15945	0	6.939403	0.021974	1	Up
circARID1B(Ex.5-6)	chr6	1.57E+08	1.57E+08	+	0	0	0	0	0	0	39.87877	57.58047	0	0	6.729009	0.02635	1	Up
circFGF11(Ex.2-4)	chr17	7344790	7346111	+	0	0	0	0	0	35.80251	39.87877	0	0	33.05457	6.869428	0.021838	1	Up
circDUS2(Ex.2-3)	chr16	68059318	68072052	+	23.79027	0	0	0	62.44731	0	0	0	0	0	-6.54945	0.030665	1	Down
circMRNIP(Ex.2-5)	chr5	1.79E+08	1.79E+08	-	309.2734	145.8364	85.96235	62.90297	93.67097	286.4201	358.9089	316.6926	514.5147	528.8732	1.522423	0.002301	0.121036	Up
circNEDD1(Ex.11-13)	chr12	97336355	97338573	+	547.1761	262.5055	401.1576	283.0634	281.0129	214.8151	159.5151	230.3219	54.15945	132.2183	-1.17693	0.014396	0.173928	Down
circAKAP9(Ex.28-31)	chr7	91700219	91709466	+	237.9027	145.8364	200.5788	220.1604	187.3419	35.80251	79.75754	57.58047	81.23917	0	-1.95849	0.033415	0.389152	Down
circPTPN1(Ex.2-5)	chr20	49177900	49191191	+	0	0	0	0	0	0	0	28.79023	0	66.10915	6.672999	0.027635	1	Up
circLONP2(Ex.10-11)	chr16	48333573	48337216	+	47.58053	58.33455	0	0	0	0	0	0	0	0	-6.84883	0.023775	1	Down
circPBRM1(Ex.20-24)	chr3	52610557	52623271	-	0	0	0	31.45149	62.44731	0	0	0	0	0	-6.66637	0.027792	1	Down
circSENP6(Ex.19-20)	chr6	76412361	76419346	+	71.3708	29.16727	57.30823	31.45149	156.1183	286.4201	279.1514	230.3219	81.23917	132.2183	1.540018	0.036005	0.415038	Up
circMIGA1(Ex.3-14)	chr1	78267016	78332076	+	0	29.16727	0	0	62.44731	0	0	0	0	0	-6.63503	0.028538	1	Down
circAGPS(Ex.11-14)	chr2	1.78E+08	1.78E+08	+	0	29.16727	0	0	62.44731	0	0	0	0	0	-6.63503	0.028538	1	Down
circEZH1(Ex.5-11)	chr17	40865227	40876442	-	0	0	57.30823	0	31.22366	0	0	0	0	0	-6.59789	0.029445	1	Down
circIL6ST(Ex.5-7)	chr5	55259180	55264224	-	23.79027	58.33455	0	0	0	0	0	0	0	0	-6.4808	0.032468	1	Down
circPCNX3(Ex.23-25)	chr11	65396066	65396886	+	47.58053	0	57.30823	0	0	0	0	0	0	0	-6.84445	0.023865	1	Down
circNDC80(Ex.8-10)	chr18	2587829	2590161	+	47.58053	0	28.65412	0	0	0	0	0	0	0	-6.39174	0.034942	1	Down
circHMGCS1(Ex.2-4)	chr5	43298111	43307926	-	0	0	0	0	0	0	39.87877	57.58047	0	0	6.729009	0.02635	1	Up
circATP13A3(Ex.9-12)	chr3	1.94E+08	1.94E+08	-	0	0	0	0	0	35.80251	0	57.58047	0	0	6.663796	0.027851	1	Up
circDNAJC1(Ex.7-8)	chr10	22171211	22193541	-	0	0	0	0	0	0	79.75754	0	27.07972	0	6.86084	0.023527	1	Up
circUSP4(Ex.12-15)	chr3	49329943	49336068	-	23.79027	58.33455	0	0	0	0	0	0	0	0	-6.4808	0.032468	1	Down
circSTAG2(Ex.32-33)	chrX	1.23E+08	1.23E+08	+	0	0	0	0	0	0	0	28.79023	54.15945	0	6.512125	0.031632	1	Up
circRNF213(Ex.19-22)	chr17	78301616	78308070	+	71.3708	0	0	0	31.22366	0	0	0	0	0	-6.78969	0.025018	1	Down
circCDC7(Ex.3-6)	chr1	91973411	91977480	+	0	0	0	31.45149	62.44731	0	0	0	0	0	-6.66637	0.027792	1	Down
circCDKL3(Ex.3-4)	chr5	1.34E+08	1.34E+08	-	23.79027	29.16727	0	31.45149	0	0	0	0	0	0	-6.51563	0.027658	1	Down
circMED24(Ex.7-9)	chr17	38188902	38189709	-	47.58053	0	28.65412	0	0	0	0	0	0	0	-6.39174	0.034942	1	Down
circEIF5(Ex.5-10)	chr14	1.04E+08	1.04E+08	+	0	0	28.65412	0	62.44731	0	0	0	0	0	-6.64051	0.028407	1	Down
circZNF562(Ex.3-4)	chr19	9768685	9770143	-	47.58053	29.16727	28.65412	0	0	0	0	0	0	0	-6.85356	0.022181	1	Down
circSH3PXD2A(Ex.8-14)	chr10	1.05E+08	1.05E+08	-	23.79027	116.6691	114.6165	125.8059	31.22366	286.4201	239.2726	259.1121	108.3189	198.3274	1.385427	0.042992	0.488128	Up
circATP7A(Ex.12-17)	chrX	77271251	77289319	+	0	0	0	0	0	0	0	28.79023	81.23917	0	6.92105	0.022328	1	Up
circWSB1(Ex.3-4)	chr17	25630393	25631937	+	0	0	0	0	0	0	0	28.79023	27.07972	33.05457	6.59481	0.02641	1	Up

circUBXN7(Ex.6-10)	chr3	1.96E+08	1.96E+08	-	23.79027	320.84	28.65412	125.8059	31.22366	322.2226	358.9089	460.6437	189.5581	264.4366	1.578774	0.024639	0.290692	Up
circRC3H2(Ex.10-11)	chr9	1.26E+08	1.26E+08	-	0	0	0	0	0	35.80251	39.87877	28.79023	0	0	6.82137	0.022648	1	Up
circDDX60L(Ex.23-29)	chr4	1.69E+08	1.69E+08	-	0	58.33455	0	31.45149	0	0	0	0	0	0	-6.60318	0.029315	1	Down
circCUL5(Ex.17-18)	chr11	1.08E+08	1.08E+08	+	452.015	437.5091	1031.548	314.5149	437.1312	71.60503	279.1514	115.1609	270.7972	198.3274	-1.52244	0.001065	0.06601	Down
circPNISR(Ex.2-4)	chr6	99860427	99864304	-	142.7416	29.16727	114.6165	0	62.44731	143.2101	279.1514	287.9023	243.7175	396.6549	1.945828	0.03658	0.421136	Up
circSLMAP(Ex.22-23)	chr3	57902633	57908750	+	0	0	0	0	0	0	0	0	54.15945	33.05457	6.571453	0.030103	1	Up
circASB3(Ex.6-7)	chr2	53941521	53943880	-	23.79027	58.33455	0	0	0	0	0	0	0	0	-6.4808	0.032468	1	Down
circRAPGEF6(Ex.22-24)	chr5	1.31E+08	1.31E+08	-	0	29.16727	28.65412	0	31.22366	0	0	0	0	0	-6.60869	0.026107	1	Down
circSUCO(Ex.10-15)	chr1	1.73E+08	1.73E+08	+	0	0	0	0	0	35.80251	0	57.58047	0	0	6.663796	0.027851	1	Up
circLRIG1(Ex.2-9)	chr3	66455622	66512933	-	0	0	0	0	0	35.80251	0	28.79023	0	33.05457	6.717306	0.024321	1	Up
circCCDC66(Ex.2-7)	chr3	56592874	56605330	+	0	0	0	0	0	35.80251	0	28.79023	27.07972	0	6.641543	0.02564	1	Up
circCIBAR1(Ex.4-7)	chr8	94717137	94731016	+	0	0	0	0	0	0	39.87877	0	0	66.10915	6.829618	0.024171	1	Up
circTIA1(Ex.3-10)	chr2	70443340	70457986	-	47.58053	0	57.30823	0	0	0	0	0	0	0	-6.84445	0.023865	1	Down
circNIT2(Ex.2-6)	chr3	1E+08	1E+08	+	23.79027	29.16727	0	31.45149	0	0	0	0	0	0	-6.51563	0.027658	1	Down
circMED12L(Ex.40-44)	chr3	1.51E+08	1.51E+08	+	47.58053	0	28.65412	0	0	0	0	0	0	0	-6.39174	0.034942	1	Down
circCWF19L2(Ex.4-8)	chr11	1.07E+08	1.07E+08	-	0	0	0	0	0	0	39.87877	57.58047	0	0	6.729009	0.02635	1	Up
circSPI1(Ex.3-4)	chr11	47380395	47381591	-	0	0	0	0	0	0	0	0	27.07972	66.10915	6.650889	0.028157	1	Up
circMFSD12(Ex.2-2)	chr19	3550982	3551192	-	23.79027	0	28.65412	0	0	143.2101	159.5151	143.9512	27.07972	66.10915	3.334276	0.049876	0.558574	Up
circCHAF1A(Ex.4-7)	chr19	4418017	4423871	+	0	29.16727	28.65412	31.45149	0	0	0	0	0	0	-6.60878	0.026106	1	Down
circAKAP9(Ex.30-31)	chr7	91707010	91709466	+	0	0	0	0	0	0	0	57.58047	54.15945	0	6.939403	0.021974	1	Up
circSTRN3(Ex.5-6)	chr14	31405701	31416469	-	47.58053	0	0	31.45149	0	0	0	0	0	0	-6.42242	0.034072	1	Down
circTMCO1(Ex.4-6)	chr1	1.66E+08	1.66E+08	-	380.6442	437.5091	257.887	1289.511	1030.381	322.2226	159.5151	86.3707	460.3553	264.4366	-1.39669	0.013686	0.171581	Down
circHSF5(Ex.2-3)	chr17	56544246	56557628	-	47.58053	58.33455	0	0	0	0	0	0	0	0	-6.84883	0.023775	1	Down
circCHD9(Ex.8-13)	chr16	53262895	53276928	+	71.3708	0	28.65412	0	0	0	0	0	0	0	-6.76605	0.02553	1	Down
circCDK5RAP2(Ex.2-12)	chr9	1.23E+08	1.23E+08	-	47.58053	0	0	0	31.22366	0	0	0	0	0	-6.42232	0.034075	1	Down
circDENND4A(Ex.30-32)	chr15	65956668	65957800	-	23.79027	0	28.65412	31.45149	31.22366	0	0	0	0	0	-6.97419	0.008909	1	Down
circEXT2(Ex.5-7)	chr11	44146339	44151688	+	47.58053	58.33455	0	0	0	0	0	0	0	0	-6.84883	0.023775	1	Down
circPPIL4(Ex.8-10)	chr6	1.5E+08	1.5E+08	-	0	0	0	0	0	0	0	28.79023	81.23917	0	6.92105	0.022328	1	Up
circATG2B(Ex.12-14)	chr14	96794684	96795971	-	23.79027	0	57.30823	0	0	0	0	0	0	0	-6.47513	0.032621	1	Down
circHERC3(Ex.5-14)	chr4	89573163	89589232	+	0	29.16727	0	0	62.44731	0	0	0	0	0	-6.63503	0.028538	1	Down
circSMARCC2(Ex.7-16)	chr12	56568435	56577714	-	0	0	0	0	0	35.80251	0	28.79023	0	33.05457	6.717306	0.024321	1	Up
circALG9(Ex.7-9)	chr11	1.12E+08	1.12E+08	-	47.58053	0	0	0	0	250.6176	39.87877	28.79023	379.1161	99.16372	4.071543	0.041366	0.471439	Up
circSEC31A(Ex.5-17)	chr4	83776056	83796975	-	0	29.16727	0	0	62.44731	0	0	0	0	0	-6.63503	0.028538	1	Down
circZDHHC17(Ex.2-5)	chr12	77191214	77203637	+	0	0	0	0	0	0	0	0	54.15945	33.05457	6.571453	0.030103	1	Up
circCPEB2(Ex.2-3)	chr4	15008929	15010051	+	0	0	0	0	0	35.80251	79.75754	0	0	0	6.963409	0.021517	1	Up
circNT5DC3(Ex.7-11)	chr12	1.04E+08	1.04E+08	-	0	0	0	0	0	0	0	28.79023	54.15945	33.05457	6.983745	0.020214	1	Up
circSAFB(Ex.3-8)	chr19	5641605	5649986	+	47.58053	58.33455	0	0	0	0	0	0	0	0	-6.84883	0.023775	1	Down
circOLA1(Ex.8-10)	chr2	1.75E+08	1.75E+08	-	0	0	0	0	0	71.60503	39.87877	0	0	0	6.908163	0.02258	1	Up
circMCTP2(Ex.9-12)	chr15	94899366	94913409	+	0	58.33455	57.30823	0	0	0	0	0	0	0	-6.98148	0.02118	1	Down
circTTF1(Ex.3-4)	chr9	1.35E+08	1.35E+08	-	23.79027	0	28.65412	0	31.22366	0	0	0	0	0	-6.52148	0.027578	1	Down
circPOLH(Ex.4-6)	chr6	43555009	43568828	+	0	0	0	0	0	0	79.75754	0	0	33.05457	6.926145	0.022229	1	Up
circPIK3R5(Ex.10-12)	chr17	8790413	8792208	-	0	0	0	0	0	0	39.87877	28.79023	27.07972	0	6.707749	0.02458	1	Up
circNOL10(Ex.6-13)	chr2	10784446	10811816	-	0	0	0	0	0	35.80251	0	0	54.15945	0	6.618938	0.028926	1	Up
circCLIP2(Ex.5-8)	chr7	73770740	73778645	+	0	0	0	0	0	0	39.87877	28.79023	27.07972	0	6.707749	0.02458	1	Up
circLPIN2(Ex.14-16)	chr18	2923773	2925366	-	95.16106	58.33455	0	62.90297	62.44731	107.4075	199.3938	230.3219	460.3553	165.2729	2.070209	0.020341	0.243479	Up
circNMI(Ex.6-7)	chr2	1.52E+08	1.52E+08	-	0	0	0	0	0	35.80251	39.87877	0	27.07972	0	6.801438	0.02301	1	Up
circPLXNC1(Ex.5-7)	chr12	94603366	94618091	+	71.3708	0	0	0	31.22366	0	0	0	0	0	-6.78969	0.025018	1	Down
circSNRNP40(Ex.3-8)	chr1	31740718	31764853	-	0	58.33455	0	31.45149	0	0	0	0	0	0	-6.60318	0.029315	1	Down
circUFD1(Ex.2-10)	chr22	19443203	19463125	-	0	0	0	0	0	35.80251	0	0	54.15945	0	6.618938	0.028926	1	Up
circNOP14(Ex.4-8)	chr4	2951661	2956290	-	0	58.33455	57.30823	0	0	0	0	0	0	0	-6.98148	0.02118	1	Down
circGOLIM4(Ex.9-10)	chr3	1.68E+08	1.68E+08	-	0	0	0	0	0	0	39.87877	0	0	66.10915	6.829618	0.024171	1	Up
circPCNX3(Ex.2-6)	chr11	65384295	65386538	+	0	29.16727	57.30823	0	0	0	0	0	0	0	-6.5651	0.030266	1	Down
circADAM10(Ex.6-8)	chr15	58932976	58938403	-	23.79027	0	28.65412	31.45149	0	0	0	0	0	0	-6.52158	0.027577	1	Down
circXPO1(Ex.17-23)	chr2	61709515	61717911	-	23.79027	0	57.30823	0	0	0	0	0	0	0	-6.47513	0.032621	1	Down
circLARP1B(Ex.11-19)	chr4	1.29E+08	1.29E+08	+	23.79027	0	28.65412	0	31.22366	0	0	0	0	0	-6.52148	0.027578	1	Down
circERLEC1(Ex.7-11)	chr2	54028534	54040210	+	0	0	0	0	0	0	39.87877	57.58047	0	0	6.729009	0.02635	1	Up

circTBC1D31(Ex.4-11)	chr8	1.24E+08	1.24E+08	+	0	29.16727	85.96235	0	0	0	0	0	0	0	-6.98579	0.0211	1	Down
circSORT1(Ex.7-11)	chr1	1.1E+08	1.1E+08	-	47.58053	0	0	31.45149	0	0	0	0	0	0	-6.42242	0.034072	1	Down
circFOXK2(Ex.2-8)	chr17	80521230	80545148	+	0	0	0	0	0	71.60503	0	0	27.07972	0	6.739484	0.026115	1	Up
circCD74(Ex.2-4)	chr5	1.5E+08	1.5E+08	-	0	0	0	0	0	71.60503	0	28.79023	0	0	6.760284	0.025655	1	Up
circKHDRBS1(Ex.5-8)	chr1	32502511	32505174	+	0	0	0	0	0	35.80251	39.87877	0	27.07972	0	6.801438	0.02301	1	Up
circDHX29(Ex.19-21)	chr5	54565241	54566534	-	0	0	0	0	0	0	0	28.79023	54.15945	0	6.512125	0.031632	1	Up
circIPO11(Ex.4-6)	chr5	61745754	61763090	+	0	58.33455	28.65412	0	0	0	0	0	0	0	-6.57615	0.029987	1	Down
circCNTRL(Ex.12-15)	chr9	1.24E+08	1.24E+08	+	0	0	0	0	0	0	0	28.79023	0	66.10915	6.672999	0.027635	1	Up
circMYCBP2(Ex.53-55)	chr13	77695508	77700677	-	0	0	0	0	0	0	0	57.58047	54.15945	0	6.939403	0.021974	1	Up
circAFG1L(Ex.4-8)	chr6	1.09E+08	1.09E+08	+	71.3708	0	28.65412	0	0	0	0	0	0	0	-6.76605	0.02553	1	Down
circREPS2(Ex.5-9)	chrX	17047649	17080655	+	0	0	57.30823	31.45149	0	0	0	0	0	0	-6.59798	0.029443	1	Down
circTLK1(Ex.6-12)	chr2	1.72E+08	1.72E+08	-	499.5956	145.8364	286.5412	62.90297	249.7892	0	39.87877	57.58047	27.07972	66.10915	-2.71095	0.00289	0.136913	Down
circZMYND8(Ex.14-18)	chr20	45855946	45875261	-	0	0	0	0	0	0	0	28.79023	54.15945	0	6.512125	0.031632	1	Up
circCCNE1(Ex.5-6)	chr19	30308044	30308448	+	23.79027	87.50182	0	0	0	0	0	0	0	0	-6.93173	0.022123	1	Down
circDOCK11(Ex.31-32)	chrX	1.18E+08	1.18E+08	+	23.79027	0	57.30823	0	31.22366	0	0	0	0	0	-6.94039	0.020957	1	Down
circATF7IP2(Ex.10-13)	chr16	10565967	10574821	+	118.9513	204.1709	143.2706	125.8059	187.3419	393.8276	558.3028	259.1121	297.8769	231.382	1.150897	0.008145	0.171436	Up
circNUP98(Ex.12-18)	chr11	3740642	3765879	-	0	0	0	0	0	0	39.87877	0	0	66.10915	6.829618	0.024171	1	Up
circKIF5B(Ex.12-14)	chr10	32320001	32322966	-	0	58.33455	0	0	31.22366	0	0	0	0	0	-6.60309	0.029317	1	Down
circSETD1A(Ex.13-13)	chr16	30982699	30983040	+	0	0	0	0	0	0	0	57.58047	27.07972	0	6.536456	0.030997	1	Up
circUNC13B(Ex.2-5)	chr9	35228012	35237823	+	47.58053	0	0	62.90297	0	0	0	0	0	0	-6.91556	0.022437	1	Down
circAPPL1(Ex.7-20)	chr3	57276884	57301820	+	23.79027	29.16727	0	31.45149	0	0	0	0	0	0	-6.51563	0.027658	1	Down
circCPLANE1(Ex.7-8)	chr5	37238959	37239971	-	0	29.16727	57.30823	0	0	0	0	0	0	0	-6.5651	0.030266	1	Down
circSMARCC1(Ex.6-10)	chr3	47747899	47770584	-	0	0	0	0	0	71.60503	0	0	27.07972	0	6.739484	0.026115	1	Up
circBLTP1(Ex.12-16)	chr4	1.23E+08	1.23E+08	+	47.58053	0	28.65412	0	31.22366	0	0	0	0	0	-6.88046	0.021737	1	Down
circCLHC1(Ex.8-9)	chr2	55433406	55435846	-	71.3708	0	28.65412	0	0	0	0	0	0	0	-6.76605	0.02553	1	Down
circTSPAN15(Ex.3-6)	chr10	71244897	71264241	+	356.854	670.8473	257.887	377.4178	780.5914	214.8151	239.2726	287.9023	81.23917	330.5457	-1.09429	0.014275	0.173928	Down
circGSR(Ex.5-8)	chr8	30550486	30560757	-	0	0	0	0	0	0	0	57.58047	54.15945	0	6.939403	0.021974	1	Up
circCASC3(Ex.4-8)	chr17	38318006	38323114	+	0	0	0	0	0	35.80251	0	28.79023	0	33.05457	6.717306	0.024321	1	Up
circTRIP12(Ex.31-33)	chr2	2.31E+08	2.31E+08	-	0	58.33455	0	0	31.22366	0	0	0	0	0	-6.60309	0.029317	1	Down
circANK1(Ex.29-35)	chr8	41545674	41551620	-	0	0	0	0	0	35.80251	0	0	0	66.10915	6.768892	0.025466	1	Up
