# Supplementary material for: Circular RNA FCHO2 promotes airway remodeling in COPD via regulating nuclear translocation of PTBP1 to repress the splicing of GRN pre-mRNA
Source: Cell Death Dis. 2025 Nov 3;16(1):779. doi: 10.1038/s41419-025-08107-9 (PMC12583663; doi:10.1038/s41419-025-08107-9)
Supplement: Supplementary file 4 — Original western blots [file 41419_2025_8107_MOESM4_ESM.pdf]

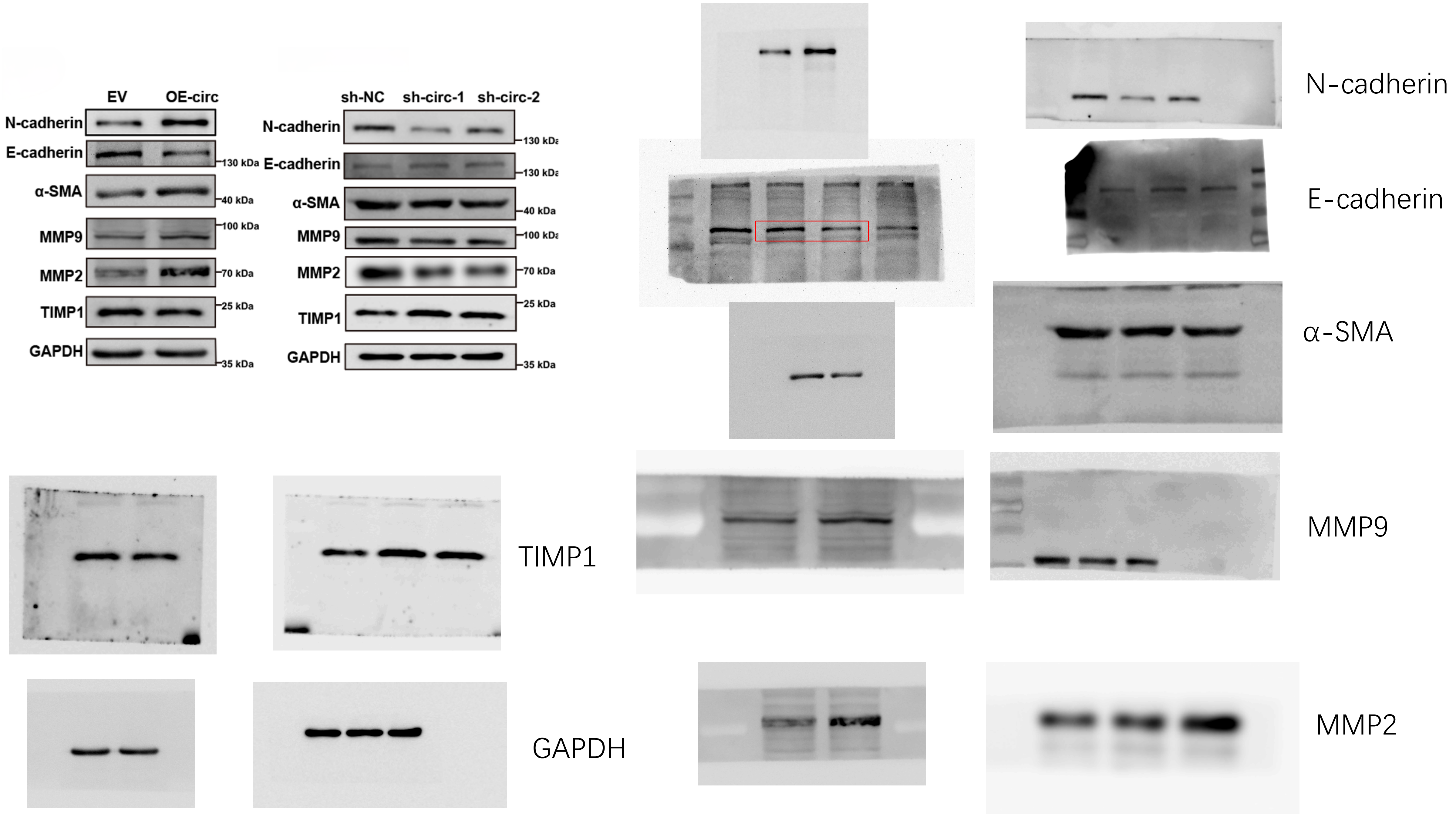
EV
OE-circ
N-cadherin
E-cadherin
α-SMA
MMP9
MMP2
TIMP1
GAPDH
130 kDa
40 kDa
100 kDa
70 kDa
25 kDa
35 kDa
sh-NC
sh-circ-1
sh-circ-2
N-cadherin
E-cadherin
α-SMA
MMP9
MMP2
TIMP1
GAPDH
130 kDa
130 kDa
40 kDa
100 kDa
70 kDa
25 kDa
35 kDa
N-cadherin
E-cadherin
α-SMA
MMP9
MMP2
TIMP1
GAPDH

Figure 2

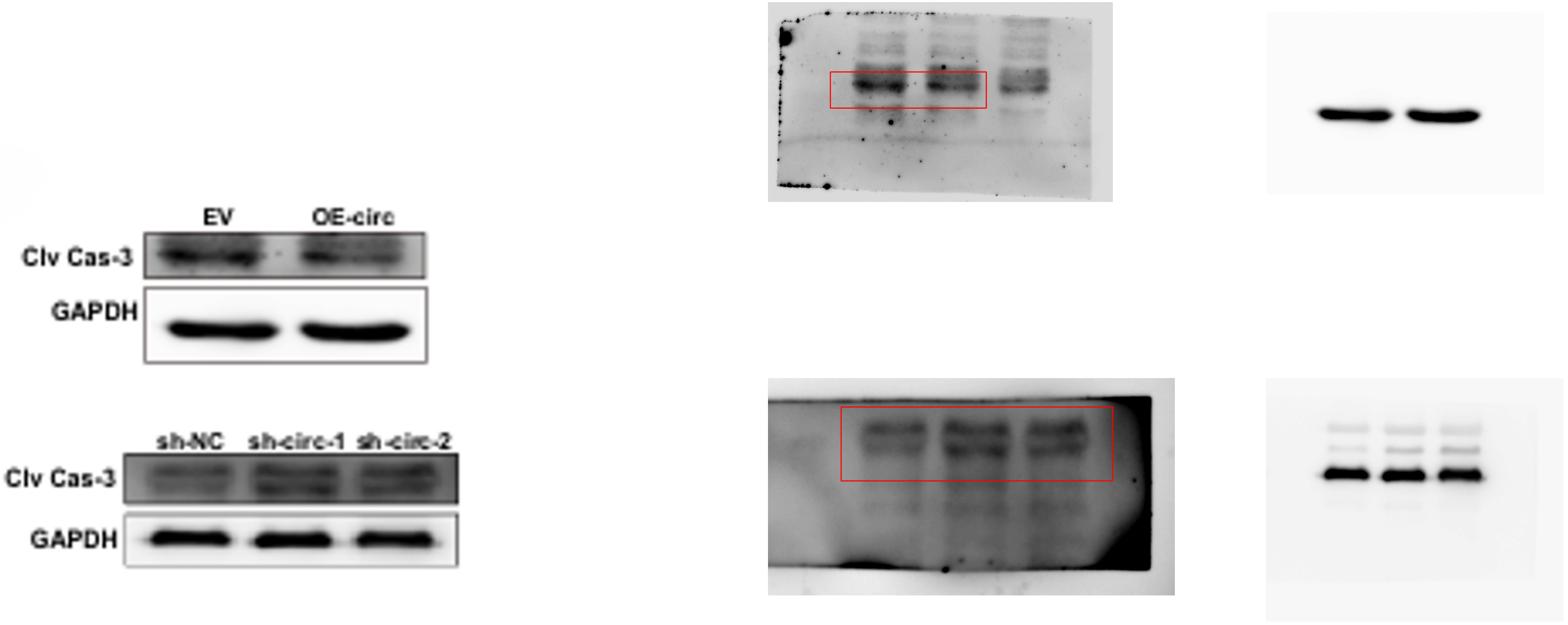
EV
OE-circ
Clv Cas-3
GAPDH
sh-NC
sh-circ-1
sh-circ-2
Clv Cas-3
GAPDH

Figure 2

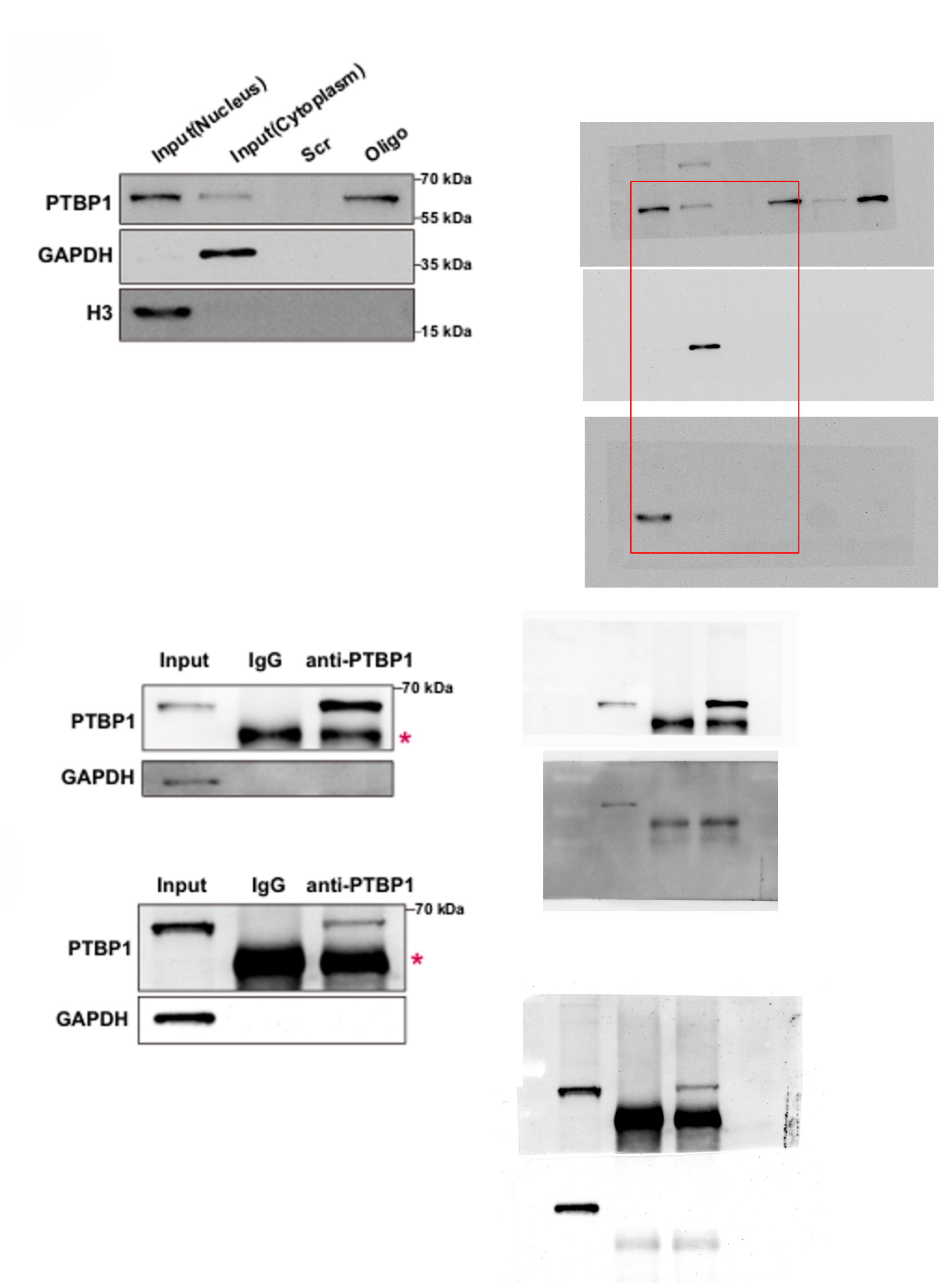
Input(Nucleus)
Input(Cytoplasm)
Scr
Oligo
PTBP1
GAPDH
H3
70 kDa
55 kDa
35 kDa
15 kDa
Input
IgG
anti-PTBP1
PTBP1
GAPDH
70 kDa
*
Input
IgG
anti-PTBP1
PTBP1
GAPDH
70 kDa
*
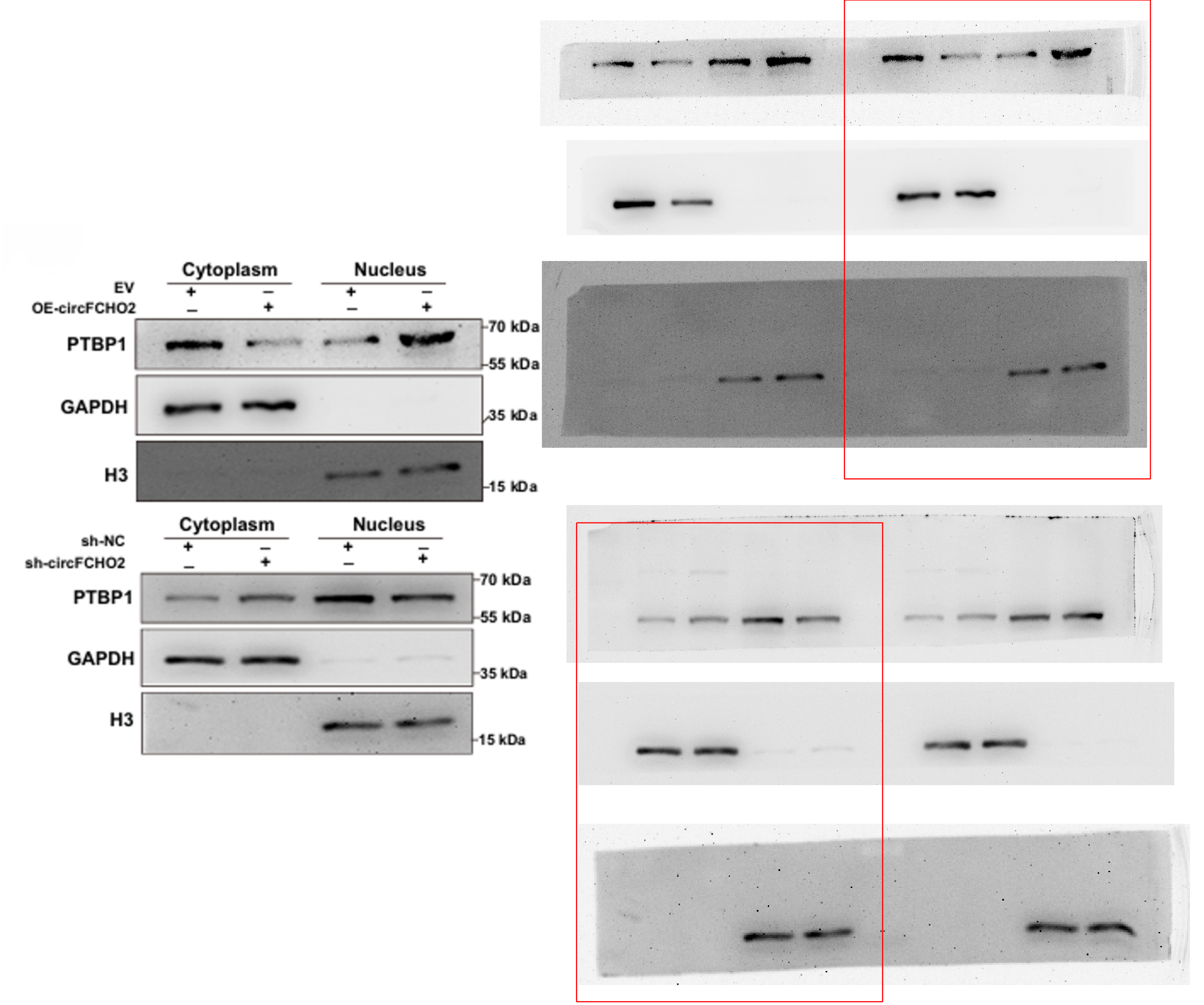
Cytoplasm
Nucleus
EV
OE-circFCHO2
PTBP1
GAPDH
H3
70 kDa
55 kDa
35 kDa
15 kDa
Cytoplasm
Nucleus
sh-NC
sh-circFCHO2
PTBP1
GAPDH
H3
70 kDa
55 kDa
35 kDa
15 kDa

Figure 3

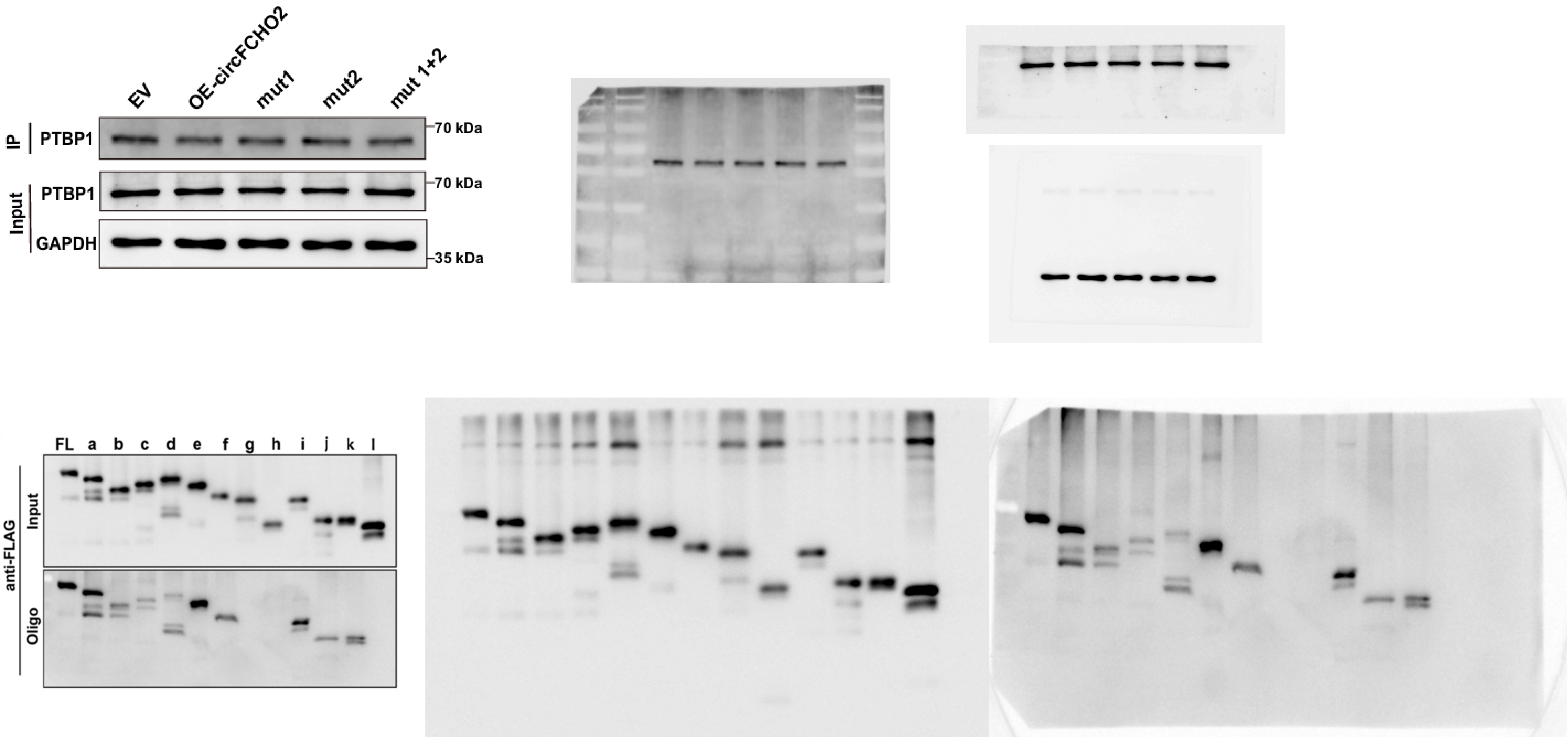
EV
OE-circFCHO2
mut1
mut2
mut 1+2
IP
PTBP1
70 kDa
Input
PTBP1
70 kDa
GAPDH
35 kDa
FL
a
b
c
d
e
f
g
h
i
j
k
l
anti-FLAG
Input
Oligo

Figure 3

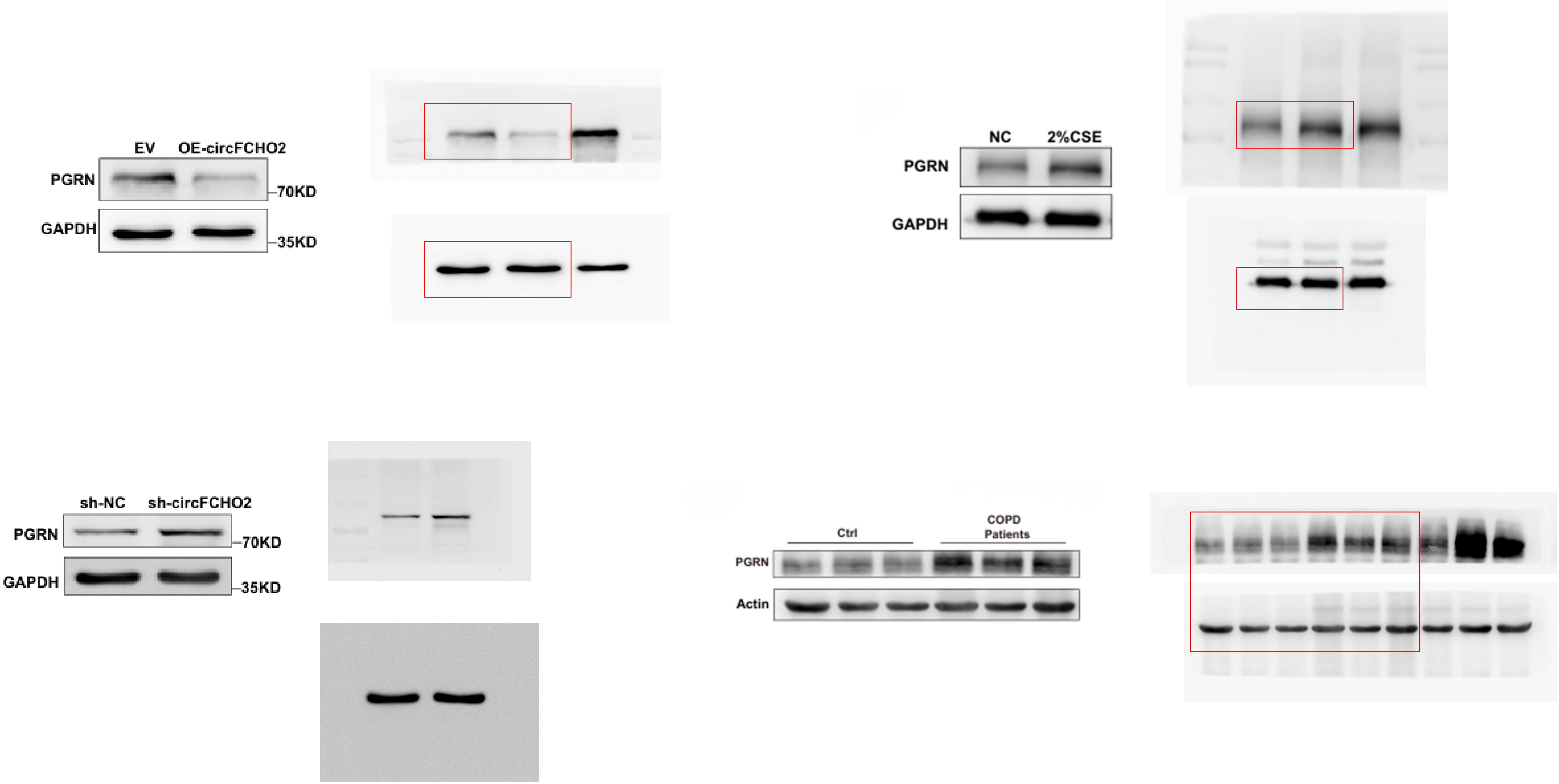
EV
OE-circFCHO2
PGRN
GAPDH
70KD
35KD
NC
2%CSE
PGRN
GAPDH
sh-NC
sh-circFCHO2
PGRN
GAPDH
70KD
35KD
Ctrl
COPD
Patients
PGRN
Actin

Figure 4

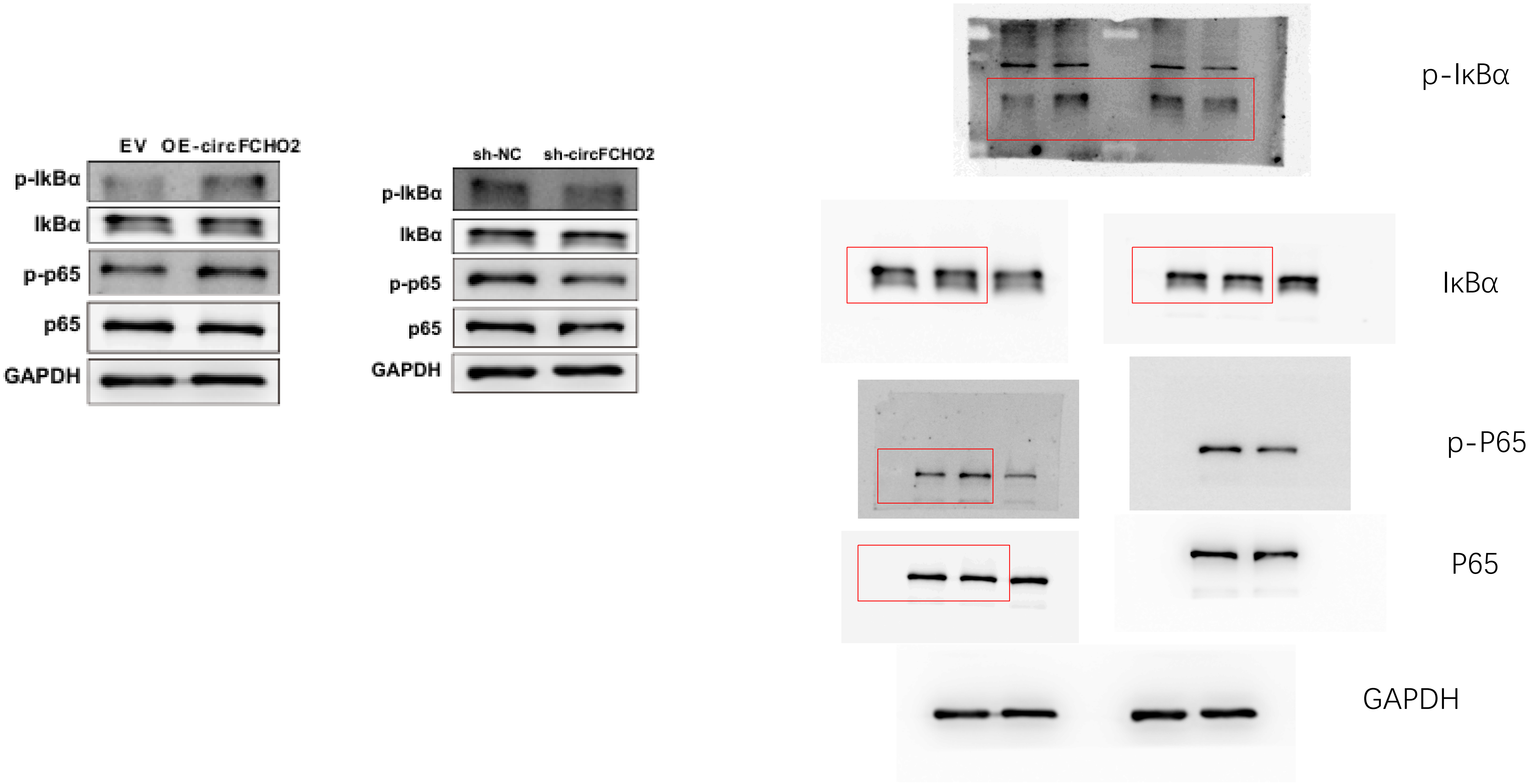
EV OE-circFCHO2
p-IkBα
IkBα
p-p65
p65
GAPDH
sh-NC sh-circFCHO2
p-IkBα
IkBα
p-p65
p65
GAPDH
p-IκBα
IκBα
p-P65
P65
GAPDH

Figure 5

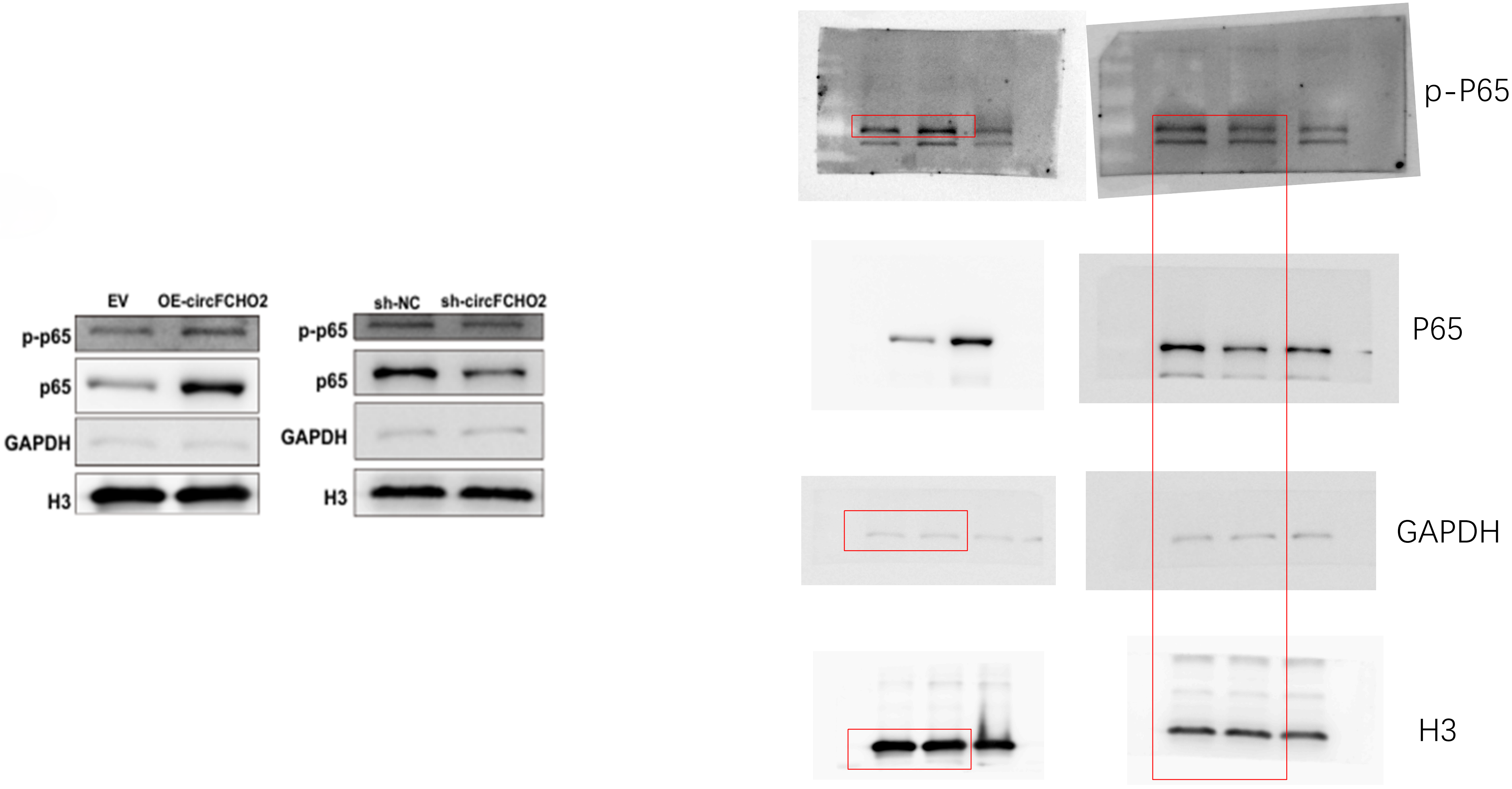
EV
OE-circFCHO2
sh-NC
sh-circFCHO2
p-p65
p65
GAPDH
H3
p-P65
P65
GAPDH
H3

Figure 5

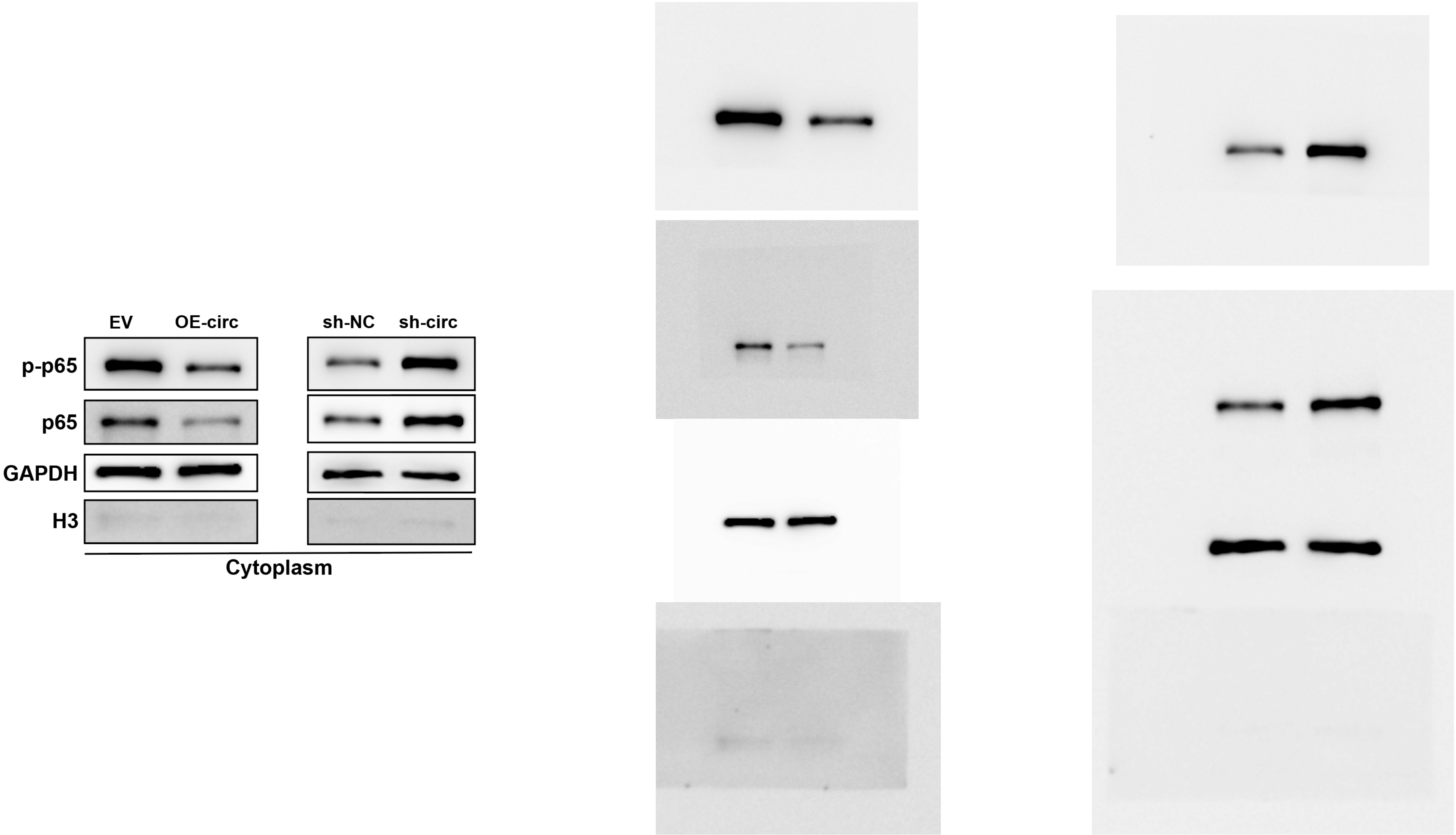

Figure 5

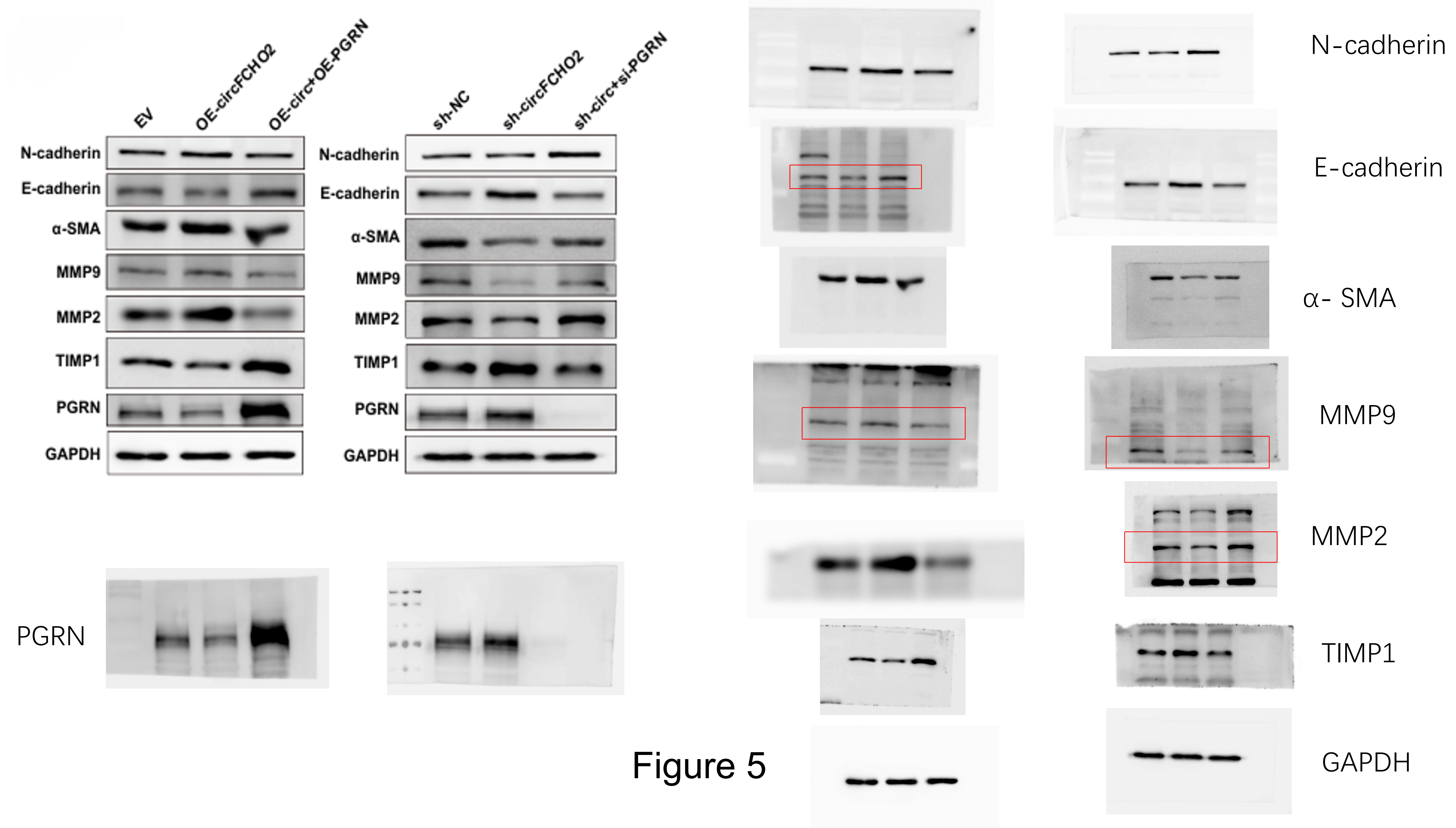
EV
OE-circFCHO2
OE-circ+OE-PGRN
sh-NC
sh-circFCHO2
sh-circ+si-PGRN
N-cadherin
E-cadherin
α-SMA
MMP9
MMP2
TIMP1
PGRN
GAPDH
N-cadherin
E-cadherin
α-SMA
MMP9
MMP2
TIMP1
PGRN
GAPDH
PGRN
N-cadherin
E-cadherin
α- SMA
MMP9
MMP2
TIMP1
GAPDH

Figure 5

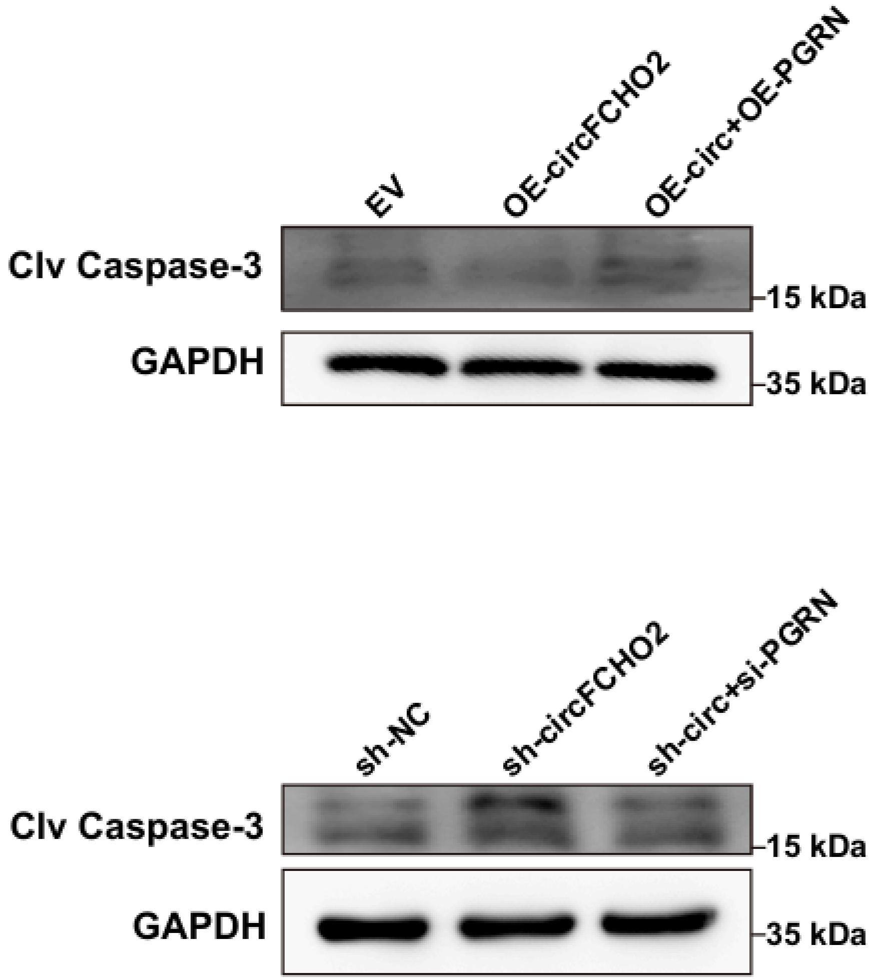
EV
OE-circFCHO2
OE-circ+OE-PGRN
Clv Caspase-3
15 kDa
GAPDH
35 kDa
sh-NC
sh-circFCHO2
sh-circ+si-PGRN
Clv Caspase-3
15 kDa
GAPDH
35 kDa

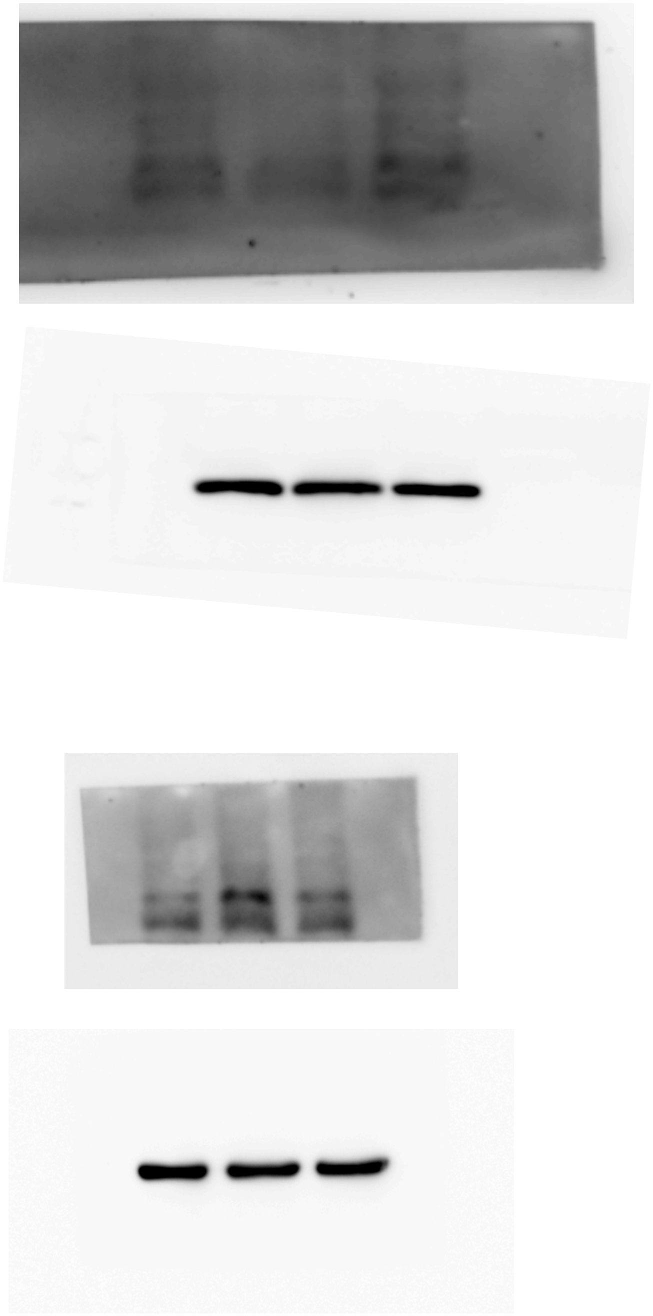

Figure 5

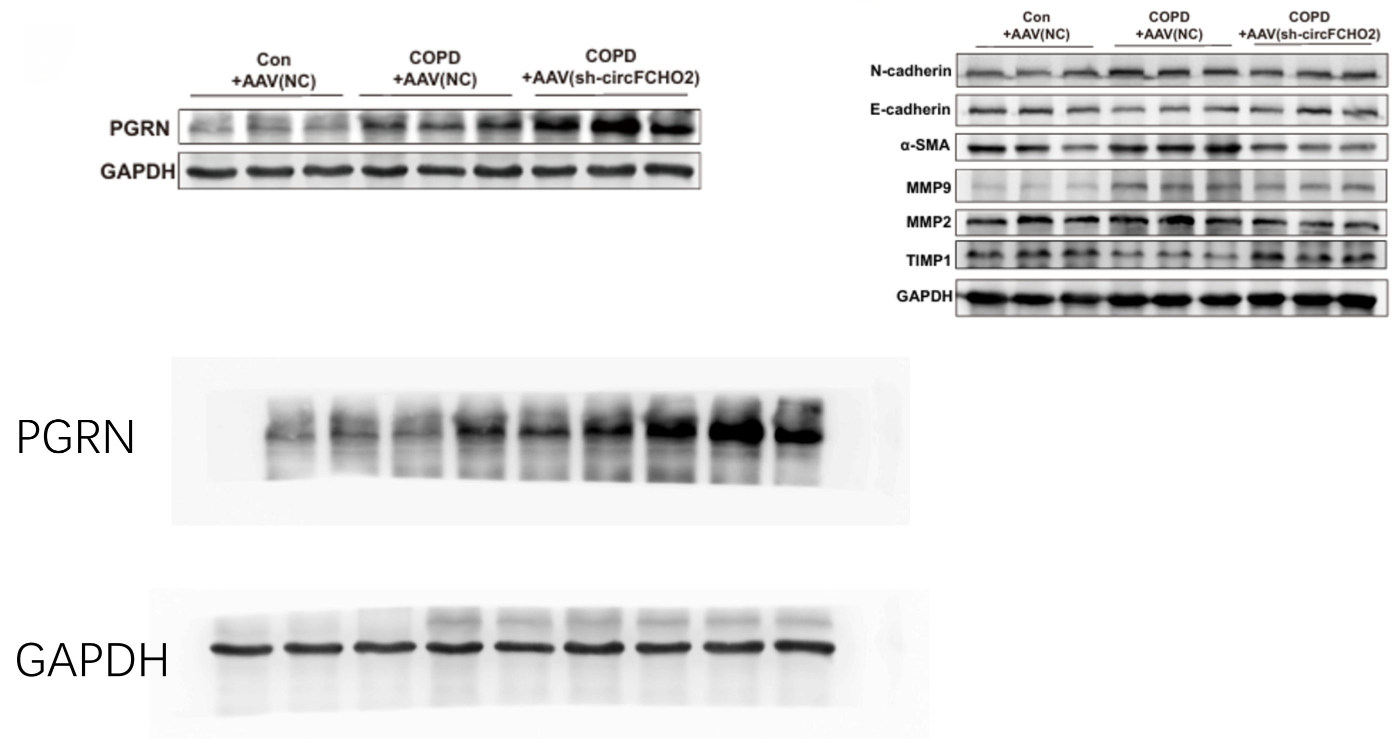
Con
+AAV(NC)
COPD
+AAV(NC)
COPD
+AAV(sh-circFCHO2)
PGRN
GAPDH
Con
+AAV(NC)
COPD
+AAV(NC)
COPD
+AAV(sh-circFCHO2)
N-cadherin
E-cadherin
α-SMA
MMP9
MMP2
TIMP1
GAPDH
PGRN
GAPDH

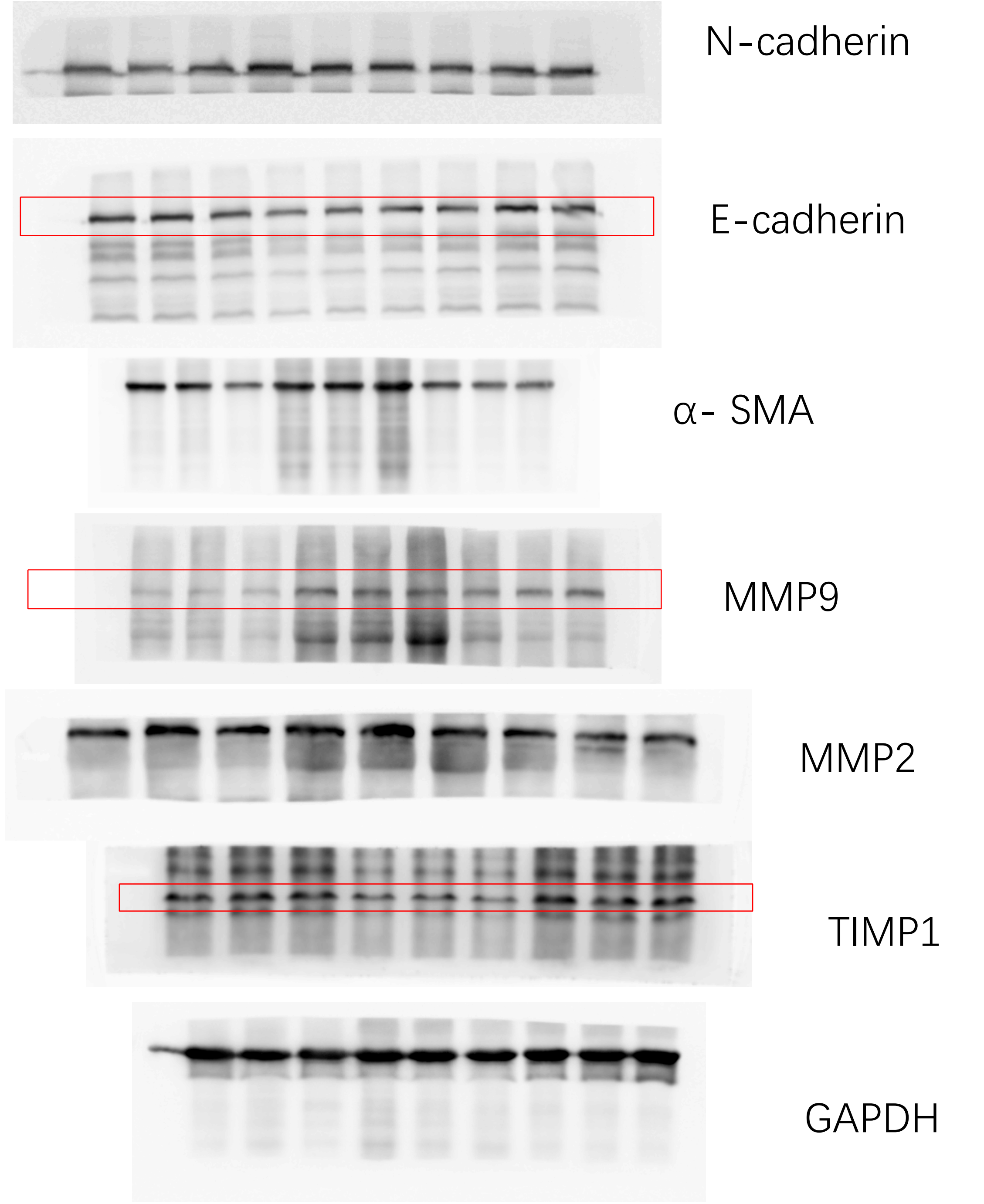
N-cadherin
E-cadherin
α- SMA
MMP9
MMP2
TIMP1
GAPDH

Figure 6

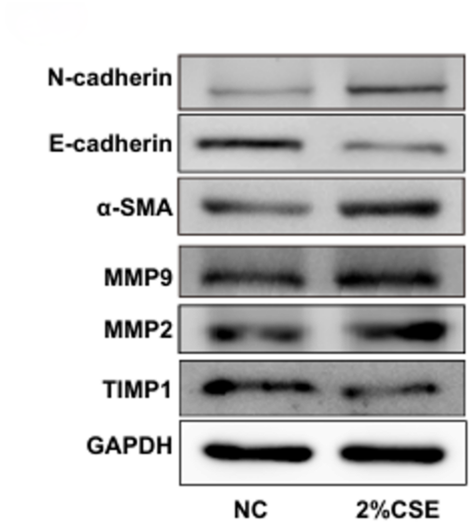

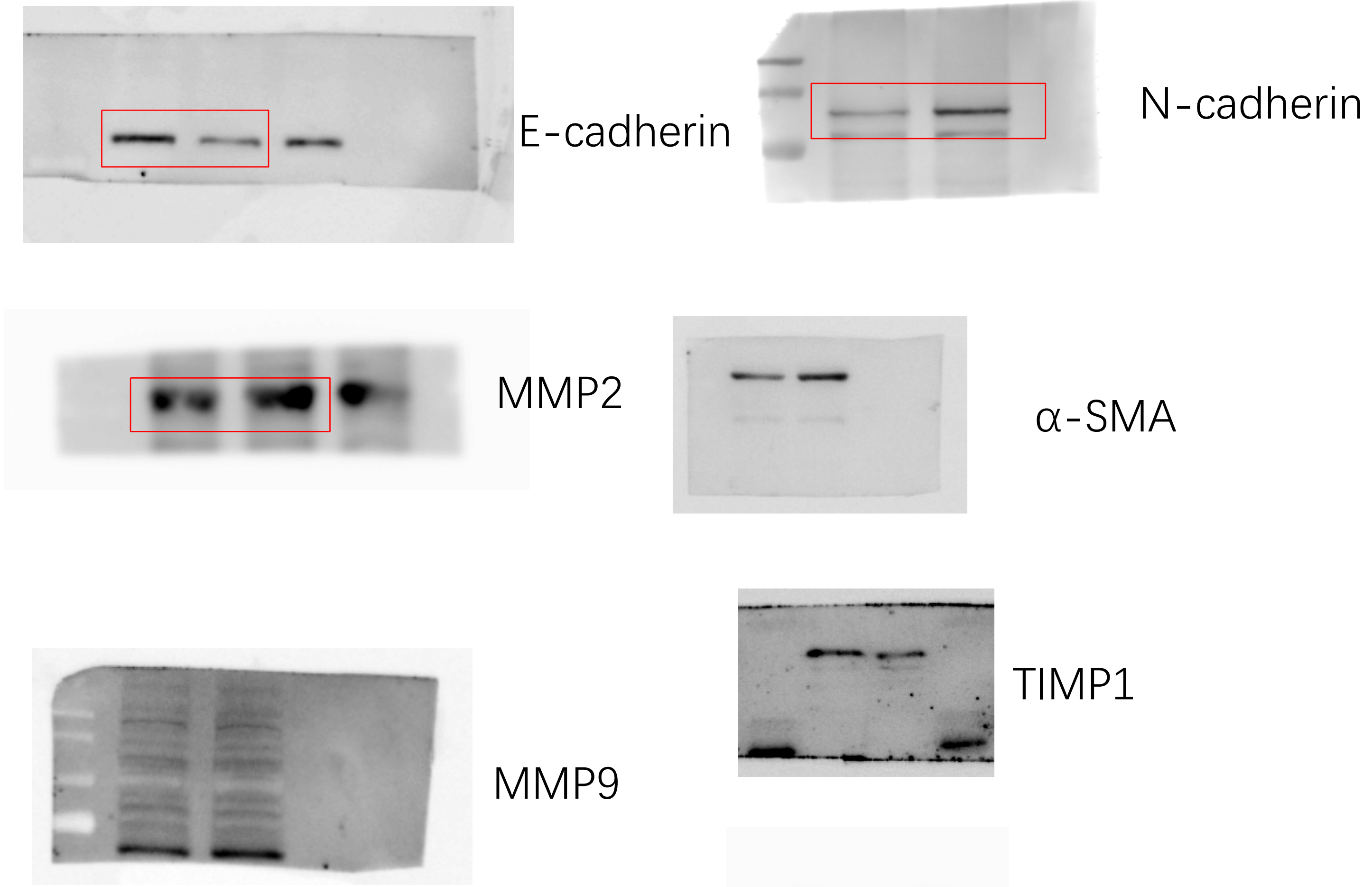

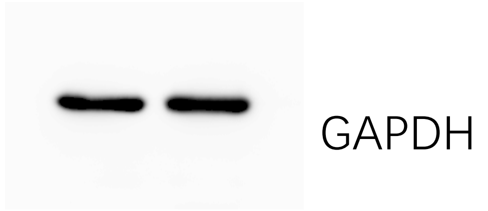

Supplementary Figure 2

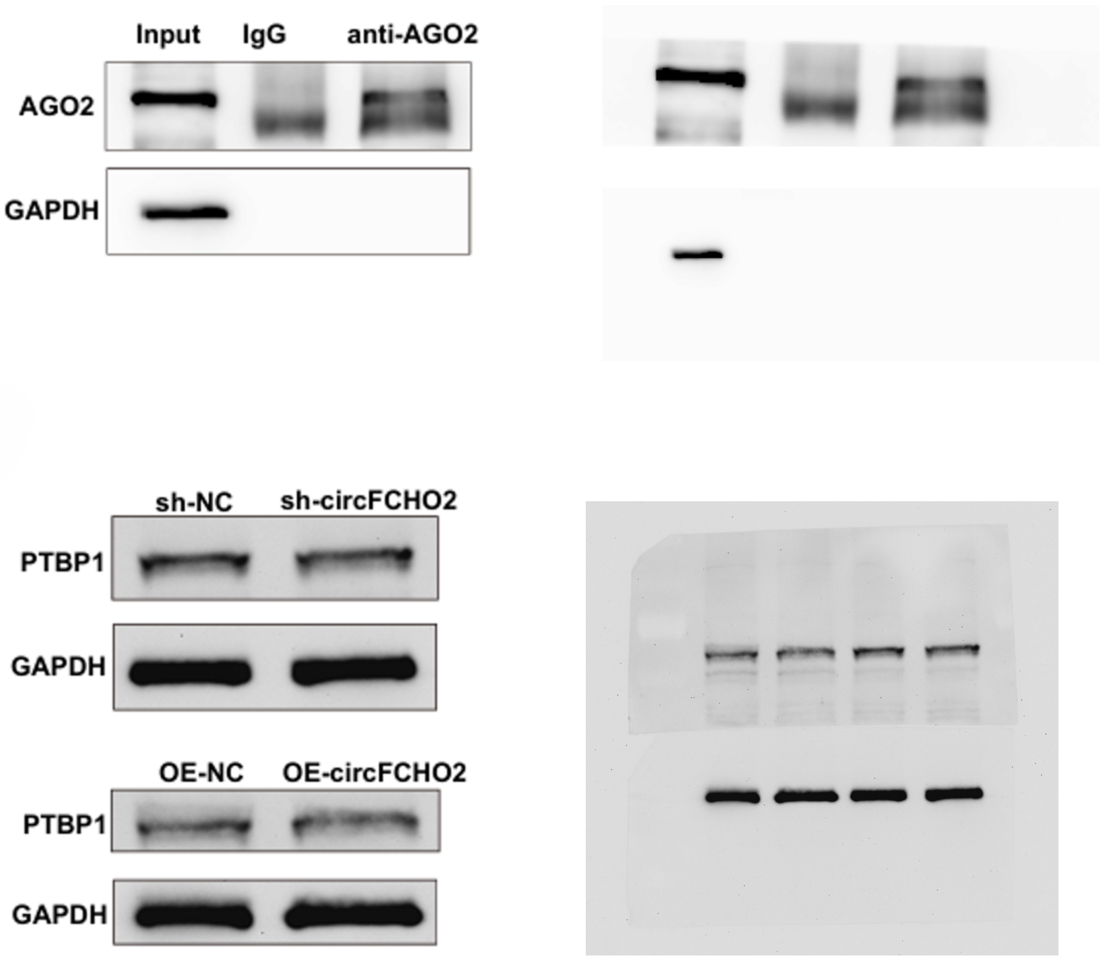

Supplementary Figure 3

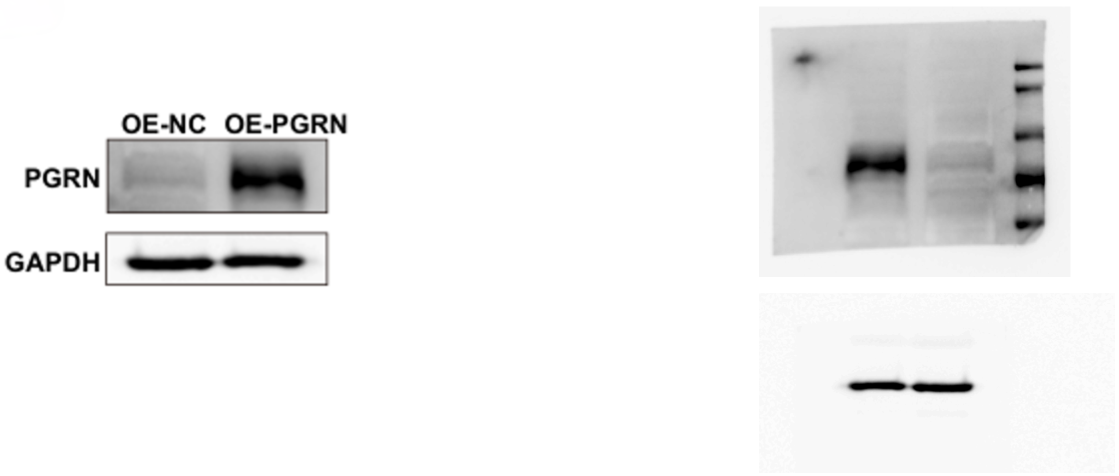

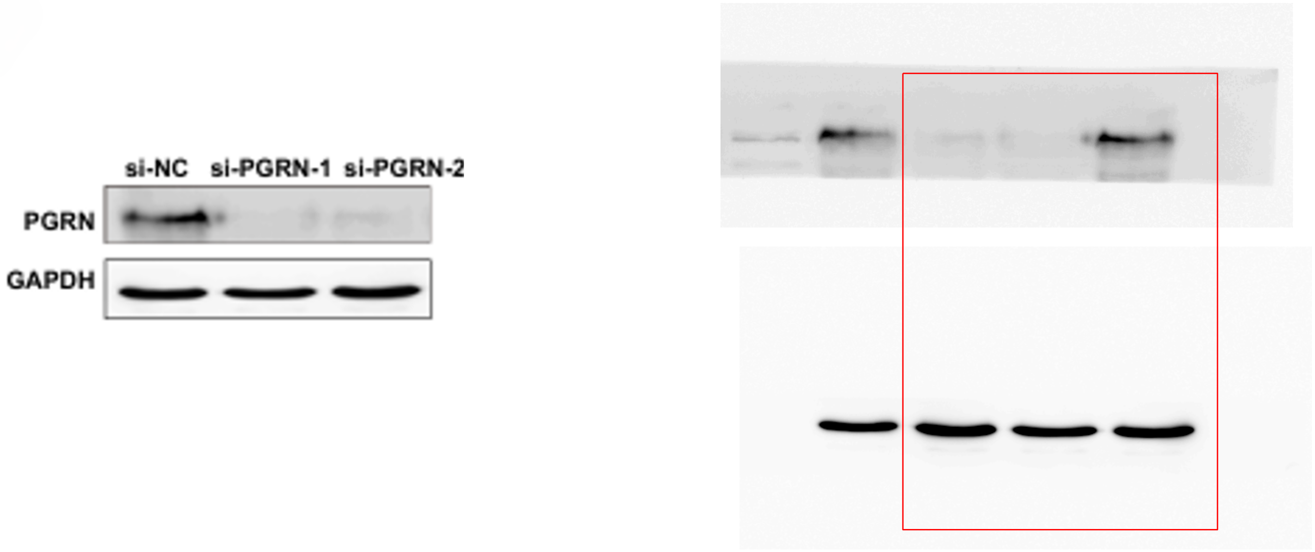

Supplementary Figure 4

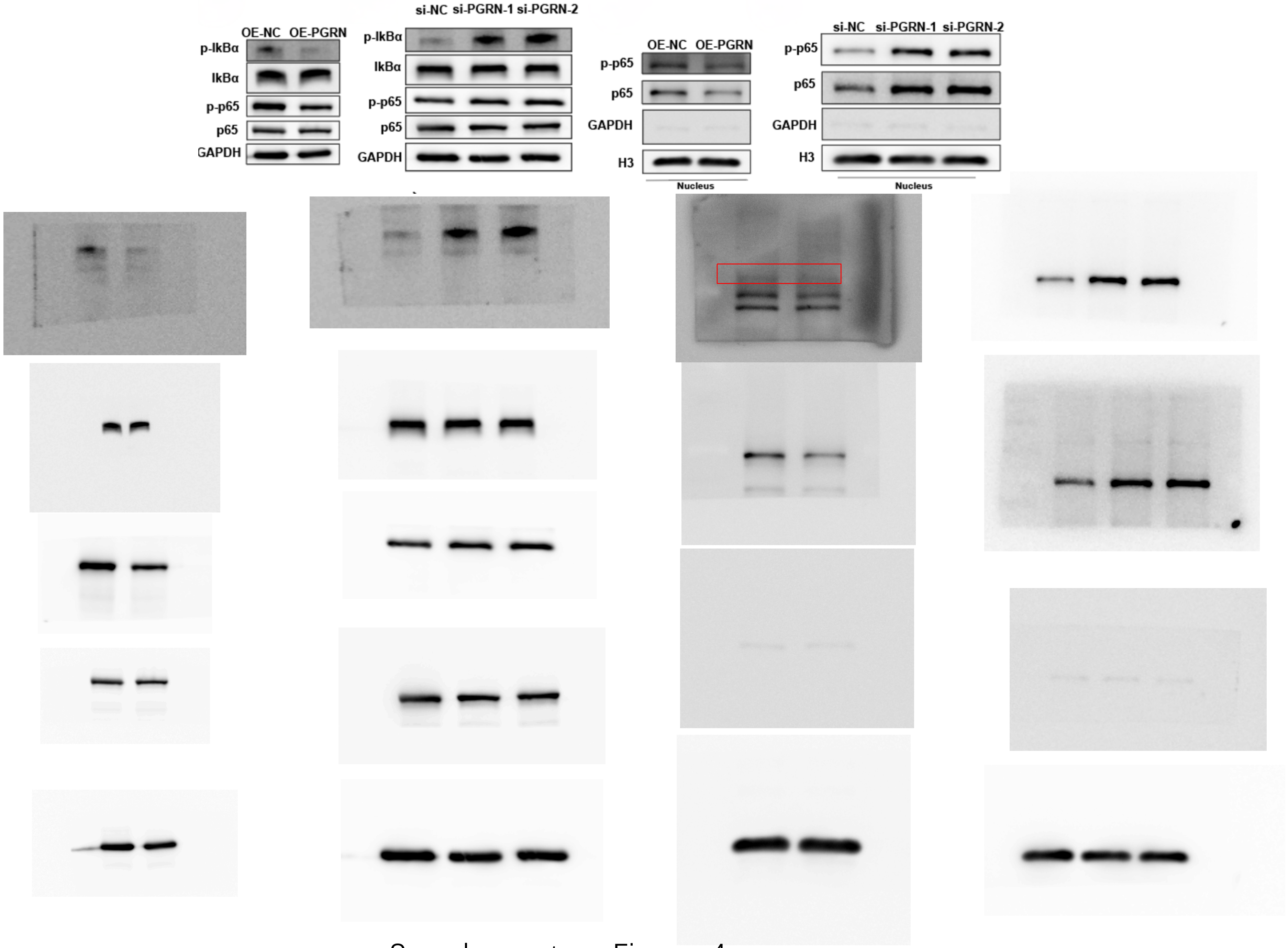

Supplementary Figure 4

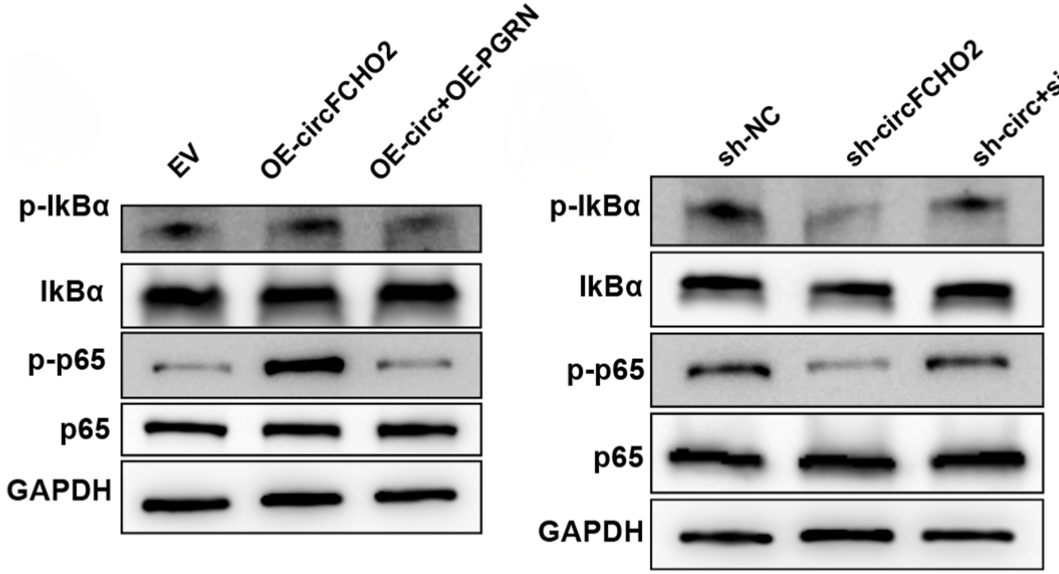

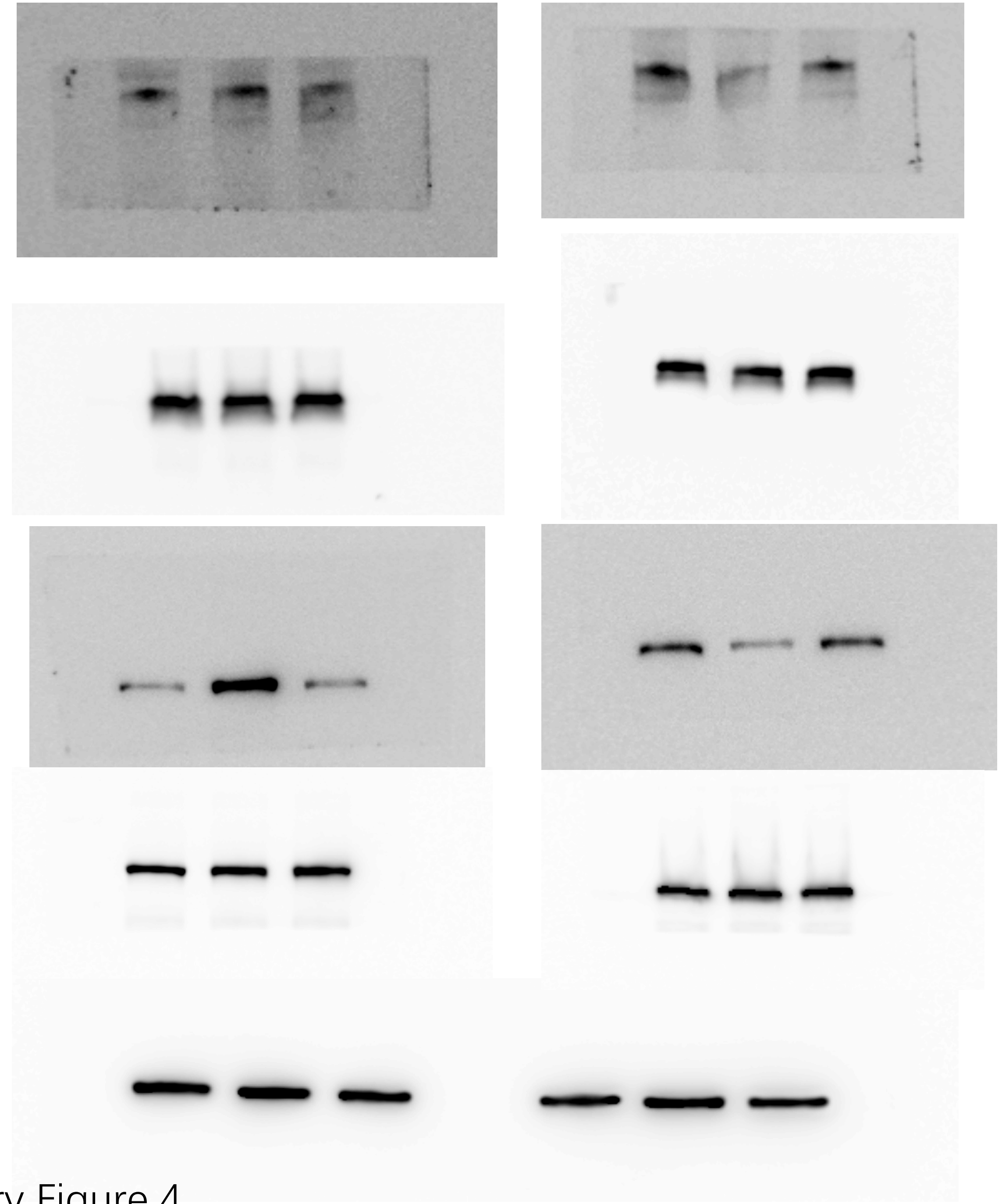

Supplementary Figure 4

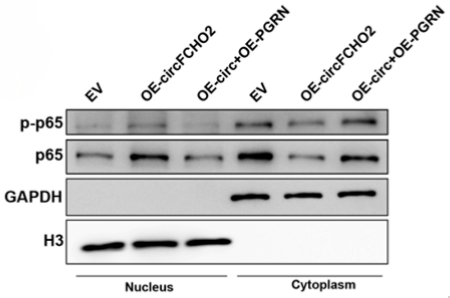

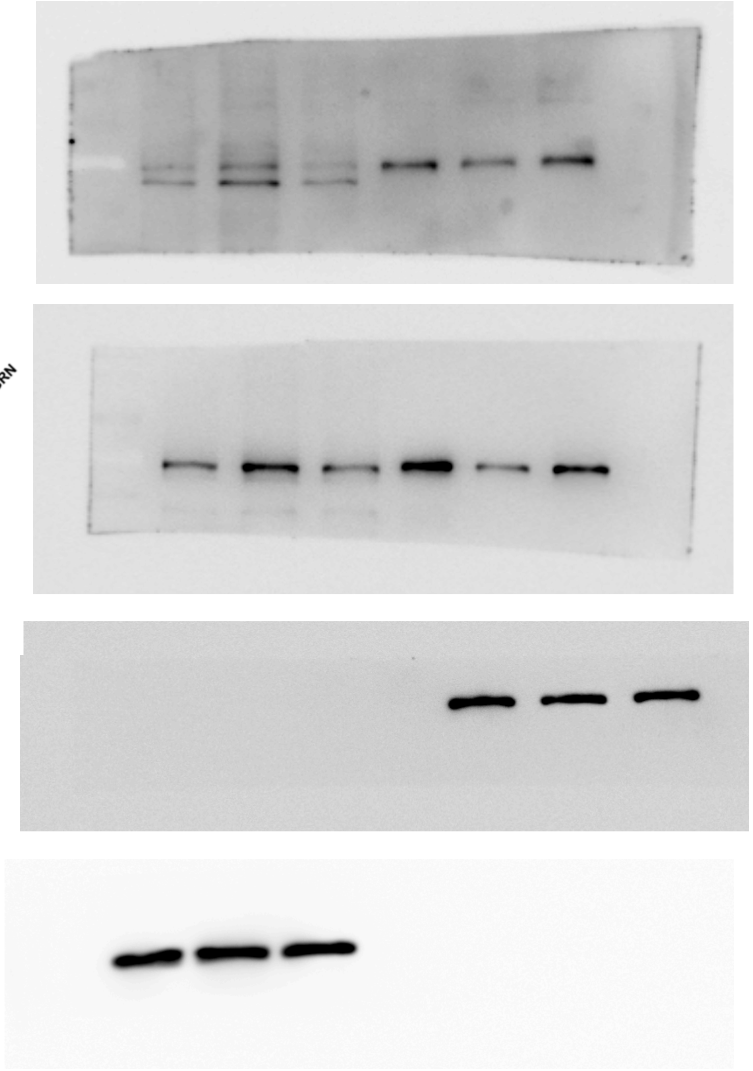

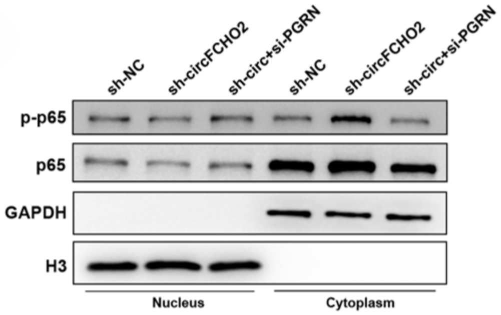

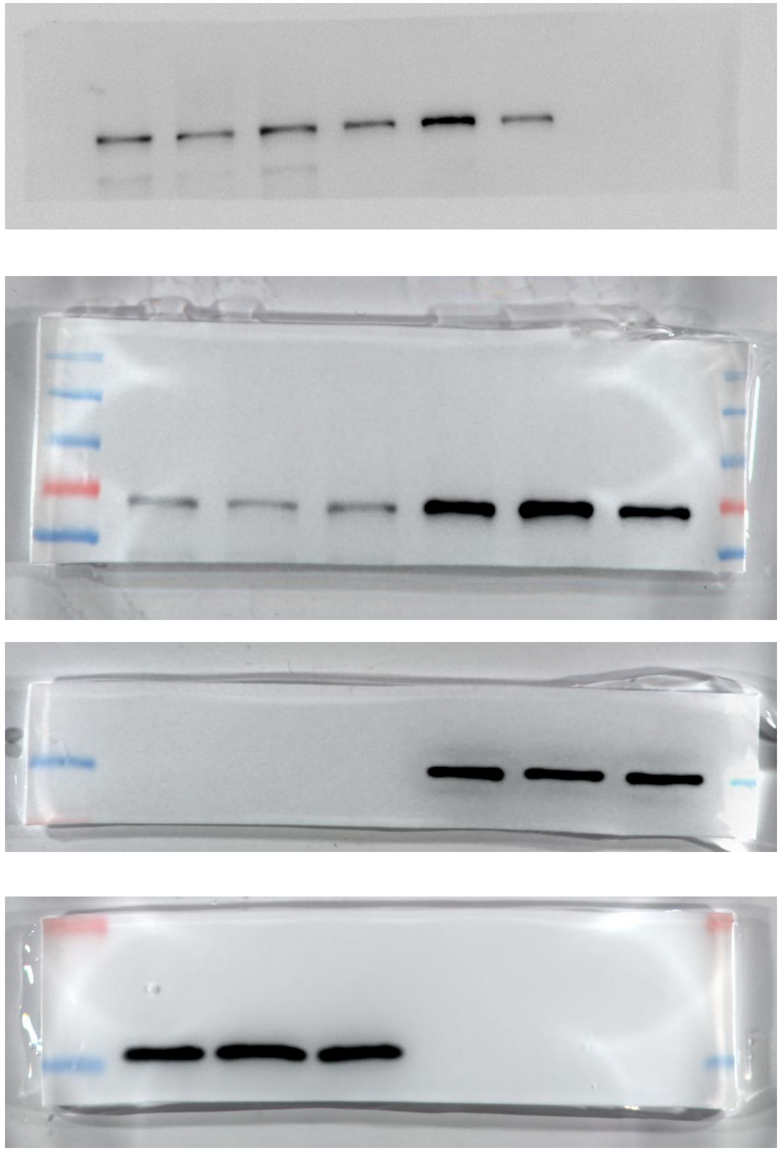

Supplementary Figure 4

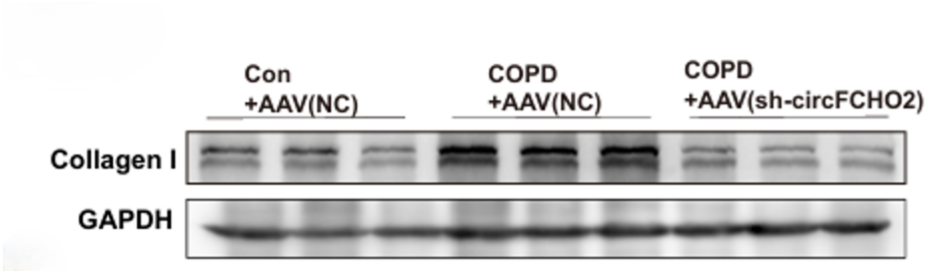

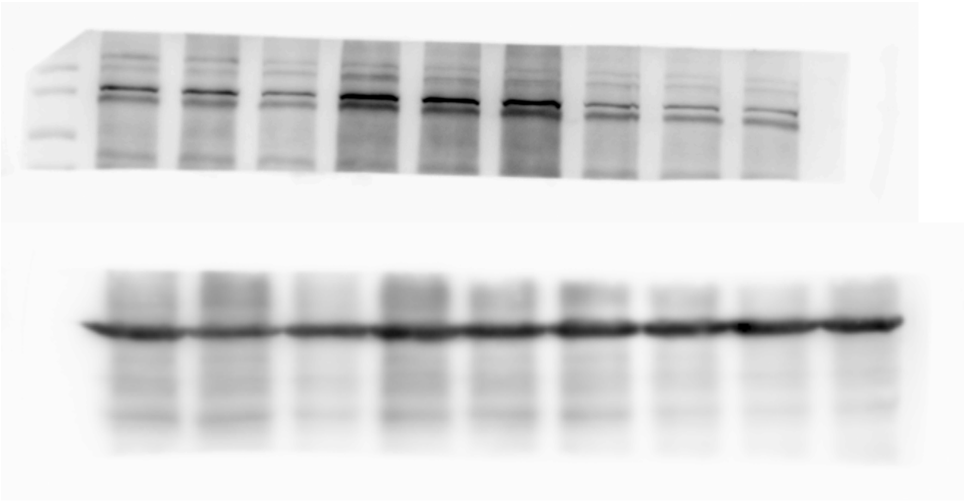

Supplementary Figure 5
